# A Novel Series of [1,2,4]Triazolo[4,3-a]Pyridine Sulfonamides as Potential Antimalarial Agents: In Silico Studies, Synthesis and In Vitro Evaluation

**DOI:** 10.3390/molecules25194485

**Published:** 2020-09-30

**Authors:** Veronika R. Karpina, Svitlana S. Kovalenko, Sergiy M. Kovalenko, Oleksandr G. Drushlyak, Natalya D. Bunyatyan, Victoriya A. Georgiyants, Vladimir V. Ivanov, Thierry Langer, Louis Maes

**Affiliations:** 1 Department of Pharmaceutical Chemistry, National University of Pharmacy, 53 Pushkinska str., 61002 Kharkiv, Ukraine; v.karpina01@gmail.com (V.R.K.); s.kovalenko.nphau@gmail.com (S.S.K.); vgeor@ukr.net (V.A.G.); 2 Department of Organic Chemistry, V.N. Karazin Kharkiv National University, 4 Svobody Sq., 61022 Kharkiv, Ukraine; kovalenko.sergiy.m@gmail.com (S.M.K.); vivanov5001@gmail.com (V.V.I.); 3 Federal State Autonomous Educational Institution of Higher Education I.M. Sechenov First Moscow State Medical University of the Ministry of Health of the Russian Federation (Sechenov University), 8-2 Trubetskaya str., Moscow 119991, Russia; ndbun@mail.ru; 4 Federal State Budgetary Institution ‘Scientific Centre for Expert Evaluation of Medicinal Products’ of the Ministry of Health of the Russian Federation, Petrovsky boulevard 8, bld. 2, Moscow 127051, Russia; 5 Department of Pharmaceutical Chemistry, University of Vienna, Althanstraße 14, A-1090 Vienna, Austria; thierry.langer@univie.ac.at; 6 Laboratory of Microbiology, Parasitology and Hygiene, University of Antwerp, Universiteitsplein 1, B-2610 Antwerp, Belgium; louis.maes@uantwerpen.be

**Keywords:** [1,2,4]triazolo[4,3-*a*]pyridines, sulfonamide, *Plasmodium falciparum*, antimalarial, falcipain-2, virtual screening, molecular docking

## Abstract

For the development of new and potent antimalarial drugs, we designed the virtual library with three points of randomization of novel [1,2,4]triazolo[4,3-*a*]pyridines bearing a sulfonamide fragment. The library of 1561 compounds has been investigated by both virtual screening and molecular docking methods using falcipain-2 as a target enzyme. 25 chosen hits were synthesized and evaluated for their antimalarial activity in vitro against *Plasmodium falciparum*. 3-Ethyl-*N*-(3-fluorobenzyl)-*N*-(4-methoxyphenyl)-[1,2,4]triazolo[4,3-*a*]pyridine-6-sulfonamide and 2-(3-chlorobenzyl)-8-(piperidin-1-ylsulfonyl)-[1,2,4]triazolo[4,3-*a*]pyridin-3(2*H*)-one showed in vitro good antimalarial activity with inhibitory concentration IC_50_ = 2.24 and 4.98 μM, respectively. This new series of compounds may serve as a starting point for future antimalarial drug discovery programs.

## 1. Introduction

Parasitic protozoal diseases have an enormous health, social and economic impact and are a particular problem in tropical regions of the world. These diseases include malaria, Chagas disease, human African trypanosomiasis, and leishmaniasis. The current chemotherapeutic options for these aggresive diseases can have severe side effects, may be ineffective, or even non-existent, and in cases where a drug treatment is available, resistance is emerging [1]. Malaria is a mosquito-borne infectious disease, which causes high morbidity and mortality rates worldwide. In 2018, malaria parasites threatened the lives of 228 million of people and caused 405,000 deaths. Most of these deaths occurred in the African Region (93%), followed by the South-East Asia Region (3.4%) and the Eastern Mediterranean Region (2.1%). Children under 5 years are particularly susceptible to malaria illness, infection and death. In 2018, malaria killed an estimated 272,000 (67% of total death) children under five years of age globally [2]. Six Plasmodium species are responsible for developing malaria in humans [3], however, the most lethal is *Plasmodium falciparum*. Thus, there is a constant need to discover and develop new antimalarial drugs, which are effective against multi-drug resistant parasites.

The biological activity of a compound, defined by the affinity of a small-molecule ligand towards the macromolecular receptor, can be explored by using in silico methods and completed with experimental methods. The constant discovery of novel anti-parasitic drug target proteins and free availability of their 3-D structures in protein data banks give an opportunity to find novel active inhibitors against previously known targets. Recently, virtual screening (VS) was routinely used as a supplement to or as a replacement for high-throughput screening in lead identification, due to virtual screening’s savings in time and money. Antimalarial studies of lead identification using VS were reported in the literature, either structure-based virtual screening (SBVS) [4,5] or ligand-based virtual screening (LBVS) [6]. The mentioned approaches are often used in combination with such methods as pharmacophore modeling, 3D QSAR and molecular docking techniques in order to prioritize molecules for testing and minimize the number of compounds to be investigated in biological screens. For example, Elumalai P. et al. [7] reported the discovery of novel inhibitors for the *P. falciparum* dihydroorotate dehydrogenase (PfDHODH), a key enzyme in the de novo pyrimidine biosynthesis pathway via a combination of the pharmacophore and SBVS approaches using 3D-QSAR pharmacophore models and docking studies. The in vitro antiplasmodial activity revealed three promising hits with IC_50_ ≤ 20 μM. The LBVS in combination with pharmacophore modeling has been used by Paul B. M. et al. [8] in the discovery of novel small molecule inhibitors of the plasmepsin aspartyl proteases, active against *P. falciparum*. V.K. Vyas et al. [9] claimed that structure-based pharmacophore modeling, virtual screening, docking and biological evaluation are the rational strategies for the identification of novel hits or leads with diverse chemical scaffolds. In their studies, in silico ADMET prediction was also conducted. In vivo antimalarial activity showed good potential for two compounds to become novel PfDHODH inhibitors.

As a follow up to our previous study [10], in collaboration with Laboratory of Microbiology, Parasitology and Hygiene, University of Antwerp, we paid attention to triazolopyridine scaffold bearing sulfonamide group as chemical entity with potential antimalarial activity. Triazolopyridines represents an important class of heteroaromatic compounds with a wide range of pharmaceutical and biological activities including antibacterial, antifungal [11], anti-inflammatory [12,13,14], herbicidal [15] and pesticidal [16], anticonvulsant [17], anxiolytic [18], and antipsychotic [19]. Triazolopyridine compounds bearing sulfonamide substituent are useful for the treatment of cystic fibrosis [20,21]. Recently, compound DSM265 with a triazolopyrimidine scaffold, which is bioisosteric to triazolopyridine scaffold, was found to be a PfDHODH inhibitor. This compound showed an encouraging safety profile in a Phase I trial and is currently in clinical development [22]. The sulfonamide functional group is widely used in medicinal chemistry because of its low toxicity and excellent biological activity. Sulfadoxine [23], Sulfolene and Sulfadiazine [24] are among the effective malaria drugs containing sulfonamide groups attached to a heterocyclic ring. A large number of sulfonamide derivatives were obtained by alkylation of the nitrogen of the sulfamide group. In particular, there is information about the antiprotozoal activity of sulfonamide derivatives. In 2007, Da Silva et al. demonstrated that synthetically prepared *N*-quinolin-8-yl-arylsulfonamides and their complexes show significant anti-parasitic activity in vitro against *Trypanosoma cruzi*, *Leishmania chagasi* and *L. Amazonensis* [25]. Sulfonamides are also used in combination with pyrimethamine for the treatment of drug-resistant malaria and for toxoplasmosis [26]. The literature describes several other sulfonamides with antimalarial activity [27,28,29]. Given the threat of antimalarial drug resistance, there is an increasing need to develop alternative treatments, novel agents, that act via a unique mechanism of action relative to currently used drugs.

Target identification is one of the most difficult tasks in developing new antimalarial drugs. The complex life cycle associated with *Plasmodium falciparum* provides a great number of targets, which can be explored to discover new drugs for the treatment of malaria. During its life span, a parasite plays an important role in metabolite synthesis, membrane transport and haemoglobin degradation. The targets, which are involved in these processes, can be used to inhibit parasitic growth by their inhibition. The best-characterized *P. falciparum* cysteine proteases are the falcipains that share sequence identity and other features with papain family cysteine proteases [30]. Among the four *P. falciparum* cysteine proteases, FP-2 is the most intensely studied enzyme and it appears to be the essential food vacuolar hemoglobinases [31]. Thus, inhibiting this target could prevent haemoglobin hydrolysis, which definitely hindered parasitic growth [32]. Indeed, any stoppage or even hindrance of the ability of *P. falciparum* to degrade the human haemoglobin through its cysteine protease kills *P. falciparum* [33], most especially at the Trophozoite stage of its development, when the FP-2 plays an important role in the parasite life cycle. [34]. This makes FP-2 an important antimalarial drug target because its inhibition kills the malaria-causing *P. falciparum.* At present, the main classes of known falcipain-2 inhibitors are peptides or peptidomimetic-bearing pharmacophores of cysteine protease inhibitors, such as vinyl sulfones, halomethyl ketones, and aldehydes. Furthermore, some nonpeptidic compounds have been identified as inhibitors of FP-2, such as isoquinolines, thiosemicarbazones, and chalcones [31].

**Scheme 1:**
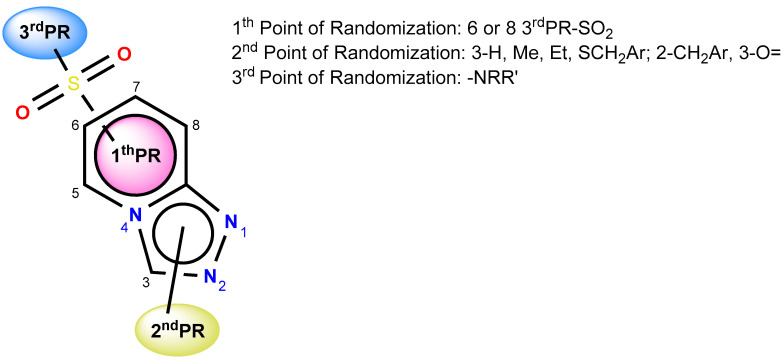
Points of randomization of the library of [1,2,4]triazolo[4,3-*a*]pyridines.

With the aim of developing novel and potent antimalarial compounds, we designed the library of [1,2,4]triazolo[4,3-*a*]pyridines, bearing a sulfonamide fragment. Three points of randomization were provided: the 1st was the position of sulfonamide substituent (6 or 8), the 2nd was substituent in triazole ring (3-H or lower alkyl, 2-benzyl-3(2*H*)-one, 3-benzylthio) and the 3rd was variation in sulfonamide substituent (Scheme 1).

An SBVS approach in combination with pharmacophore modeling using LigandScout was applied for library analysis. Subsequently, the hits with good pharmacophoric fit values were docked into the crystal structure of FP-2 using AutoDock Vina and filtered accordingly. Following this, 25 hits were selected for the synthesis. All synthesized compounds were analyzed in vitro for chloroquine-resistant *P. falciparum* 2/K 1-strain.

## 2. Results and Discussion

### 2.1. Pharmacophore Model Generation, Virtual Screening and Molecular Docking

We selected the complex of Falcipain-2 with the epoxysuccinate E64 for our molecular modeling studies (pdb entry 3BPF). Cocrystallized FP2-E64 consists of residues 1–241 of the mature enzyme. The active site of enzyme is located in a cleft between the structurally distinct domains of the papain-like fold. The sulfhydryl group in the active site of cysteine protease FP2 forms a covalent, irreversible hemithioketal with the E64 epoxide group [35,36].

**Figure 1:**
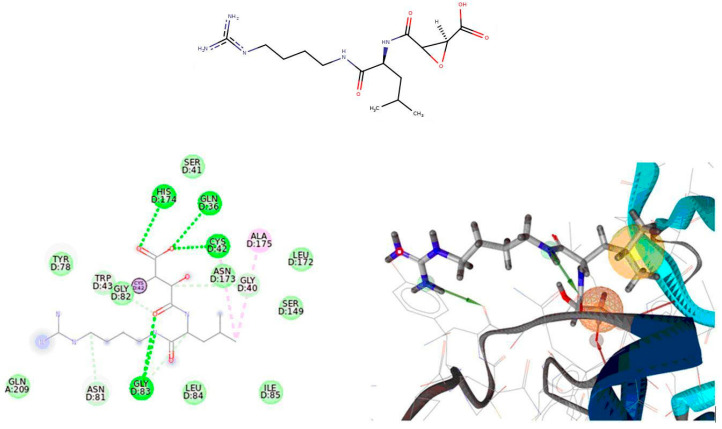
Ligand E64 and pharmacophore model generated within LigandScout from falcipain-2/epoxysuccinate E64 complex for D chain in 2D and 3D view.

The mentioned complex was then used as a starting point for an extensive investigation of the chemical features important for ligand–protein interaction, and thus for pharmacophore generation by means of the software LigandScout. LigandScout generates structure-based pharmacophore models with the most important chemical features such as hydrogen bonds, charge interactions and hydrophobic areas. While hydrogen bond features are defined by direction and distance constraints, hydrophobic interactions and ionizable areas have a distance constraint only. In addition to LigandScout’s supported chemical features, the program analyzes the shape of the active site and places excluded volume spheres in positions that are sterically claimed by the macromolecular environment. Initially, the re-docking methodology was performed with AutoDock Vina using the ligand from each chain. The root–mean–square deviation (RMSD) values of the heavy atoms in the re-docked ligands were 2.55, 2.65, 2.63, 2.54 Å for chains A, B, C and D respectively. This re-docking procedure showed that there are no significant differences in the crystallographic and docked ligand–protein complexes. Besides, the analysis of docking results was performed based on the main ranking criteria meaning Binding Energy (or Affinity, kcal/mol). As for Binding Affinity Score, it is a much-formalized parameter, which is calculated and used in different ways in different programs. The BAS parameter has been calculated with the LigandScout default criteria. For further studies, we chose the chain D with the lowest RMSD value. The pharmacophore model was generated based on the mentioned crystal structure and contained eight features: four hydrogen bond donors (HBD), three hydrogen bond acceptors (HBA), one hydrophobic group (Figure 1). Moreover, the software automatically increases the selectivity of the generated pharmacophore with the excluded volumes. LigandScout analyzes the shape of the active site and places excluded volume spheres in positions that are sterically claimed by the macromolecular environment. This process ensures that molecules retrieved via virtual screening match the sterical requirements of the active site, while simultaneously drastically increasing selectivity [37].

As one can see from Figure 1, the E64–protein complex characterizes several important interactions of the active site. Among them the hydrogen bond acceptors are Cys42, Gln36, His174 and Gly83, and hydrogen bond donors are Ala175 and Asn173.

Then the in-house library of 1561 compounds was filtered by virtual screening procedure using the generated model. A total of 150 compounds with the highest pharmacophore-fit score, well-fitted to the pharmacophore model, were chosen for the docking calculations. Afterwards, the poses of every ligand were visualized and the protein–ligand binding energies were obtained. The most important ligand–protein binding properties, as well as corresponding physico-chemical parameters of the aforementioned hits, are presented in Table 1. Then, the structures were ranked and the best 25 hits were chosen for synthesis and biological assay. The main ligand–protein binding properties and corresponding physico-chemical parameters of the mentioned hits are presented in Table 1.

The retrieved hits showed interactions with key amino acid residues near the active site, which included GLN36D, GLY83D, ALA175D, ILE85D, LEU84D and CYS42D. The binding modes and docking scores of retrieved hits were compared with the reference inhibitor E64 in order to evaluate the docking results, and it was found that all 25 compounds showed better interactions than E64 in the binding cavity of the enzyme. The binding energy values against the FP-2 ranged from 15.9 to 14 kcal/mol when compared to the reference ligand with −10.80 kcal/mol.

**Figure 2:**
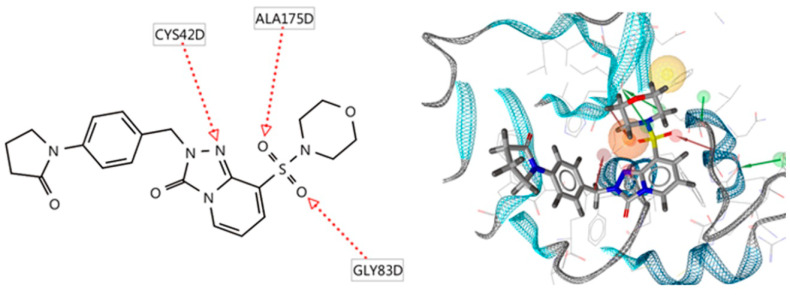
Binding site interactions of **13e** in FP-2 enzyme proposed by docking studies in 2D (**left side**) and 3D (**right side**) view. HBA—red vectors.

**Figure 3:**
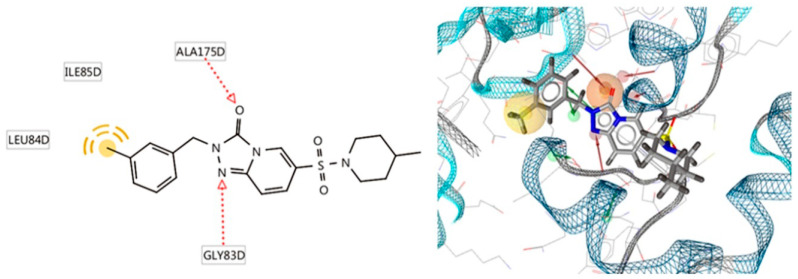
Binding site interactions of **13g** in FP-2 enzyme proposed by docking studies in 2D (**left side**) and 3D (**right side**) view. HBA—red vectors, hydrophobic group-yellow area.

Compounds **13e** and **13g** have the highest binding energies of −15.9 and −15.6 kcal/mol, respectively. The binding site interactions between **13e** and FP-2 are illustrated in Figure 2 in 2D and 3D view. The triazolopyridine ring system forms a hydrogen-bond between Cys42 and the nitrogen N1. Two sulfonamide oxygen atoms formed H-bonds with the H atoms of Ala175 and Gly83–NH_2_ groups. The 3-Methylbenzyl group of **13g** compound was in contact with the hydrophobic pocket formed by Ile85 and Leu84. A 2D and 3D view of binding site interactions are illustrated in Figure 3. Non-hydrophobic interaction included H-bonds between the Ala175/N1 atom and the Gly83/oxygen atom of the triazolopyridine ring system.

The review of other compounds showed the following binding interactions:

- Oxygen atoms of sulfonamide group were most commonly involved in interactions with Gln36, Cys42, Gly83 amino acids for all compounds, except **13i** and **15b**;
- N1 atom of fused triazolopyridine ring system was involved in hydrogen bonding with Ala175 (**13a**, **13b**, **13d**, **13c**), Cys42 (**15e**, **15c**, **15a**, **15b**), Gly83 (**15d**, **13i**) and Gln36 (**8e**);
- N2 atom of fused triazolopyridine ring system was involved in hydrogen bonding with Gly83 (**8a**, **8b**, **8c**, **15f**, **15d**) and Gln36 (**15e**,**15c**,**15a**);
- Oxygen atom in the 3 position of fused triazolopyridine ring system was involved in hydrogen bonding with Gly83 (**13b**, **15d**, **13f**, **13h**, **13j**) and Ala175 (**13i**);
- Amino acids such as Ile 85, Leu 84 were most commonly involved in hydrophobic interactions for all compounds, except **8a**, **8b**, **8c**, **15f**, **15e**, **15c**, **15a**.

The docking study suggested that these interactions contributed to the potent binding of the retrieved hits. Based on the interaction results, all 25 compounds, including chloroquine, showed interactions with at least two main amino acid residues of the enzyme active site. It is to be noted that none of the compounds from the library showed interaction with His174.

### 2.2. Chemistry

**Scheme 2:**
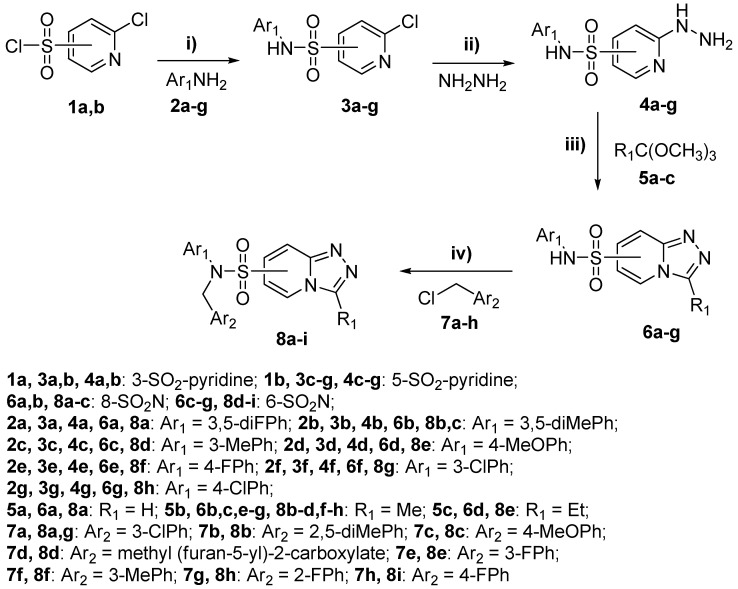
Synthesis of compounds **8a**–**i**. (**i**) Triethylamine, dioxane, 60 °C, 3 h; (**ii**) N_2_H_4_, *i*-propanol, reflux, 4 h; (**iii**) DMF, reflux, 16 h; (**iv**) K_2_CO_3_, DMF, 90 °C, 2 h.

**Scheme 3:**
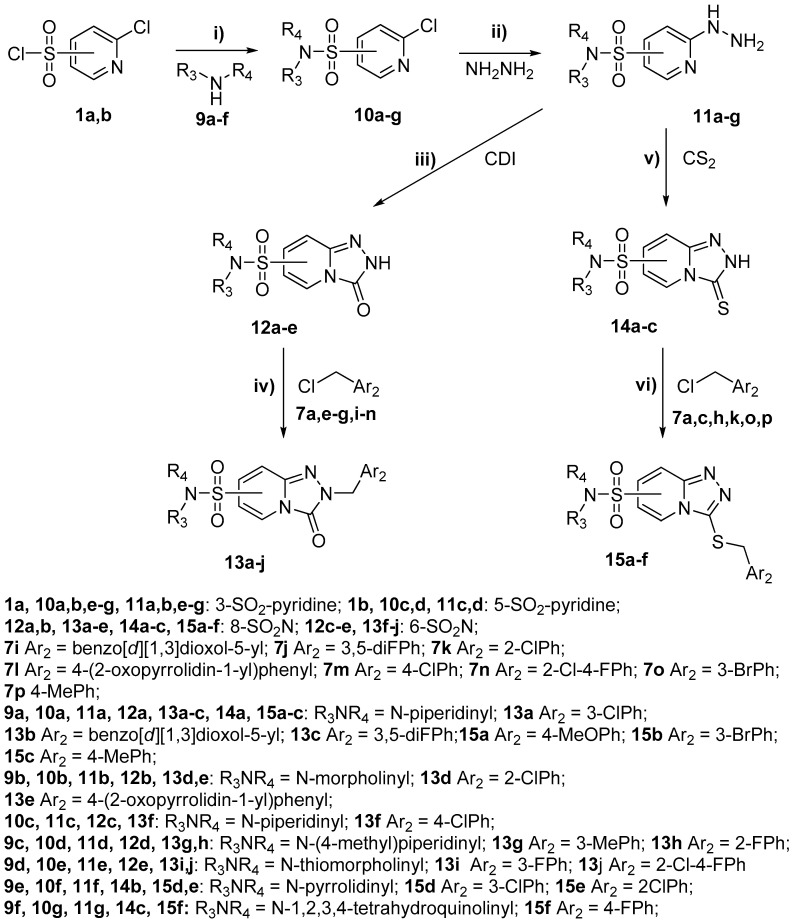
Synthesis of compounds **13a**–**j** and **15a**–**f**. (**i**) Dioxane, 60 °C, 1 h; (**ii**) N_2_H_4_, *i*-propanol, reflux, 4 h; (**iii**) CDI, dioxane, reflux, 8 h; (**iv**) K_2_CO_3_, DMF, 100 °C, 2 h; (**v**) CS_2_, triethylamine, DMF, 90 °C, 6 h; (**vi**) triethylamine, DMF, 80 °C, 2 h.

The reaction sequences started from commercially available 2-chloropyridine-3-sulfonyl chloride **1a** or 2-chloropyridine-5-sulfonyl chloride **1b** to provide the first randomization point in the position of the sulfonamide substituent (Scheme 2 and Scheme 3). The chosen strategy of synthesis involved the further introduction of a benzyl substituent at the nitrogen atom of sulfonamide group to create the new randomization point, therefore, sulfonyl chlorides **1a,b** were treated by primary anilines **2a**–**g** to produce 2-chloro-*N*-(aryl)pyridinesulfonamides **3a**–**g**. The chlorine atom in compounds **3a**–**g** was replaced by action of hydrazine hydrate in *i*-propanol to provide 2-hydrazinyl-*N*-(aryl)pyridinesulfonamides **4a**–**g**. Synthesis of [1,2,4]triazolo[4,3-*a*]pyridinesulfonamides **6a**–**g** was performed by the reaction of *ortho*-esters **5a**–**c** with compounds **4a**–**g** in DMF with reflux for 16 h. Further, benzyl substituent was introduced by alkylation of compounds **6a**–**g** by action of benzyl chlorides **7a**–**h** due to the nucleophilic properties of NHSO-group. As a result, we obtained novel *N,N*-disubstituted [1,2,4]triazolo[4,3-*a*]pyridinesulfonamides **8a**–**i** (Scheme 2), (Table 2).

Syntheses of novel 6- and 8-sulfonamido[1,2,4]triazolo[4,3-*a*]pyridin-3-one derivatives **13a–j**, and their 3-thio analogues **15a–f** were performed by analogy with the known synthetic pathway [20,38] shown in Scheme 3. Since the alkylation of unsubstituted sulfonamido[1,2,4]triazolopyridin-3-ones, and especially 3-thioxo[1,2,4]triazolo[4,3-*a*]pyridines, can lead to two different alkylation products (with substituent at the sulfonamide nitrogen or at the nitrogen at 2-position of the triazole moiety or at the sulfur atom), therefore, in order to obtain target products with the only possible and strictly defined structure, secondary amines **9a–f** were chosen to prepare sulfonamides **10a–g**. This excluded further alkylation of the sulfonamide group. The reactions conditions for syntheses of 2-chloro-*N*-substitutedpyridinesulfonamides **10a–g** and 2-hydrazinyl-*N*-substitutedpyridinesulfonamides **11a–g** (Scheme 3) were similar to the reaction conditions described above (Scheme 2). Sulfonamido[1,2,4]triazolopyridin-3-ones **12a–e** were prepared by cyclization of the corresponding hydrazines **11a–e** with an excess of CDI in anhydrous dioxane with reflux for 8 h. Subsequent alkylation was produced by the action of benzyl chlorides **7a,e–g,i–n** in DMF at 100 °C in the presence of excess of K_2_CO_3_ and resulted in the formation of 2-benzyl-sulfonamido[1,2,4]triazolopyridin-3-ones **13a–j**. 3-Thioxo-sulfonamido[1,2,4]triazolopyridines **14a–c** were synthesized by the reaction of CS_2_ with 2-hydrazinyl-*N*-substitutedpyridinesulfonamides **11a,f,g** in DMF with the presence of triethylamine. Further alkylation of compounds **14a–c** was carried out by the action of benzyl chlorides **7a,c,h,k,o,p** in DMF at 80 °C in the presence of triethylamine, and gave 3-thio-sulfonamido[1,2,4]triazolo[4,3-*a*]pyridines **15a–f** with high yields (Scheme 3), (Table 3).

All compounds were characterized by elemental analysis and ^1^H-NMR data. Target compounds **8a**–**i**, **13a**–**j** and **15a**–**f** were additionally characterized by ^13^C-NMR and LC/MS data. (The NMR and LC/MS data may be accessed in the Supplementary Materials).

### 2.3. Antiprotozoal Activity Assay

The newly synthesized compounds were evaluated for their in vitro antiprotozoal inhibitory activity against the *Plasmodium falciparum* 2/K chloroquine-resistant strain by determining the concentration required to inhibit parasite development by 50% (IC_50_ values). The protocols are described in the experimental section. The IC_50_ values and the cytotoxicity against human fibroblast of all the new compounds are listed in Table 4.

Chloroquine, a clinical candidate with an IC_50_ value of 0.02 μM, was used as a reference compound. Two of the 25 compounds were active against *Plasmodium falciparum* 2/K chloroquine-resistant strain at a concentration of 4.98 μM for 6e and 2.24 μM for 10a. Seven compounds showed an inhibitory concentration ranging from 8 to 10 μM, and six compounds showed an inhibitory concentration ranging from 10 to 14 μM.

Two of the 25 tested samples showed cytotoxicity against MRC-5 cells at a concentration of IC_50_ = 48.50 μg/mL for compound 8b and 18.19 μg/mL for compound 8e. At the same time, the 23 other tested samples did not show cytotoxicity against MRC-5 cells (IC_50_ > 64 μg/mL), resulting in a high selectivity.

The drug-like properties of all compounds including the two main active compounds 13a and 8e, according to the Lipinski Rule of Five, are summarized in Table 5. Based on these values, we can confirm that compounds 13a and 8e obey the Lipinski Rule of Five, which states that, for a good absorption and permeation, compounds should have a MW less than 500 Da, no more than 5 HBDs, 10 HBAs, LogP value (partition coefficient between octanol and water) from 2 to 5 and a TPSA less than 140 Å^2^ [39,40,41]. Based on the practical data, the compounds **8e** and **13a** are moderately soluble in DMSO/Water.

In addition, in silico ADMET parameters, such as gastrointestinal absorption, P-glycoprotein substrate, blood brain barrier (BBB) permeability and CYP450 inhibition were predicted using Swiss-ADME [42].

The ability of active compounds to pass the blood–brain barrier is absent, with this being correlated with a reduced risk of developing side effects in the central nervous system upon administration. Our active compounds have no ability to pass the BBB. P-glycoprotein (Pgp) is a plasma membrane protein, which acts as a localized drug transport mechanism, actively exporting drugs out of the cell. Being a substrate to Pgp, compounds can generate a lot of drug–drug interactions and seriously modify the bioavailability and safety of a certain drug [43]. Compound **13a** does not seem to be a substrate for Pgp, while **8e** seems to be a substrate. A high gastrointestinal absorption was also predicted for active compounds. Moreover, the studied compounds act as non-inhibitors of CYP1A2, CYP2D6, CYP3A4, so drug–drug interactions with compounds that are metabolized by this isoform of CYP450 superfamily are less likely to appear.

## 3. Materials and Methods

### 3.1. Pharmacophore Model Generation, Virtual Screening and Molecular Docking

Pharmacophore models are often used together with other methods to further increase the number of active molecules in the hit list via the application of a consensus approach [44]. In the present work, a combination of pharmacophore-based and docking approaches was used. The pharmacophore model was constructed using LigandScout software [45], which contains all the chemical features’ information, such as hydrogen bond donors, hydrogen bond acceptors, and hydrophobic residues within the binding site sphere of the receptor [37,44]. The crystal structure of FP-2 bound to the epoxysuccinate E64 inhibitor with high resolution was downloaded from the Protein Data Bank [46] (PDB entry: 3bpf). The structure of the protein was imported into LigandScout software for structural alignment with the protein preparation wizard to ensure its structural correctness. Preparation of protein: the addition of hydrogens and removal of water molecules were done with LigandScout default settings. The respective crystal ligands of E64 in every of four chains (A, B, C, D) were checked to ensure they were correctly depicted, for example, whether bond orders are correct and were subjected to energy minimization. After that, ligands were re-docked by AutoDock Vina [47] into the active sites of the target protein (3bpf) to validate the docking protocol. Chain D was selected for further studies because it had the lowest RMSD value. The structure-based method was used to construct the 3D pharmacophore model.

The generated 3D pharmacophore model was used as a 3D search query for retrieving potent hit molecules from our in-house database. The AutoDock Vina software was used for molecular docking simulations and LigandScout with Biovia Discovery Studio Visualizer for visualizing the obtained results.

### 3.2. Chemistry. General Information

All NMR spectra were recorded on a Varian MR-400 spectrometer (Varian, Inc., Walnut Creek, CA, USA) with standard pulse sequences operating at 200, 300 or 400 MHz for ^1^H-NMR, and 75 MHz or 100 MHz for ^13^C-NMR. For all NMR spectra, DMSO-d6 was used as a solvent. Chemical shift values are referenced to residual protons (δ 2.49 ppm) and carbons (δ 39.6 ppm) of the solvent as an internal standard. Elemental analysis was performed on EuroEA-3000 CHNS-O Analyzer (Euro Vector, Milan, Italy). Melting points were measured with a Buchi B-520 melting point apparatus (Buchi AG, Flawil, Switzerland). LC/MS spectra were recorded with ELSD Alltech 3300 liquid chromatograph (Buchi AG, Flawil, Switzerland) equipped with a UV detector (λ_max_ 254 nm), API-150EX mass-spectrometer and using a Zorbax SB-C18 column, Phenomenex (100 × 4 mm) Rapid Resolution HT Cartrige 4.6 × 30 mm, 1.8-Micron. Elution started with 0.1 M solution of HCOOH in water and ended with 0.1 M solution of HCOOH in acetonitrile used a linear gradient at a flow rate of 0.15 mL/min and an analysis cycle time of 25 min. UV/Vis spectra of solutions in CH3CN were recorded on a Specord 200 spectrometer (Analytik Jena AG, Jena, Germany). IR spectra in KBr pellets were recorded on a Bruker Vertex 70 FTIR spectrometer (Bruker Optik GmbH, Ettlingen, Germany). The purity of compounds was checked by thin layer chromatography, which was performed on Silufol UV254 aluminum plates precoated with silica gel with EtOAc:Hex (1:2, 1:4), EtOAc, EtOAc:MeOH (9:1) as eluents.

Starting 2-chloropyridinesulfonyl chlorides **1a,b** were purchased from Life Chemicals (Kyiv, Ukraine). Aniline**s 2a**–**g**, benzyl chlorides **7a**–**p**, secondary amines **9a**–**f**, other reagents and solvents are commercially available, were reagent grade and were used without further purification. Silica gel (40–60 μm) from Merck was used for column chromatography.

### 3.3. General Procedures for the Synthesis

#### 3.3.1. Synthesis of 2-Chloro-*N*-(aryl)Pyridinesulfonamides **3a**–**g**. General Procedure A

Primary aniline **2a**–**g** (0.12 mol) and triethylamine (15 mL, 0.12 mol) were added to a stirred solution of sulfonyl chloride 1a,b (21.2 g, 0.1 mol) in dry dioxane (100 mL) for 5 min at room temperature. The reaction mixture was heated at 60 °C for 3 h. After cooling, the reaction mixture was diluted with water (500 mL). The precipitate that formed was filtered and recrystallized from *i*-propanol. Yields of 2-chloro-*N*-(aryl)pyridinesulfonamides **3a**–**g** were 70–82%.

*2-Chloro-N-(3,5-difluorophenyl)pyridine-3-sulfonamide* (**3a**). According to General Procedure A, 2-chloropyridine-3-sulfonyl chloride **1a** (21.2 g, 0.1 mol) and 3,5-difluoroaniline **2a** (15.5 g, 0.12 mol) yielded 2-chloro-*N*-(3,5-difluorophenyl)pyridine-3-sulfonamide **3a** (21.9 g, 72%), as a white solid, m.p. 196–198 °C. ^1^H-NMR spectrum δ, ppm (*J*, Hz): 6.31–6.37 (m, 1H, Ph H-4), 6.55 (dd, *J* = 11.4, *J* = 4.6, 2H, Ph H-2,6), 7.63 (dd, *J* = 8.4, *J* = 6.8, 1H, H-5), 8.04 (dd, *J* = 8.4, *J* = 1.5, 1H, H-6), 8.73 (dd, *J* = 6.8, *J* = 1.5, 1H, H-4), 10.20 (s, 1H, NH). Anal. calcd. for C_11_H_7_ClF_2_N_2_O_2_S %: C 43.36; H 2.32; N 9.19; S 10.52. Found, %: C 43.22; H 2.31; N 9.23; S 10.49.

*2-Chloro-N-(3,5-dimethylphenyl)pyridine-3-sulfonamide* (**3b**). According to General Procedure A, 2-chloropyridine-3-sulfonyl chloride **1a** (21.2 g, 0.1 mol) and 3,5-dimethylaniline **2b** (14.5 g, 0.12 mol) yielded 2-chloro-*N*-(3,5-dimethylphenyl)pyridine-3-sulfonamide **3b** (24.0 g, 81%), as a white solid, m.p. 174–176 °C. ^1^H-NMR spectrum δ, ppm (*J*, Hz): 2.24 (s, 6H, 2 CH_3_), 6.50 (s, 1H, Ph H-4), 6.71 (s, 2H, Ph H-2,6), 7.64 (dd, *J* = 8.4, *J* = 6.8, 1H, H-5), 8.04 (dd, *J* = 8.4, *J* = 1.5, 1H, H-6), 8.73 (dd, *J* = 6.8, *J* = 1.5, 1H, H-4), 10.20 (s, 1H, NH). Anal. calcd. for C_13_H_13_ClN_2_O_2_S %: C 52.61; H 4.42; N 9.44; S 10.80. Found, %: C 52.78; H 4.41; N 9.47; S 10.76.

*2-Chloro-N-(3-methylphenyl)pyridine-5-sulfonamide* (**3c**). According to General Procedure A, 2-chloropyridine-5-sulfonyl chloride **1b** (21.2 g, 0.1 mol) and 3-methylaniline **2c** (12.8 g, 0.12 mol) yielded 2-chloro-*N*-(3-methylphenyl)pyridine-5-sulfonamide **3c** (21.5 g, 76%), as a white solid, m.p. 171–173 °C. ^1^H-NMR spectrum δ, ppm (*J*, Hz): 2.20 (s, 3H, CH_3_), 6.82–6.98 (m, 3H, Ar H), 7.12 (t, *J* = 7.6, 1H, Ph H-5), 7.71 (d, *J* = 8.0, 1H, H-3), 8.11 (dd, *J* = 8.0, *J* = 2.2, 1H, H-4), 8.70 (d, *J* = 2.2, 1H, H-6), 10.42 (s, 1H, NH). Anal. calcd. for C_12_H_11_ClN_2_O_2_S %: C 50.98; H 3.92; N 9.91; S 11.34. Found, %: C 51.16; H 3.93; N 9.88; S 11.29.

*2-Chloro-N-(4-methoxyphenyl)pyridine-5-sulfonamide* (**3d**). According to General Procedure A, 2-chloropyridine-5-sulfonyl chloride **1b** (21.2 g, 0.1 mol) and 4-methoxyaniline **2d** (14.8 g, 0.12 mol) yielded 2-chloro-*N*-(4-methoxyphenyl)pyridine-5-sulfonamide **3d** (24.5 g, 82%), as a white solid, m.p. 182–183 °C. ^1^H-NMR spectrum δ, ppm *(J*, Hz): 3.67 (s, 3H, OCH_3_), 6.80 (d, *J* = 8.0, 2H, Ph H-3,5), 6.98 (d, *J* = 8.0, 2H, Ph H-2,6), 7.70 (d, *J* = 8.0, 1H, H-3), 8.11 (dd, *J* = 8.0, *J* = 2.2, 1H, H-4), 8.54 (d, *J* = 2.2, 1H, H-6), 10.19 (s, 1H, NH). Anal. calcd. for C_12_H_11_ClN_2_O_3_S %: C 48.25; H 3.71; N 9.38; S 10.73. Found, %: C 48.06; H 3.73; N 9.41; S 10.69.

*2-Chloro-N-(4-fluorophenyl)pyridine-5-sulfonamide* (**3e**). According to General Procedure A, 2-chloropyridine-5-sulfonyl chloride **1b** (21.2 g, 0.1 mol) and 4-fluoroaniline **2e** (13.3 g, 0.12 mol) yielded 2-chloro-*N*-(4-fluorophenyl)pyridine-5-sulfonamide **3e** (20.9 g, 73%), as a white solid, m.p. 202–203 °C. ^1^H-NMR spectrum δ, ppm *(J*, Hz): 7.02–7.18 (m, 4H, Ar H), 7.72 (d, *J* = 8.0, 1H, H-3), 8.07 (dd, *J* = 8.0, *J* = 2.2, 1H, H-4), 8.63 (d, *J* = 2.2, 1H, H-6), 10.48 (br. s, 1H, NH). Anal. calcd. for C_11_H_8_ClFN_2_O_2_S %: C 46.08; H 2.81; N 9.77; S 11.18. Found, %: C 45.92; H 2.82; N 9.80; S 11.22.

*2-Chloro-N-(3-chlorophenyl)pyridine-5-sulfonamide* (**3f**). According to General Procedure A, 2-chloropyridine-5-sulfonyl chloride **1b** (21.2 g, 0.1 mol) and 3-chloroaniline **2f** (15.3 g, 0.12 mol) yielded 2-chloro-*N*-(3-chlorophenyl)pyridine-5-sulfonamide **3f** (21.2 g, 70%), as a white solid, m.p. 191–193 °C. ^1^H-NMR spectrum δ, ppm *(J*, Hz): 7.02–7.18 (m, 3H, Ar H), 7.28 (t, *J* = 7.6, 1H, Ph H-5), 7.72 (d, *J* = 8.0, 1H, H-3), 8.12 (dd, *J* = 8.0, *J* = 2.2, 1H, H-4), 8.74 (d, *J* = 2.2, 1H, H-6), 10.80 (br. s, 1H, NH). Anal. calcd. for C_11_H_8_Cl_2_N_2_O_2_S %: C 43.58; H 2.66; N 9.24; S 10.58. Found, %: C 43.42; H 2.65; N 9.20; S 10.62.

*2-Chloro-N-(4-chlorophenyl)pyridine-5-sulfonamide* (**3g**). According to General Procedure A, 2-chloropyridine-5-sulfonyl chloride **1b** (21.2 g, 0.1 mol) and 4-chloroaniline **2g** (15.3 g, 0.12 mol) yielded 2-chloro-*N*-(4-chlorophenyl)pyridine-5-sulfonamide **3g** (21.2 g, 70%), as a white solid, m.p. 207–209 °C. ^1^H-NMR spectrum δ, ppm *(J*, Hz): 7.11 (d, *J* = 8.0, 2H, Ph H-3,5), 7.32 (d, *J* = 8.0, 2H, Ph H-2,6), 7.71 (d, *J* = 8.0, 1H, H-3), 8.09 (dd, *J* = 8.0, *J* = 2.2, 1H, H-4), 8.70 (d, *J* = 2.2, 1H, H-6), 10.70 (s, 1H, NH). Anal. calcd. for C_11_H_8_Cl_2_N_2_O_2_S %: C 43.58; H 2.66; N 9.24; S 10.58. Found, %: C 43.65; H 2.67; N 9.22; S 10.56.

#### 3.3.2. Synthesis of *N*-(aryl)-2-Hydrazinylpyridinesulfonamides **4a**–**g**. General Procedure B

The corresponding 2-chloro-*N*-(aryl)pyridinesulfonamide **3a**–**g** (50 mmol) was added to a solution of hydrazine hydrate (13 mL, 200 mmol) in *i*-propanol (50 mL). The reaction mixture was refluxed for 4 h. After cooling, the reaction mixture was diluted with water (200 mL). The precipitate that formed was filtered and recrystallized from *i*-propanol. Yields of *N*-(aryl)-2-hydrazinylpyridinesulfonamides **4a**–**g** were 84–91%.

*N-(3,5-difluorophenyl)-2-hydrazinylpyridine-3-sulfonamide* (**4a**). According to General Procedure B, 2-chloro-*N*-(3,5-difluorophenyl)pyridine-3-sulfonamide **3a** (15.2 g, 50 mmol) yielded *N*-(3,5-difluorophenyl)-2-hydrazinylpyridine-3-sulfonamide **4a** (13.1 g, 87%), as a white solid, m.p. 214–216 °C. ^1^H-NMR spectrum δ, ppm (*J*, Hz): 4.49 (br. s, 2H, NH_2_), 6.31–6.37 (m, 1H, Ph H-4), 7.10 (d, *J* = 6.6, 2H, Ph H-2,6), 7.40 (dd, *J* = 8.4, *J* = 6.8, 1H, H-5), 7.95 (dd, *J* = 8.4, *J* = 1.5, 1H, H-6), 8.25 (dd, *J* = 6.8, *J* = 1.5, 1H, H-4), 8.80 (br. s, 1H, NH), 10.20 (s, 1H, SO_2_NH). Anal. calcd. for C_11_H_10_F_2_N_4_O_2_S %: C 44.00; H 3.36; N 18.66; S 10.68. Found, %: C 43.87; H 3.37; N 18.73; S 10.65.

*N-(3,5-dimethylphenyl)-2-hydrazinylpyridine-3-sulfonamide* (**4b**). According to General Procedure B, 2-chloro-*N*-(3,5-dimethylphenyl)pyridine-3-sulfonamide **3b** (14.8 g, 50 mmol) yielded *N*-(3,5-dimethylphenyl)-2-hydrazinylpyridine-3-sulfonamide **4b** (12.4 g, 85%), as a white solid, m.p. 201–203 °C. ^1^H-NMR spectrum δ, ppm (*J*, Hz): 2.24 (s, 6H, 2 CH_3_), 4.59 (br. s, 2H, NH_2_), 6.50 (s, 1H, Ph H-4), 6.66 (dd, *J* = 8.4, *J* = 6.8, 1H, H-5), 6.71 (s, 2H, Ph H-2,6), 7.50 (dd, *J* = 6.8, *J* = 1.5, 1H, H-4), 7.87 (dd, *J* = 8.4, *J* = 1.5, 1H, H-6), 8.46 (br. s, 1H, NH), 9.75 (s, 1H, SO_2_NH). Anal. calcd. for C_13_H_16_N_4_O_2_S %: C 53.41; H 5.52; N 19.16; S 10.97. Found, %: C 53.26; H 5.54; N 19.24; S 11.01.

*2-Hydrazinyl-N-(3-methylphenyl)pyridine-5-sulfonamide* (**4c**). According to General Procedure B, 2-chloro-*N*-(3-methylphenyl)pyridine-5-sulfonamide **3c** (14.1 g, 50 mmol) yielded 2-hydrazinyl-*N*-(3-methylphenyl)pyridine-5-sulfonamide **4c** (11.7 g, 84%), as a white solid, m.p. 187–189 °C. ^1^H-NMR spectrum δ, ppm (*J*, Hz): 2.18 (s, 3H, CH_3_), 4.35 (br. s, 2H, NH_2_), 6.67 (d, *J* = 8.0, 1H, H-3), 6.78–6.90 (m, 3H, Ar H), 7.09 (t, *J* = 7.6, 1H, Ph H-5), 7.62 (dd, *J* = 8.0, *J* = 2.2, 1H, H-4), 8.21 (s, 1H, H-6), 8.41 (br. s, 1H, NH), 9.90 (br. s, 1H, SO_2_NH). Anal. calcd. for C_12_H_14_N_4_O_2_S %: C 51.78; H 5.07; N 20.13; S 11.52. Found, %: C 51.96; H 5.05; N 20.19; S 11.47.

*2-Hydrazinyl-N-(4-methoxyphenyl)pyridine-5-sulfonamide* (**4d**). According to General Procedure B, 2-chloro-*N*-(4-methoxyphenyl)pyridine-5-sulfonamide **3d** (14.9 g, 50 mmol) yielded 2-hydrazinyl-*N*-(4-methoxyphenyl)pyridine-5-sulfonamide **4d** (13.1 g, 89%), as a white solid, m.p. 198–200 °C. 1H-NMR spectrum δ, ppm *(J*, Hz): 3.67 (s, 3H, OCH_3_), 4.35 (br. s, 2H, NH_2_), 6.67 (d, *J* = 8.0, 1H, H-3), 6.79 (d, *J* = 8.0, 2H, Ph H-3,5), 6.97 (d, *J* = 8.0, 2H, Ph H-2,6), 7.57 (dd, *J* = 8.0, *J* = 2.2, 1H, H-4), 8.09 (d, *J* = 2.2, 1H, H-6), 8.36 (br. s, 1H, NH), 9.54 (br. s, 1H, SO_2_NH). Anal. calcd. for C_12_H_14_N_4_O_3_S %: C 48.97; H 4.79; N 19.04; S 10.89. Found, %: C 49.14; H 4.80; N 18.98; S 10.93.

*N-(4-fluorophenyl)-2-hydrazinylpyridine-5-sulfonamide* (**4e**). According to General Procedure B, 2-chloro-*N*-(4-fluorophenyl)pyridine-5-sulfonamide **3e** (14.3 g, 50 mmol) yielded *N*-(4-fluorophenyl)-2-hydrazinylpyridine-5-sulfonamide **4e** (12.8 g, 91%), as a white solid, m.p. 219–221 °C. 1H-NMR spectrum δ, ppm *(J*, Hz): 4.35 (br. s, 2H, NH_2_), 6.67 (d, *J* = 8.0, 1H, H-3), 7.02–7.12 (m, 4H, Ar H), 7.57 (dd, *J* = 8.0, *J* = 2.2, 1H, H-4), 8.17 (d, *J* = 2.2, 1H, H-6), 8.43 (br. s, 1H, NH), 9.86 (br. s, 1H, SO_2_NH). Anal. calcd. for C_11_H_11_FN_4_O_2_S %: C 46.80; H 3.93; N 19.85; S 11.36. Found, %: C 46.62; H 3.92; N 19.91; S 11.32.

*N-(3-chlorophenyl)-2-hydrazinylpyridine-5-sulfonamide* (**4f**). According to General Procedure B, 2-chloro-*N*-(3-chlorophenyl)pyridine-5-sulfonamide **3f** (15.2 g, 50 mmol) yielded *N*-(3-chlorophenyl)-2-hydrazinylpyridine-5-sulfonamide **4f** (13.3 g, 89%), as a white solid, m.p. 212–214 °C. 1H-NMR spectrum δ, ppm (*J*, Hz): 4.38 (br. s, 2H, NH_2_), 6.68 (d, *J* = 8.0, 1H, H-3), 7.00–7.10 (m, 3H, Ar H), 7.28 (t, *J* = 7.6, 1H, Ph H-5), 7.64 (dd, *J* = 8.0, *J* = 2.2, 1H, H-4), 8.26 (d, *J* = 2.2, 1H, H-6), 8.49 (s, 1H, NH), 10.20 (br. s, 1H, SO_2_NH). Anal. calcd. for C_11_H_11_ClN_4_O_2_S %: C 44.22; H 3.71; N 18.75; S 10.73. Found, %: C 44.07; H 3.72; N 18.78; S 10.76.

*N-(4-chlorophenyl)-2-hydrazinylpyridine-5-sulfonamide* (**4g**). According to General Procedure B, 2-chloro-*N*-(4-chlorophenyl)pyridine-5-sulfonamide **3g** (15.2 g, 50 mmol) yielded *N*-(4-chlorophenyl)-2-hydrazinylpyridine-5-sulfonamide **4g** (13.6 g, 91%), as a white solid, m.p. 224–226 °C. 1H-NMR spectrum δ, ppm *(J*, Hz): 4.38 (br. s, 2H, NH_2_), 6.68 (d, *J* = 8.0, 1H, H-3), 7.09 (d, *J* = 8.0, 2H, Ph H-3,5), 7.28 (d, *J* = 8.0, 2H, Ph H-2,6), 7.61 (dd, *J* = 8.0, *J* = 2.2, 1H, H-4), 8.21 (d, *J* = 2.2, 1H, H-6), 8.50 (s, 1H, NH), 10.12 (br. s, 1H, SO_2_NH). Anal. calcd. for C_11_H_11_ClN_4_O_2_S %: C 44.22; H 3.71; N 18.75; S 10.73. Found, %: C 44.34; H 3.69; N 18.70; S 10.69.

#### 3.3.3. Synthesis of *N*-(aryl)-[1,2,4]Triazolo[4,3-a]Pyridinesulfonamides **6a**–**g**. General Procedure C

A corresponding methyl *ortho*-ester **5a**–**c** (25 mmol) was added to a stirred solution of corresponding N-(aryl)-2-hydrazinylpyridinesulfonamide 4a–g (20 mmol) in anhydrous DMF (20 mL). The reaction mixture was refluxed for 16 h. After cooling, the reaction mixture was diluted with water (100 mL). The precipitate that formed was filtered and recrystallized from a mixture of DMF (10 mL) and *i*-propanol (20 mL). Yields of *N*-(aryl)-[1,2,4]triazolo[4,3-*a*]pyridinesulfonamides **6a**–**g** were 68–82%.

*N-(3,5-difluorophenyl)-[1,2,4]triazolo[4,3-a]pyridine-8-sulfonamide* (**6a**). According to General Procedure C, *N*-(3,5-difluorophenyl)-2-hydrazinylpyridine-3-sulfonamide **4a** (6.0 g, 20 mmol) and methyl *ortho*-formate **5a** (2.74 mL, 25 mmol) yielded *N*-(3,5-difluorophenyl)-[1,2,4]triazolo[4,3-*a*]pyridine-8-sulfonamide **6a** (5.09 g, 82%), as a cream solid, m.p. 184–186 °C. ^1^H-NMR spectrum δ, ppm (*J*, Hz): 6.70–6.88 (m, 3H, Ar H), 7.11 (t, *J* = 7.6, 1H, H-6), 8.10 (d, *J* = 7.6, 1H, H-7), 8.79 (d, *J* = 7.6, 1H, H-5), 9.40 (s, 1H, H-3), 11.44 (br. s, 1H, SO_2_NH). Anal. calcd. for C_12_H_8_F_2_N_4_O_2_S %: C 46.45; H 2.60; N 18.06; S 10.33. Found, %: C 46.29; H 2.61; N 17.99; S 10.38.

*N-(3,5-dimethylphenyl)-3-methyl-[1,2,4]triazolo[4,3-a]pyridine-8-sulfonamide***(6b)**. According to General Procedure C, *N*-(3,5-dimethylphenyl)-2-hydrazinylpyridine-3-sulfonamide **4b** (5.85 g, 20 mmol) and methyl *ortho*-acetate **5b** (3.18 mL, 25 mmol) yielded *N*-(3,5-dimethylphenyl)-3-methyl-[1,2,4]triazolo[4,3-*a*]pyridine-8-sulfonamide **6b** (4.81 g, 76%), as a cream solid, m.p. 176–178 °C. ^1^H-NMR spectrum δ, ppm (*J*, Hz): 2.06 (s, 6H, 2 CH_3_), 2.68 (s, 3H, 3-CH_3_), 6.57 (s, 1H, Ph H-4), 6.70 (s, 2H, Ph H-2,6), 7.04 (t, *J* = 7.6, 1H, H-6), 7.91 (d, *J* = 7.6, 1H, H-7), 8.53 (d, *J* = 7.6, 1H, H-5), 10.00 (br. s, 1H, SO_2_NH). Anal. calcd. for C_15_H_16_N_4_O_2_S %: C 56.95; H 5.10; N 17.71; S 10.13. Found, %: C 57.12; H 5.08; N 17.66; S 10.09.

*3-Methyl-N-(3-methylphenyl)-[1,2,4]triazolo[4,3-a]pyridine-6-sulfonamide* (**6c**). According to General Procedure C, *N*-(3-methylphenyl)-2-hydrazinylpyridine-5-sulfonamide **4c** (5.57 g, 20 mmol) and methyl *ortho*-acetate **5b** (3.18 mL, 25 mmol) yielded 3-methyl-*N*-(3-methylphenyl)-[1,2,4]triazolo[4,3-*a*]pyridine-6-sulfonamide **6c** (4.23 g, 70%), as a cream solid, m.p. 164–166 °C. ^1^H-NMR spectrum δ, ppm (*J*, Hz): 2.20 (s, 3H, Ph CH_3_), 2.71 (s, 3H, 3-CH_3_), 6.88 (d, *J* = 7.6, 1H, Ar H), 6.91–6.98 (m, 2H, Ar H), 7.11 (t, *J* = 7.6, 1H, Ph H-5), 7.46 (d, *J* = 7.6, 1H, H-8), 7.85 (d, *J* = 7.6, 1H, H-7), 8.76 (s, 1H, H-5), 10.45 (br. s, 1H, SO_2_NH). Anal. calcd. for C_14_H_14_N_4_O_2_S %: C 55.62; H 4.67; N 18.53; S 10.60. Found, %: C 55.48; H 4.68; N 18.60; S 10.57.

*3-Ethyl-N-(4-methoxyphenyl)-[1,2,4]triazolo[4,3-a]pyridine-6-sulfonamide* (**6d**). According to General Procedure C, 2-hydrazinyl-*N*-(4-methoxyphenyl)pyridine-5-sulfonamide **4d** (5.88 g, 20 mmol) and methyl *ortho*-propionate **5c** (3.55 mL, 25 mmol) yielded 3-ethyl-*N*-(4-methoxyphenyl)-[1,2,4]triazolo[4,3-*a*]pyridine-6-sulfonamide **6d** (4.52 g, 68%), as a cream solid, m.p. 161–163 °C. ^1^H-NMR spectrum δ, ppm (*J*, Hz): 1.28 (t, *J* = 7.0, 3H, CH_2_CH_3_), 3.09 (q, *J* = 7.0, 2H, CH_2_CH_3_), 3.66 (s, 3H, OCH_3_), 6.80 (d, *J* = 7.6, 2H, Ph H-3,5), 7.00 (d, *J* = 7.6, 2H, Ph H-2,6), 7.44 (d, *J* = 7.6, 1H, H-8), 7.88 (d, *J* = 7.6, 1H, H-7), 8.59 (s, 1H, H-5), 10.22 (s, 1H, SO_2_NH). Anal. calcd. for C_15_H_16_N_4_O_3_S %: C 54.20; H 4.85; N 16.86; S 9.65. Found, %: C 54.03; H 4.87; N 16.90; S 9.67.

*N-(4-fluorophenyl)-3-methyl-[1,2,4]triazolo[4,3-a]pyridine-6-sulfonamide* (**6e**). According to General Procedure C, *N*-(4-fluorophenyl)-2-hydrazinylpyridine-5-sulfonamide **4e** (5.97 g, 20 mmol) and methyl *ortho*-acetate **5b** (3.18 mL, 25 mmol) yielded *N*-(4-fluorophenyl)-3-methyl-[1,2,4]triazolo[4,3-*a*]pyridine-6-sulfonamide **6e** (4.90 g, 80%), as a cream solid, m.p. 176–177°C. ^1^H-NMR spectrum δ, ppm (*J*, Hz): 2.69 (s, 3H, 3-CH_3_), 7.00–7.18 (m, 4H, Ar H), 7.40 (d, *J* = 7.6, 1H, H-8), 7.87 (d, *J* = 7.6, 1H, H-7), 8.68 (s, 1H, H-5), 10.50 (br. s, 1H, SO_2_NH). Anal. calcd. for C_13_H_11_FN_4_O_2_S %: C 50.97; H 3.62; N 18.29; S 10.47. Found, %: C 51.14; H 3.62; N 18.35; S 10.50.

*N-(3-chlorophenyl)-3-methyl-[1,2,4]triazolo[4,3-a]pyridine-6-sulfonamide* (**6f**). According to General Procedure C, *N*-(3-chlorophenyl)-2-hydrazinylpyridine-5-sulfonamide **4f** (5.97 g, 20 mmol) and methyl *ortho*-acetate **5b** (3.18 mL, 25 mmol) yielded *N*-(3-chlorophenyl)-3-methyl-[1,2,4]triazolo[4,3-*a*]pyridine-6-sulfonamide **6f** (4.97 g, 77%), as a cream solid, m.p. 179–181 °C. ^1^H-NMR spectrum δ, ppm (*J*, Hz): 2.70 (s, 3H, 3-CH_3_), 7.01–7.20 (m, 3H, Ar H), 7.24 (t, *J* = 7.6, 1H, Ph H-5), 7.44 (d, *J* = 7.6, 1H, H-8), 7.88 (d, *J* = 7.6, 1H, H-7), 8.83 (s, 1H, H-5), 10.90 (br. s, 1H, SO_2_NH). Anal. calcd. for C_13_H_11_ClN_4_O_2_S %: C 48.38; H 3.44; N 17.36; S 9.93. Found, %: C 48.22; H 3.42; N 17.41; S 9.90.

*N-(4-chlorophenyl)-3-methyl-[1,2,4]triazolo[4,3-a]pyridine-6-sulfonamide* (**6g**). According to General Procedure C, *N*-(4-chlorophenyl)-2-hydrazinylpyridine-5-sulfonamide **4g** (5.97 g, 20 mmol) and methyl *ortho*-acetate **5b** (3.18 mL, 25 mmol) yielded *N*-(4-chlorophenyl)-3-methyl-[1,2,4]triazolo[4,3-*a*]pyridine-6-sulfonamide **6g** (5.23 g, 81%), as a cream solid, m.p. 206–208 °C. ^1^H-NMR spectrum δ, ppm (*J*, Hz): 2.70 (s, 3H, 3-CH_3_), 7.15 (d, *J* = 8.0, 2H, Ph H-3,5), 7.28 (d, *J* = 8.0, 2H, Ph H-2,6), 7.42 (d, *J* = 7.6, 1H, H-8), 7.87 (d, *J* = 7.6, 1H, H-7), 8.78 (s, 1H, H-5), 10.75 (br. s, 1H, SO_2_NH). Anal. calcd. for C_13_H_11_ClN_4_O_2_S %: C 48.38; H 3.44; N 17.36; S 9.93. Found, %: C 48.47; H 3.46; N 17.30; S 9.97.

#### 3.3.4. Synthesis of *N,N*-Disubstituted [1,2,4]Triazolo[4,3-a]Pyridinesulfonamides **8a**–**i**. General Procedure D

A powder of dry K_2_CO_3_ (0.42 g, 3 mmol) was added to a stirred solution of corresponding N-(aryl)-[1,2,4]triazolo[4,3-a]pyridinesulfonamide 6a-g (1 mmol) in anhydrous DMF (5 mL). Then, appropriate benzyl chloride **7a**–**h** (1.1 mmol) was added and the reaction mixture was heated at 90 °C for 2 h. After cooling, the reaction mixture was diluted with water (25 mL). The precipitate that formed was filtered off, washed with water (5 mL) and recrystallized from a mixture of DMF (5 mL) and *i*-propanol (20 mL). Yields of *N,N*-disubstituted [1,2,4]triazolo[4,3-a]pyridinesulfonamides **8a–i** were 60–72%.

*N-(3-chlorobenzyl)-N-(3,5-difluorophenyl)-[1,2,4]triazolo[4,3-a]pyridine-8-sulfonamide* (**8a**). According to General Procedure D, *N*-(3,5-difluorophenyl)-[1,2,4]triazolo[4,3-*a*]pyridine-8-sulfonamide **6a** (0.31 g, 1 mmol) and 3-chlorobenzyl chloride **7a** (0.18 g, 1.1 mmol) yielded *N*-(3-chlorobenzyl)-*N*-(3,5-difluorophenyl)-[1,2,4]triazolo[4,3-*a*]pyridine-8-sulfonamide **8a** (0.27 g, 62%), as a white solid, m.p. 160–162 °C, ^1^H-NMR spectrum, δ, ppm (*J*, Hz): 5.25 (s, 2H, CH_2_), 7.00–7.18 (m, 4H, Ar H), 7.22–7.45 (m, 4H, Ar H), 7.87 (d, *J* = 7.6, 1H, H-7), 8.88 (d, *J* = 7.6, 1H, H-5), 9.51 (s, 1H, H-3). ^13^C-NMR spectrum, δ, ppm: 53.8 (CH_2_), 103.5 (t, *J_C__-F_* = 25.7 Hz, Ph C-4), 111.5 (dd, *J_C__-F_* = 18.1 Hz, 9.1 Hz, 2 C, Ph C-2,6), 112.5, 124.4, 126.6, 127.6, 127.7, 130.4, 130.9, 132.9, 133.1, 137.9, 138.8, 140.7 (t, *J_C__-F_* = 12.8 Hz, Ph C-1), 143.6, 161.9 (dd, *J_C__-F_* = 246.8 Hz, 15.1 Hz, 2 C, Ph C-3,5). LC/MS *m*/*z* (%): 435.6 [M + H]^+^ (100.0). Anal. calcd. for C_19_H_13_ClF_2_N_4_O_2_S %: C 52.48, H 3.01, N 12.88, S 7.37. Found, %: C 52.65, H 2.99, N 12.86, S 7.40.

*N-(2,5-dimethylbenzyl)-N-(3,5-dimethylphenyl)-3-methyl-[1,2,4]triazolo[4,3-a]pyridine-8-sulfonamide* (**8b**). According to General Procedure D, *N*-(3,5-dimethylphenyl)-3-methyl-[1,2,4]triazolo[4,3-*a*]pyridine-8-sulfonamide **6b** (0.31 g, 1 mmol) and 2,5-dimethylbenzyl chloride **7b** (0.17 g, 1.1 mmol) yielded *N*-(2,5-dimethylbenzyl)-*N*-(3,5-dimethylphenyl)-3-methyl-[1,2,4]triazolo[4,3-*a*]pyridine-8-sulfonamide **8b** (0.26 g, 60%), as a white solid, m.p. 195–196 °C, ^1^H-NMR spectrum, δ, ppm (*J*, Hz): 2.06 (s, 6H, 2 CH_3_), 2.15 (s, 3H, CH_3_), 2.18 (s, 3H, CH_3_), 2.78 (s, 3H, 3-CH_3_), 5.20 (s, 2H, CH_2_), 6.66 (s, 2H, Ph H-2,6), 6.80 (s, 1H, Ar H), 6.88–7.01 (m, 3H, Ar H), 7.06 (s, 1H, Ar H), 7.55 (d, *J* = 7.6, 1H, H-7), 8.61 (d, *J* = 7.6, 1H, H-5). ^13^C-NMR spectrum, δ, ppm: 10.0 (3-CH_3_), 18.3 (CH_3_), 20.6 (3 C, CH_3_), 53.9 (CH_2_), 111.6, 125.4, 126.0 (2 C), 128.0, 129.0, 129.4, 129.6, 130.0, 131.2, 132.9, 134.5, 134.6, 138.0 (2 C), 138.4, 144.6, 145.2. LC/MS *m*/*z* (%): 435.4 [M + H]^+^ (100.0). Anal. calcd. for C_24_H_26_N_4_O_2_S %: C 66.33, H 6.03, N 12.89, S 7.38. Found, %: C 66.15, H 6.05, N 12.95, S 7.41.

*N-(3,5-dimethylphenyl)-N-(4-methoxybenzyl)-3-methyl-[1,2,4]triazolo[4,3-a]pyridine-8-sulfonamide* (**8c**). According to General Procedure D, *N*-(3,5-dimethylphenyl)-3-methyl-[1,2,4]triazolo[4,3-*a*]pyridine-8-sulfonamide **6b** (0.31 g, 1 mmol) and 4-methoxybenzyl chloride **7c** (0.17 g, 1.1 mmol) yielded *N*-(2,5-dimethylbenzyl)-*N*-(4-methoxyphenyl)-3-methyl-[1,2,4]triazolo[4,3-*a*]pyridine-8-sulfonamide **8b** (0.27 g, 62%), as a white solid, m.p. 168–169 °C, ^1^H-NMR spectrum, δ, ppm (*J*, Hz): 2.04 (s, 6H, 2CH_3_), 2.76 (s, 3H, 3-CH_3_), 3.71 (s, 3H, OCH_3_), 5.16 (s, 2H, CH_2_), 6.60 (s, 2H, Ph H-2,6), 6.78–6.85 (m, 3H, Ph H-4 + Bn H-3,5), 7.00 (t, *J* = 7.6, 1H, H-6), 7.19 (d, *J* = 8.0, 2H, Bn H-2,6), 7.60 (d, *J* = 7.6, 1H, H-7), 8.62 (d, *J* = 7.6, 1H, H-5). ^13^C-NMR spectrum, δ, ppm: 10.0 (3-CH_3_), 20.7 (2C, Ph 3,5-CH_3_), 55.0 (CH_2_), 55.6 (OCH_3_), 111.6, 113.8 (2C), 125.7, 126.1 (2C), 129.0, 129.1, 129.3 (2C), 129.4, 131.0, 138.1 (2C), 138.3, 144.4, 145.0, 158.6 (Bn C-4). LC/MS *m*/*z* (%): 437.4 [M + H]^+^ (100.0). Anal. calcd. for C_23_H_24_N_4_O_3_S %: C 63.28, H 5.54, N 12.83, S 7.35. Found, %: C 63.44, H 5.55, N 12.89, S 7.32.

*Methyl 5-{[3-methyl-N-(3-methylphenyl)-[1,2,4]triazolo[4,3-a]pyridine-6-sulfonamido]methyl}furan-2-carboxylate* (**8d**). According to General Procedure D, 3-methyl-*N*-(3-methylphenyl)-[1,2,4]triazolo[4,3-*a*]pyridine-6-sulfonamide **6c** (0.30 g, 1 mmol) and methyl 5-(chloromethyl)furan-2-carboxylate **7d** (0.19 g, 1.1 mmol) yielded methyl 5-{[3-methyl-*N*-(3-methylphenyl)-[1,2,4]triazolo[4,3-*a*]pyridine-6-sulfonamido]methyl}furan-2-carboxylate **8d** (0.28 g, 60%), as a white solid, m.p. 152–154 °C, ^1^H-NMR spectrum, δ, ppm (*J*, Hz): 2.24 (s, 3H, Ph CH_3_), 2.77 (s, 3H, 3-CH_3_), 3.74 (s, 3H, OCH_3_), 4.98 (s, 2H, CH_2_), 6.42 (d, *J* = 3.6, 1H, furan H-4), 7.00 (d, *J* = 7.6, 1H, Ar H), 7.06–7.18 (m, 4H, Ar H), 7.23 (t, *J* = 7.6, 1H, Ar H), 7.81 (d, *J* = 7.6, 1H, H-7), 8.81 (s, 1H, H-5). ^13^C-NMR spectrum, δ, ppm: 9.9 (3-CH_3_), 20.7 (CH_3_), 47.6 (CH_2_), 51.8 (OCH_3_), 111.8, 115.8, 119.0, 123.8, 125.1, 125.6, 127.3, 129.0, 129.2, 129.4, 138.2, 138.7, 143.4, 145.8, 148.4, 154.0, 158.0. LC/MS *m*/*z* (%): 441.4 [M + H]^+^ (100.0). Anal. calcd. for C_21_H_20_N_4_O_5_S %: C 57.26, H 4.58, N 12.72, S 7.28. Found, %: C 57.08, H 4.56, N 12.69, S 7.31.

*3-Ethyl-N-(3-fluorobenzyl)-N-(4-methoxyphenyl)-[1,2,4]triazolo[4,3-a]pyridine-6-sulfonamide* (**8e**). According to General Procedure D, 3-ethyl-*N*-(4-methoxyphenyl)-[1,2,4]triazolo[4,3-*a*]pyridine-6-sulfonamide **6d** (0.33 g, 1 mmol) and 3-fluorobenzyl chloride **7e** (0.16 g, 1.1 mmol) yielded 3-ethyl-*N*-(3-fluorobenzyl)-*N*-(4-methoxyphenyl)-[1,2,4]triazolo[4,3-*a*]pyridine-6-sulfonamide **8e** (0.28 g, 64%), as a brown solid, m.p. 158–159 °C, ^1^H-NMR spectrum, δ, ppm (*J*, Hz): 1.34 (t, *J* = 7.0, 3H, CH_2_CH_3_), 3.18 (q, *J* = 7.0, 2H, CH_2_CH_3_), 3.70 (s, 3H, OCH_3_), 4.83 (s, 2H, CH_2_), 6.83 (d, *J* = 7.6, 2H, Ph H-3,5), 6.98–7.30 (m, 7H, Ar H), 7.85 (d, *J* = 7.6, 1H, H-7), 8.78 (s, 1H, H-5). ^13^C NMR spectrum, δ, ppm: 10.8 (3-Et-CH_3_), 17.3 (3-Et-CH_2_), 53.5 (N-CH_2_), 55.3 (OCH_3_), 114.3 (2C), 114.6, 114.7 (d, *J_C-F_* = 27.2 Hz), 116.2, 124.1 (d, *J_C-F_* = 17.4 Hz), 124.2, 125.0, 126.9, 130.1 (2C), 130.4 (d, *J_C-F_* = 8.3 Hz), 130.6, 139.2 (d, *J_C-F_* = 7.5 Hz), 148.5, 149.9, 158.8 (Ph C-4), 162.1 (d, *J_C-F_* = 243.8 Hz, Bn C-3). LC/MS *m*/*z* (%): 441.2 [M + H]^+^ (100.0). Anal. calcd. for C_22_H_21_FN_4_O_3_S %: C 59.99, H 4.81, N 12.72, S 7.28. Found, %: C 59.82, H 4.84, N 12.65, S 7.33.

*N-(4-fluorophenyl)-3-methyl-N-(3-methylbenzyl)-[1,2,4]triazolo[4,3-a]pyridine-6-sulfonamide* (**8f**). According to General Procedure D, *N*-(4-fluorophenyl)-3-methyl-[1,2,4]triazolo[4,3-*a*]pyridine-6-sulfonamide **6e** (0.31 g, 1 mmol) and 3-methylbenzyl chloride **7f** (0.16 g, 1.1 mmol) yielded *N*-(4-fluorophenyl)-3-methyl-*N*-(3-methylbenzyl)-[1,2,4]triazolo[4,3-*a*]pyridine-6-sulfonamide **8f** (0.25 g, 61%), as a brown solid, m.p. 104–105 °C, ^1^H-NMR spectrum, δ, ppm (*J*, Hz): 2.16 (s, 3H, Bn CH_3_), 2.77 (s, 3H, 3-CH_3_), 4.78 (s, 2H, CH_2_), 6.93–7.28 (m, 9H, Ar H), 7.82 (d, *J* = 7.6, 1H, H-7), 8.82 (s, 1H, H-5). ^13^C NMR spectrum, δ, ppm: 10.3 (3-CH_3_), 21.3 (Bn CH_3_), 54.5 (CH_2_), 116.3 (d, *J_C-F_* = 23.7 Hz, 2C, Ph C-3,5), 116.5, 124.1, 125.6, 125.7, 127.5, 127.6, 128.6, 128.7, 129.2, 131.5 (d, *J_C-F_* = 9.2 Hz, 2C, Ph C-2,6), 135.0, 136.1, 138.0, 146.2, 161.7 (d, *J_C-F_* = 243.4 Hz, Ph C-4). LC/MS *m*/*z* (%): 411.0 [M + H]^+^ (100.0). Anal. calcd. for C_21_H_19_FN_4_O_2_S %: C 61.45, H 4.67, N 13.65, S 7.81. Found, %: C 61.66, H 4.70, N 13.58, S 7.83.

*N-(3-chlorobenzyl)-N-(4-fluorophenyl)-3-methyl-[1,2,4]triazolo[4,3-a]pyridine-6-sulfonamide* (**8g**). According to General Procedure D, *N*-(4-fluorophenyl)-3-methyl-[1,2,4]triazolo[4,3-*a*]pyridine-6-sulfonamide **6e** (0.31 g, 1 mmol) and 3-chlorobenzyl chloride **7a** (0.18 g, 1.1 mmol) yielded *N*-(3-chlorobenzyl)-*N*-(4-fluorophenyl)-3-methyl-[1,2,4]triazolo[4,3-*a*]pyridine-6-sulfonamide **8g** (0.27 g, 63%), as a pink solid, m.p. 107–109 °C, ^1^H-NMR spectrum, δ, ppm (*J*, Hz): 2.77 (s, 3H, 3-CH_3_), 4.84 (s, 2H, CH_2_), 7.08–7.35 (m, 9H, Ar H), 7.83 (d, *J* = 7.6, 1H, H-7), 8.84 (s, 1H, H-5). ^13^C NMR spectrum, δ, ppm:10.3 (3-CH_3_), 53.8 (CH_2_), 116.4 (d, *J_C-F_* = 22.9 Hz, 2C, Ph C-3,5), 116.5, 124.1, 125.3, 127.3, 127.8, 128.0, 128.4, 130.8, 131.4 (d, *J_C-F_* = 9.2 Hz, 2C, Ph C-2,6), 133.5, 134.8, 138.9, 146.1, 148.9, 161.7 (d, *J_C-F_* = 247.2 Hz, Ph C-4). LC/MS *m*/*z* (%): 431.0 [M + H]^+^ (100.0). Anal. calcd. for C_20_H_16_ClFN_4_O_2_S %: C 55.75, H 3.74, N 13.00, S 7.44. Found, %: C 55.59, H 3.75, N 12.94, S 7.42.

*N-(3-chlorophenyl)-N-(2-fluorobenzyl)-3-methyl-[1,2,4]triazolo[4,3-a]pyridine-6-sulfonamide* (**8h**). According to General Procedure D, *N*-(3-chlorophenyl)-3-methyl-[1,2,4]triazolo[4,3-*a*]pyridine-6-sulfonamide **6f** (0.32 g, 1 mmol) and 2-fluorobenzyl chloride **7g** (0.16 g, 1.1 mmol) yielded *N*-(3-chlorophenyl)-*N*-(4-fluorobenzyl)-3-methyl-[1,2,4]triazolo[4,3-*a*]pyridine-6-sulfonamide **8h** (0.29 g, 67%), as a pink solid, m.p. 153–155 °C, ^1^H-NMR spectrum, δ, ppm (*J*, Hz): 2.77 (s, 3H, 3-CH_3_), 4.94 (s, 2H, CH_2_), 7.03–7.38 (m, 9H, Ar H), 7.82 (d, *J* = 7.6, 1H, H-7), 8.88 (s, 1H, H-5). ^13^C NMR spectrum, δ, ppm:10.3 (3-CH_3_), 48.5 (CH_2_), 115.7 (d, *J_C-F_* = 21.4 Hz), 116.4, 122.8 (d, *J_C-F_* = 13.8 Hz), 124.1, 124.8, 125.3, 127.9, 128.0, 128.8, 129.2, 130.5 (d, *J_C-F_* = 8.4 Hz), 130.9, 131.5, 133.5, 140.1, 146.3, 148.9, 160.8 (d, *J_C-F_* = 248.7 Hz, Bn C-2). LC/MS *m*/*z* (%): 431.4 [M + H]^+^ (100.0). Anal. calcd. for C_20_H_16_ClFN_4_O_2_S %: C 55.75, H 3.74, N 13.00, S 7.44. Found, %: C 55.62, H 3.73, N 12.96, S 7.48.

*N-(4-chlorophenyl)-N-(4-fluorobenzyl)-3-methyl-[1,2,4]triazolo[4,3-a]pyridine-6-sulfonamide* (**8i**). According to General Procedure D, *N*-(4-chlorophenyl)-3-methyl-[1,2,4]triazolo[4,3-*a*]pyridine-6-sulfonamide **6g** (0.32 g, 1 mmol) and 4-fluorobenzyl chloride **7h** (0.16 g, 1.1 mmol) yielded *N*-(4-chlorophenyl)-*N*-(4-fluorobenzyl)-3-methyl-[1,2,4]triazolo[4,3-*a*]pyridine-6-sulfonamide **8h** (0.31 g, 72%), as a pink solid, m.p. 198–199 °C, ^1^H-NMR spectrum, δ, ppm (*J*, Hz): 2.77 (s, 3H, 3-CH_3_), 4.81 (s, 2H, CH_2_), 7.00–7.38 (m, 9H, Ar H), 7.82 (d, *J* = 7.6, 1H, H-7), 8.88 (s, 1H, H-5). ^13^C NMR spectrum, δ, ppm:10.3 (3-CH_3_), 53.5 (CH_2_), 115.7 (d, *J_C-F_* = 21.4 Hz, 2C, Bn C-3,5), 116.6, 124.0, 125.4, 127.7, 129.5 (2C), 130.7 (d, *J_C-F_* = 8.4 Hz, 2C, Bn C-2,6), 131.0 (2C), 132.2, 133.1, 137.5, 146.3, 148.8, 162.0 (d, *J_C-F_* = 251.0 Hz, Bn C-4). LC/MS *m*/*z* (%): 431.0 [M + H]^+^ (100.0). Anal. calcd. for C_20_H_16_ClFN_4_O_2_S %: C 55.75, H 3.74, N 13.00, S 7.44. Found, %: C 55.89, H 3.76, N 12.95, S 7.40.

#### 3.3.5. Synthesis of 2-Chloro-*N*-Substitutedpyridinesulfonamides **10a**–**g**. General Procedure E

Secondary amine 9a–f (0.22 mol) was added, for 5 min at room temperature, to a stirred solution of sulfonyl chloride **1a,b** (21.2 g, 0.1 mol) in dry dioxane (100 mL). The reaction mixture was stirred at 60 °C for 1 h. After cooling, the reaction mixture was diluted with water (500 mL). The precipitate that formed was filtered and recrystallized from *i*-propanol. Yields of 2-chloro-*N*-substitutedpyridinesulfonamides **10a**–**g** were 67–92%.

*2-Chloro-3-(piperidin-1-ylsulfonyl)pyridine* (**10a**). According to General Procedure E, 2-chloropyridine-3-sulfonyl chloride **1a** (21.2 g, 0.1 mol) and piperidine **9a** (21.7 mL, 0.22 mol) yielded 2-chloro-3-(piperidin-1-ylsulfonyl)pyridine **10a** (22.9 g, 88%), as a white solid, m.p. 116–118 °C. ^1^H-NMR spectrum δ, ppm (*J*, Hz): 1.45–1.55 (m, 6H, 3 CH_2_), 3.16–3.24 (m, 4H, 2 NCH_2_), 7.65 (dd, *J* = 8.0, *J* = 7.2, 1H, H-5), 8.35 (dd, *J* = 8.0, *J* = 1.5, 1H, H-6), 8.65 (dd, *J* = 7.2, *J* = 1.5, 1H, H-4). Anal. calcd. for C_10_H_13_ClN_2_O_2_S %: C 46.06; H 5.03; N 10.74; S 12.30. Found, %: C 45.92; H 5.01; N 10.77; S 12.25.

*4-(2-Chloropyridin-3-ylsulfonyl)morpholine* (**10b**). According to General Procedure E, 2-chloropyridine-3-sulfonyl chloride **1a** (21.2 g, 0.1 mol) and morpholine **9b** (19.0 mL, 0.22 mol) yielded 4-(2-chloropyridin-3-ylsulfonyl)morpholine **10b** (24.2 g, 92%), as a white solid, m.p. 127–128 °C (128–129 °C [38]**).** ^1^H-NMR spectrum δ, ppm (*J*, Hz): 3.17–3.24 (m, 4H, 2 NCH_2_), 3.55–3.62 (m, 4H, 2 OCH_2_), 7.66 (dd, *J* = 8.0, *J* = 7.2, 1H, H-5), 8.37 (dd, *J* = 8.0, *J* = 1.5, 1H, H-6), 8.67 (dd, *J* = 7.2, *J* = 1.5, 1H, H-4). Anal. calcd. for C_9_H_11_ClN_2_O_3_S %: C 41.15; H 4.22; N 10.66; S 12.20. Found, %: C 41.02; H 4.21; N 10.70; S 12.24.

*2-Chloro-5-(piperidin-1-ylsulfonyl)pyridine* (**10c**). According to General Procedure E, 2-chloropyridine-5-sulfonyl chloride **1b** (21.2 g, 0.1 mol) and piperidine **9a** (21.7 mL, 0.22 mol) yielded 2-chloro-5-(piperidin-1-ylsulfonyl)pyridine **10c** (24.0 g, 92%), as a white solid, m.p. 114–116 °C. ^1^H-NMR spectrum δ, ppm (*J*, Hz): 1.27–1.58 (m, 6H, 3 CH_2_), 2.88–2.98 (m, 4H, 2 NCH_2_), 7.78 (d, *J* = 8.0, 1H, H-3), 8.16 (dd, *J* = 8.0, *J* = 2.2, 1H, H-4), 8.73 (d, *J* = 2.2, 1H, H-6). Anal. calcd. for C_10_H_13_ClN_2_O_2_S %: C 46.06; H 5.03; N 10.74; S 12.30. Found, %: C 45.95; H 5.04; N 10.71; S 12.32.

*2-Chloro-5-(4-methylpiperidin-1-ylsulfonyl)pyridine* (**10d**). According to General Procedure E, 2-chloropyridine-5-sulfonyl chloride **1b** (21.2 g, 0.1 mol) and 4-methylpiperidine **9c** (25.4 mL, 0.22 mol) yielded 2-chloro-5-(4-methylpiperidin-1-ylsulfonyl)pyridine **10d** (23.9 g, 87%), as a white solid, m.p. 105–107 °C. ^1^H-NMR spectrum δ, ppm (*J*, Hz): 0.86 (d, *J* = 7.0, 3H, CH_3_), 0.98–1.40 (m, 3H), 1.54–1.68 (m, 2H), 2.22–2.40 (m, 2H, NCH_2_), 3.52–3.66 (m, 2H, NCH_2_), 7.77 (d, *J* = 8.0, 1H, H-3), 8.17 (dd, *J* = 8.0, *J* = 2.2, 1H, H-4), 8.72 (d, *J* = 2.2, 1H, H-6). Anal. calcd. for C_11_H_15_ClN_2_O_2_S %: C 48.08; H 5.50; N 10.20; S 11.67. Found, %: C 47.93; H 5.48; N 10.17; S 11.71.

*4-(6-Chloropyridin-3-ylsulfonyl)thiomorpholine* (**10e**). According to General Procedure E, 2-chloropyridine-5-sulfonyl chloride **1b** (21.2 g, 0.1 mol) and thiomorpholine **9d** (22.1 mL, 0.22 mol) yielded 4-(6-chloropyridin-3-ylsulfonyl)thiomorpholine **10e** (24.8 g, 89%), as a white solid, m.p. 117–119 °C. ^1^H-NMR spectrum δ, ppm (*J*, Hz): 2.60–2.68 (m, 4H, 2 SCH_2_), 3.20–3.32 (m, 4H, 2 NCH_2_), 7.79 (d, *J* = 8.0, 1H, H-3), 8.18 (dd, *J* = 8.0, *J* = 2.2, 1H, H-4), 8.77 (d, *J* = 2.2, 1H, H-6). Anal. calcd. for C_9_H_11_ClN_2_O_2_S_2_ %: C 38.78; H 3.98; N 10.05; S 23.00. Found, %: C 38.92; H 3.96; N 10.01; S 22.95.

*2-Chloro-3-(pyrrolidine-1-ylsulfonyl)pyridine* (**10f**). According to General Procedure E, 2-chloropyridine-3-sulfonyl chloride **1a** (21.2 g, 0.1 mol) and pyrrolidine **9e** (18.1 mL, 0.22 mol) yielded 2-chloro-3-(pyrrolidin-1-ylsulfonyl)pyridine **10f** (21.7 g, 88%), as a white solid, m.p. 101–102 °C. ^1^H-NMR spectrum δ, ppm (*J*, Hz): 1.61–1.70 (m, 4H, 2 CH_2_), 3.16–3.24 (m, 4H, 2 NCH_2_), 7.40 (dd, *J* = 8.0, *J* = 7.2, 1H, H-5), 8.22 (dd, *J* = 8.0, *J* = 1.5, 1H, H-6), 8.52 (dd, *J* = 7.2, *J* = 1.5, 1H, H-4). Anal. calcd. for C_9_H_11_ClN_2_O_2_S %: C 43.82; H 4.49; N 11.35; S 13.00. Found, %: C 43.96; H 4.51; N 11.38; S 12.95.

*1-(2-Chloropyridin-3-ylsulfonyl)-1,2,3,4-tetrahydroquinoline* (**10g**). According to General Procedure E, 2-chloropyridine-3-sulfonyl chloride **1a** (21.2 g, 0.1 mol) and 1,2,3,4-tetrahydroquinoline **9f** (27.7 mL, 0.22 mol) yielded 1-(2-chloropyridin-3-ylsulfonyl)-1,2,3,4-tetrahydroquinoline **10g** (20.7 g, 67%), as a white solid, m.p. 144–146 °C. ^1^H-NMR spectrum δ, ppm (*J*, Hz): 1.82–1.94 (m, 2H, THQ 3-CH_2_), 2.54–2.80 (m, 2H, THQ 4-CH_2_), 3.37–3.47 (m, 2H, THQ 2-CH_2_), 6.56 (t, *J* = 7.6, 1H, Ar H), 6.64 (d, *J* = 7.6, 1H, Ar H), 7.02 (d, *J* = 7.6, 1H, Ar H), 7.13 (t, *J* = 7.6, 1H, Ar H), 7.71 (dd, *J* = 8.0, *J* = 7.2, 1H, H-5), 8.41 (dd, *J* = 8.0, *J* = 1.5, 1H, H-6), 8.68 (dd, *J* = 7.2, *J* = 1.5, 1H, H-4). Anal. calcd. for C_14_H_13_ClN_2_O_2_S %: C 54.46; H 4.24; N 9.07; S 10.38. Found, %: C 54.64; H 4.22; N 9.11; S 10.41.

#### 3.3.6. Synthesis of 2-Hydrazinyl-Pyridinesulfonamides **11a**–**g**. General Procedure F

Corresponding 2-chloro-*N*-substitutedpyridinesulfonamide **10a**–**g** (50 mmol) was added to a solution of hydrazine hydrate (13 mL, 200 mmol) in *i*-propanol (50 mL). The reaction mixture was refluxed for 4 h. After cooling, the reaction mixture was diluted with water (200 mL). The precipitate that formed was filtered and recrystallized from EtOH. The yields of 2-hydrazinyl-pyridinesulfonamides **11a**–**g** were 82–90%.

*2-Hydrazinyl-3-(piperidin-1-ylsulfonyl)pyridine* (**11a**). According to General Procedure F, 2-chloro-3-(piperidin-1-ylsulfonyl)pyridine **10a** (13.0 g, 50 mmol) yielded 2-hydrazinyl-3-(piperidin-1-ylsulfonyl)pyridine **11a** (11.0 g, 86%), as a white solid, m.p. 154–156 °C. ^1^H-NMR spectrum δ, ppm (*J*, Hz): 1.26–1.46 (m, 6H, 3 CH_2_), 3.00–3.15 (m, 4H, 2 NCH_2_), 4.59 (br. s, 2H, NH_2_), 6.72 (t, *J* = 8.0, 1H, H-5), 7.85 (d, *J* = 8.0, 1H, H-6), 8.35 (d, *J* = 7.2, 1H, H-4), 8.80 (br. s, 1H, NH). Anal. calcd. for C_10_H_16_N_4_O_2_S %: C 46.86; H 6.29; N 21.86; S 12.51. Found, %: C 47.02; H 6.31; N 21.79; S 12.46.

*4-(2-Hydrazinylpyridin-3-ylsulfonyl)morpholine* (**11b**)**.** According to General Procedure F, 4-(2-chloropyridin-3-ylsulfonyl)morpholine **10b** (13.1 g, 50 mmol) yielded 4-(2-hydrazinylpyridin-3-ylsulfonyl)morpholine **11b** (11.5 g, 89%), as a white solid, m.p. 160–162 °C (160–161 °C [38]). ^1^H-NMR spectrum δ, ppm (*J*, Hz): 2.93–3.09 (m, 4H, 2 NCH_2_), 3.61–3.67 (m, 4H, 2 OCH_2_), 4.20 (br. s, 2H, NH_2_), 7.20 (dd, *J* = 8.0, *J* = 7.2, 1H, H-5), 8.11 (dd, *J* = 8.0, *J* = 1.5, 1H, H-6), 8.51 (dd, *J* = 7.2, *J* = 1.5, 1H, H-4), 9.00 (br. s, 1H, NH). Anal. calcd. for C_9_H_14_N_4_O_3_S %: C 41.85; H 5.46; N 21.69; S 12.41. Found, %: C 41.73; H 5.48; N 21.74; S 12.44.

*2-Hydrazinyl-5-(piperidin-1-ylsulfonyl)pyridine* (**11c**)**.** According to General Procedure F, 2-chloro-5-(piperidin-1-ylsulfonyl)pyridine **10c** (13.0 g, 50 mmol) yielded 2-hydrazinyl-5-(piperidin-1-ylsulfonyl)pyridine **11c** (11.5 g, 90%), as a white solid, m.p. 158–160 °C. ^1^H-NMR spectrum δ, ppm (*J*, Hz): 1.26–1.59 (m, 6H, 3 CH_2_), 2.74–2.87 (m, 4H, 2 NCH_2_), 4.36 (br. s, 2H, NH_2_), 6.78 (d, *J* = 8.0, 1H, H-3), 7.63 (dd, *J* = 8.0, *J* = 2.2, 1H, H-4), 8.20 (d, *J* = 2.2, 1H, H-6), 8.44 (br. s, 1H, NH). Anal. calcd. for C_10_H_16_N_4_O_2_S %: C 46.86; H 6.29; N 21.86; S 12.51. Found, %: C 47.00; H 6.28; N 21.92; S 12.48.

*2-Hydrazinyl-5-(4-methylpiperidin-1-ylsulfonyl)pyridine* (**11d**). According to General Procedure F, 2-chloro-5-(4-methylpiperidin-1-ylsulfonyl)pyridine **10d** (13.7 g, 50 mmol) yielded 2-hydrazinyl-5-(4-methylpiperidin-1-ylsulfonyl)pyridine **11d** (11.6 g, 86%), as a white solid, m.p. 151–153 °C. ^1^H-NMR spectrum δ, ppm (*J*, Hz): 0.86 (d, *J* = 7.0, 3H, CH_3_), 0.98–1.36 (m, 3H), 1.54–1.68 (m, 2H), 2.07–2.22 (m, 2H, NCH_2_), 3.44–3.56 (m, 2H, NCH_2_), 4.38 (br. s, 2H, NH_2_), 6.78 (d, *J* = 8.0, 1H, H-3), 7.62 (dd, *J* = 8.0, *J* = 2.2, 1H, H-4), 8.19 (d, *J* = 2.2, 1H, H-6), 8.48 (br. s, 1H, NH). Anal. calcd. for C_11_H_18_N_4_O_2_S %: C 48.87; H 6.71; N 20.72; S 11.86. Found, %: C 49.04; H 6.69; N 20.66; S 11.90.

*4-(6-Hydrazinylpyridin-3-ylsulfonyl)thiomorpholine* (**11e**)**.** According to General Procedure F, 4-(6-chloropyridin-3-ylsulfonyl)thiomorpholine **10e** (13.9 g, 50 mmol) yielded 4-(6-hydrazinylpyridin-3-ylsulfonyl)thiomorpholine **11e** (11.4 g, 83%), as a white solid, m.p. 147–149 °C. ^1^H-NMR spectrum δ, ppm (*J*, Hz): 2.56–2.68 (m, 4H, 2 SCH_2_), 3.00–3.20 (m, 4H, 2 NCH_2_), 4.44 (br. s, 2H, NH_2_), 6.78 (d, *J* = 8.0, 1H, H-3), 7.63 (dd, *J* = 8.0, *J* = 2.2, 1H, H-4), 8.22 (d, *J* = 2.2, 1H, H-6), 8.53 (br. s, 1H, NH). Anal. calcd. for C_9_H_14_N_4_O_2_S_2_ %: C 39.40; H 5.14; N 20.42; S 23.37. Found, %: C 39.29; H 5.13; N 20.36; S 23.44.

*2-Hydrazinyl-3-(pyrrolidin-1-ylsulfonyl)pyridine* (**11f**)**.** According to General Procedure F, 2-chloro-3-(pyrrolidin-1-ylsulfonyl)pyridine **10f** (12.3 g, 50 mmol) yielded 2-hydrazinyl-3-(pyrrolidin-1-ylsulfonyl)pyridine **11f** (10.3 g, 85%), as a white solid, m.p. 142–144 °C. ^1^H-NMR spectrum δ, ppm (*J*, Hz): 1.67–1.73 (m, 4H, 2 CH_2_), 3.15–3.19 (m, 4H, 2 NCH_2_), 4.59 (br. s, 2H, NH_2_), 7.41 (dd, *J* = 8.0, *J* = 7.2, 1H, H-5), 8.21 (dd, *J* = 8.0, *J* = 1.5, 1H, H-6), 8.51 (dd, *J* = 7.2, *J* = 1.5, 1H, H-4), 9.10 (t, *J* = 4.0, 1H, NH). Anal. calcd. for C_9_H_14_N_4_O_2_S %: C 44.61; H 5.82; N 23.12; S 13.23. Found, %: C 44.49; H 5.81; N 23.07; S 13.18.

*1-(2-Hydrazinylpyridin-3-ylsulfonyl)-1,2,3,4-tetrahydroquinoline* (**11g**)**.** According to General Procedure F, 1-(2-chloropyridin-3-ylsulfonyl)-1,2,3,4-tetrahydroquinoline **10g** (15.4 g, 50 mmol) yielded 1-(2-hydrazinylpyridin-3-ylsulfonyl)-1,2,3,4-tetrahydroquinoline **11g** (12.5 g, 82%), as a white solid, m.p. 167–169 °C. ^1^H-NMR spectrum δ, ppm (*J*, Hz): 1.84–1.92 (m, 2H, THQ 3-CH_2_), 2.49–2.76 (m, 2H, THQ 4-CH_2_), 3.37–3.47 (m, 2H, THQ 2-CH_2_), 4.59 (br. s, 2H, NH_2_), 6.56 (t, *J* = 7.6, 1H, Ar H), 6.64 (d, *J* = 7.6, 1H, Ar H), 7.02 (d, *J* = 7.6, 1H, Ar H), 7.13 (t, *J* = 7.6, 1H, Ar H), 7.55 (dd, *J* = 8.0, *J* = 7.2, 1H, H-5), 8.32 (dd, *J* = 8.0, *J* = 1.5, 1H, H-6), 8.70 (dd, *J* = 7.2, *J* = 1.5, 1H, H-4), 9.30 (t, *J* = 4.0, NH). Anal. calcd. for C_14_H_16_N_4_O_2_S %: C 55.25; H 5.30; N 18.41; S 10.53. Found, %: C 55.38; H 5.28; N 18.37; S 10.49.

#### 3.3.7. Synthesis of Sulfonamido[1,2,4]Triazolo[4,3-a]Pyridin-3-Ones **12a**–**e**. General Procedure G

CDI (4.86 g, 30 mmol) was added to a stirred solution of corresponding 2-hydrazinyl-pyridinesulfonamide **11a**–**g** (20 mmol) in anhydrous dioxane (50 mL). The reaction mixture was refluxed for 8 h. After cooling, the reaction mixture was diluted with water (100 mL). The precipitate that formed was filtered and recrystallized from a mixture of DMF (10 mL) and EtOH (20 mL). Yields of sulfonamido[1,2,4]triazolo[4,3-*a*]pyridin-3-ones **12a**–**e** were 80–86%.

*8-(Piperidin-1-ylsulfonyl)-[1,2,4]triazolo[4,3-a]pyridin-3(2H)-one* (**12a**). According to General Procedure G, 2-hydrazinyl-3-(piperidin-1-ylsulfonyl)pyridine **11a** (5.13 g, 20 mmol) yielded 8-(piperidin-1-ylsulfonyl)-[1,2,4]triazolo[4,3-*a*]pyridin-3(2*H*)-one **12a** (4.63 g, 82%), as a white solid, m.p. 284–286 °C. ^1^H-NMR spectrum δ, ppm (*J*, Hz): 1.32–1.54 (m, 6H, 3 CH_2_), 3.08–3.18 (m, 4H, 2 NCH_2_), 6.66 (t, *J* = 7.6, 1H, H-6), 7.67 (d, *J* = 7.6, 1H, H-7), 8.02 (d, *J* = 7.6, 1H, H-5), 12.72 (s, 1H, NH). Anal. calcd. for C_11_H_14_N_4_O_3_S %: C 46.80; H 5.00; N 19.84; S 11.36. Found, %: C 46.62; H 4.98; N 19.79; S 11.40.

*8-(Morpholinosulfonyl)-[1,2,4]triazolo[4,3-a]pyridin-3(2H)-one* (**12b**). According to General Procedure G, 4-(2-hydrazinylpyridin-3-ylsulfonyl)morpholine **11b** (5.16 g, 20 mmol) yielded 8-(morpholinosulfonyl)-[1,2,4]triazolo[4,3-*a*]pyridin-3(2*H*)-one **12b** (4.83 g, 85%), as a white solid, m.p. 290–292 °C (160–161 °C [L]). ^1^H-NMR spectrum δ, ppm (*J*, Hz): 3.12–3.18 (m, 4H, 2 NCH_2_), 3.52–3.60 (m, 4H, 2 OCH_2_), 6.67 (t, *J* = 7.6, 1H, H-6), 7.68 (d, *J* = 7.6, 1H, H-7), 8.08 (d, *J* = 7.6, 1H, H-5), 12.40 (br. s, 1H, NH). Anal. calcd. for C_10_H_12_N_4_O_4_S %: C 42.25; H 4.25; N 19.71; S 11.28. Found, %: C 42.40; H 4.24; N 21.64; S 11.26.

*6-(Piperidin-1-ylsulfonyl)-[1,2,4]triazolo[4,3-a]pyridin-3(2H)-one* (**12c**)**.** According to General Procedure G, 2-hydrazinyl-5-(piperidin-1-ylsulfonyl)pyridine **11c** (5.13 g, 20 mmol) yielded 6-(piperidin-1-ylsulfonyl)-[1,2,4]triazolo[4,3-*a*]pyridin-3(2*H*)-one **12c** (4.87 g, 86%), as a white solid, m.p. 303–305 °C. ^1^H-NMR spectrum δ, ppm (*J*, Hz): 1.30–1.56 (m, 6H, 3 CH_2_), 2.95–3.09 (m, 4H, 2 NCH_2_), 7.20–7.38 (m, 2H, H-7 + H-8), 8.00 (s, 1H, H-5), 12.55 (br. s, 1H, NH). Anal. calcd. for C_11_H_14_N_4_O_3_S %: C 46.80; H 5.00; N 19.84; S 11.36. Found, %: C 46.67; H 4.99; N 19.87; S 11.31.

*6-(4-Methylpiperidin-1-ylsulfonyl)-[1,2,4]triazolo[4,3-a]pyridin-3(2H)-one* (**12d**)**.** According to General Procedure G, 2-hydrazinyl-5-(4-methylpiperidin-1-ylsulfonyl)pyridine **11d** (5.41 g, 20 mmol) yielded 6-(4-methylpiperidin-1-ylsulfonyl)-[1,2,4]triazolo[4,3-*a*]pyridin-3(2*H*)-one **12d** (4.74 g, 80%), as a white solid, m.p. 279–281 °C. ^1^H-NMR spectrum δ, ppm (*J*, Hz): 0.86 (d, *J* = 7.0, 3H, CH_3_), 0.98–1.36 (m, 3H), 1.54–1.72 (m, 2H), 2.17–2.42 (m, 2H, NCH_2_), 3.44–3.60 (m, 2H, NCH_2_), 7.20–7.40 (m, 2H, H-7 + H-8), 8.06 (s, 1H, H-5), 12.60 (br. s, 1H, NH). Anal. calcd. for C_12_H_16_N_4_O_3_S %: C 48.64; H 5.44; N 18.91; S 10.82. Found, %: C 48.81; H 5.46; N 18.86; S 10.79.

*6-(Thiomorpholinosulfonyl)-[1,2,4]triazolo[4,3-a]pyridin-3(2H)-one* (**12e**). According to General Procedure G, 4-(6-hydrazinylpyridin-3-ylsulfonyl)thiomorpholine **11e** (5.49 g, 20 mmol) yielded 6-(thiomorpholinosulfonyl)-[1,2,4]triazolo[4,3-*a*]pyridin-3(2*H*)-one **12e** (5.05 g, 84%), as a white solid, m.p. >320 °C. ^1^H-NMR spectrum δ, ppm (*J*, Hz): 2.52–2.78 (m, 4H, 2 SCH_2_), 3.34–3.50 (m, 4H, 2 NCH_2_), 7.20–7.38 (m, 2H, H-7 + H-8), 8.07 (s, 1H, H-5), 12.75 (br. s, 1H, NH). Anal. calcd. for C_10_H_12_N_4_O_3_S_2_ %: C 39.99; H 4.03; N 18.65; S 21.35. Found, %: C 40.12; H 4.01; N 18.59; S 21.27.

#### 3.3.8. Synthesis of 2-Benzyl-Sulfonamido[1,2,4]Triazolo[4,3-*a*]Pyridin-3-Ones **13a**–**j**. General Procedure H

A powder of dry K_2_CO_3_ (0.42 g, 3 mmol) was added to a stirred solution of corresponding sulfonamido[1,2,4]triazolo[4,3-*a*]pyridin-3-one **12a**–**e** (1 mmol) in anhydrous DMF (5 mL). Then, appropriate benzyl chloride **7a,e**–**g,i**–**n** (1.1 mmol) was added and the reaction mixture was heated at 100 °C for 2 h. After cooling, the reaction mixture was diluted with water (25 mL). The precipitate that formed was filtered off, washed with water (5 mL) and recrystallized from a mixture of DMF (5 mL) and EtOH (20 mL). The yields of 2-benzyl-sulfonamido[1,2,4]triazolo[4,3-*a*]pyridin-3-ones **13a–j** were 60–65%.

*2-(3-Chlorobenzyl)-8-(piperidin-1-ylsulfonyl)-[1,2,4]triazolo[4,3-a]pyridin-3(2H)-one* (**13a**). According to General Procedure H, 8-(piperidin-1-ylsulfonyl)-[1,2,4]triazolo[4,3-*a*]pyridin-3(2*H*)-one **12a** (0.28 g, 1 mmol) and 3-chlorobenzyl chloride **7a** (0.18 g, 1.1 mmol) yielded 2-(3-chlorobenzyl)-8-(piperidin-1-ylsulfonyl)-[1,2,4]triazolo[4,3-*a*]pyridin-3(2*H*)-one **13a** (0.25 g 62%), as a yellow solid, m.p. 176–177 °C, ^1^H-NMR spectrum, δ, ppm (*J*, Hz): 1.29–1.47 (m, 6H, 3 CH_2_), 3.04–3.15 (m, 4H, 2 NCH_2_), 5.17 (s, 2H, CH_2_), 6.72 (t, *J* = 7.6, 1H, H-6), 7.27–7.40 (m, 4H, Ar H), 7.71 (d, *J* = 7.6, 1H, H-7), 8.12 (d, *J* = 7.6, 1H, H-5). ^13^C-NMR spectrum, δ, ppm: 22.9 (piperidine 4-CH_2_), 25.1 (2C, piperidine 3,5-CH_2_), 46.3 (2C, piperidine 2,6-CH_2_), 48.1 (Bn CH_2_), 109.5, 124.7, 126.6, 127.6, 127.7, 129.0, 130.3, 133.2, 134.8, 136.8, 138.7, 147.8 (C=O). LC/MS *m*/*z* (%): 407.3 [M + H]^+^ (100.0). Anal. calcd. for C_18_H_19_ClN_4_O_3_S %: C 53.13, H 4.71, N 13.77, S 7.88. Found, %: C 52.96, H 4.73, N 13.84, S 7.82.

*2-(Benzo[d][1,3]dioxol-5-ylmethyl)-8-(piperidin-1-ylsulfonyl)-[1,2,4]triazolo[4,3-a]pyridin-3(2H)-one* (**13b**). According to General Procedure H, 8-(piperidin-1-ylsulfonyl)-[1,2,4]triazolo[4,3-*a*]pyridin-3(2*H*)-one **12a** (0.28 g, 1 mmol) and 5-(chloromethyl)benzo[d][1,3]dioxole **7i** (0.19 g, 1.1 mmol) yielded 2-(benzo[d][1,3]dioxol-5-ylmethyl)-8-(piperidin-1-ylsulfonyl)-[1,2,4]triazolo[4,3-*a*]pyridin-3(2*H*)-one **13b** (0.25 g, 60%), as a yellow solid, m.p. 160–161°C, ^1^H-NMR spectrum, δ, ppm (*J*, Hz): 1.38–1.52 (m, 6H, 3 CH_2_), 3.06–3.18 (m, 4H, 2 NCH_2_), 5.17 (s, 2H, CH_2_), 5.96 (s, 2H, OCH_2_O), 6.70 (t, *J* = 7.6, 1H, H-6), 6.79–6.86 (m, 2H, Ph H-3,4), 6.88 (s, 1H, Ph H-6), 7.68 (d, *J* = 7.6, 1H, H-7), 8.06 (d, *J* = 7.6, 1H, H-5). ^13^C-NMR spectrum, δ, ppm: 23.4 (piperidine 4-CH_2_), 25.5 (2C, piperidine 3,5-CH_2_), 46.7 (2C, piperidine 2,6-CH_2_), 49.2 (Bn CH_2_), 101.5 (OCH_2_O), 108.5, 108.9, 109.8, 122.0, 129.1, 130.4, 134.9, 135.0, 136.9, 147.2, 147.8, 148.1 (C=O). LC/MS *m*/*z* (%): 417.2 [M + H]^+^ (100.0). Anal. calcd. for C_19_H_20_N_4_O_5_S %: C 54.80, H 4.84, N 13.45, S 7.70. Found, %: C 54.97, H 4.87, N 13.48, S 7.75.

*2-(3,5-Difluorobenzyl)-8-(piperidin-1-ylsulfonyl)-[1,2,4]triazolo[4,3-a]pyridin-3(2H)-one* (**13c**). According to General Procedure H, 8-(piperidin-1-ylsulfonyl)-[1,2,4]triazolo[4,3-*a*]pyridin-3(2*H*)-one **12a** (0.28 g, 1 mmol) and 3,5-difluorobenzyl chloride **7j** (0.18 g, 1.1 mmol) yielded 2-(3,5-difluorobenzyl)-8-(piperidin-1-ylsulfonyl)-[1,2,4]triazolo[4,3-*a*]pyridin-3(2*H*)-one **13c** (0.26 g, 64%), as a yellow solid, m.p. 221–222 °C, ^1^H-NMR spectrum, δ, ppm (*J*, Hz): 1.30–1.48 (m, 6H, 3 CH_2_), 3.09–3.17 (m, 4H, 2 NCH_2_), 5.18 (s, 2H, CH_2_), 6.73 (t, *J* = 7.6, 1H, H-6), 7.05 (d, *J* = 9.2, 2H, Ph H-2,6), 7.09 (t, *J* = 9.6, 1H, Ph H-4), 7.73 (d, *J* = 7.6, 1H, H-7), 8.11 (d, *J* = 7.6, 1H, H-5). ^13^C-NMR spectrum, δ, ppm: 22.9 (piperidine 4-CH_2_), 25.0 (2C, piperidine 3,5-CH_2_), 46.3 (2C, piperidine 2,6-CH_2_), 47.8 (Bn CH_2_), 103.0 (t, *J_C-F_* = 25.7 Hz, Ph C-4), 109.4, 110.9 (dd, *J_C-F_* = 22.6 Hz, 7.9 Hz, 2C, Ph C-2,6), 124.6, 129.1, 135.0, 137.0, 140.8 (t, *J_C-F_* = 9.4 Hz, Ph C-1), 147.9 (C=O), 162.4 (dd, *J_C-F_* = 246.0 Hz, *J_C-F_* = 13.2 Hz, 2C, Ph C-3,5). LC/MS *m*/*z* (%): 409.5 [M + H]^+^ (100.0). Anal. calcd. for C_18_H_18_F_2_N_4_O_3_S %: C 52.93, H 4.44, N 13.72, S 7.85. Found, %: C 53.07, H 4.46, N 13.76, S 7.90.

*2-(2-Chlorobenzyl)-8-(morpholinosulfonyl)-[1,2,4]triazolo[4,3-a]pyridin-3(2H)-one* (**13d**). According to General Procedure H, 8-(morpholinosulfonyl)-[1,2,4]triazolo[4,3-*a*]pyridin-3(2*H*)-one **12b** (0.28 g, 1 mmol) and 2-chlorobenzyl chloride **7k** (0.18 g, 1.1 mmol) yielded 2-(3-chlorobenzyl)-8-(piperidin-1-ylsulfonyl)-[1,2,4]triazolo[4,3-*a*]pyridin-3(2*H*)-one **13a** (0.25 g, 62%), as a yellow solid, m.p. 226–227 °C, ^1^H-NMR spectrum, δ, ppm (*J*, Hz): 3.08–3.12 (m, 4H, 2 NCH_2_), 3.46–3.50 (m, 4H, 2 OCH_2_), 5.20 (s, 2H, Bn CH_2_), 6.74 (t, *J* = 7.6, 1H, H-6), 7.28–7.35 (m, *3*H, Ar H), 7.45 (d, *J* = 7.6, 1H, Ar H), 7.72 (d, *J* = 7.6, 1H, H-7), 8.12 (d, *J* = 7.6, 1H, H-5). ^13^C NMR spectrum, δ, ppm: 46.0 (2C, 2 NCH_2_), 47.2 (Bn CH_2_), 66.1 (2C, 2 OCH_2_), 109.9, 124.8, 127.7, 129.6, 129.8, 130.2, 131.1, 132.9, 133.8, 135.6, 137.1, 148.2 (C=O). LC/MS *m*/*z* (%): 409.1 [M + H]^+^ (100.0). Anal. calcd. for C_17_H_17_ClN_4_O_4_S %: C 49.94, H 4.19, N 13.70, S 7.84. Found, %: C 50.09, H 4.17, N 13.76, S 7.78.

*8-(Morpholinosulfonyl)-2-[4-(2-oxopyrrolidin-1-yl)benzyl]-[1,2,4]triazolo[4,3-a]pyridin-3(2H)-one* (**13e**). According to General Procedure H, 8-(morpholinosulfonyl)-[1,2,4]triazolo[4,3-*a*]pyridin-3(2*H*)-one **12b** (0.28 g, 1 mmol) and 1-(4-(chloromethyl)phenyl)pyrrolidin-2-one **7l** (0.23 g, 1.1 mmol) yielded 8-(morpholinosulfonyl)-2-[4-(2-oxopyrrolidin-1-yl)benzyl]-[1,2,4]triazolo[4,3-*a*]pyridin-3(2*H*)-one **13e** (0.28 g, 61%), as a yellow solid, m.p. 258–260 °C (dec.), ^1^H-NMR spectrum, δ, ppm (*J*, Hz): 2.10 (qn, *J* = 7.0, 2H, pyrrolidine 4-CH_2_), 2.42–2.48 (m, 2H, pyrrolidine 3-CH_2_), 3.12–3.18 (m, 4H, 2 NCH_2_), 3.46–3.52 (m, 4H, 2 OCH_2_), 3.82 (t, *J* = 7.0, 2H, pyrrolidine 5-CH_2_), 5.08 (s, 2H, Bn CH_2_), 6.72 (t, *J* = 7.6, 1H, H-6), 7.34 (d, *J* = 7.6, 2H, Bn H-3,5), 7.61 (d, *J* = 7.6, 2H, Bn H-2,6), 7.71 (d, *J* = 7.6, 1H, H-7), 8.09 (d, *J* = 7.6, 1H, H-5). ^13^C NMR spectrum, δ, ppm: 17.8 (pyrrolidine 4-CH_2_), 32.7 (pyrrolidine 3-CH_2_), 46.1 (2C, 2 NCH_2_), 48.5 (pyrrolidine 5-CH_2_), 49.0 (Bn CH_2_), 66.1 (2C, 2 OCH_2_), 109.8, 119.9 (2C), 128.8 (2C), 129.4, 129.5, 131.9, 135.4, 136.8, 136.9, 139.6 (C=O), 174.2 (pyrrolidone C=O). LC/MS *m*/*z* (%): 458.1 [M + H]^+^ (100.0). Anal. calcd. for C_21_H_23_N_5_O_5_S %: C 55.13, H 5.07, N 15.31, S 7.01. Found, %: C 52.98, H 5.10, N 15.26, S 6.98.

*2-(4-Chlorobenzyl)-6-(piperidin-1-ylsulfonyl)-[1,2,4]triazolo[4,3-a]pyridin-3(2H)-one* (**13f**). According to General Procedure H, 6-(piperidin-1-ylsulfonyl)-[1,2,4]triazolo[4,3-*a*]pyridin-3(2*H*)-one **12c** (0.28 g, 1 mmol) and 4-chlorobenzyl chloride **7m** (0.18 g, 1.1 mmol) yielded 2-(4-chlorobenzyl)-6-(piperidin-1-ylsulfonyl)-[1,2,4]triazolo[4,3-*a*]pyridin-3(2*H*)-one **13f** (0.26 g, 64%), as a white solid, m.p. 173–174 °C, ^1^H-NMR spectrum, δ, ppm (*J*, Hz): 1.30–1.52 (m, 6H, 3 CH_2_), 2.95–3.09 (m, 4H, 2 NCH_2_), 5.15 (s, 2H, CH_2_), 7.28–7.35 (m, 6H, Ar H), 8.07 (s, 1H, H-5). ^13^C NMR spectrum, δ, ppm: 23.3 (piperidine 4-CH_2_), 25.3 (2C, piperidine 3,5-CH_2_), 46.7 (2C, piperidine 2,6-CH_2_), 48.8 (Bn CH_2_), 116.8, 122.2, 127.3, 127.4, 128.9 (2C), 130.3 (2C), 132.9, 135.5, 140.7, 148.4 (C=O). LC/MS *m*/*z* (%): 407.1 [M + H]^+^ (100.0). Anal. calcd. for C_18_H_19_ClN_4_O_3_S %: C 53.13, H 4.71, N 13.77, S 7.88. Found, %: C 52.98, H 4.70, N 13.81, S 7.83.

*2-(3-Methylbenzyl)-6-(4-methylpiperidin-1-ylsulfonyl)-[1,2,4]triazolo[4,3-a]pyridin-3(2H)-one* (**13g**). According to General Procedure H, 6-(4-methylpiperidin-1-ylsulfonyl)-[1,2,4]triazolo[4,3-*a*]pyridin-3(2*H*)-one **12d** (0.30 g, 1 mmol) and 3-methylbenzyl chloride **7f** (0.15 g, 1.1 mmol) yielded 2-(3-methylbenzyl)-6-(4-methylpiperidin-1-ylsulfonyl)-[1,2,4]triazolo[4,3-*a*]pyridin-3(2*H*)-one **13g** (0.24 g, 60%), as a beige solid, m.p. 145–146 °C, ^1^H-NMR spectrum, δ, ppm (*J*, Hz): 0.86 (d, *J* = 7.0, 3H, piperidine 4-CH_3_), 1.00–1.70 (m, 5H), 2.35 (s, 3H, Ph CH_3_), 3.52–3.67 (m, 4H, 2 NCH_2_), 5.09 (s, 2H, CH_2_), 7.15–7.40 (m, 6H, Ar H), 8.06 (s, 1H, H-5). ^13^C NMR spectrum, δ, ppm:19.2 (CH_3_), 21.6 (piperidine 4-CH_3_), 29.6 (piperidine 4-CH), 33.4(2C, piperidine 3,5-CH_2_), 46.2 (2C, piperidine 2,6-CH_2_), 47.5 (Bn CH_2_), 116.8, 122.1, 126.3, 127.1, 127.4, 128.3, 129.5, 130.6, 134.5, 136.6, 140.6, 148.3 (C=O). LC/MS *m*/*z* (%): 401.3 [M + H]^+^ (100.0). Anal. calcd. for C_20_H_24_N_4_O_3_S %: C 59.98, H 6.04, N 13.99, S 8.01. Found, %: C 60.11, H 6.05, N 13.94, S 7.98.

*2-(2-Fluorobenzyl)-6-(4-methylpiperidin-1-ylsulfonyl)-[1,2,4]triazolo[4,3-a]pyridin-3(2H)-one* (**13h**). According to General Procedure H, 6-(4-methylpiperidin-1-ylsulfonyl)-[1,2,4]triazolo[4,3-*a*]pyridin-3(2*H*)-one **12d** (0.30 g, 1 mmol) and 2-fluorobenzyl chloride **7g** (0.16 g, 1.1 mmol) yielded 2-(2-fluorobenzyl)-6-(4-methylpiperidin-1-ylsulfonyl)-[1,2,4]triazolo[4,3-*a*]pyridin-3(2*H*)-one **13h** (0.25 g, 63%), as a beige solid, m.p. 150–151 °C, ^1^H-NMR spectrum, δ, ppm (*J*, Hz): 0.86 (d, *J* = 7.0, 3H, piperidine 4-CH_3_), 1.00–1.70 (m, 5H), 3.52–3.67 (m, 4H, 2 NCH_2_), 5.16 (s, 2H, Bn CH_2_), 7.12–7.40 (m, 6H, Ar H), 8.06 (s, 1H, H-5). ^13^C NMR spectrum, δ, ppm: 21.6 (piperidine 4-CH_3_), 29.6 (piperidine 4-CH), 33.4 (2C, piperidine 3,5-CH_2_), 43.4 (2C, piperidine 2,6-CH_2_), 46.2 (Bn CH_2_), 115.8 (d, *J_C-F_* = 19.8 Hz), 116.8, 122.3, 123.2 (d, *J_C-F_* = 14.6 Hz), 124.9, 127.3, 127.4, 130.7, 131.2 (d, *J_C-F_* = 8.4 Hz), 140.7, 148.3 (C=O), 160.5 (d, *J_C-F_* = 251.0 Hz, Bn C-2). LC/MS *m*/*z* (%): 405.2 [M + H]^+^ (100.0). Anal. calcd. for C_19_H_21_FN_4_O_3_S %: C 56.42, H 5.23, N 13.85, S 7.93. Found, %: C 56.61, H 5.25, N 13.90, S 7.88.

*2-(3-Fluorobenzyl)-6-(thiomorpholinosulfonyl)-[1,2,4]triazolo[4,3-a]pyridin-3(2H)-one* (**13i**). According to General Procedure H, 6-(thiomorpholinosulfonyl)-[1,2,4]triazolo[4,3-*a*]pyridin-3(2*H*)-one **12e** (0.30 g, 1 mmol) and 3-fluorobenzyl chloride **7e** (0.16 g, 1.1 mmol) yielded 2-(3-fluorobenzyl)-6-(thiomorpholinosulfonyl)-[1,2,4]triazolo[4,3-*a*]pyridin-3(2*H*)-one **13i** (0.29 g, 65%), as a white solid, m.p. 154–155 °C, ^1^H-NMR spectrum, δ, ppm (*J*, Hz): 2.60–2.72 (m, 4H, 2 SCH_2_), 3.36–3.50 (m, 4H, 2 NCH_2_), 5.15 (s, 2H, Bn CH_2_), 7.03–7.43 (m, 6H, Ar H), 8.11 (s, 1H, H-5). ^13^C NMR spectrum, δ, ppm: 27.0 (2C, 2 SCH_2_), 47.9 (2C, 2 NCH_2_), 48.8 (Bn CH_2_), 114.9 (d, *J_C-F_* = 21.4 Hz, Bn C-4), 115.1, 117.1, 122.5, 124.3, 126.9, 127.8, 131.0 (d, *J_C-F_* = 8.4 Hz), 139.3, 140.8, 148.5 (C=O), 162.6 (d, *J_C-F_* = 244.9 Hz, Bn C-3). LC/MS *m*/*z* (%): 409.1 [M + H]^+^ (100.0). Anal. calcd. for C_17_H_17_FN_4_O_3_S_2_ %: C 49.99, H 4.19, N 13.72, S 15.70. Found, %: C 50.08, H 4.21, N 13.78, S 15.63.

*2-(2-Chloro-4-fluorobenzyl)-6-(thiomorpholinosulfonyl)-[1,2,4]triazolo[4,3-a]pyridin-3(2H)-one* (**13j**). According to General Procedure H, 6-(thiomorpholinosulfonyl)-[1,2,4]triazolo[4,3-*a*]pyridin-3(2*H*)-one **12e** (0.30 g, 1 mmol) and 2-chloro-4-fluorobenzyl chloride **7n** (0.20 g, 1.1 mmol) yielded 2-(2-chloro-4-fluorobenzyl)-6-(thiomorpholinosulfonyl)-[1,2,4]triazolo[4,3-*a*]pyridin-3(2*H*)-one **13j** (0.26 g, 64%), as a white solid, m.p. 190–191 °C, ^1^H-NMR spectrum, δ, ppm (*J*, Hz): 2.60–2.72 (m, 4H, 2 SCH_2_), 3.46–3.50 (m, 4H, 2 NCH_2_), 5.20 (s, 2H, Bn CH_2_), 7.18–7.53 (m, 5H, Ar H), 8.07 (s, 1H, H-5). ^13^C NMR spectrum, δ, ppm: 27.0 (2C, 2 SCH_2_), 46.6 (2C, 2 NCH_2_), 47.9 (Bn CH_2_), 115.0 (d, *J_C-F_* = 20.6 Hz), 117.1, 122.6, 126.0, 127.0, 127.8, 130.2, 131.6, 132.5 (d, *J_C-F_* = 9.2 Hz), 140.9, 148.4 (C=O), 162.0 (d, *J_C-F_* = 248.0 Hz, Bn C-4). LC/MS *m*/*z* (%): 443.4 [M + H]^+^ (100.0). Anal. calcd. for C_17_H_16_ClFN_4_O_3_S_2_. Calculated, %: C 46.10, H 3.64, N 12.65, S 14.48. Found, %: C 45.94, H 3.62, N 12.59, S 14.53.

#### 3.3.9. Synthesis of 3-Thioxo-Sulfonamido[1,2,4]Triazolo[4,3**-***a*]Pyridines **14a**–**c**. General Procedure I

Corresponding 2-hydrazinyl-pyridinesulfonamide **11a,f,g** (20 mmol) was dissolved in DMF (50 mL). Then, triethylamine (9.76 mL, 70 mmol) and carbon disulfide (1.0 mL, 40 mmol) were added. The reaction mixture was heated at 40 °C for 2 h, and then the temperature was raised to 90 °C. The obtained solution was heated at 90 °C for 6 h. After cooling to room temperature, the reaction mixture was acidified by AcOH (4.0 mL, 70 mmol) and diluted with water (200 mL). The precipitate that formed was filtered, washed with water (10 mL) and recrystallized from a mixture of DMF (20 mL) and EtOH (20 mL). The yields of 3-thioxo-sulfonamido[1,2,4]triazolo[4,3-*a*]pyridines **14a**–**c** were 67–74%.

*8-(Piperidin-1-ylsulfonyl)-[1,2,4]triazolo[4,3-a]pyridin-3(2H)-thione* (**14a**)**.** According to General Procedure I, 2-hydrazinyl-3-(piperidin-1-ylsulfonyl)pyridine **11a** (5.13 g, 20 mmol) yielded 8-(piperidin-1-ylsulfonyl)-[1,2,4]triazolo[4,3-*a*]pyridine-3(2*H*)-thione **14a** (4.30 g, 72%), as a yellow solid, m.p. 303–305 °C. ^1^H-NMR spectrum δ, ppm (*J*, Hz): 1.46–1.54 (m, 6H, 3 CH_2_), 3.16–3.24 (m, 4H, 2 NCH_2_), 7.05 (dd, *J* = 8.0, *J* = 7.2, 1H, H-6), 7.90 (dd, *J* = 7.0, *J* = 1.5, 1H, H-7), 8.45 (dd, *J* = 8.0, *J* = 1.5, 1H, H-5), 14.85 (s, 1H, NH). Anal. calcd. for C_11_H_14_N_4_O_2_S_2_ %: C 44.28; H 4.73; N 18.78; S 21.49. Found, %: C 44.43; H 4.72; N 18.72; S 21.41.

*8-(Pyrrolidin-1-ylsulfonyl)-[1,2,4]triazolo[4,3-a]pyridin-3(2H)-thione* (**14b**)**.** According to General Procedure I, 2-hydrazinyl-3-(pyrrolidin-1-ylsulfonyl)pyridine **11f** (4.85 g, 20 mmol) yielded 8-(pyrrolidin-1-ylsulfonyl)-[1,2,4]triazolo[4,3-*a*]pyridine-3(2*H*)-thione **14b** (4.21 g, 74%), as a yellow solid, m.p. 303–305 °C. ^1^H-NMR spectrum δ, ppm (*J*, Hz): 1.62–1.78 (m, 4H, 2 CH_2_), 3.32–3.40 (m, 4H, 2 NCH_2_), 7.06 (t, *J* = 7.2, 1H, H-6), 7.93 (d, *J* = 7.2, 1H, H-7), 8.44 (d, *J* = 7.6, 1H, H-5), 14.70 (s, 1H, NH). Anal. calcd. for C_10_H_12_N_4_O_2_S_2_ %: C 42.24; H 4.25; N 19.70; S 22.55. Found, %: C 42.36; H 4.27; N 19.63; S 22.49.

*8-(3,4-Dihydroquinolin-1(2H)-ylsulfonyl)-[1,2,4]triazolo[4,3-a]pyridin-3(2H)-thione* (**14c**)**.** According to General Procedure I, 1-(2-hydrazinylpyridin-3-ylsulfonyl)-1,2,3,4-tetrahydroquinoline **11g** (6.09 g, 20 mmol) yielded 8-(3,4-dihydroquinolin-1(2*H*)-ylsulfonyl)-[1,2,4]triazolo[4,3-*a*]pyridine-3(2*H*)-thione **14c** (4.64 g, 67%), as a yellow solid, m.p. >320 °C. ^1^H-NMR spectrum δ, ppm (*J*, Hz): 1.82–1.94 (m, 2H, THQ 3-CH_2_), 2.49–2.79 (m, 2H, THQ 4-CH_2_), 3.37–3.47 (m, 2H, THQ 2-CH_2_), 6.56 (t, *J* = 7.6, 1H, Ar H), 6.64 (d, *J* = 7.6, 1H, Ar H), 7.02 (d, *J* = 7.6, 1H, Ar H), 7.08–7.16 (m, 2H, H-6 + Ar H), 7.93 (d, *J* = 7.2, 1H, H-7), 8.51 (d, *J* = 7.6, 1H, H-5), 14.95 (s, 1H, NH). Anal. calcd. for C_15_H_14_N_4_O_2_S_2_ %: C 52.01; H 4.07; N 16.17; S 18.51. Found, %: C 51.84; H 4.09; N 16.23; S 18.45.

#### 3.3.10. Synthesis of 3-Thio-Sulfonamido[1,2,4]Triazolo[4,3-*a*]Pyridines **15a–f**. General Procedure J

The corresponding benzyl chloride **7a,c,h,k,o,p** (1.1 mmol) was added to the stirred solution of 3-thioxo-sulfonamido[1,2,4]triazolopyridine **14a**–**c** (1 mmol) and triethylamine (0.17 mL, 1.2 mmol) in anhydrous DMF (5 mL). The reaction mixture was heated at 80 °C for 2 h. After cooling, the reaction mixture was diluted with water (25 mL). The precipitate that formed was filtered off, washed with water (5 mL) and recrystallized from a mixture of DMF (5 mL) and EtOH (20 mL). The yields of 3-thio-sulfonamido[1,2,4]triazolo[4,3-*a*]pyridines **15a–f** were 81–94%.

*3-(4-Methoxybenzylthio)-8-(piperidin-1-ylsulfonyl)-[1,2,4]triazolo[4,3-a]pyridine* (**15a**). According to General Procedure H, 8-(piperidin-1-ylsulfonyl)-[1,2,4]triazolo[4,3-*a*]pyridine-3(2*H*)-thione **14a** (0.30 g, 1 mmol) and 4-methoxybenzyl chloride **7c** (0.17 g, 1.1 mmol) yielded 3-(4-methoxybenzylthio)-8-(piperidin-1-ylsulfonyl)-[1,2,4]triazolo[4,3-*a*]pyridine **15a** (0.34 g, 81%), as a cream solid, m.p. 169–170 °C, ^1^H-NMR spectrum, δ, ppm (*J*, Hz): 1.30–1.52 (m, 6H, 3 CH_2_), 3.16–3.25 (m, 4H, 2 NCH_2_), 3.66 (s, 3H, OCH_3_), 4.28 (s, 2H, Bn CH_2_), 6.72 (d, *J* = 8.0, 2H, Bn H-3,5), 7.02–7.12 (m, 3H, H-6 + Bn H-2,6), 7.85 (d, *J* = 7.2, 1H, H-7), 8.38 (d, *J* = 7.2, 1H, H-5). ^13^C-NMR spectrum, δ, ppm: 23.0 (piperidine 4-CH_2_), 25.1 (2C, piperidine 3,5-CH_2_), 38.2 (Bn CH_2_), 46.6 (2C, piperidine 2,6-CH_2_), 55.1 (OCH_3_), 113.0, 113.8 (2C), 125.4, 128.3, 128.6, 130.1 (2C), 131.5, 141.5, 145.9, 158.7 (Bn C-4). LC/MS *m*/*z* (%): 419.4 [M + H]^+^ (100.0). Anal. calcd. for C_19_H_22_N_4_O_3_S_2_ %: C 54.53, H 5.30, N 13.39, S 15.32. Found, %: C 54.36, H 5.29, N 13.42, S 15.38.

*3-(3-Bromobenzylthio)-8-(piperidin-1-ylsulfonyl)-[1,2,4]triazolo[4,3-a]pyridine* (**15b**). According to General Procedure H, 8-(piperidin-1-ylsulfonyl)-[1,2,4]triazolo[4,3-*a*]pyridine-3(2*H*)-thione **14a** (0.30 g, 1 mmol) and 3-bromobenzyl chloride **7o** (0.23 g, 1.1 mmol) yielded 3-(3-bromobenzylthio)-8-(piperidin-1-ylsulfonyl)-[1,2,4]triazolo[4,3-*a*]pyridine **15b** (0.44 g, 94%), as a cream solid, m.p. 160–162 °C, ^1^H-NMR spectrum, δ, ppm (*J*, Hz): 1.32–1.52 (m, 6H, 3 CH_2_), 3.16–3.25 (m, 4H, 2 NCH_2_), 4.30 (s, 2H, Bn CH_2_), 7.05–7.23 (m, 3H, H-6 + Ar H), 7.05–7.23 (m, 2H, Ar H), 7.86 (d, *J* = 7.2, 1H, H-7), 8.40 (d, *J* = 7.2, 1H, H-5). ^13^C-NMR spectrum, δ, ppm: 23.0 (piperidine 4-CH_2_), 25.1 (2C, piperidine 3,5-CH_2_), 37.6 (Bn CH_2_), 46.6 (2C, piperidine 2,6-CH_2_), 113.1, 121.4, 125.4, 128.0, 128.2, 130.3, 130.6, 131.5, 131.6, 139.9, 141.1, 146.1. LC/MS *m*/*z* (%): 469.1 [M + H]^+^ (100.0). Anal. calcd. for C_18_H_19_BrN_4_O_2_S_2_ %: C 46.26, H 4.10, N 11.99, S 13.72. Found, %: C 46.11, H 4.09, N 12.04, S 13.68.

*3-(4-Methylbenzylthio)-8-(piperidin-1-ylsulfonyl)-[1,2,4]triazolo[4,3-a]pyridine* (**15c**). According to General Procedure H, 8-(piperidin-1-ylsulfonyl)-[1,2,4]triazolo[4,3-*a*]pyridine-3(2*H*)-thione **14a** (0.30 g, 1 mmol) and 4-methylbenzyl chloride **7p** (0.15 g, 1.1 mmol) yielded 3-(4-methylbenzylthio)-8-(piperidin-1-ylsulfonyl)-[1,2,4]triazolo[4,3-*a*]pyridine **15c** (0.37 g, 92%), as a cream solid, m.p. 155–156 °C, ^1^H-NMR spectrum, δ, ppm (*J*, Hz): 1.30–1.50 (m, 6H, 3 CH_2_), 2.18 (s, 3H, CH_3_), 3.16–3.25 (m, 4H, 2 NCH_2_), 4.28 (s, 2H, Bn CH_2_), 6.93–7.09 (m, 5H, H-6 + Ar H), 7.86 (d, *J* = 7.2, 1H, H-7), 8.38 (d, *J* = 7.2, 1H, H-5). ^13^C-NMR spectrum, δ, ppm: 20.6 (CH_3_), 23.0 (piperidine 4-CH_2_), 25.1 (piperidine CH_2_), 25.5 (piperidine CH_2_), 38.3 (Bn CH_2_), 46.6 (2C, piperidine 2,6-CH_2_), 112.9, 125.4, 128.3, 128.8 (2 C), 129.0 (2 C), 131.5, 133.7, 136.8, 141.4, 145.9. LC/MS *m*/*z* (%): 403.4 [M + H]^+^ (100.0). Anal. calcd. for C_19_H_22_N_4_O_2_S_2_ %: C 56.69, H 5.51, N 13.92, S 15.93. Found, %: C 56.86, H 5.49, N 13.87, S 15.88.

*3-(3-Chlorobenzylthio)-8-(pyrrolidin-1-ylsulfonyl)-[1,2,4]triazolo[4,3-a]pyridine* (**15d**). According to General Procedure H, 8-(pyrrolidin-1-ylsulfonyl)-[1,2,4]triazolo[4,3-*a*]pyridine-3(2*H*)-thione **14b** (0.28 g, 1 mmol) and 3-chlorobenzyl chloride **7a** (0.18 g, 1.1 mmol) yielded 3-(3-chlorobenzylthio)-8-(pyrrolidin-1-ylsulfonyl)-[1,2,4]triazolo[4,3-*a*]pyridine **15d** (0.38 g, 93%), as a cream solid, m.p. 143–145 °C, ^1^H-NMR spectrum, δ, ppm (*J*, Hz): 1.59–1.74 (m, 4H, 2 CH_2_), 3.40–3.52 (m, 4H, 2 NCH_2_), 4.31 (s, 2H, Bn CH_2_), 7.02–7.22 (m, 5H, H-6 + Ar H), 7.91 (d, *J* = 7.2, 1H, H-7), 8.40 (d, *J* = 7.2, 1H, H-5). ^13^C-NMR spectrum, δ, ppm: 25.3 (2C, pyrrolidine 3,4-CH_2_), 37.7 (Bn CH_2_), 47.9 (2C, pyrrolidine 2,5-CH_2_), 113.1, 125.3, 127.4, 127.6, 128.2, 128.7, 130.2, 132.4, 132.9, 139.7, 141.1, 146.2. LC/MS *m*/*z* (%): 409.4 [M + H]^+^ (100.0). Anal. calcd. for C_17_H_17_ClN_4_O_2_S_2_ %: C 49.93, H 4.19, N 13.70, S 15.68. Found, %: C 50.11, H 4.18, N 13.65, S 15.72.

*3-(2-Chlorobenzylthio)-8-(pyrrolidin-1-ylsulfonyl)-[1,2,4]triazolo[4,3-a]pyridine* (**15e**). According to General Procedure H, 8-(pyrrolidin-1-ylsulfonyl)-[1,2,4]triazolo[4,3-*a*]pyridine-3(2*H*)-thione **14b** (0.28 g, 1 mmol) and 2-chlorobenzyl chloride **7k** (0.18 g, 1.1 mmol) yielded 3-(2-chlorobenzylthio)-8-(pyrrolidin-1-ylsulfonyl)-[1,2,4]triazolo[4,3-*a*]pyridine **15e** (0.36 g, 88%), as a cream solid, m.p. 164–166 °C, ^1^H-NMR spectrum, δ, ppm (*J*, Hz): 1.59–1.74 (m, 4H, 2 CH_2_), 3.40–3.52 (m, 4H, 2 NCH_2_), 4.34 (s, 2H, Bn CH_2_), 7.06–7.26 (m, 4H, H-6 + Ar H), 7.32 (d, *J* = 7.6, 1H, Ar H), 7.91 (d, *J* = 7.2, 1H, H-7), 8.38 (d, *J* = 7.2, 1H, H-5). ^13^C-NMR spectrum, δ, ppm: 25.3 (2C, pyrrolidine 3,4-CH_2_), 36.4 (Bn CH_2_), 47.9 (2C, pyrrolidine 2,5-CH_2_), 113.2, 125.3, 127.3, 128.0, 129.4, 129.6, 131.4, 132.4, 132.9, 134.6, 140.7, 146.3. LC/MS *m*/*z* (%): 409.4 [M + H]^+^ (100.0). Anal. calcd. for C_17_H_17_ClN_4_O_2_S_2_ %: C 49.93, H 4.19, N 13.70, S 15.68. Found, %: C 50.07, H 4.21, N 13.68, S 15.73.

*1-(3-(4-Fluorobenzylthio)-[1,2,4]triazolo[4,3-a]pyridin-8-ylsulfonyl)-1,2,3,4-tetrahydroquinoline* (**15f**). According to General Procedure H, 8-(3,4-dihydroquinolin-1(2*H*)-ylsulfonyl)-[1,2,4]triazolo[4,3-*a*]pyridine-3(2*H*)-thione **14c** (0.35 g, 1 mmol) and 4-fluorobenzyl chloride **7h** (0.16 g, 1.1 mmol) yielded 1-(3-(4-fluorobenzylthio)-[1,2,4]triazolo[4,3-*a*]pyridin-8-ylsulfonyl)-1,2,3,4-tetrahydroquinoline **15f** (0.42 g, 92%), as a cream solid, m.p. 113–115 °C, ^1^H-NMR spectrum, δ, ppm (*J*, Hz): 2.75–2.85 (m, 2H, THQ 3-CH_2_), 3.60–3.70 (m, 2H, CH_2_), 4.29 (s, 2H, Bn CH_2_), 4.46–4.53 (m, 2H, CH_2_), 6.90 (t, *J* = 7.6, 2H, Bn H-3,5), 7.02–7.12 (m, 5H, H-6 + Ar H), 7.18 (t, *J* = 7.6, 2H, Bn H-2,6), 7.96 (d, *J* = 7.2, 1H, H-7), 8.38 (d, *J* = 7.2, 1H, H-5). ^13^C-NMR spectrum, δ, ppm: 28.3 (THQ 3-CH_2_), 37.6 (Bn CH_2_), 43.6 (THQ CH_2_), 46.7 (THQ CH_2_), 113.1, 115.2 (d, *J_CF_* = 21.9 Hz, 2C, Bn C-3,5), 125.4, 126.1, 126.2, 126.6, 128.4, 128.6, 130.9 (d, *J_CF_* = 8.3 Hz, 2C, Bn C-2,6), 131.8, 132.0, 133.3, 133.4, 141.3, 145.9, 161.5 (d, *J_CF_* = 244 Hz, Bn C-4). LC/MS *m*/*z* (%): 455.3 [M + H]^+^ (100.0). Anal. calcd. for C_22_H_19_FN_4_O_2_S_2_ %: C 58.13, H 4.21, N 12.33, S 14.11. Found, %: C 57.98, H 4.19, N 12.38, S 14.14.

### 3.4. Antimalarial Activity Assay

The in vitro antiplasmodial activity of all synthesized compounds was evaluated at the Laboratory of Microbiology, Parasitology and Hygiene (LMPH, University of Antwerp, Belgium) against red blood cells of chloroquine-resistant P. falciparum 2/K strain.

Stock solutions were prepared in 100% DMSO at 20 mg/mL just prior to screening. For the tests, appropriate reference drugs were used as positive control: tamoxifen for MRC-5, chloroquine for P. falciparum. Reference drugs were either obtained from the fine chemical supplier Sigma or from WHO-TDR.

The integrated panel of microbial screens and standard screening methodologies was adopted as previously described [48]. All assays were performed in triplicate at the Laboratory of Microbiology, Parasitology and Hygiene at the University of Antwerp, Belgium. Compounds were tested at five concentrations (64, 16, 4, 1 and 0.25 µg/mL) to establish a full dose-titration and determination of the IC_50_ (inhibitory concentration 50%). The in-test concentration of DMSO did not exceed 0.5%. The selectivity antiprotozoal potential was assessed by simultaneous evaluation of cytotoxicity on a fibroblast (MRC-5) cell line. The criterion for activity was an IC_50_ < 10 µg/mL and a selectivity index of ≥4.

#### 3.4.1. Antiplasmodial Activity

Chloroquine-resistant P. falciparum 2/K 1-strain was cultured in human erythrocytes O+ at 37 °C under a low oxygen atmosphere (3% O_2_, 4% CO_2_, and 93% N_2_) in RPMI-1640, supplemented with 10% human serum. Infected human red blood cells (200 µL, 1% parasitaemia, 2% haematocrit) were added to each well and incubated for 72 h. After incubation, test plates were frozen at −20 °C. Parasite multiplication was measured by the Malstat method [48,49].

#### 3.4.2. Cytotoxicity Assay

MRC-5 SV2 cells were cultivated in MEM, supplemented with L-glutamine (20 mMol), 16.5 mMol sodium hydrogen carbonate and 5% FCS. For the assay, 104 MRC-5 cells/well were seeded onto the test plates containing the pre-diluted sample and incubated at 37 °C and 5% CO_2_ for 72 h. Cell viability was assessed fluorimetrically after 4 h of addition of resazurin. Fluorescence was measured (excitation 550 nm, emission 590 nm) and the results were expressed as % reduction in cell viability compared to control.

## 4. Conclusions

In order to develop new antimalarial drugs, a virtual library with three randomization points for novel [1,2,4] triazolo [4,3-a] pyridines bearing a sulfonamide moiety was designed. By means of virtual screening and molecular docking techniques using falcipain-2 as a target, 25 final compounds were chosen for synthesis and in vitro screening for their antimalarial activity against *Plasmodium falciparum*. 3-Ethyl-*N*-(3-fluorobenzyl)-*N*-(4-methoxyphenyl)-[1,2,4]triazolo[4,3-*a*]pyridine-6-sulfonamide and 2-(3-chlorobenzyl)-8-(piperidin-1-ylsulfonyl)-[1,2,4]triazolo[4,3-*a*]pyridin-3(2*H*)-one showed, in vitro, good antimalarial activity with inhibitory concentration IC_50_ = 2.24 and 4.98 μM, respectively. This new series of compounds may serve as a starting point for future antimalarial drug discovery programs.

## Figures and Tables

**Table 1:** Binding affinity parameters and molecular properties of 25 hit compounds filtered by structure-based virtual screening (SBVS) and docking using protein–ligand complex FP-2/E64, chain D with (pdb: 3 bpf).

Ligand	Binding Energy (kcal/mol)	Binding Affinity Score	cLogP	Mol Weight	Hydrogen Bond Donors (HBA)	Hydrogen Bond Acceptors (HBD)	Topological Polar Surface Area (TPSA)
**13e**	−15.9	−3.94	1.52	457.51	6	0	107.91
**13g**	−15.6	−8.43	3.22	400.50	4	0	76.68
**8h**	−15.5	−16.71	5.02	430.89	5	0	67.57
**8d**	−15.5	−10.46	3.69	440.48	5	0	107.01
**15f**	−15.5	−9.37	5.10	454.55	6	0	67.57
**13c**	−15.3	−20.01	2.95	408.43	6	0	76.68
**15c**	−15.3	−16.10	4.30	402.54	5	0	67.57
**8a**	−15.2	−9.45	4.85	434.85	6	0	67.57
**8b**	−15.2	−9.27	5.46	434.56	4	0	67.57
**13h**	−15.2	−9.23	3.05	404.46	5	0	76.68
**8f**	−15.1	−13.39	4.68	410.47	5	0	67.57
**13f**	−14.9	−15.77	3.32	406.89	4	0	76.68
**8g**	−14.9	−15.02	5.02	430.89	5	0	67.57
**13a**	−14.9	−6.36	3.32	406.89	4	0	76.68
**8e**	−14.8	−12.24	4.63	440.50	6	0	76.80
**13b**	−14.8	−1.08	3.33	416.46	4	0	102.96
**15b**	−14.7	−24.47	4.75	467.41	5	0	67.57
**13i**	−14.7	−5.87	2.37	408.47	6	0	76.68
**15a**	−14.6	−21.83	4.00	418.54	6	0	76.80
**8c**	−14.6	−10.35	4.85	436.53	5	0	76.80
**8i**	−14.4	−12.31	5.02	430.89	5	0	67.57
**15d**	−14.3	−20.25	4.26	408.93	5	0	67.57
**3j**	−14.3	−8.49	3.02	442.92	6	0	76.68
**13d**	−14.3	−2.59	2.17	408.86	5	0	85.91
**15e**	−14.0	−23.42	4.26	408.93	5	0	67.57
**E64**	−10.8	−7.81	-5.49	359.43	5	6	182.20
**Chloroquine**	−11.0	−5.59	4.81	319.90	1	1	28.16

**Table 2:** Compounds presented in Scheme 2.

Compound	Position of SO_2_R	Ar_1_	R_1_	Ar_2_
**1a**	3-SO_2_Cl	—	—	—
**1b**	5-SO_2_Cl	—	—	—
**2a**	—	3,5-diFPh	—	—
**2b**	—	3,5-diMePh	—	—
**2c**	—	3-MePh	—	—
**2d**	—	4-MeOPh	—	—
**2e**	—	4-FPh	—	—
**2f**	—	3-ClPh	—	—
**2g**	—	4-ClPh	—	—
**3a**	3-SO_2_Ar_1_	3,5-diFPh	—	—
**3b**	3-SO_2_Ar_1_	3,5-diMePh	—	—
**3c**	5-SO_2_Ar_1_	3-MePh	—	—
**3d**	5-SO_2_Ar_1_	4-MeOPh	—	—
**3e**	5-SO_2_Ar_1_	4-FPh	—	—
**3f**	5-SO_2_Ar_1_	3-ClPh	—	—
**3g**	5-SO_2_Ar_1_	4-ClPh	—	—
**4a**	3-SO_2_Ar_1_	3,5-diFPh	—	—
**4b**	3-SO_2_Ar_1_	3,5-diMePh	—	—
**4c**	5-SO_2_Ar_1_	3-MePh	—	—
**4d**	5-SO_2_Ar_1_	4-MeOPh	—	—
**4e**	5-SO_2_Ar_1_	4-FPh	—	—
**4f**	5-SO_2_Ar_1_	3-ClPh	—	—
**4g**	5-SO_2_Ar_1_	4-ClPh	—	—
**5a**	—	—	H	—
**5b**	—	—	Me	—
**5c**	—	—	Et	—
**6a**	8-SO_2_Ar_1_	3,5-diFPh	H	—
**6b**	8-SO_2_Ar_1_	3,5-diMePh	Me	—
**6c**	6-SO_2_Ar_1_	3-MePh	Me	—
**6d**	6-SO_2_Ar_1_	4-MeOPh	Et	—
**6e**	6-SO_2_Ar_1_	4-FPh	Me	—
**6f**	6-SO_2_Ar_1_	3-ClPh	Me	—
**6g**	6-SO_2_Ar_1_	4-ClPh	Me	—
**7a**	—	—	—	3-ClPh
**7b**	—	—	—	2,5-diMePh
**7c**	—	—	—	4-MeOPh
**7d**	—	—	—	5-methoxycarbonyl-(furan-2-yl)
**7e**	—	—	—	3-FPh
**7f**	—	—	—	3-MePh
**7g**	—	—	—	2-FPh
**7h**	—	—	—	4-FPh
**8a**	8-SO_2_Ar_1_	3,5-diFPh	H	3-ClPh
**8b**	8-SO_2_Ar_1_	3,5-diMePh	Me	2,5-diMePh
**8c**	8-SO_2_Ar_1_	3,5-diMePh	Me	4-MeOPh
**8d**	6-SO_2_Ar_1_	3-MePh	Me	5-methoxycarbonyl-(furan-2-yl)
**8e**	6-SO_2_Ar_1_	4-MeOPh	Et	3-FPh
**8f**	6-SO_2_Ar_1_	4-FPh	Me	3-MePh
**8g**	6-SO_2_Ar_1_	4-FPh	Me	3-ClPh
**8h**	6-SO_2_Ar_1_	3-ClPh	Me	2-FPh
**8i**	6-SO_2_Ar_1_	4-ClPh	Me	4-FPh

**Table 3:** Compounds presented in the Scheme 3.

Compound	Position of SO_2_R	R_3_NR_4_	Ar_2_
**1a**	3-SO_2_Cl	—	—
**1b**	5-SO_2_Cl	—	—
**7i**	—	—	benzo[d][1,3]dioxol-5-yl
**7j**	—	—	3,5-diFPh
**7k**	—	—	2-ClPh
**7l**	—	—	4-(2-oxopyrrolidin-1-yl)phenyl
**7m**	—	—	4-ClPh
**7n**	—	—	2-Cl-4-FPh
**7o**	—	—	3-BrPh
**7p**	—	—	4-MePh
**9a**	—	*N*-piperidinyl	—
**9b**	—	*N*-morpholinyl	—
**9c**	—	*N*-(4-methyl)piperidinyl	—
**9d**	—	*N*-thiomorpholinyl	—
**9e**	—	*N*-pyrrolidinyl	—
**9f**	—	*N*-1,2,3,4-tetrahydroquinolinyl	—
**10a**	3-SO_2_NR_3_R_4_	*N*-piperidinyl	—
**10b**	3-SO_2_NR_3_R_4_	*N*-morpholinyl	—
**10c**	5-SO_2_NR_3_R_4_	*N*-piperidinyl	—
**10d**	5-SO_2_NR_3_R_4_	*N*-(4-methyl)piperidinyl	—
**10e**	3-SO_2_NR_3_R_4_	*N*-thiomorpholinyl	—
**10f**	3-SO_2_NR_3_R_4_	*N*-pyrrolidinyl	—
**10g**	3-SO_2_NR_3_R_4_	*N*-1,2,3,4-tetrahydroquinolinyl	—
**11a**	3-SO_2_NR_3_R_4_	*N*-piperidinyl	—
**11b**	3-SO_2_NR_3_R_4_	*N*-morpholinyl	—
**11c**	5-SO_2_NR_3_R_4_	*N*-piperidinyl	—
**11d**	5-SO_2_NR_3_R_4_	*N*-(4-methyl)piperidinyl	—
**11e**	3-SO_2_NR_3_R_4_	*N*-thiomorpholinyl	—
**11f**	3-SO_2_NR_3_R_4_	*N*-pyrrolidinyl	—
**11g**	3-SO_2_NR_3_R_4_	*N*-1,2,3,4-tetrahydroquinolinyl	—
**12a**	8-SO_2_NR_3_R_4_	*N*-piperidinyl	—
**12b**	8-SO_2_NR_3_R_4_	*N*-morpholinyl	—
**12c**	6-SO_2_NR_3_R_4_	*N*-piperidinyl	—
**12d**	6-SO_2_NR_3_R_4_	*N*-(4-methyl)piperidinyl	—
**12e**	6-SO_2_NR_3_R_4_	*N*-thiomorpholinyl	—
**13a**	8-SO_2_NR_3_R_4_	*N*-piperidinyl	3-ClPh
**13b**	8-SO_2_NR_3_R_4_	*N*-piperidinyl	benzo[d][1,3]dioxol-5-yl
**13c**	8-SO_2_NR_3_R_4_	*N*-piperidinyl	3,5-diFPh
**13d**	8-SO_2_NR_3_R_4_	*N*-morpholinyl	2-ClPh
**13e**	8-SO_2_NR_3_R_4_	*N*-morpholinyl	4-(2-oxopyrrolidin-1-yl)phenyl
**13f**	6-SO_2_NR_3_R_4_	*N*-piperidinyl	4-ClPh
**13g**	6-SO_2_NR_3_R_4_	*N*-(4-methyl)piperidinyl	3-MePh
**13h**	6-SO_2_NR_3_R_4_	*N*-(4-methyl)piperidinyl	2-FPh
**13i**	6-SO_2_NR_3_R_4_	*N*-thiomorpholinyl	3-FPh
**13j**	6-SO_2_NR_3_R_4_	*N*-thiomorpholinyl	2-Cl-4-FPh
**14a**	8-SO_2_NR_3_R_4_	*N*-piperidinyl	—
**14b**	8-SO_2_NR_3_R_4_	*N*-pyrrolidinyl	—
**14c**	8-SO_2_NR_3_R_4_	*N*-1,2,3,4-tetrahydroquinolinyl	—
**15a**	8-SO_2_NR_3_R_4_	*N*-piperidinyl	4-MeOPh
**15b**	8-SO_2_NR_3_R_4_	*N*-piperidinyl	3-BrPh
**15c**	8-SO_2_NR_3_R_4_	*N*-piperidinyl	4-MePh
**15d**	8-SO_2_NR_3_R_4_	*N*-pyrrolidinyl	3-ClPh
**15e**	8-SO_2_NR_3_R_4_	*N*-pyrrolidinyl	2-ClPh
**15f**	8-SO_2_NR_3_R_4_	*N*-1,2,3,4-tetrahydroquinolinyl	4-FPh

**Table 4:** Integrated in vitro activity screening against *Plasmodium falciparum*. Results are expressed as 50% inhibitory concentration (IC_50_-value).

Compound No	Structure of Compound	IC_50_ (µM)	
		MRC-5	*P. falciparum*
**13a**	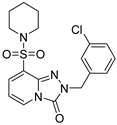	>64	2.24
**8e**	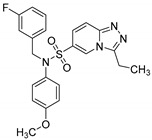	18.19	4.98
**8i**	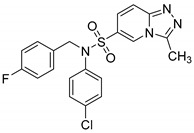	>64	8.00
**13g**	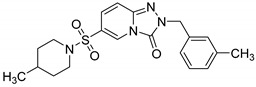	>64	8.00
**15b**	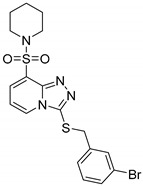	>64	8.52
**13b**	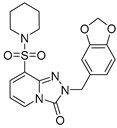	>64	8.93
**13f**	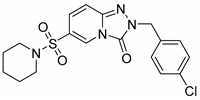	>64	9.62
**15a**	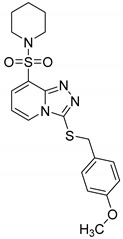	>64	9.71
**8c**	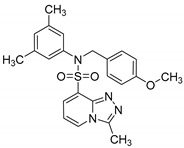	>64	9.75
**15c**	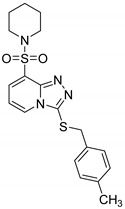	>64	10.73
**8d**	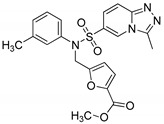	>64	11.31
**8b**	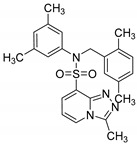	48.50	11.84
**8g**	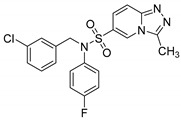	>64	12.13
**13d**	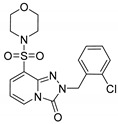	>64	13.59
**8f**	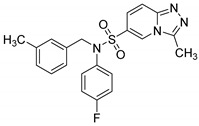	>64	13.93
**13e**	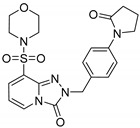	>64	27.86
**8h**	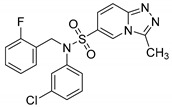	>64	32.00
**8a**	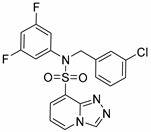	>64	40.32
**13h**	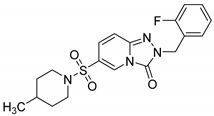	>64	40.32
**13c**	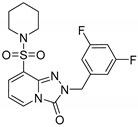	>64	>64
**13i**	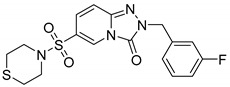	>64	>64
**13j**	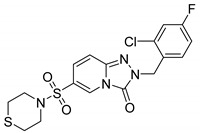	>64	>64
**15e**	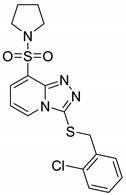	>64	>64
**15d**	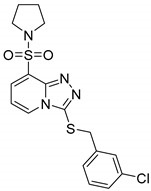	>64	>64
**15f**	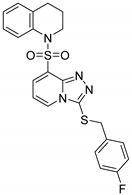	>64	>64

**Table 5:** Predicted pharmacokinetics of the compounds **13a** and **8e.**

Compound	GIA ^1^	BBBP ^2^	Pgp ^3^ Substrate	CYP450 Inhibition				
				CYP1A2	CYP2C19	CYP2C9	CYP2D6	CYP3A4
**13a**	High	No	No	No	Yes	Yes	No	No
**8e**	High	No	Yes	No	Yes	Yes	No	Yes

^1^ GIA = gastrointestinal absorption; ^2^ BBBP = blood brain barrier permeant; ^3^ Pgp = P-glycoprotein.

## Supplementary Materials

The ^1^H-NMR, ^13^C-NMR spectra of compounds and LCMS data are available online.
