# Supplementary material for: A Novel Series of [1,2,4]Triazolo[4,3-a]Pyridine Sulfonamides as Potential Antimalarial Agents: In Silico Studies, Synthesis and In Vitro Evaluation
Source: Molecules. 2020 Sep 30;25(19):4485. doi: 10.3390/molecules25194485 (PMC7582516; doi:10.3390/molecules25194485)
Supplement: Supplementary file 1 [file molecules-25-04485-s001.pdf]

A Novel Series of [1,2,4]Triazolo[4,3-a]pyridine Sulfonamides as Potential Antimalarial Agents: *In Silico* Studies, Synthesis and *In Vitro* Evaluation

Veronika R. Karpina [1], Svitlana S. Kovalenko [1], Sergiy M. Kovalenko [2,3], Oleksandr G. Drushlyak [1,*] Natalya D. Bunyatyan [3,4], Victoriya A. Georgiyants [1], Vladimir V. Ivanov [2], Thierry Langer [5], and Louis Maes [6]

1. Department of Pharmaceutical Chemistry, The National University of Pharmacy, 61002, 53 Pushkinska str., Kharkiv, Ukraine; v.karpina01@gmail.com (V.R.K.); s.kovalenko.nphau@gmail.com (S.S.K.); aldry18@hotmail.com (O.G.D.); vgeor@ukr.net (V.A.G.)
2. Department of Organic Chemistry, V.N. Karazin Kharkiv National University, 61022, 4 Svobody Sq., Kharkiv, Ukraine; kovalenko.sergiy.m@gmail.com (S.M.K); vivanov5001@gmail.com (V.V.I)
3. Federal State Autonomous Educational Institution of Higher Education I.M. Sechenov First Moscow State Medical University of the Ministry of Healthcare of the Russian Federation (Sechenovskiy University). 8 Trubeckaya st., Moscow, 119991, Russia; e-mail: ndbun@mail.ru
4. Federal State Budgetary Institution "Scientific Centre for Expert Evaluation of Medicinal Products" of the Ministry of Health of the Russian Federation, Petrovsky boulevard 8, bld. 2, Moscow, 127051, Russia, e-mail: ndbun@mail.ru
5. Department of Pharmaceutical Chemistry, University of Vienna, Althanstraße 14, A-1090, Vienna, Austria; e-mail: thierry.langer@univie.ac.at
6. Laboratory of Microbiology, Parasitology and Hygiene, University of Antwerp, Universiteitsplein 1, B-2610 Antwerp, Belgium; louis.maes@uantwerpen.be

* Correspondence: aldry18@hotmail.com; Tel.: +38-050-196-5129
Received: date; Accepted: date; Published: date

SUPPLEMENTARY MATERIAL

Figure S1. ^{1}H NMR spectrum (400 MHz, DMSO-d6) of 2-chloro-N-(3,5-difluorophenyl) pyridine-3-sulfonamide **3a**.
Figure S2. ^{1}H NMR spectrum (400 MHz, DMSO-d6) of 2-chloro-N-(3,5-dimethylphenyl) pyridine-3-sulfonamide **3b**.
Figure S3. ^{1}H NMR spectrum (200 MHz, DMSO-d6) of 2-chloro-N-(3-methylphenyl) pyridine-5-sulfonamide **3c**.
Figure S4. ^{1}H NMR spectrum (200 MHz, DMSO-d6) of 2-Chloro-N-(4-methoxyphenyl) pyridine-5-sulfonamide **3d**.
Figure S5. ^{1}H NMR spectrum (200 MHz, DMSO-d6) of 2-Chloro-N-(4-fluorophenyl) pyridine-5-sulfonamide **3e**.
Figure S6. ^{1}H NMR spectrum (200 MHz, DMSO-d6) of 2-Chloro-N-(3-chlorophenyl) pyridine-5-sulfonamide **3f.**
Figure S7. ^{1}H NMR spectrum (200 MHz, DMSO-d6) of 2-Chloro-N-(4-chlorophenyl) pyridine-5-sulfonamide **3g**.
Figure S8. ^{1}H NMR spectrum (400 MHz, DMSO-d6) of N-(3,5-difluorophenyl)-2-hydrazinylpyridine-3-sulfonamide **4a**.
Figure S9. ^{1}H NMR spectrum (400 MHz, DMSO-d6) of N-(3,5-dimethylphenyl)-2-hydrazinylpyridine-3-sulfonamide **4b**.
Figure S10. ^{1}H NMR spectrum (200 MHz, DMSO-d6) of 2-hydrazinyl-N-(3-methylphenyl) pyridine-5-sulfonamide **4c**.
Figure S11. ^{1}H NMR spectrum (200 MHz, DMSO-d6) of 2-hydrazinyl-N-(4-methoxyphenyl) pyridine-5-sulfonamide **4d**.
Figure S12. ^{1}H NMR spectrum (200 MHz, DMSO-d6) of N-(4-fluorophenyl)-2-hydrazinylpyridine-5-sulfonamide **4e**.
Figure S13. ^{1}H NMR spectrum (200 MHz, DMSO-d6) of N-(3-chlorophenyl)-2-hydrazinylpyridine-5-sulfonamide **4f**.
Figure S14. ^{1}H NMR spectrum (200 MHz, DMSO-d6) of N-(4-chlorophenyl)-2-hydrazinylpyridine-5-sulfonamide **4g**.
Figure S15. ^{1}H NMR spectrum (200 MHz, DMSO-d6) of N-(3,5-difluorophenyl)-[1,2,4]triazolo[4,3-a]pyridine-8-sulfonamide **6a**.
Figure S16. ^{1}H NMR spectrum (200 MHz, DMSO-d6) of N-(3,5-dimethylphenyl)-3-methyl-[1,2,4]triazolo[4,3-a]pyridine-8-sulfonamide **6b**.
Figure S17. ^{1}H NMR spectrum (200 MHz, DMSO-d6) of 3-methyl-N-(3-methyphenyl)-[1,2,4]triazolo[4,3-a]pyridine-6-sulfonamide **6c**.
Figure S18. ^{1}H NMR spectrum (200 MHz, DMSO-d6) of 3-ethyl-N-(4-methoxyphenyl)-[1,2,4]triazolo[4,3-a]pyridine-6-sulfonamide **6d**.
Figure S19. ^{1}H NMR spectrum (200 MHz, DMSO-d6) of N-(4-fluorophenyl)-3-methyl-[1,2,4]triazolo[4,3-a]pyridine-6-sulfonamide **6e**.
Figure S20. ^{1}H NMR spectrum (200 MHz, DMSO-d6) of N-(3-chlorophenyl)-3-methyl-[1,2,4]triazolo[4,3-a]pyridine-6-sulfonamide **6f**.
Figure S21. ^{1}H NMR spectrum (200 MHz, DMSO-d6) of N-(4-chlorophenyl)-3-methyl-[1,2,4]triazolo[4,3-a]pyridine-6-sulfonamide **6g**.
Figure S22. ^{1}H NMR spectrum (300 MHz, DMSO-d6) of N-(3-chlorobenzyl)-N-(3,5-difluorophenyl)-[1,2,4]triazolo[4,3-a]pyridine-8-sulfonamide **8a**.
Figure S23. ^{13}C NMR spectrum (100 MHz, DMSO-d6) of N-(3-chlorobenzyl)-N-(3,5-difluorophenyl)-[1,2,4]triazolo[4,3-a]pyridine-8-sulfonamide **8a**.
Figure S24. LC/MS data for N-(3-chlorobenzyl)-N-(3,5-difluorophenyl)-[1,2,4]triazolo[4,3-a]pyridine-8-sulfonamide **8a**.
Figure S25. ^{1}H NMR spectrum (300 MHz, DMSO-d6) of N-(2,5-dimethylbenzyl)-N-(3,5-dimethylphenyl)-3-methyl-[1,2,4]triazolo[4,3-a]pyridine-8-sulfonamide **8b**.

Figure S26. ^{13}C NMR spectrum (100 MHz, DMSO-d6) of N-(2,5-dimethylbenzyl)-N-(3,5-dimethylphenyl)-3-methyl-[1,2,4]triazolo[4,3-a]pyridine-8-sulfonamide **8b**.
Figure S27. LC/MS data for N-(2,5-dimethylbenzyl)-N-(3,5-dimethylphenyl)-3-methyl-[1,2,4]triazolo[4,3-a]pyridine-8-sulfonamide **8b**.
Figure S28. ^{1}H NMR spectrum (300 MHz, DMSO-d6) of N-(3,5-dimethylphenyl)-N-(4-methoxybenzyl)-3-methyl-[1,2,4]triazolo[4,3-a]pyridine-8-sulfonamide **8c**.
Figure S29. ^{13}C NMR spectrum (100 MHz, DMSO-d6) of N-(3,5-dimethylphenyl)-N-(4-methoxybenzyl)-3-methyl-[1,2,4]triazolo[4,3-a]pyridine-8-sulfonamide **8c**.
Figure S30. LC/MS data for N-(3,5-dimethylphenyl)-N-(4-methoxybenzyl)-3-methyl-[1,2,4]triazolo[4,3-a]pyridine-8-sulfonamide **8c**.
Figure S31. ^{1}H NMR spectrum (300 MHz, DMSO-d6) of Methyl 5-{[3-methyl-N-(3-methylphenyl)-[1,2,4]triazolo[4,3-a]pyridine-6-sulfonamido]methyl}furan-2-carboxylate **8d**.
Figure S32. ^{13}C NMR spectrum (100 MHz, DMSO-d6) of Methyl 5-{[3-methyl-N-(3-methylphenyl)-[1,2,4]triazolo[4,3-a]pyridine-6-sulfonamido]methyl}furan-2-carboxylate **8d**.
Figure S33. LC/MS data for Methyl 5-{[3-methyl-N-(3-methylphenyl)-[1,2,4]triazolo[4,3-a]pyridine-6-sulfonamido]methyl}furan-2-carboxylate **8d**.
Figure S34. ^{1}H NMR spectrum (300 MHz, DMSO-d6) of 3-Ethyl-N-(3-fluorobenzyl)-N-(4-methoxyphenyl)-[1,2,4]triazolo[4,3-a]pyridine-6-sulfonamide **8e**.
Figure S35. ^{13}C NMR spectrum (100 MHz, DMSO-d6) of 3-Ethyl-N-(3-fluorobenzyl)-N-(4-methoxyphenyl)-[1,2,4]triazolo[4,3-a]pyridine-6-sulfonamide **8e**.
Figure S36. LC/MS data for 3-Ethyl-N-(3-fluorobenzyl)-N-(4-methoxyphenyl)-[1,2,4]triazolo[4,3-a]pyridine-6-sulfonamide **8e.**
Figure S37. ^{1}H NMR spectrum (200 MHz, DMSO-d6) of N-(4-fluorophenyl)-3-methyl-N-(3-methylbenzyl)-[1,2,4]triazolo[4,3-a]pyridine-6-sulfonamide **8f**.
Figure S38. ^{13}C NMR spectrum (100 MHz, DMSO-d6) of N-(4-fluorophenyl)-3-methyl-N-(3-methylbenzyl)-[1,2,4]triazolo[4,3-a]pyridine-6-sulfonamide **8f**.
Figure S39. LC/MS data for N-(4-fluorophenyl)-3-methyl-N-(3-methylbenzyl)-[1,2,4]triazolo[4,3-a]pyridine-6-sulfonamide **8f**.
Figure S40. ^{1}H NMR spectrum (200 MHz, DMSO-d6) of N-(3-chlorobenzyl)-N-(4-fluorophenyl)-3-methyl-[1,2,4]triazolo[4,3-a]pyridine-6-sulfonamide **8g**.
Figure S41. ^{13}C NMR spectrum (100 MHz, DMSO-d6) of N-(3-chlorobenzyl)-N-(4-fluorophenyl)-3-methyl-[1,2,4]triazolo[4,3-a]pyridine-6-sulfonamide **8g**.
Figure S42. LC/MS data for N-(3-chlorobenzyl)-N-(4-fluorophenyl)-3-methyl-[1,2,4]triazolo[4,3-a]pyridine-6-sulfonamide **8g**.
Figure S43. ^{1}H NMR spectrum (200 MHz, DMSO-d6) of N-(3-chlorophenyl)-N-(2-fluorobenzyl)-3-methyl-[1,2,4]triazolo[4,3-a]pyridine-6-sulfonamide **8h**.
Figure S44. ^{13}C NMR spectrum (100 MHz, DMSO-d6) of N-(3-chlorophenyl)-N-(2-fluorobenzyl)-3-methyl-[1,2,4]triazolo[4,3-a]pyridine-6-sulfonamide **8h**.
Figure S45. LC/MS data for N-(3-chlorophenyl)-N-(2-fluorobenzyl)-3-methyl-[1,2,4]triazolo[4,3-a]pyridine-6-sulfonamide **8h**.
Figure S46. ^{1}H NMR spectrum (200 MHz, DMSO-d6) of N-(4-chlorophenyl)-N-(4-fluorobenzyl)-3-methyl-[1,2,4]triazolo[4,3-a]pyridine-6-sulfonamide **8i**.
Figure S47. ^{13}C NMR spectrum (100 MHz, DMSO-d6) of N-(4-chlorophenyl)-N-(4-fluorobenzyl)-3-methyl-[1,2,4]triazolo[4,3-a]pyridine-6-sulfonamide **8i.**
Figure S48. LC/MS data for N-(4-chlorophenyl)-N-(4-fluorobenzyl)-3-methyl-[1,2,4]triazolo[4,3-a]pyridine-6-sulfonamide **8i.**
Figure S49. ^{1}H NMR spectrum (400 MHz, DMSO-d6) of 2-chloro-3-(piperidin-1-ylsulfonyl)pyridine **10a**.
Figure S50. ^{1}H NMR spectrum (200 MHz, DMSO-d6) of 4-(2-chloropyridin-3-ylsulfonyl)morpholine **10b**.
Figure S51. ^{1}H NMR spectrum (200 MHz, DMSO-d6) of 2-chloro-5-(piperidin-1-ylsulfonyl)pyridine **10c**.

Figure S52. ^{1}H NMR spectrum (200 MHz, DMSO-d6) of 2-chloro-5-(4-methylpiperidin-1-ylsulfonyl)pyridine **10d**.
Figure S53. ^{1}H NMR spectrum (200 MHz, DMSO-d6) of 4-(6-chloropyridin-3-ylsulfonyl)thiomorpholine **10e**.
Figure S54. ^{1}H NMR spectrum (400 MHz, DMSO-d6) of 2-chloro-3-(pyrrolidin-1-ylsulfonyl)pyridine **10f.**
Figure S55. ^{1}H NMR spectrum (400 MHz, DMSO-d6) of 1-(2-chloropyridin-3-ylsulfonyl)-1,2,3,4-tetrahydroquinoline **10g**.
Figure S56. ^{1}H NMR spectrum (400 MHz, DMSO-d6) of 2-hydrazinyl-3-(piperidin-1-ylsulfonyl)pyridine **11a**.
Figure S57. ^{1}H NMR spectrum (400 MHz, DMSO-d6) of 4-(2-hydrazinylpyridin-3-ylsulfonyl)morpholine **11b**.
Figure S58. ^{1}H NMR spectrum (200 MHz, DMSO-d6) of 2-hydrazinyl-5-(piperidin-1-ylsulfonyl)pyridine **11c**.
Figure S59. ^{1}H NMR spectrum (200 MHz, DMSO-d6) of 2-hydrazinyl-5-(4-methylpiperidin-1-ylsulfonyl)pyridine **11d**.
Figure S60. ^{1}H NMR spectrum (200 MHz, DMSO-d6) of 4-(6-hydrazinylpyridin-3-ylsulfonyl)thiomorpholine **11e.**
Figure S61. ^{1}H NMR spectrum (400 MHz, DMSO-d6) of 2-hydrazinyl-3-(pyrrolidin-1-ylsulfonyl)pyridine **11f.**
Figure S62. ^{1}H NMR spectrum (400 MHz, DMSO-d6) of 1-(2-hydrazinylpyridin-3-ylsulfonyl)-1,2,3,4-tetrahydroquinoline **11g**.
Figure S63. ^{1}H NMR spectrum (200 MHz, DMSO-d6) of 8-(piperidin-1-ylsulfonyl)-[1,2,4]triazolo[4,3-a]pyridin-3(2H)-one **12a.**
Figure S64. ^{1}H NMR spectrum (200 MHz, DMSO-d6) of 8-(morpholinosulfonyl)-[1,2,4]triazolo[4,3-a]pyridin-3(2H)-one **12b**.
Figure S65. ^{1}H NMR spectrum (200 MHz, DMSO-d6) of 6-(piperidin-1-ylsulfonyl)-[1,2,4]triazolo[4,3-a]pyridin-3(2H)-one **12c**.
Figure S66. ^{1}H NMR spectrum (200 MHz, DMSO-d6) of 6-(4-methylpiperidin-1-ylsulfonyl)-[1,2,4]triazolo[4,3-a]pyridin-3(2H)-one **12d**.
Figure S67. ^{1}H NMR spectrum (200 MHz, DMSO-d6) of 6-(thiomorpholinosulfonyl)-[1,2,4]triazolo[4,3-a]pyridin-3(2H)-one **12e**.
Figure S68. ^{1}H NMR spectrum (300 MHz, DMSO-d6) of 2-(3-chlorobenzyl)-8-(piperidin-1-ylsulfonyl)-[1,2,4]triazolo[4,3-a]pyridin-3(2H)-one **13a**.
Figure S69. ^{13}C NMR spectrum (100 MHz, DMSO-d6) of 2-(3-chlorobenzyl)-8-(piperidin-1-ylsulfonyl)-[1,2,4]triazolo[4,3-a]pyridin-3(2H)-one **13a**.
Figure S70. LC/MS data for 2-(3-chlorobenzyl)-8-(piperidin-1-ylsulfonyl)-[1,2,4]triazolo[4,3-a]pyridin-3(2H)-one **13a**.
Figure S71. ^{1}H NMR spectrum (400 MHz, DMSO-d6) of 2-(benzo[d][1,3]dioxol-5-ylmethyl)-8-(piperidin-1-ylsulfonyl)-[1,2,4]triazolo[4,3-a]pyridin-3(2H)-one **13b**.
Figure S72. ^{13}C NMR spectrum (100 MHz, DMSO-d6) of 2-(benzo[d][1,3]dioxol-5-ylmethyl)-8-(piperidin-1-ylsulfonyl)-[1,2,4]triazolo[4,3-a]pyridin-3(2H)-one **13b**.
Figure S73. LC/MS data for 2-(benzo[d][1,3]dioxol-5-ylmethyl)-8-(piperidin-1-ylsulfonyl)-[1,2,4]triazolo[4,3-a]pyridin-3(2H)-one **13b**.
Figure S74. ^{1}H NMR spectrum (300 MHz, DMSO-d6) of 2-(3,5-difluorobenzyl)-8-(piperidin-1-ylsulfonyl)-[1,2,4]triazolo[4,3-a]pyridin-3(2H)-one **13c**.
Figure S75. ^{13}C NMR spectrum (100 MHz, DMSO-d6) of 2-(3,5-difluorobenzyl)-8-(piperidin-1-ylsulfonyl)-[1,2,4]triazolo[4,3-a]pyridin-3(2H)-one **13c**.
Figure S76. LC/MS data for 2-(3,5-difluorobenzyl)-8-(piperidin-1-ylsulfonyl)-[1,2,4]triazolo[4,3-a]pyridin-3(2H)-one **13c**.
Figure S77. ^{1}H NMR spectrum (400 MHz, DMSO-d6) of 2-(2-chlorobenzyl)-8-(morpholinosulfonyl)-[1,2,4]triazolo[4,3-a]pyridin-3(2H)-one **13d**.
Figure S78. ^{13}C NMR spectrum (100 MHz, DMSO-d6) of 2-(2-chlorobenzyl)-8-(morpholinosulfonyl)-[1,2,4]triazolo[4,3-a]pyridin-3(2H)-one **13d**.

Figure S79. LC/MS data for 2-(2-chlorobenzyl)-8-(morpholinosulfonyl)-[1,2,4]triazolo[4,3-a]pyridin-3(2H)-one **13d**.

Figure S80. ^{1}H NMR spectrum (400 MHz, DMSO-d6) of 8-(morpholinosulfonyl)-2-[4-(2-oxopyrrolidin-1-yl)benzyl]-[1,2,4]triazolo[4,3-a]pyridin-3(2H)-one **13e**.

Figure S81. ^{13}C NMR spectrum (100 MHz, DMSO-d6) of 8-(morpholinosulfonyl)-2-[4-(2-oxopyrrolidin-1-yl)benzyl]-[1,2,4]triazolo[4,3-a]pyridin-3(2H)-one **13e**.

Figure S82. LC/MS data for 8-(morpholinosulfonyl)-2-[4-(2-oxopyrrolidin-1-yl)benzyl]-[1,2,4]triazolo[4,3-a]pyridin-3(2H)-one **13e**.

Figure S83. ^{1}H NMR spectrum (400 MHz, DMSO-d6) of 2-(4-chlorobenzyl)-6-(piperidin-1-ylsulfonyl)-[1,2,4]triazolo[4,3-a]pyridin-3(2H)-one **13f**.

Figure S84. ^{13}C NMR spectrum (100 MHz, DMSO-d6) of 2-(4-chlorobenzyl)-6-(piperidin-1-ylsulfonyl)-[1,2,4]triazolo[4,3-a]pyridin-3(2H)-one **13f**.

Figure S85. LC/MS data for 2-(4-chlorobenzyl)-6-(piperidin-1-ylsulfonyl)-[1,2,4]triazolo[4,3-a]pyridin-3(2H)-one **13f**.

Figure S86. ^{1}H NMR spectrum (400 MHz, DMSO-d6) of 2-(3-methylbenzyl)-6-(4-methylpiperidin-1-ylsulfonyl)-[1,2,4]triazolo[4,3-a]pyridin-3(2H)-one **13g**.

Figure S87. ^{13}C NMR spectrum (100 MHz, DMSO-d6) of 2-(3-methylbenzyl)-6-(4-methylpiperidin-1-ylsulfonyl)-[1,2,4]triazolo[4,3-a]pyridin-3(2H)-one **13g**.

Figure S88. LC/MS data for 2-(3-methylbenzyl)-6-(4-methylpiperidin-1-ylsulfonyl)-[1,2,4]triazolo[4,3-a]pyridin-3(2H)-one **13g**.

Figure S89. ^{1}H NMR spectrum (400 MHz, DMSO-d6) of 2-(2-fluorobenzyl)-6-(4-methylpiperidin-1-ylsulfonyl)-[1,2,4]triazolo[4,3-a]pyridin-3(2H)-one **13h**.

Figure S90. ^{13}C NMR spectrum (100 MHz, DMSO-d6) of 2-(2-fluorobenzyl)-6-(4-methylpiperidin-1-ylsulfonyl)-[1,2,4]triazolo[4,3-a]pyridin-3(2H)-one **13h**.

Figure S91. LC/MS data for 2-(2-fluorobenzyl)-6-(4-methylpiperidin-1-ylsulfonyl)-[1,2,4]triazolo[4,3-a]pyridin-3(2H)-one **13h**.

Figure S92. ^{1}H NMR spectrum (400 MHz, DMSO-d6) of 2-(3-fluorobenzyl)-6-(thiomorpholinosulfonyl)-[1,2,4]triazolo[4,3-a]pyridin-3(2H)-one **13i**.

Figure S93. ^{13}C NMR spectrum (100 MHz, DMSO-d6) of 2-(3-fluorobenzyl)-6-(thiomorpholinosulfonyl)-[1,2,4]triazolo[4,3-a]pyridin-3(2H)-one **13i**.

Figure S94. LC/MS data for 2-(3-fluorobenzyl)-6-(thiomorpholinosulfonyl)-[1,2,4]triazolo[4,3-a]pyridin-3(2H)-one **13i**.

Figure S95. ^{1}H NMR spectrum (400 MHz, DMSO-d6) of 2-(2-chloro-4-fluorobenzyl)-6-(thiomorpholinosulfonyl)-[1,2,4]triazolo[4,3-a]pyridin-3(2H)-one **13j**.

Figure S96. ^{13}C NMR spectrum (100 MHz, DMSO-d6) of 2-(2-chloro-4-fluorobenzyl)-6-(thiomorpholinosulfonyl)-[1,2,4]triazolo[4,3-a]pyridin-3(2H)-one **13j**.

Figure S97. LC/MS data for 2-(2-chloro-4-fluorobenzyl)-6-(thiomorpholinosulfonyl)-[1,2,4]triazolo[4,3-a]pyridin-3(2H)-one **13j**.

Figure S98. ^{1}H NMR spectrum (400 MHz, DMSO-d6) of 8-(piperidin-1-ylsulfonyl)-[1,2,4]triazolo[4,3-a]pyridine-3(2H)-thione **14a**.

Figure S99. ^{1}H NMR spectrum (400 MHz, DMSO-d6) of 8-(pyrrolidin-1-ylsulfonyl)-[1,2,4]triazolo[4,3-a]pyridine-3(2H)-thione **14b**.

Figure S100. ^{1}H NMR spectrum (400 MHz, DMSO-d6) of 8-(3,4-dihydroquinolin-1(2H)-ylsulfonyl)-[1,2,4]triazolo[4,3-a]pyridine-3(2H)-thione **14c**.

Figure S101. ^{1}H NMR spectrum (300 MHz, DMSO-d6) of 3-(4-methoxybenzylthio)-8-(piperidin-1-ylsulfonyl)-[1,2,4]triazolo[4,3-a]pyridine **15a**.

Figure S102. ^{13}C NMR spectrum (100 MHz, DMSO-d6) of 3-(4-methoxybenzylthio)-8-(piperidin-1-ylsulfonyl)-[1,2,4]triazolo[4,3-a]pyridine **15a**.

Figure S103. LC/MS data for 3-(4-methoxybenzylthio)-8-(piperidin-1-ylsulfonyl)-[1,2,4]triazolo[4,3-a]pyridine **15a**.

Figure S104. ^{1}H NMR spectrum (300 MHz, DMSO-d6) of 3-(3-bromobenzylthio)-8-(piperidin-1-ylsulfonyl)-[1,2,4]triazolo[4,3-a]pyridine **15b**.
Figure S105. ^{13}C NMR spectrum (100 MHz, DMSO-d6) of 3-(3-bromobenzylthio)-8-(piperidin-1-ylsulfonyl)-[1,2,4]triazolo[4,3-a]pyridine **15b**.
Figure S106. LC/MS data for 3-(3-bromobenzylthio)-8-(piperidin-1-ylsulfonyl)-[1,2,4]triazolo[4,3-a]pyridine **15b**.
Figure S107. ^{1}H NMR spectrum (300 MHz, DMSO-d6) of 3-(4-methylbenzylthio)-8-(piperidin-1-ylsulfonyl)-[1,2,4]triazolo[4,3-a]pyridine **15c**.
Figure S108. ^{13}C NMR spectrum (100 MHz, DMSO-d6) of 3-(4-methylbenzylthio)-8-(piperidin-1-ylsulfonyl)-[1,2,4]triazolo[4,3-a]pyridine **15c**.
Figure S109. LC/MS data for 3-(4-methylbenzylthio)-8-(piperidin-1-ylsulfonyl)-[1,2,4]triazolo[4,3-a]pyridine **15c**.
Figure S110. ^{1}H NMR spectrum (300 MHz, DMSO-d6) of 3-(3-chlorobenzylthio)-8-(pyrrolidin-1-ylsulfonyl)-[1,2,4]triazolo[4,3-a]pyridine **15d**.
Figure S111. ^{13}C NMR spectrum (100 MHz, DMSO-d6) of 3-(3-chlorobenzylthio)-8-(pyrrolidin-1-ylsulfonyl)-[1,2,4]triazolo[4,3-a]pyridine **15d**.
Figure S112. LC/MS data for 3-(3-chlorobenzylthio)-8-(pyrrolidin-1-ylsulfonyl)-[1,2,4]triazolo[4,3-a]pyridine **15d**.
Figure S113. ^{1}H NMR spectrum (300 MHz, DMSO-d6) of 3-(2-chlorobenzylthio)-8-(pyrrolidin-1-ylsulfonyl)-[1,2,4]triazolo[4,3-a]pyridine **15e**.
Figure S114. ^{13}C NMR spectrum (100 MHz, DMSO-d6) of 3-(2-chlorobenzylthio)-8-(pyrrolidin-1-ylsulfonyl)-[1,2,4]triazolo[4,3-a]pyridine **15e**.
Figure S115. LC/MS data for 3-(2-chlorobenzylthio)-8-(pyrrolidin-1-ylsulfonyl)-[1,2,4]triazolo[4,3-a]pyridine **15e**.
Figure S116. ^{1}H NMR spectrum (300 MHz, DMSO-d6) of 1-(3-(4-fluorobenzylthio)-[1,2,4]triazolo[4,3-a]pyridin-8-ylsulfonyl)-1,2,3,4-tetrahydroquinoline **15f**.
Figure S117. ^{13}C NMR spectrum (100 MHz, DMSO-d6) of 1-(3-(4-fluorobenzylthio)-[1,2,4]triazolo[4,3-a]pyridin-8-ylsulfonyl)-1,2,3,4-tetrahydroquinoline **15f**.
Figure S118. LC/MS data for 1-(3-(4-fluorobenzylthio)-[1,2,4]triazolo[4,3-a]pyridin-8-ylsulfonyl)-1,2,3,4-tetrahydroquinoline **15f**.

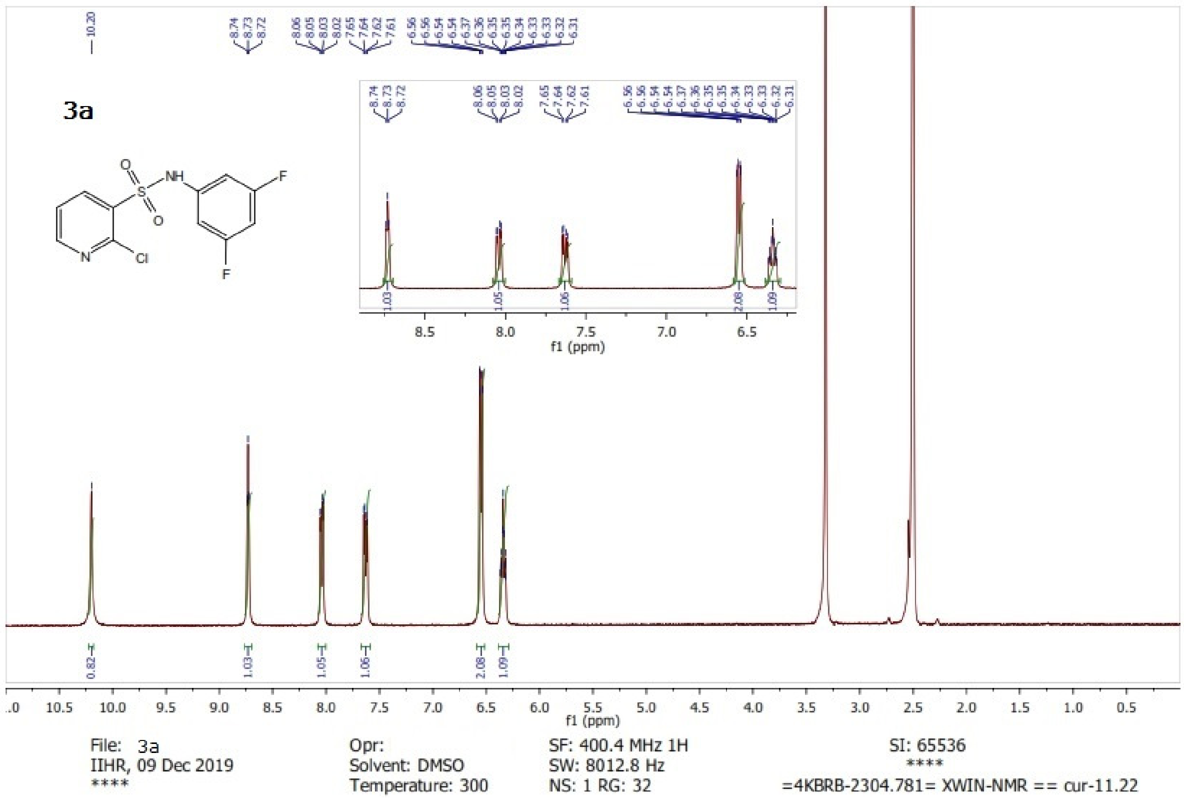

Figure S1. ^{1}H NMR spectrum (400 MHz, DMSO-d6) of 2-chloro-N-(3,5-difluorophenyl) pyridine-3-sulfonamide **3a**.

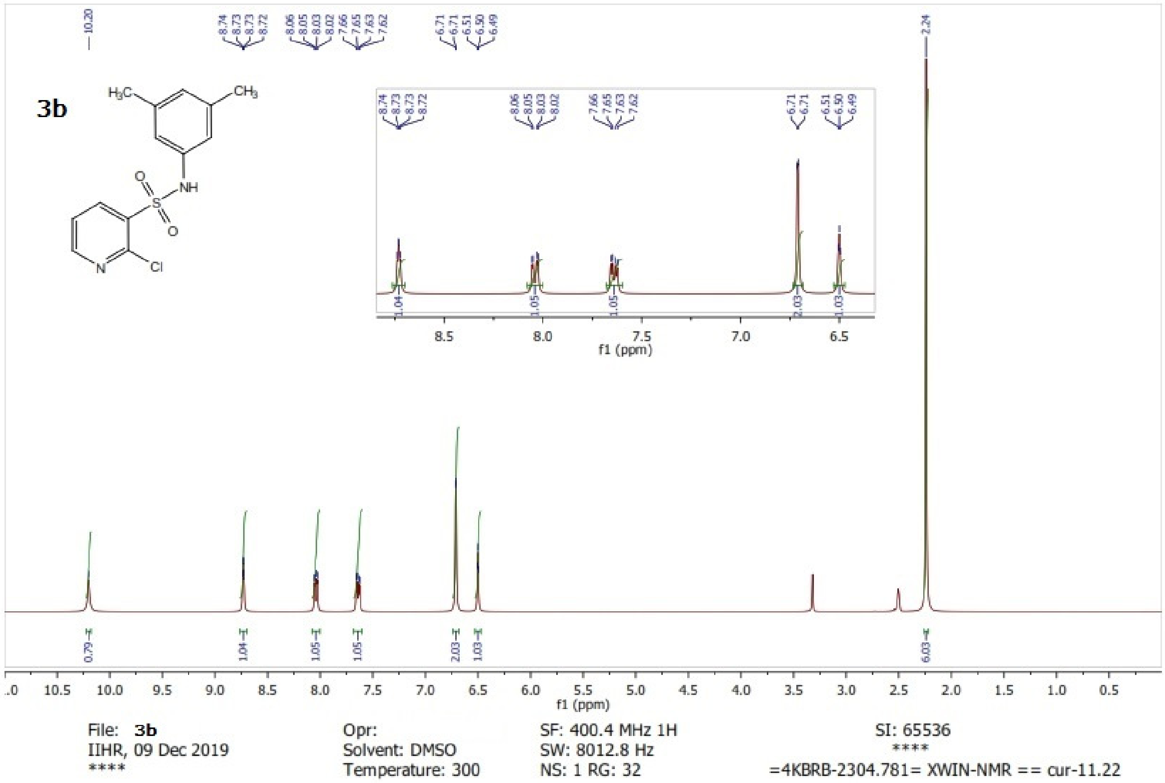

Figure S2. ^{1}H NMR spectrum (400 MHz, DMSO-d6) of 2-chloro-N-(3,5-dimethylphenyl) pyridine-3-sulfonamide **3b**.

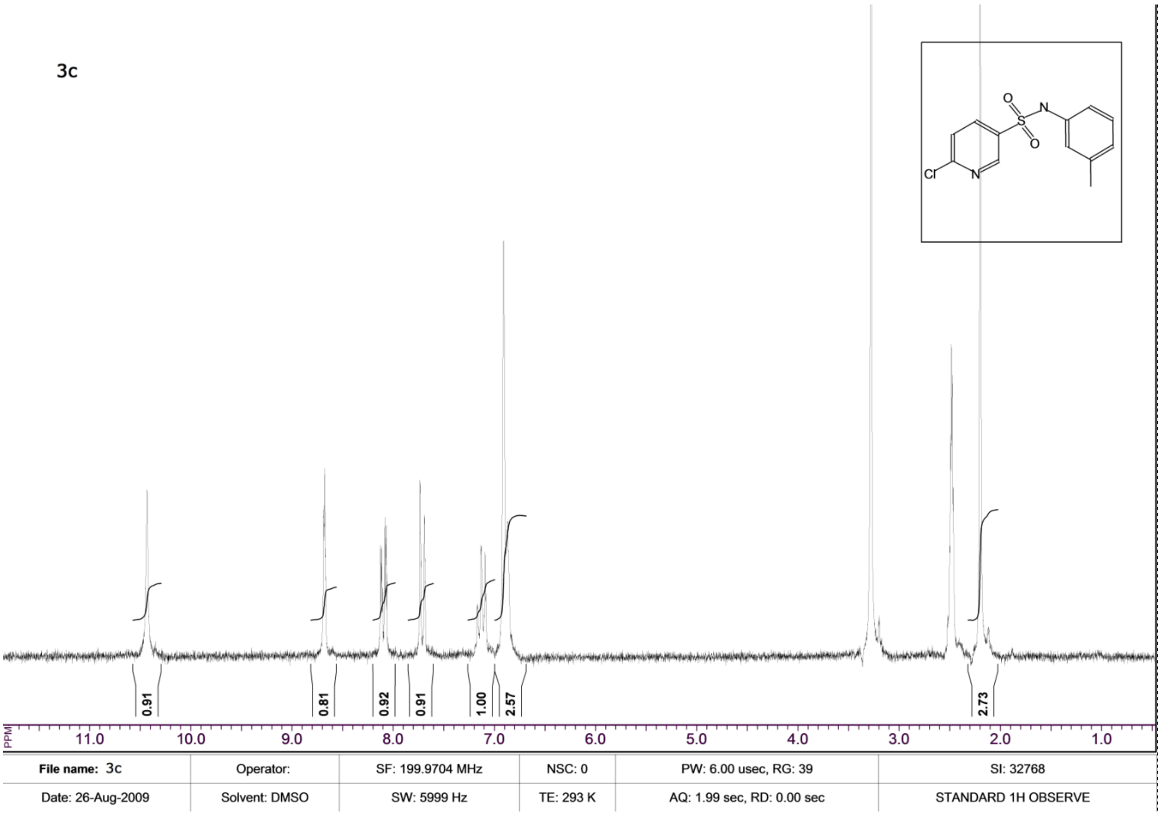

Figure S3. ^{1}H NMR spectrum (200 MHz, DMSO-d6) of 2-chloro-N-(3-methylphenyl) pyridine-5-sulfonamide **3c**.

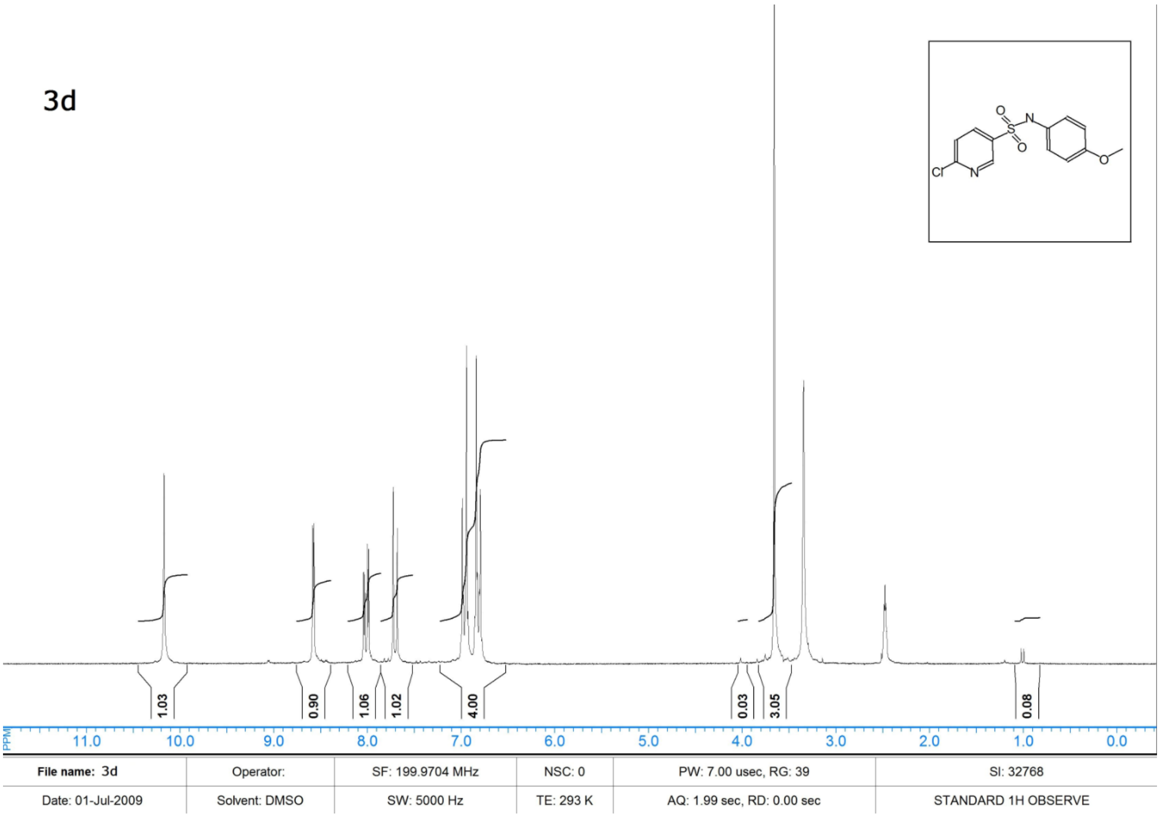

Figure S4. ^{1}H NMR spectrum (200 MHz, DMSO-d6) of 2-Chloro-N-(4-methoxyphenyl) pyridine-5-sulfonamide **3d**.

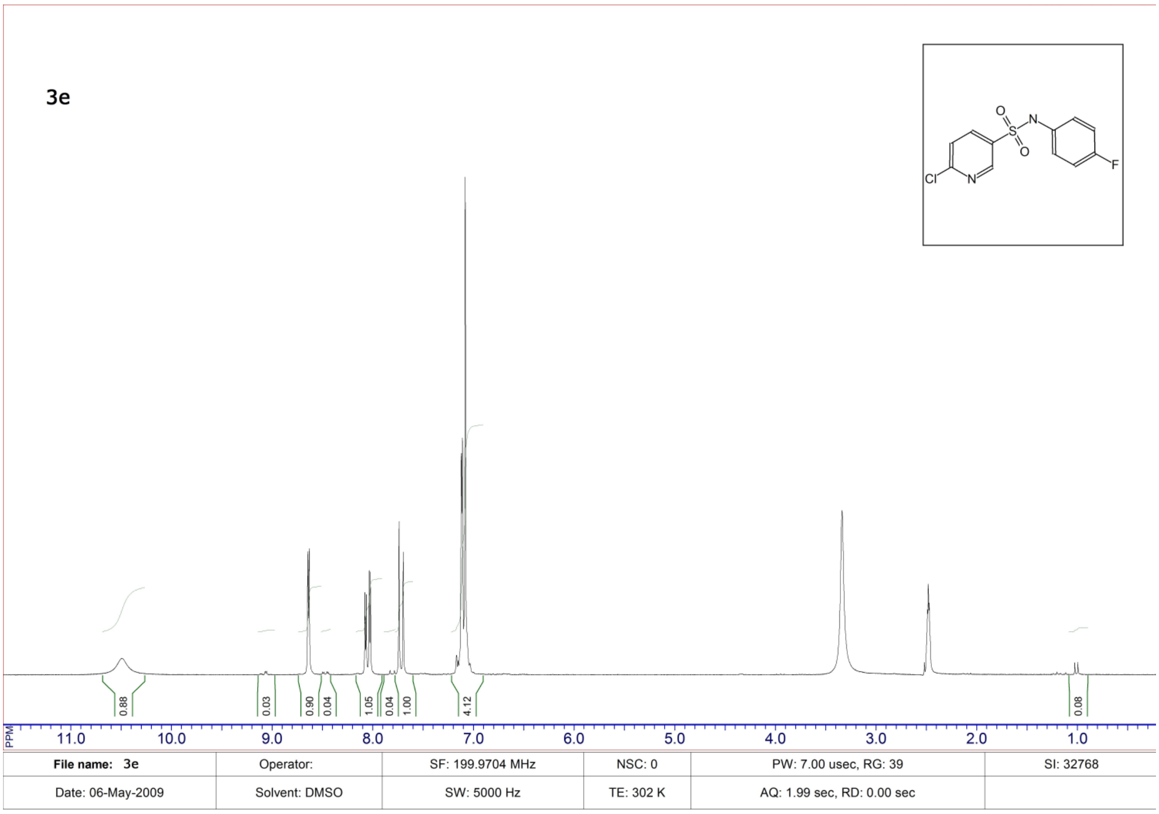

Figure S5. ^{1}H NMR spectrum (200 MHz, DMSO-d6) of 2-Chloro-N-(4-fluorophenyl) pyridine-5-sulfonamide **3e**.

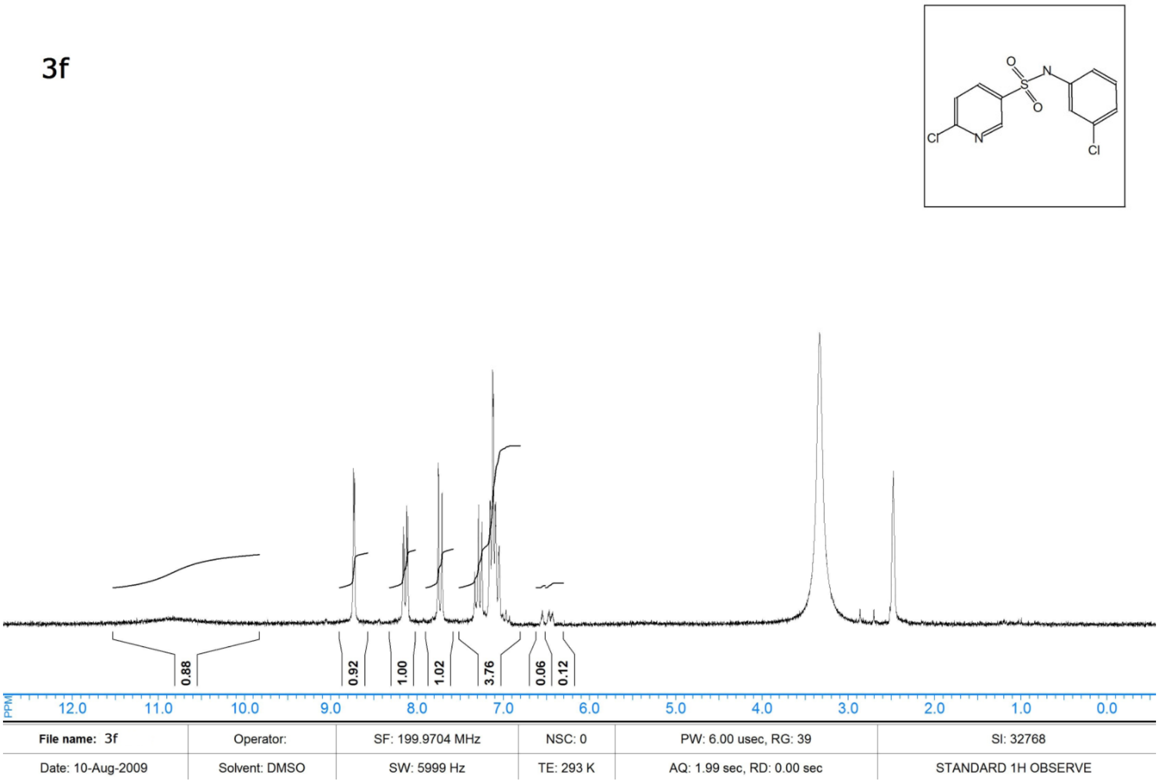

Figure S6. ^{1}H NMR spectrum (200 MHz, DMSO-d6) of 2-Chloro-N-(3-chlorophenyl) pyridine-5-sulfonamide **3f.**

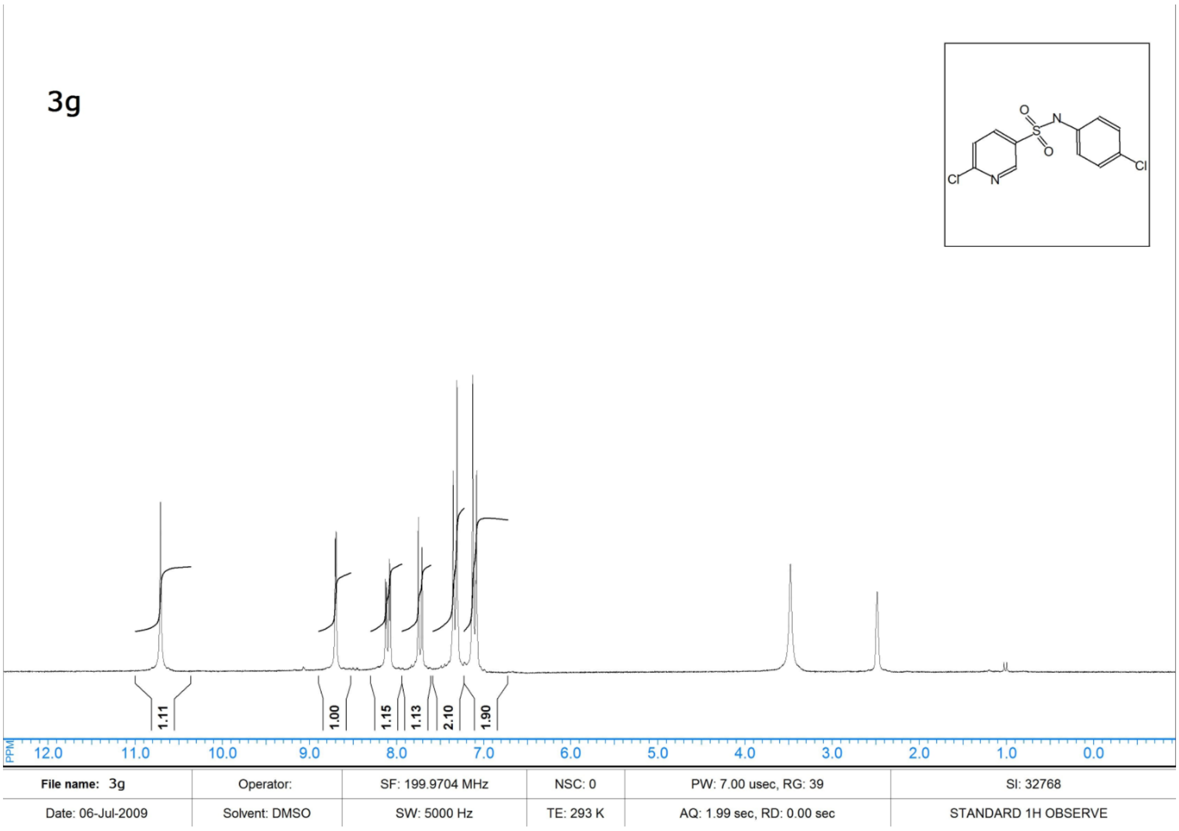

Figure S7. ¹H NMR spectrum (200 MHz, DMSO-d6) of 2-Chloro-N-(4-chlorophenyl) pyridine-5-sulfonamide **3g**.

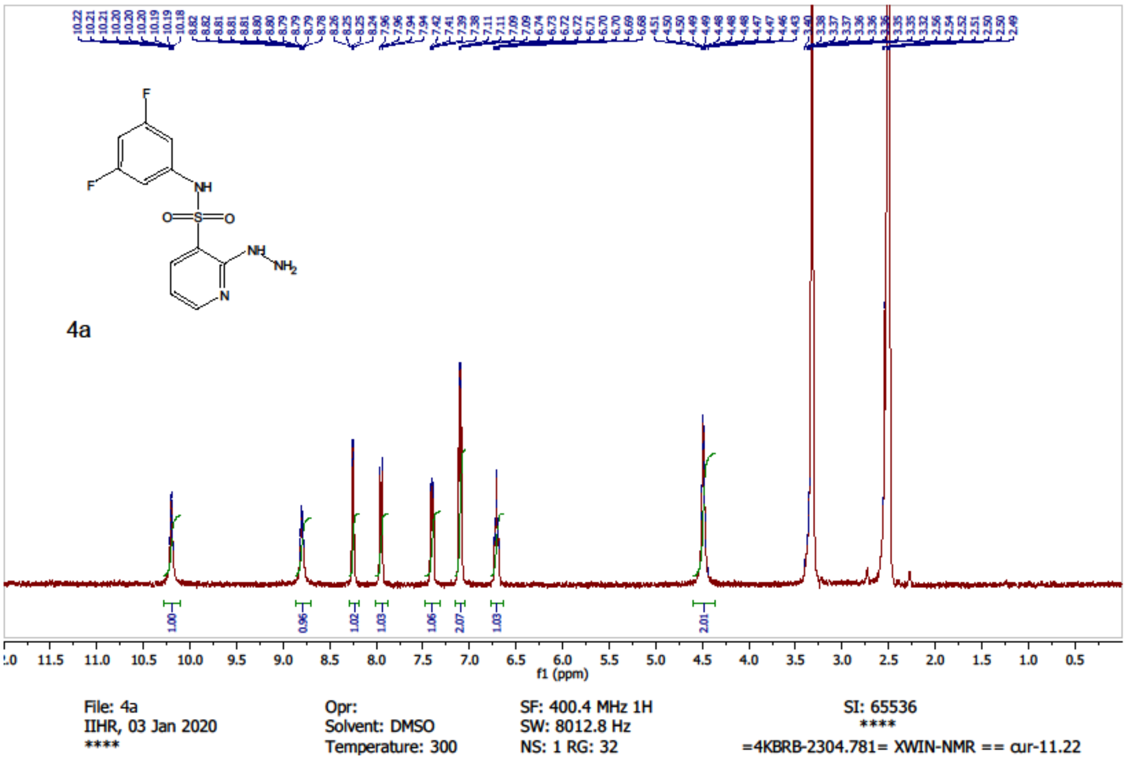

Figure S8. ¹H NMR spectrum (400 MHz, DMSO-d6) of N-(3,5-difluorophenyl)-2-hydrazinylpyridine-3-sulfonamide **4a**.

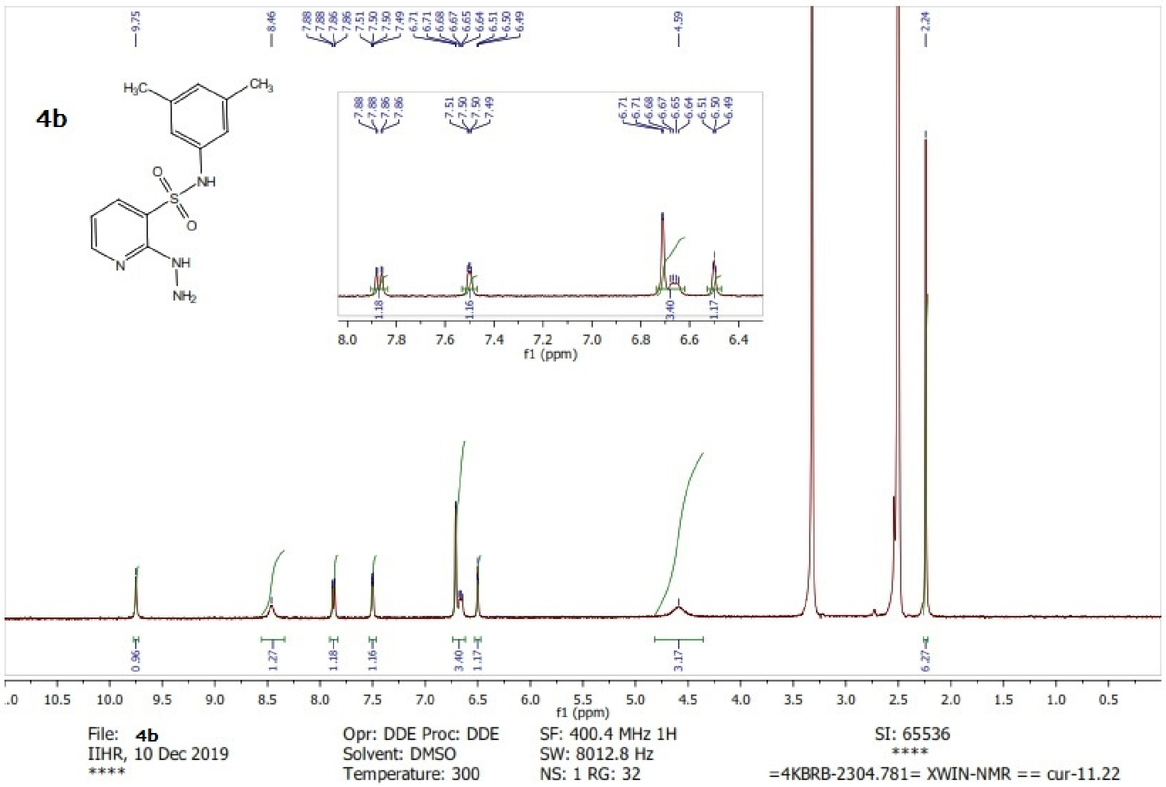

Figure S9. ^{1}H NMR spectrum (400 MHz, DMSO-d6) of N-(3,5-dimethylphenyl)-2-hydrazinylpyridine-3-sulfonamide **4b**.

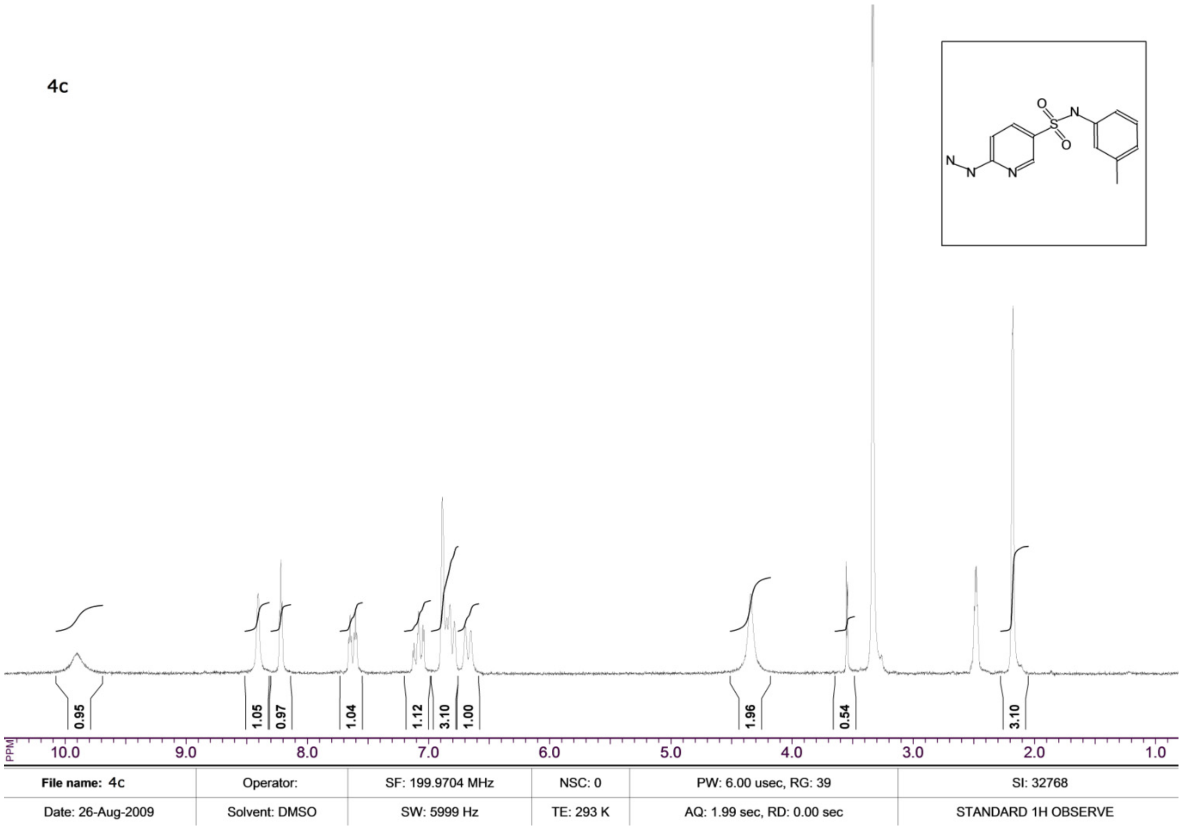

Figure S10. ^{1}H NMR spectrum (200 MHz, DMSO-d6) of 2-hydrazinyl-N-(3-methylphenyl) pyridine-5-sulfonamide **4c**.

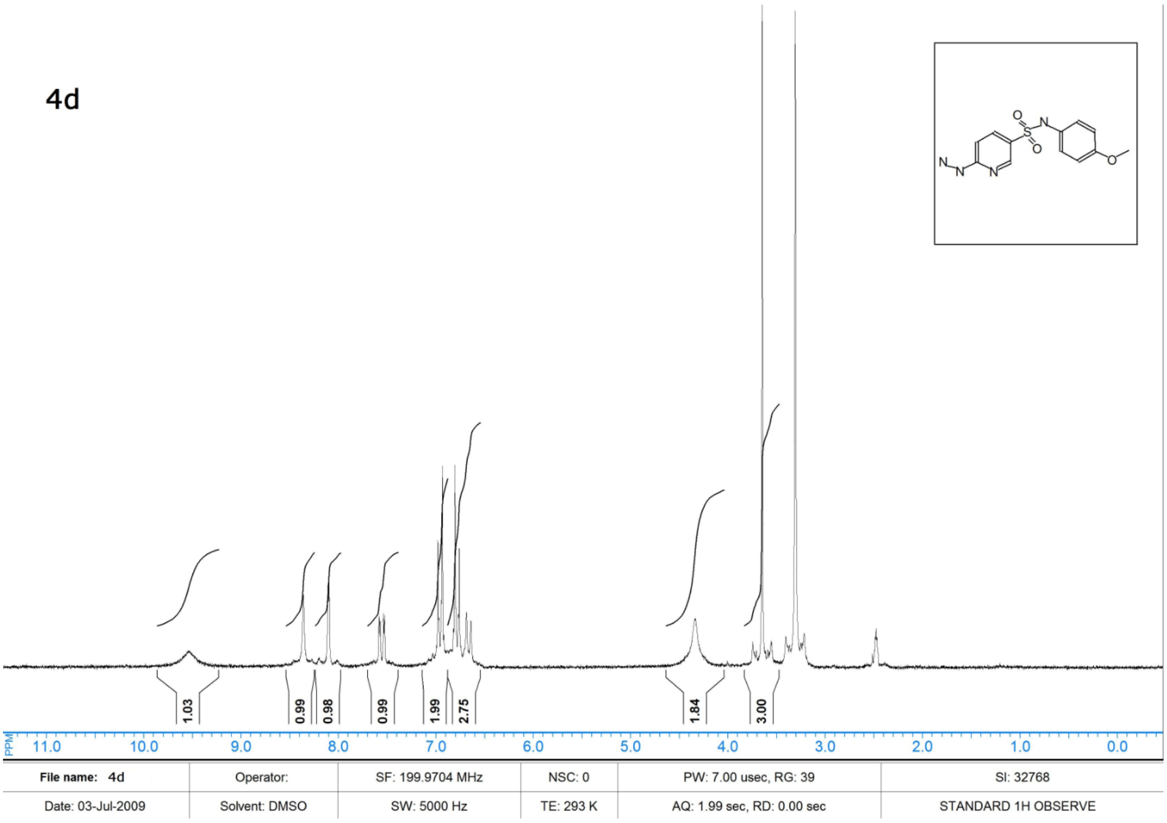

Figure S11. ^{1}H NMR spectrum (200 MHz, DMSO-d6) of 2-hydrazinyl-N-(4-methoxyphenyl) pyridine-5-sulfonamide **4d**.

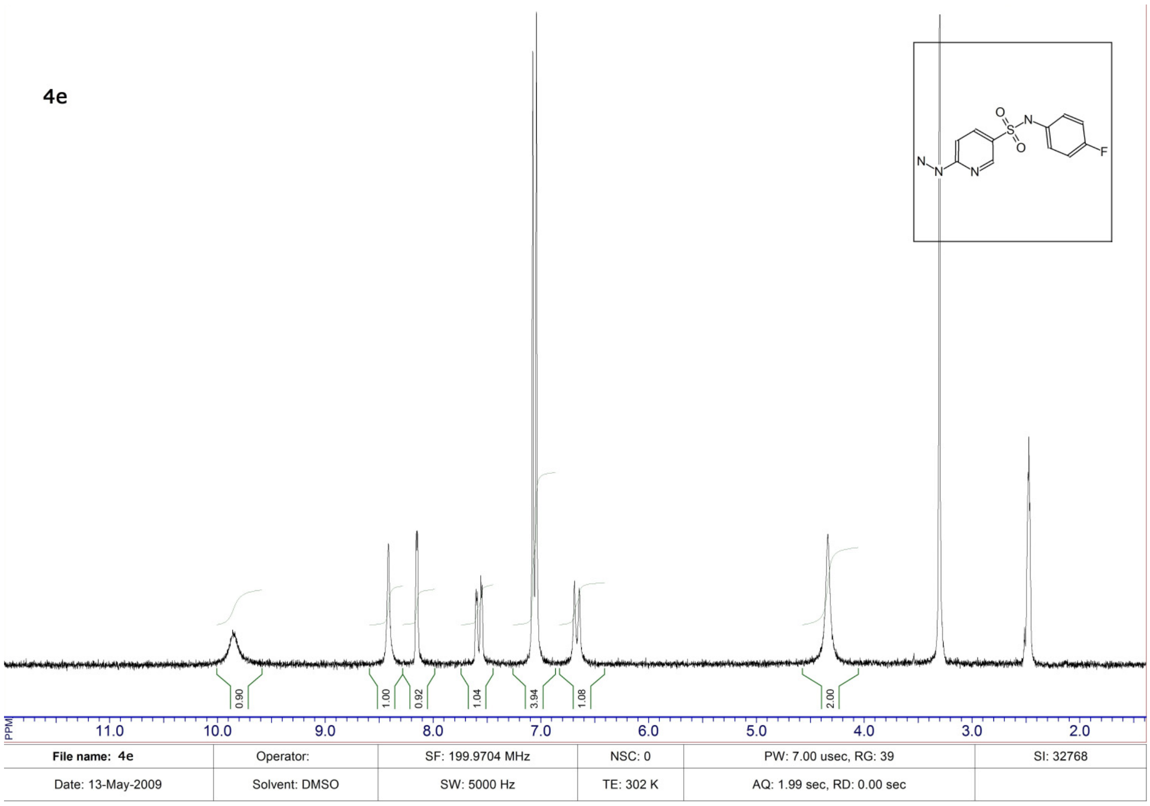

Figure S12. ^{1}H NMR spectrum (200 MHz, DMSO-d6) of N-(4-fluorophenyl)-2-hydrazinylpyridine-5-sulfonamide **4e**.

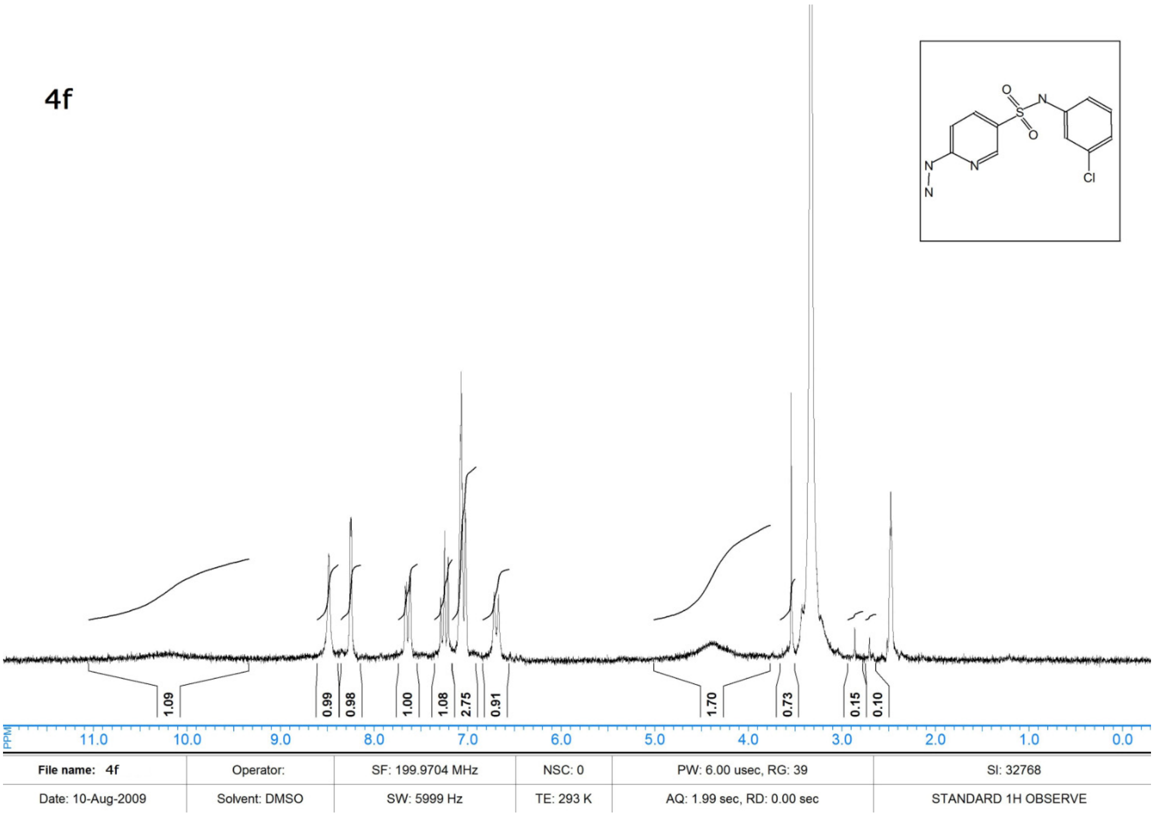

Figure S13. ¹H NMR spectrum (200 MHz, DMSO-d6) of N-(3-chlorophenyl)-2-hydrazinylpyridine-5-sulfonamide **4f**.

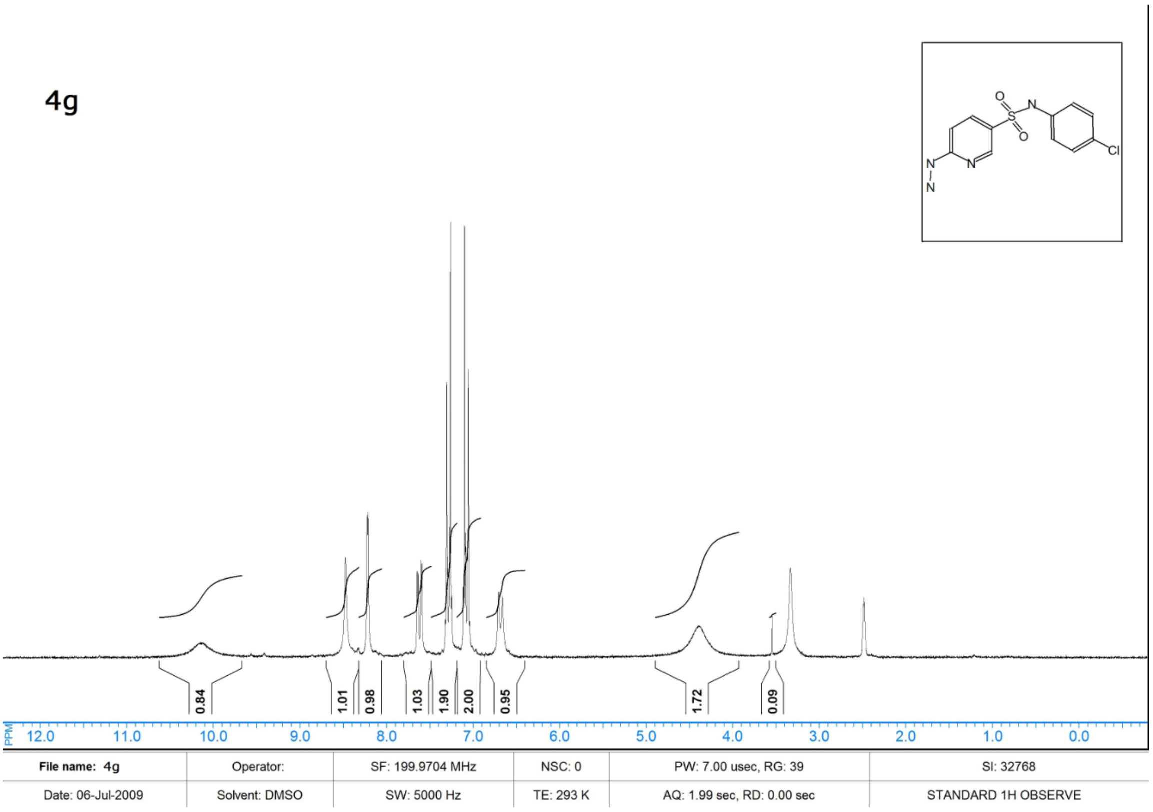

Figure S14. ¹H NMR spectrum (200 MHz, DMSO-d6) of N-(4-chlorophenyl)-2-hydrazinylpyridine-5-sulfonamide **4g**.

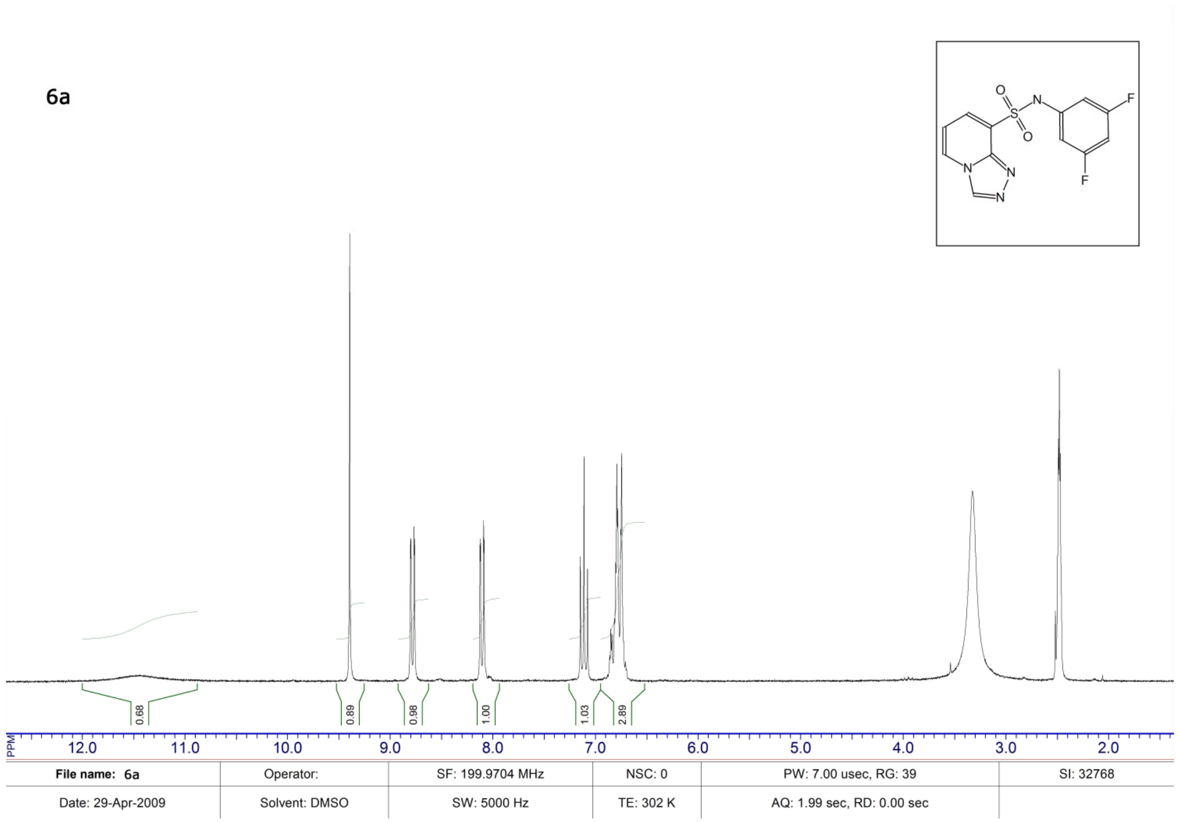

Figure S15. ^{1}H NMR spectrum (200 MHz, DMSO-d6) of N-(3,5-difluorophenyl)-[1,2,4]triazolo[4,3-a]pyridine-8-sulfonamide **6a**.

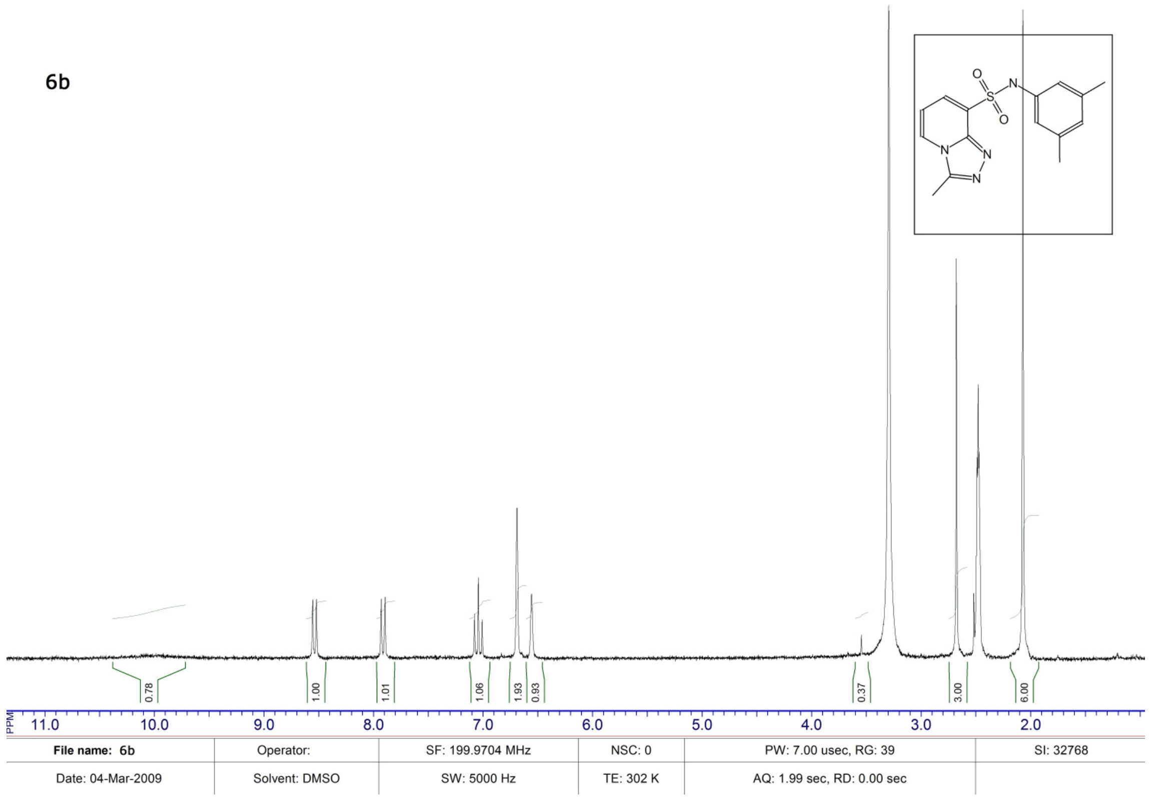

Figure S16. ^{1}H NMR spectrum (200 MHz, DMSO-d6) of N-(3,5-dimethylphenyl)-3-methyl-[1,2,4]triazolo[4,3-a]pyridine-8-sulfonamide **6b**.

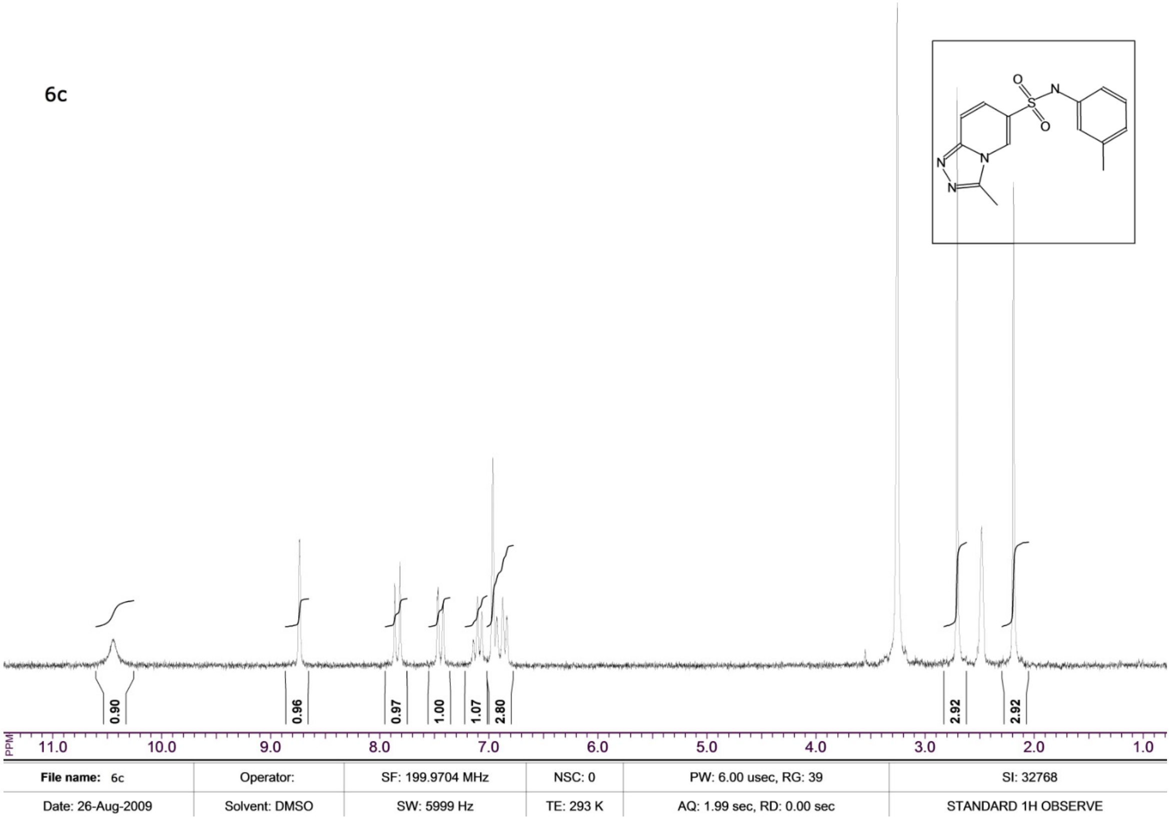

Figure S17. ^{1}H NMR spectrum (200 MHz, DMSO-d6) of 3-methyl-N-(3-methyphenyl)-[1,2,4]triazolo[4,3-a]pyridine-6-sulfonamide **6c**.

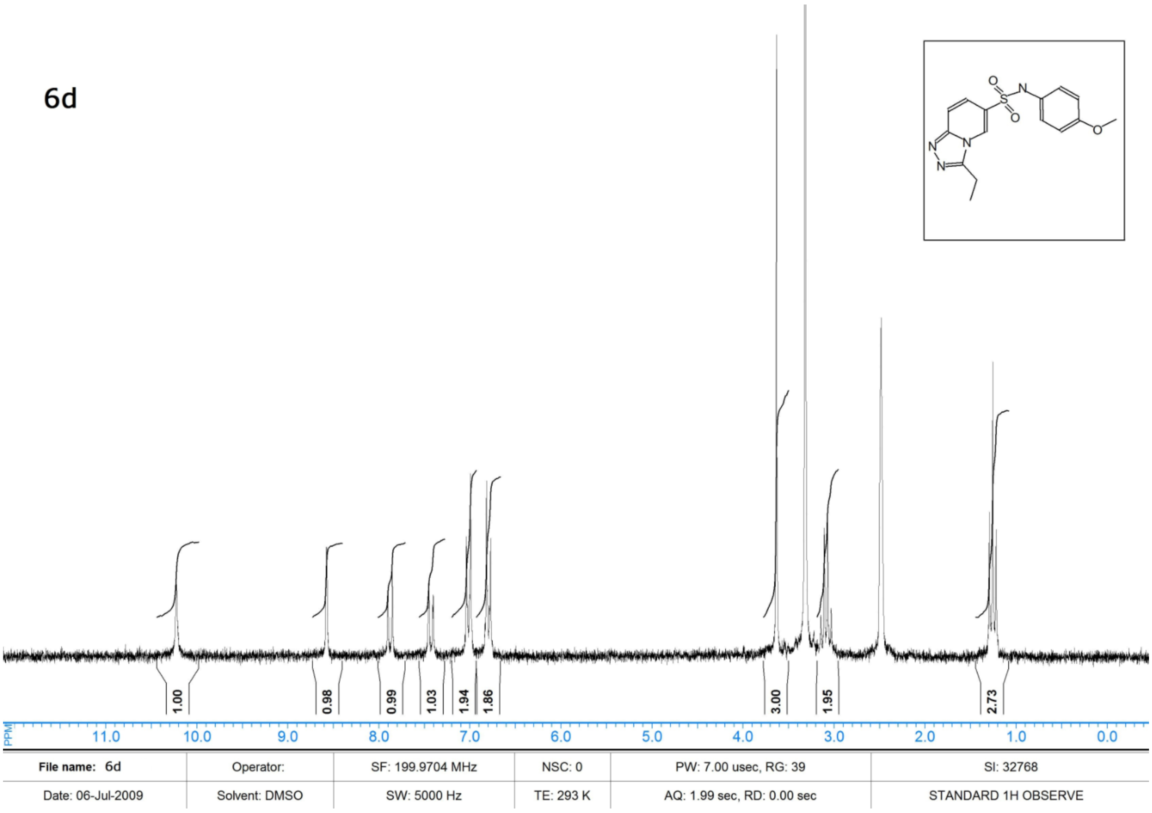

Figure S18. ^{1}H NMR spectrum (200 MHz, DMSO-d6) of 3-ethyl-N-(4-methoxyphenyl)-[1,2,4]triazolo[4,3-a]pyridine-6-sulfonamide **6d**.

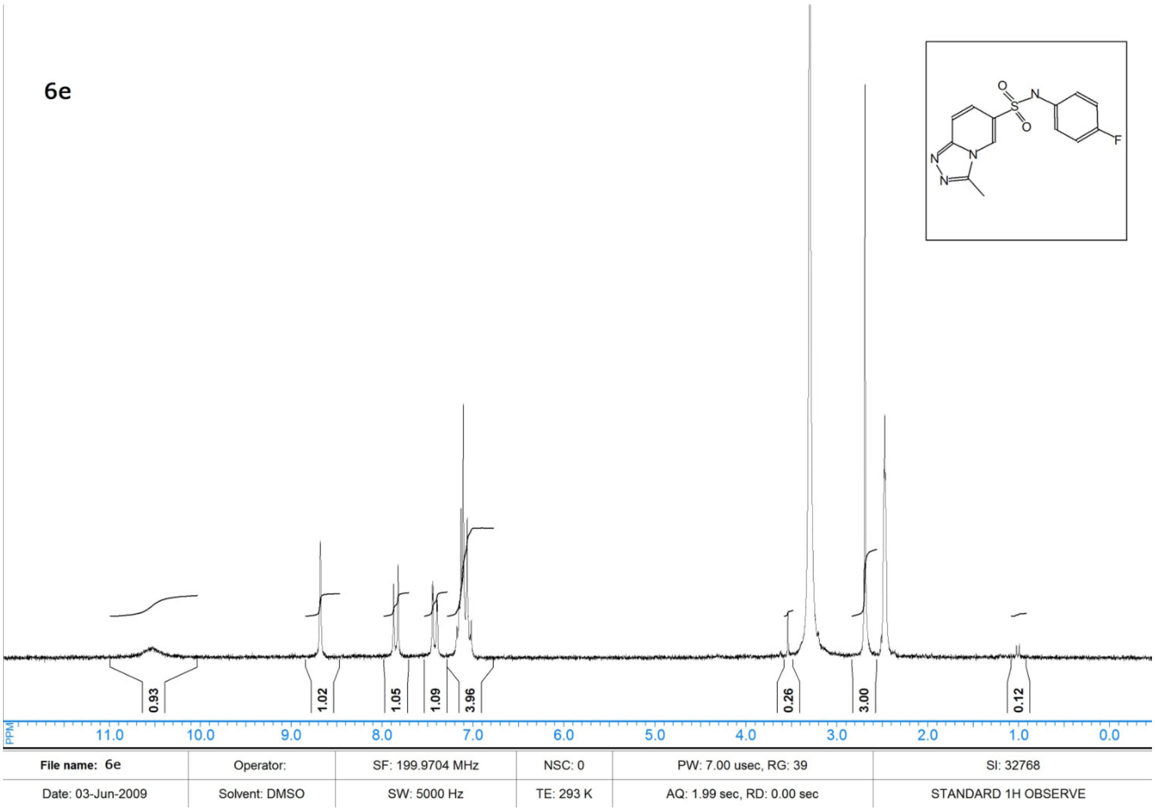

Figure S19. ^{1}H NMR spectrum (200 MHz, DMSO-d6) of N-(4-fluorophenyl)-3-methyl-[1,2,4]triazolo[4,3-a]pyridine-6-sulfonamide **6e**.

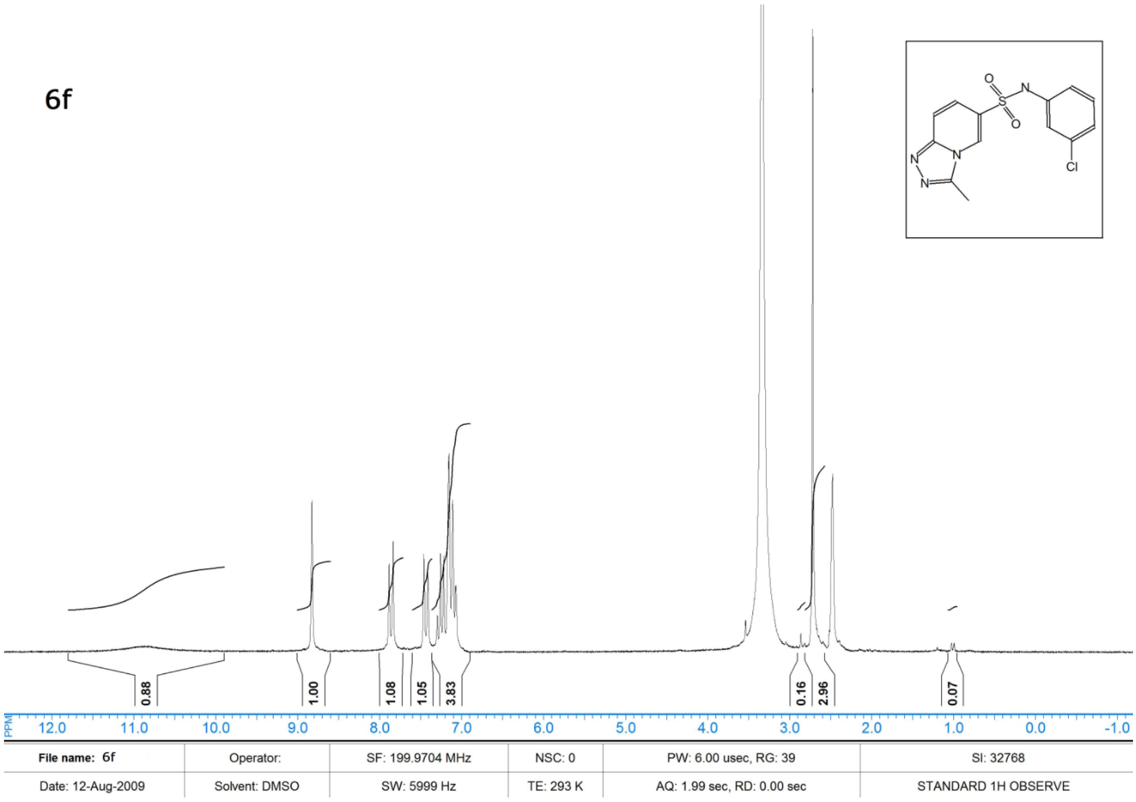

Figure S20. ^{1}H NMR spectrum (200 MHz, DMSO-d6) of N-(3-chlorophenyl)-3-methyl-[1,2,4]triazolo[4,3-a]pyridine-6-sulfonamide **6f**.

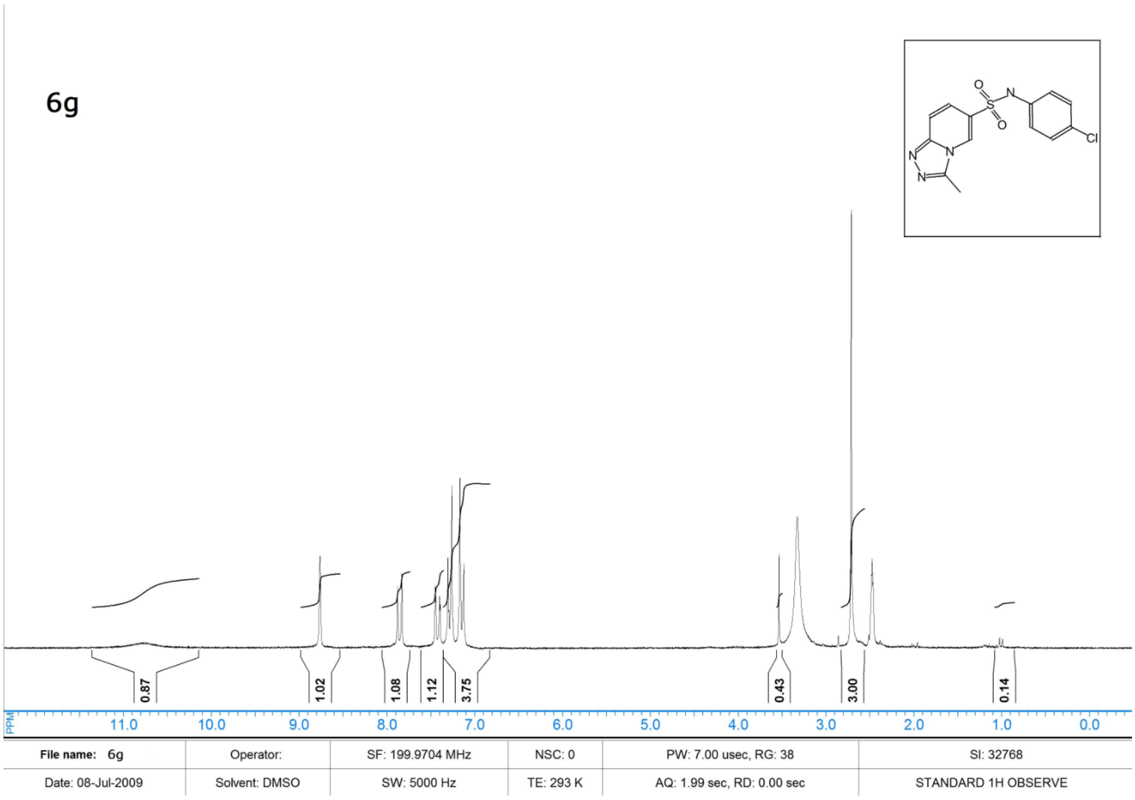

Figure S21. ^{1}H NMR spectrum (200 MHz, DMSO-d6) of N-(4-chlorophenyl)-3-methyl-[1,2,4]triazolo[4,3-a]pyridine-6-sulfonamide **6g**.

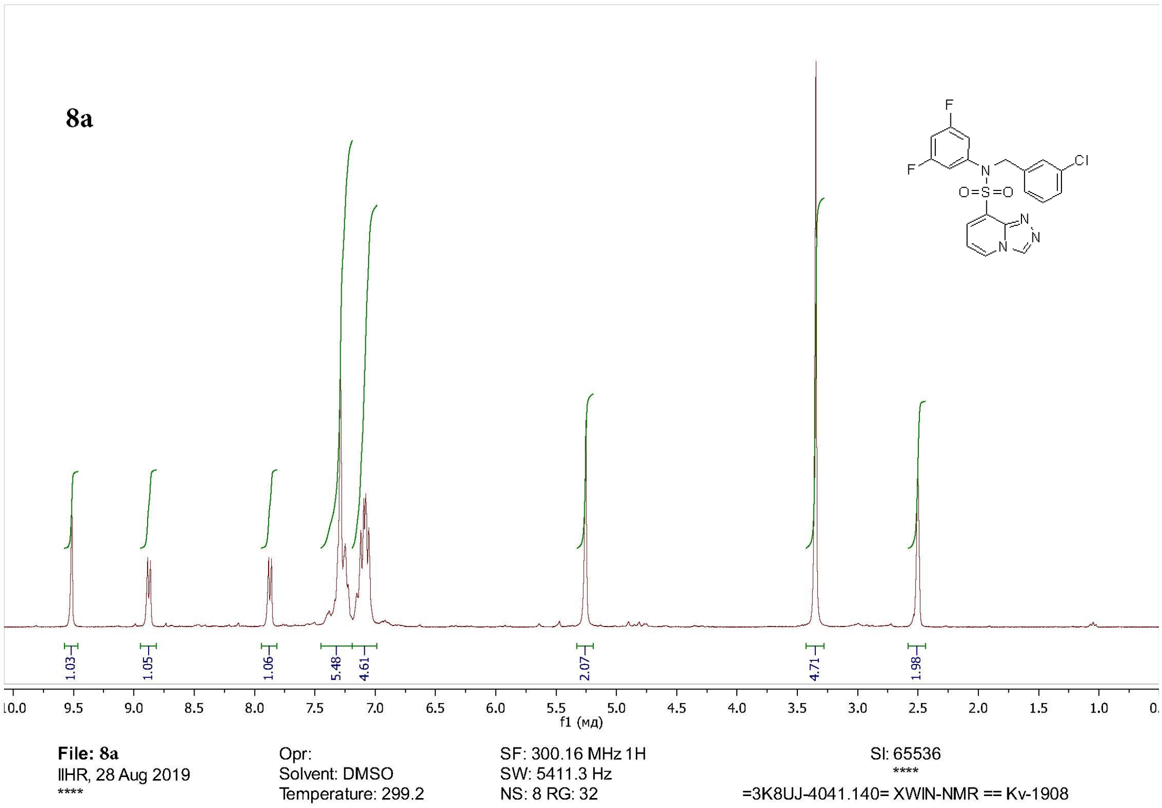

Figure S22. ^{1}H NMR spectrum (300 MHz, DMSO-d6) of N-(3-chlorobenzyl)-N-(3,5-difluorophenyl)-[1,2,4]triazolo[4,3-a]pyridine-8-sulfonamide **8a**.

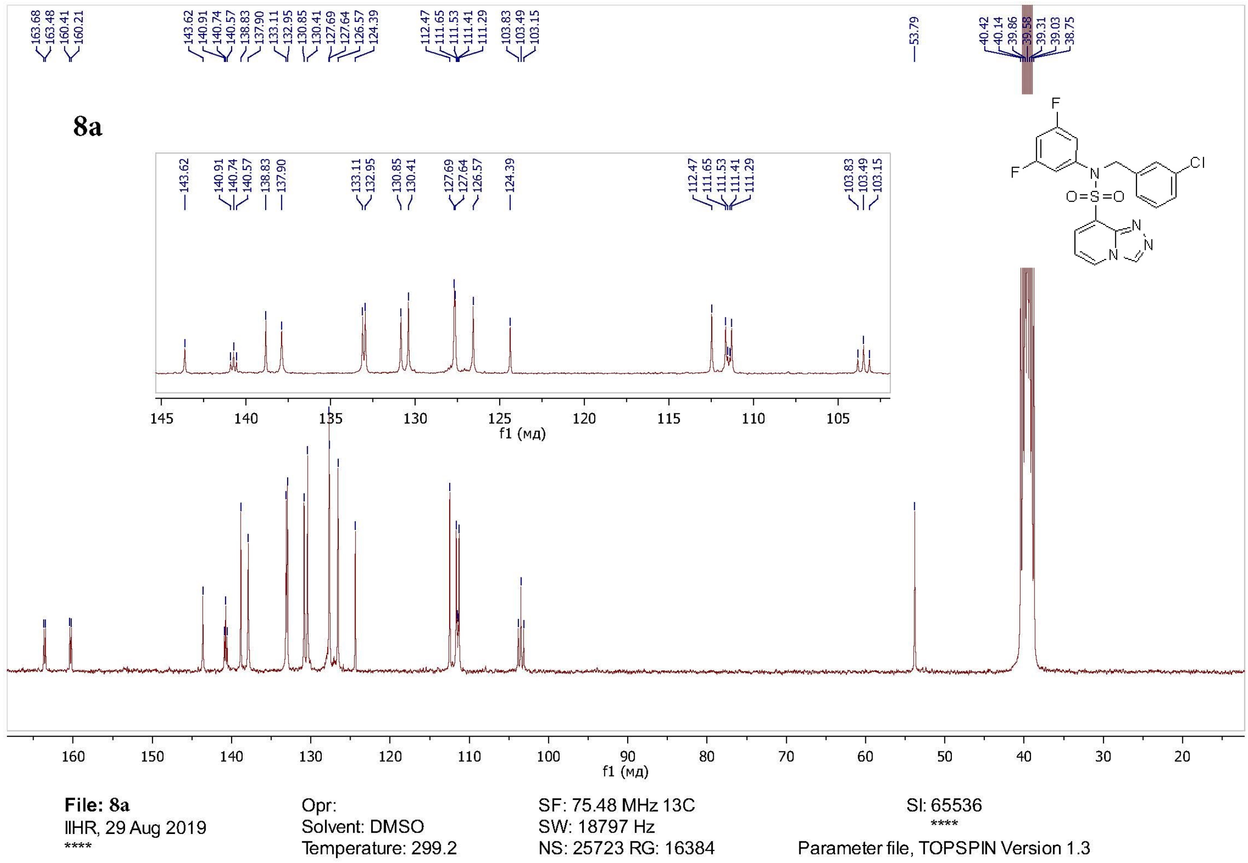

Figure S23. ^{13}C NMR spectrum (75 MHz, DMSO-d6) of N-(3-chlorobenzyl)-N-(3,5-difluorophenyl)-[1,2,4]triazolo[4,3-a]pyridine-8-sulfonamide **8a**.

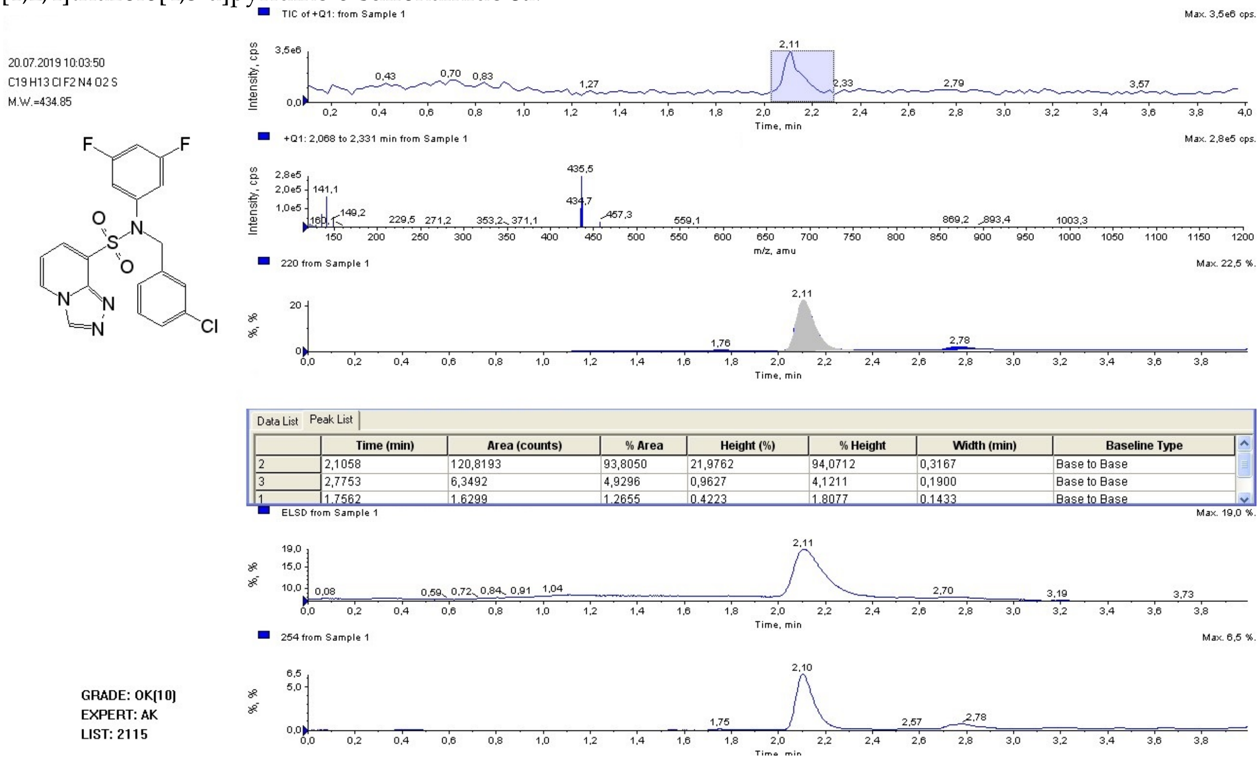

	Time (min)	Area (counts)	% Area	Height (%)	% Height	Width (min)	Baseline Type
2	2,1058	120,8193	93,8050	21,9762	94,0712	0,3167	Base to Base
3	2,7753	6,3492	4,9296	0,9627	4,1211	0,1900	Base to Base
1	1,7562	1,6299	1,2655	0,4223	1,8077	0,1433	Base to Base

Figure S24. LC/MS data for N-(3-chlorobenzyl)-N-(3,5-difluorophenyl)-[1,2,4]triazolo[4,3-a]pyridine-8-sulfonamide **8a**.

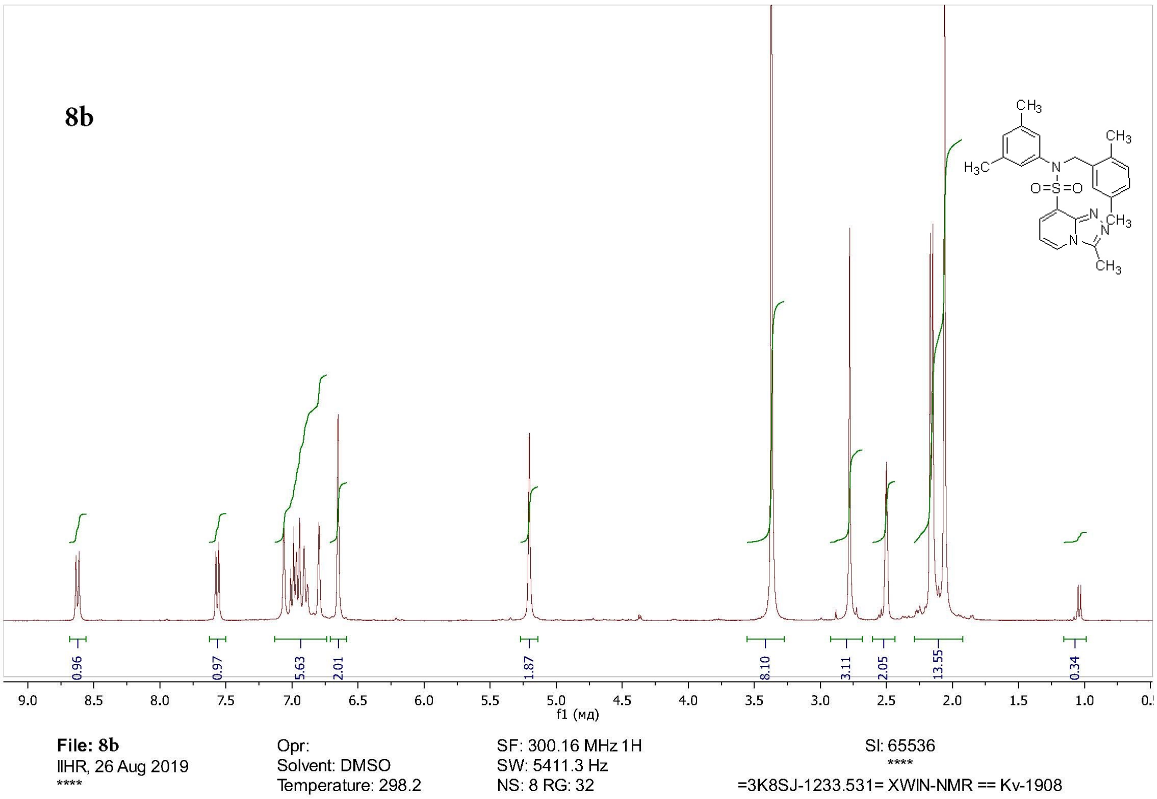

Figure S25. ^{1}H NMR spectrum (300 MHz, DMSO-d6) of N-(2,5-dimethylbenzyl)-N-(3,5-dimethylphenyl)-3-methyl-[1,2,4]triazolo[4,3-a]pyridine-8-sulfonamide **8b**.

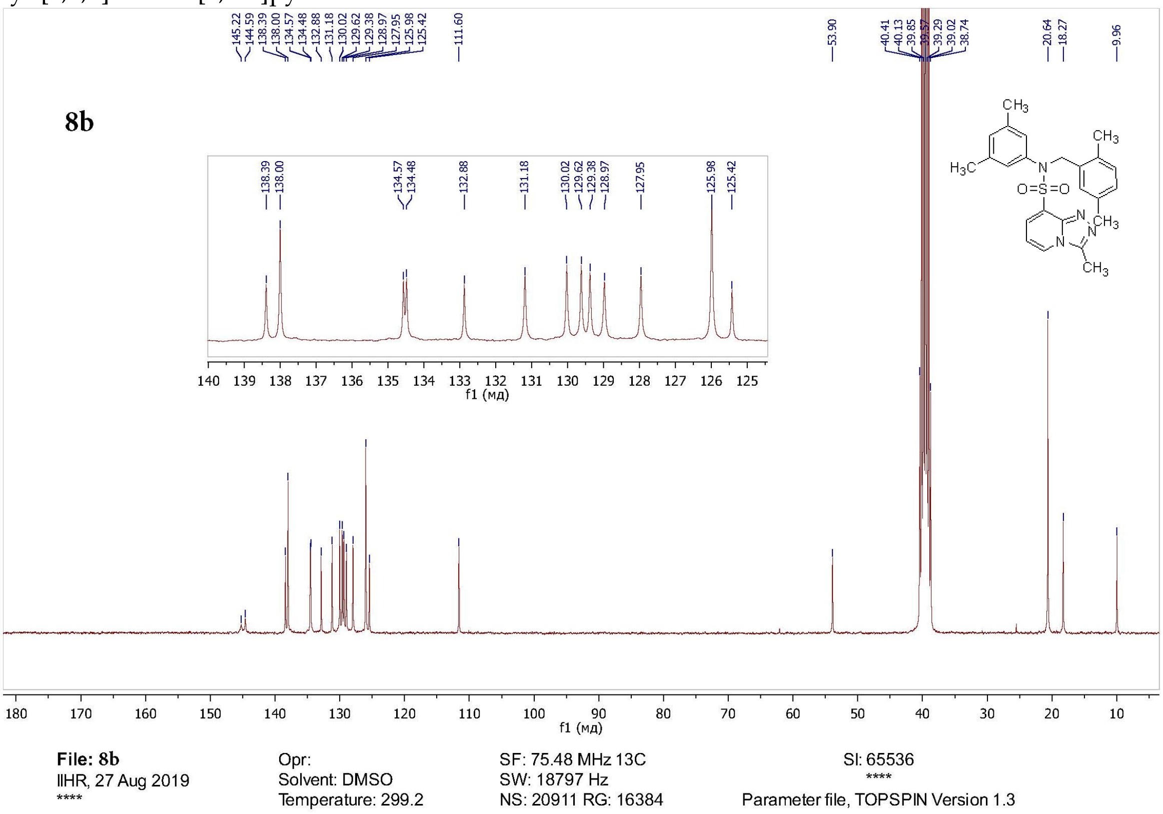

Figure S26. ^{13}C NMR spectrum (75 MHz, DMSO-d6) of N-(2,5-dimethylbenzyl)-N-(3,5-dimethylphenyl)-3-methyl-[1,2,4]triazolo[4,3-a]pyridine-8-sulfonamide **8b**.

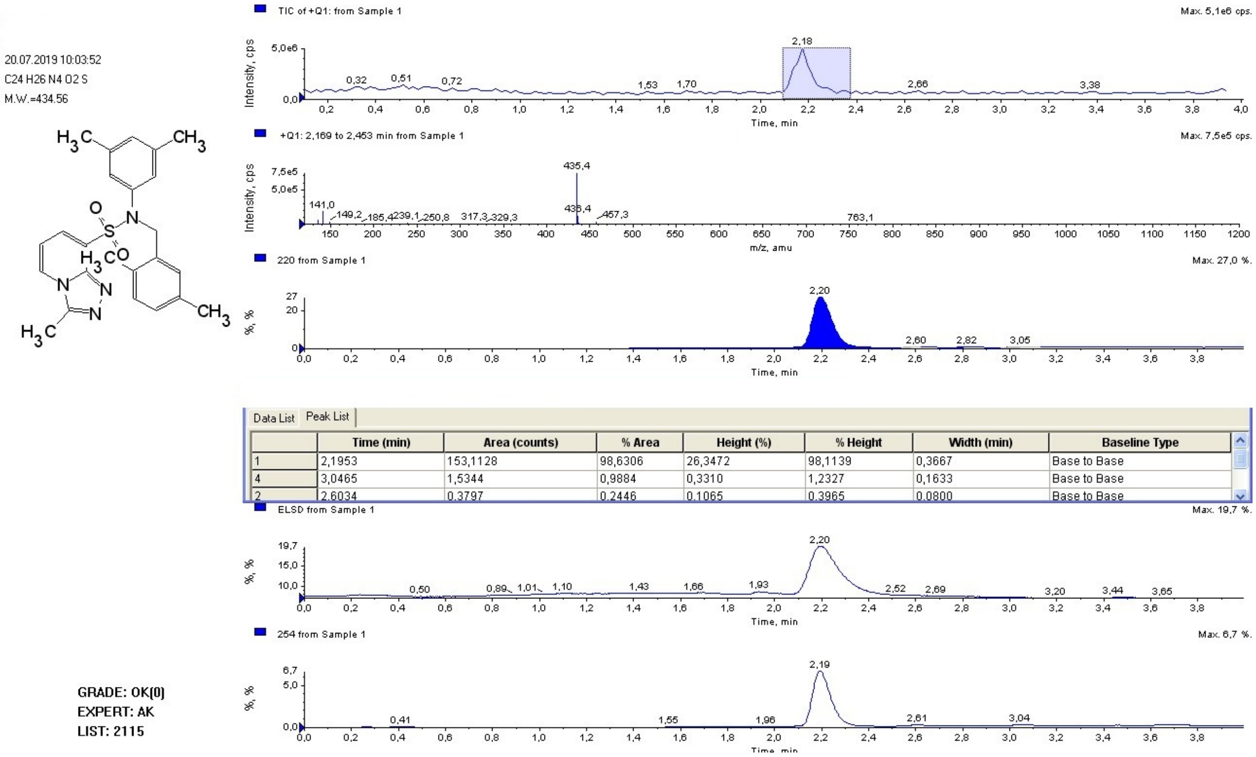

	Time (min)	Area (counts)	% Area	Height (%)	% Height	Width (min)	Baseline Type
1	2,1953	153,1128	98,6306	26,3472	98,1139	0,3667	Base to Base
4	3,0465	1,5344	0,9884	0,3310	1,2327	0,1633	Base to Base
2	2,6034	0,3797	0,2446	0,1065	0,3965	0,0800	Base to Base

Figure S27. LC/MS data for N-(2,5-dimethylbenzyl)-N-(3,5-dimethylphenyl)-3-methyl-[1,2,4]triazolo[4,3-a]pyridine-8-sulfonamide **8b**.

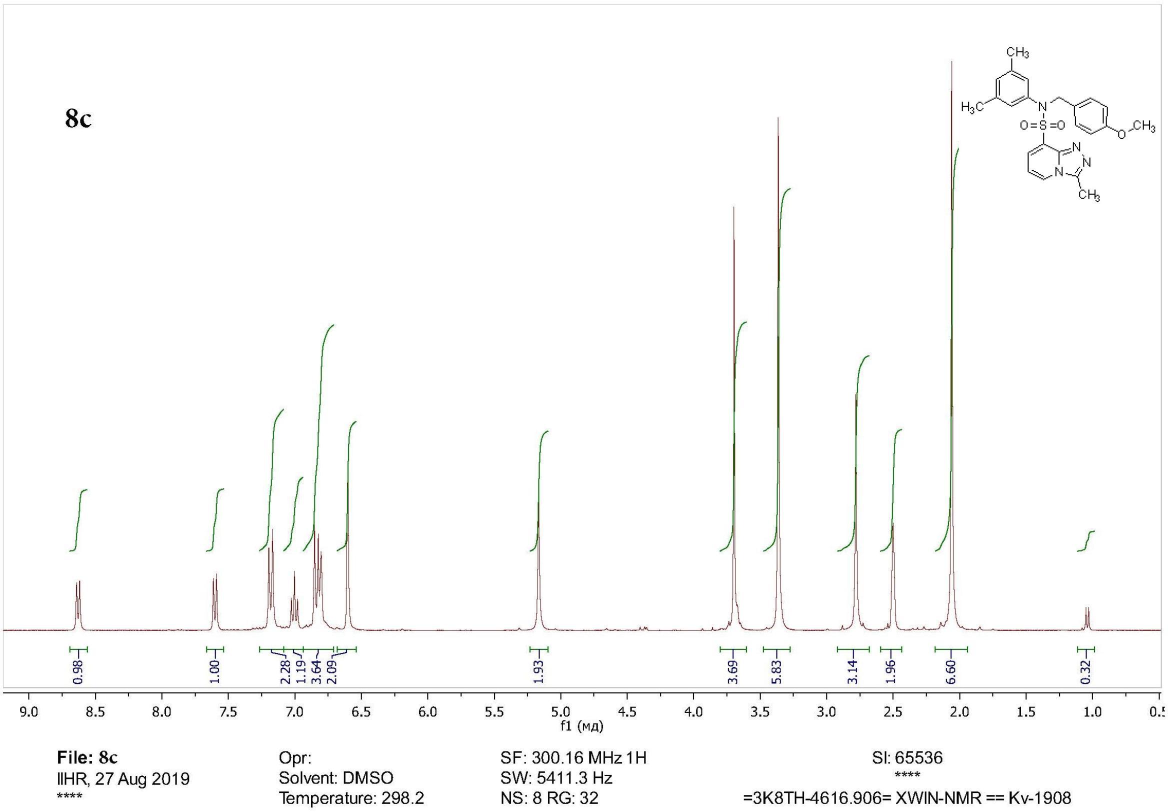

Figure S28. ^{1}H NMR spectrum (300 MHz, DMSO-d6) of N-(3,5-dimethylphenyl)-N-(4-methoxybenzyl)-3-methyl-[1,2,4]triazolo[4,3-a]pyridine-8-sulfonamide **8c**.

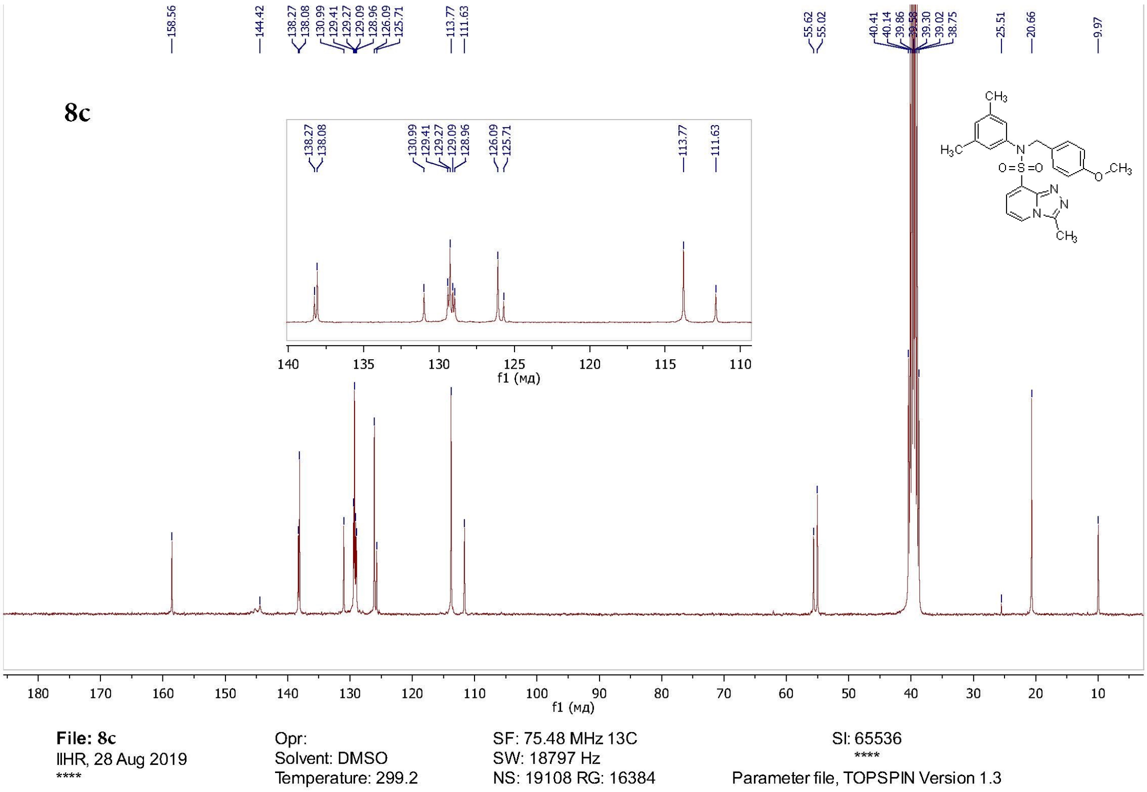

Figure S29. ^{13}C NMR spectrum (75 MHz, DMSO-d6) of N-(3,5-dimethylphenyl)-N-(4-methoxybenzyl)-3-methyl-[1,2,4]triazolo[4,3-a]pyridine-8-sulfonamide **8c**.

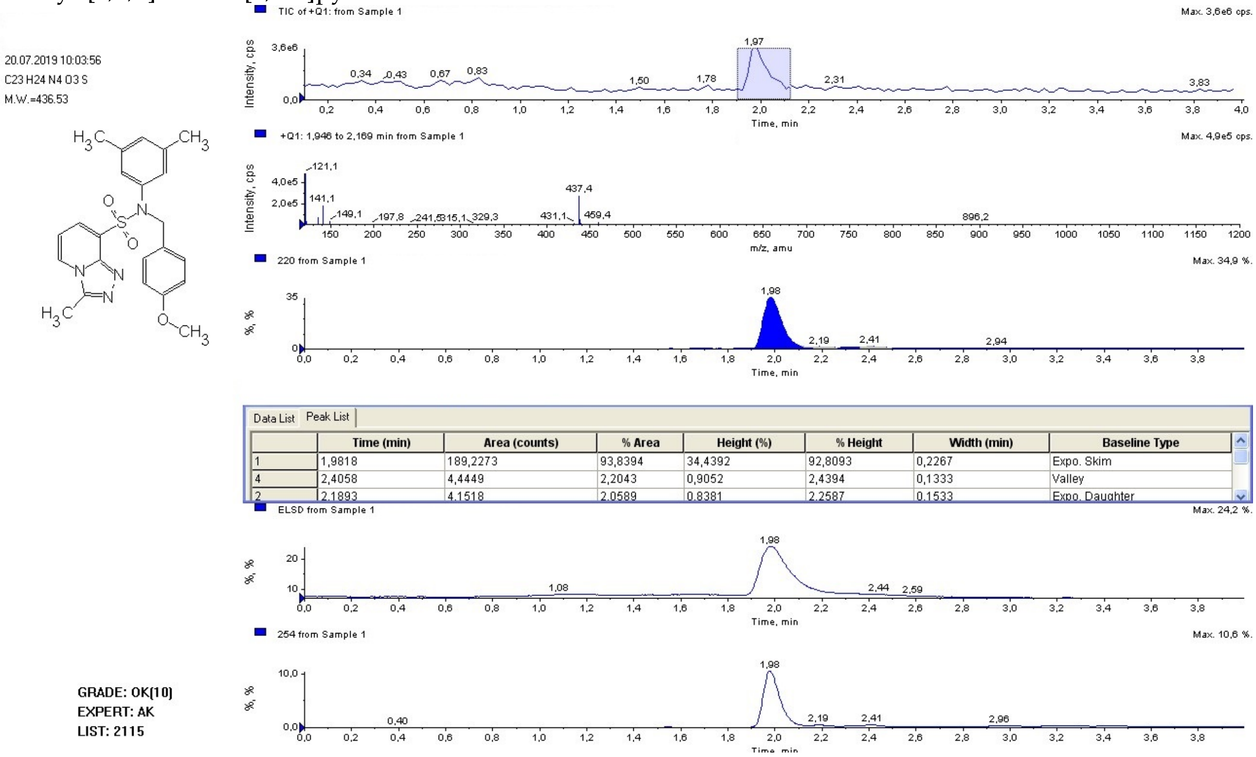

	Time (min)	Area (counts)	% Area	Height (%)	% Height	Width (min)	Baseline Type
1	1,9818	189,2273	93,8394	34,4392	92,8093	0,2267	Expo. Skim
4	2,4058	4,4449	2,2043	0,9052	2,4394	0,1333	Valley
2	2,1893	4,1518	2,0589	0,8381	2,2587	0,1533	Expo. Daughter

Figure S30. LC/MS data for N-(3,5-dimethylphenyl)-N-(4-methoxybenzyl)-3-methyl-[1,2,4]triazolo[4,3-a]pyridine-8-sulfonamide **8c**.

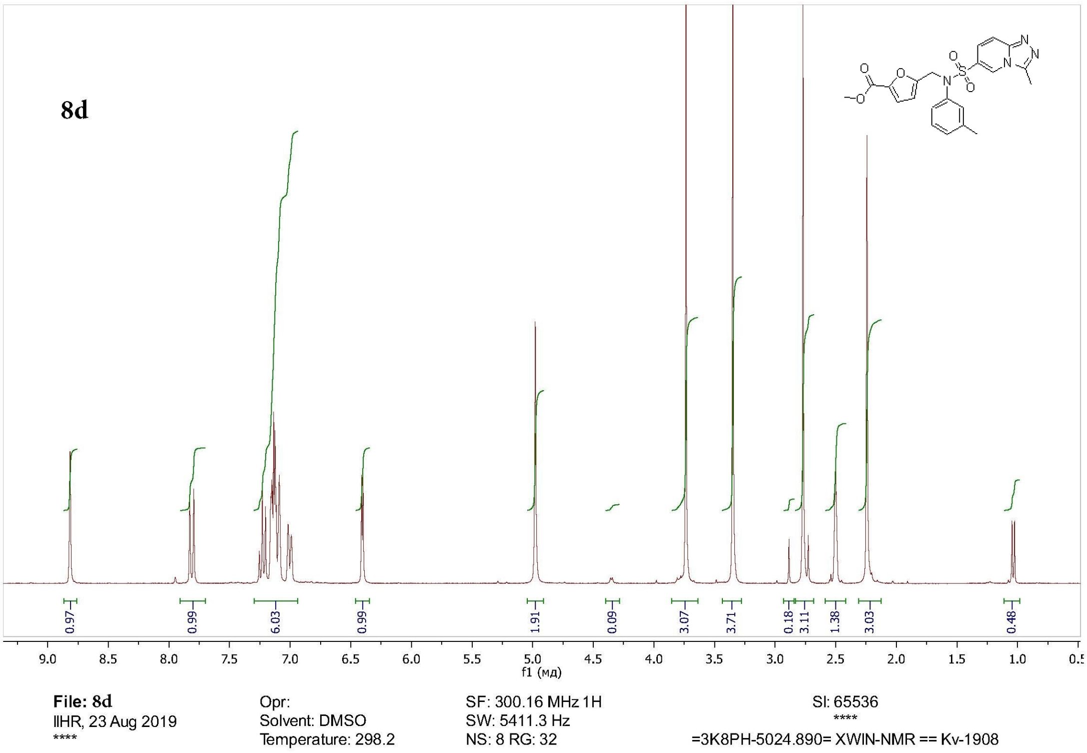

Figure S31. ^{1}H NMR spectrum (300 MHz, DMSO-d6) of Methyl 5-{[3-methyl-N-(3-methylphenyl)-[1,2,4]triazolo[4,3-a]pyridine-6-sulfonamido]methyl}furan-2-carboxylate **8d**.

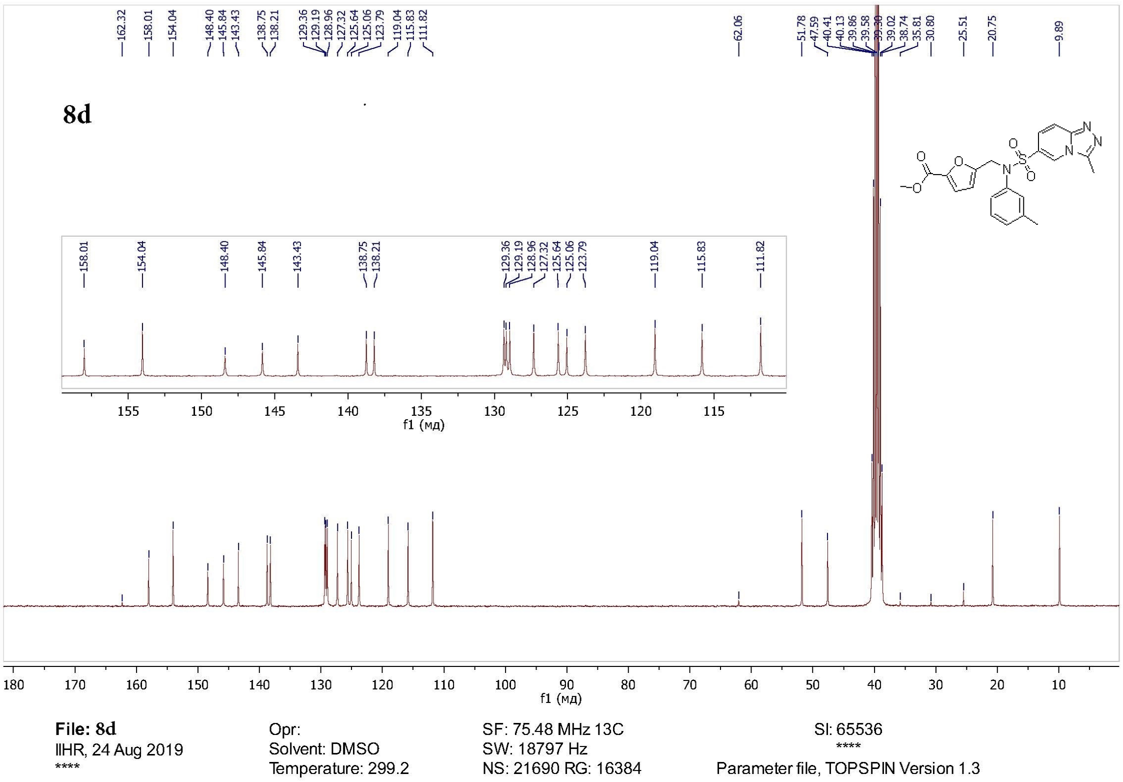

Figure S32. ^{13}C NMR spectrum (75 MHz, DMSO-d6) of Methyl 5-{[3-methyl-N-(3-methylphenyl)-[1,2,4]triazolo[4,3-a]pyridine-6-sulfonamido]methyl}furan-2-carboxylate **8d**.

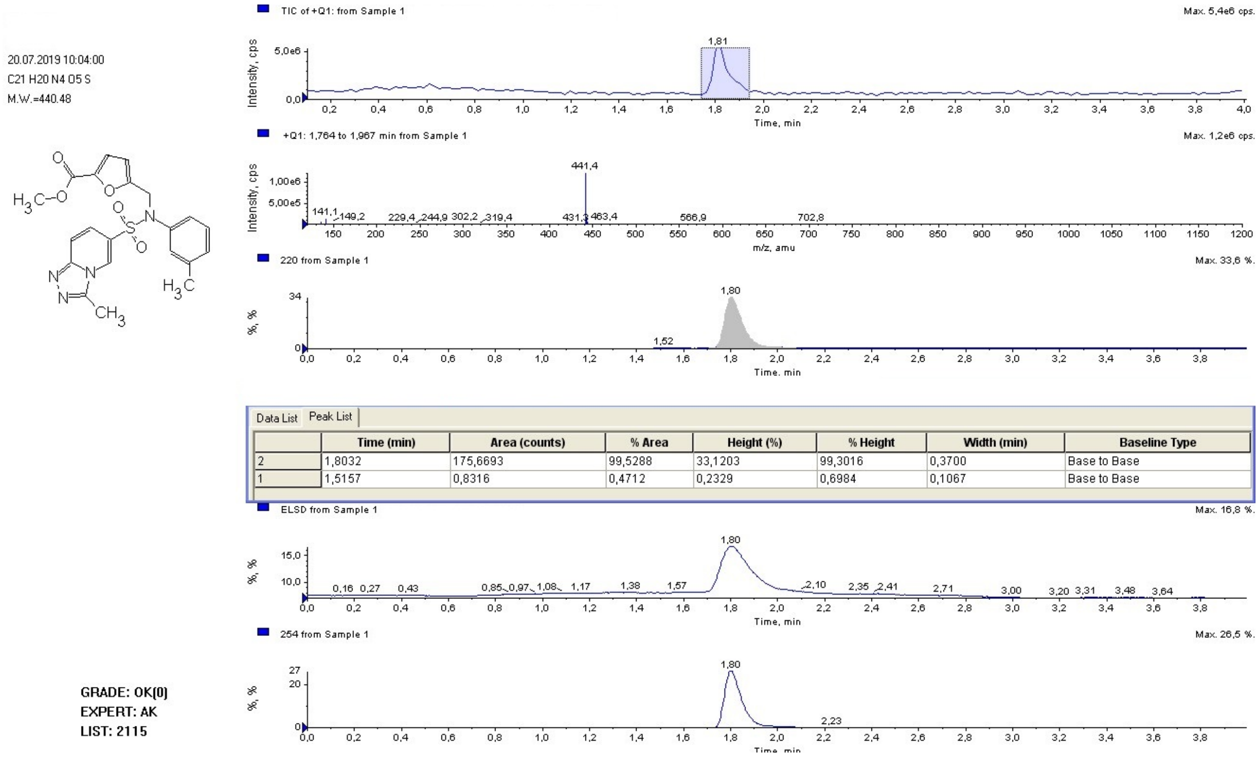

	Time (min)	Area (counts)	% Area	Height (%)	% Height	Width (min)	Baseline Type
2	1,8032	175,6693	99,5288	33,1203	99,3016	0,3700	Base to Base
1	1,5157	0,8316	0,4712	0,2329	0,6984	0,1067	Base to Base

Figure S33. LC/MS data for Methyl 5-{[3-methyl-N-(3-methylphenyl)-[1,2,4]triazolo[4,3-a]pyridine-6-sulfonamido]methyl}furan-2-carboxylate **8d**.

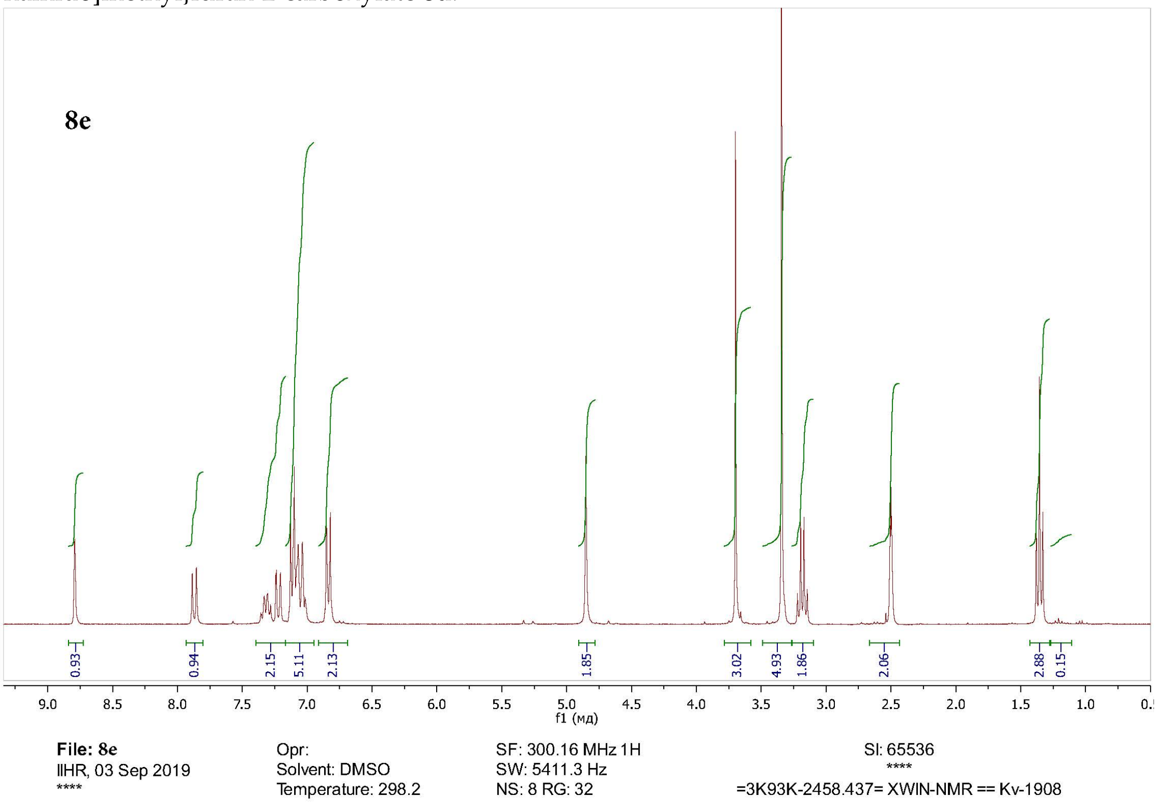

Figure S34. ^{1}H NMR spectrum (300 MHz, DMSO-d6) of 3-Ethyl-N-(3-fluorobenzyl)-N-(4-methoxyphenyl)-[1,2,4]triazolo[4,3-a]pyridine-6-sulfonamide **8e**.

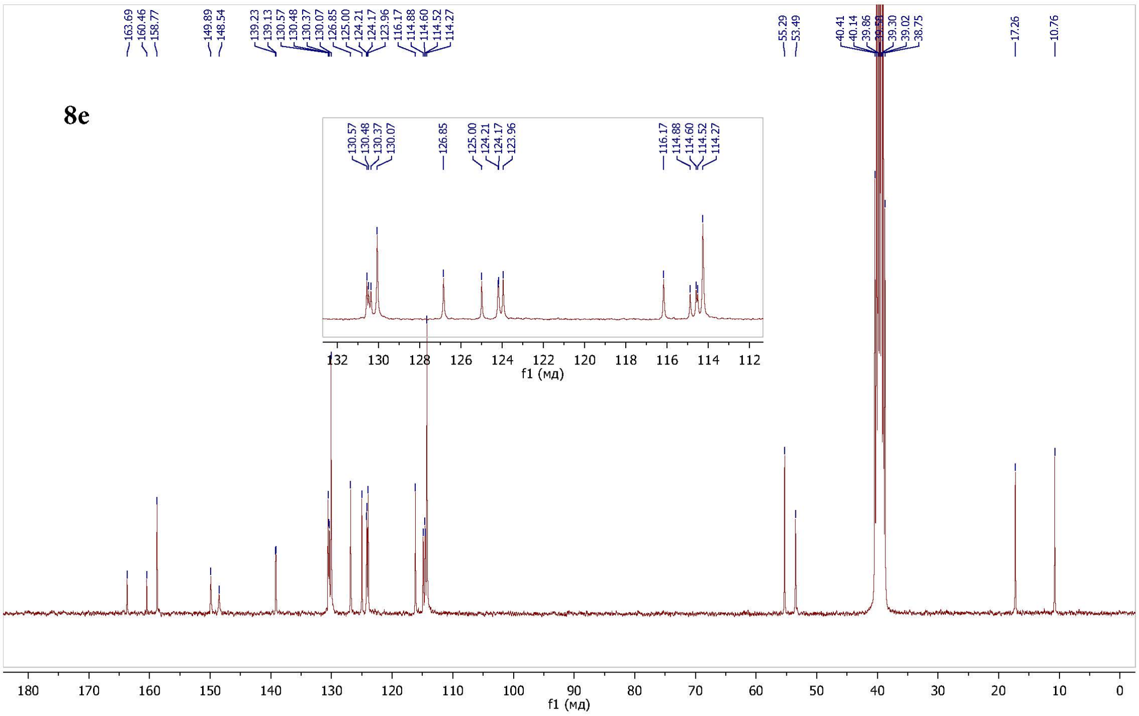

File: 8e
IIHR, 04 Sep 2019
****

Opr:
Solvent: DMSO
Temperature: 299.2

SF: 75.48 MHz 13C
SW: 18797 Hz
NS: 17695 RG: 16384

SI: 65536
****
Parameter file, TOPSPIN Version 1.3

Figure S35. ^{13}C NMR spectrum (75 MHz, DMSO-d6) of 3-Ethyl-N-(3-fluorobenzyl)-N-(4-methoxyphenyl)-[1,2,4]triazolo[4,3-a]pyridine-6-sulfonamide **8e**.

X:\2010\10 11\101119\M7000\34068493.D Sample Name: 8e Page 1 of 1

-.o.-Syntez Purity Report -.o.-

Agilent 1100 LC/MSD SL — Mobile Phase:A-H2O+0.1%HCOOH;B-MeCN+0.1HCOOH
Diodearrey G1315B (DAD1A-215nm; DAD1B-254nm) — Separation column:
Mass Quad G1956B (MSD1-Pos, MSD2-Neg) — Rapid Resolutionn HT Cartige 4.6x30mm,
ELSD Altech 3300 (ADC1 A, ELSD) — 1.8-Micron, Zorbx SB-C18

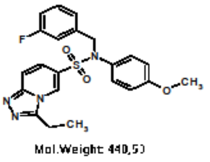

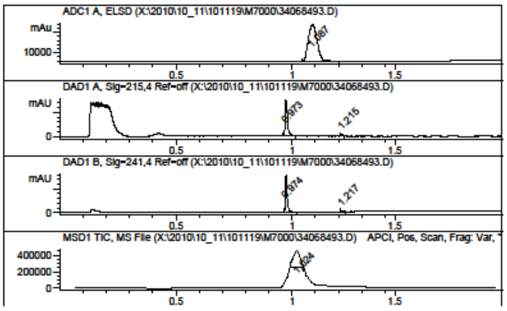

M7000 ->

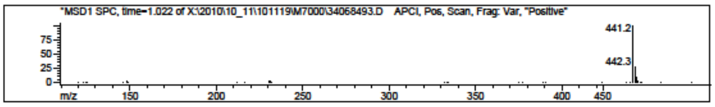

#	Signal	R.Time	Area %
1	ADC1 A, ELSD	1.087	100.000

#	Signal	R.Time	Area %
1	DAD1 A, Sig=215,4 Ref=off	0.973	97.029
2		1.215	2.971

#	Signal	R.Time	Area %
1	DAD1 B, Sig=241,4 Ref=off	0.974	97.823
2		1.217	2.177

#	Signal	R.Time	Area %
1	MSD1 TIC, MS File	1.024	100.000

Figure S36. LC/MS data for 3-Ethyl-N-(3-fluorobenzyl)-N-(4-methoxyphenyl)-[1,2,4]triazolo[4,3-a]pyridine-6-sulfonamide **8e**.

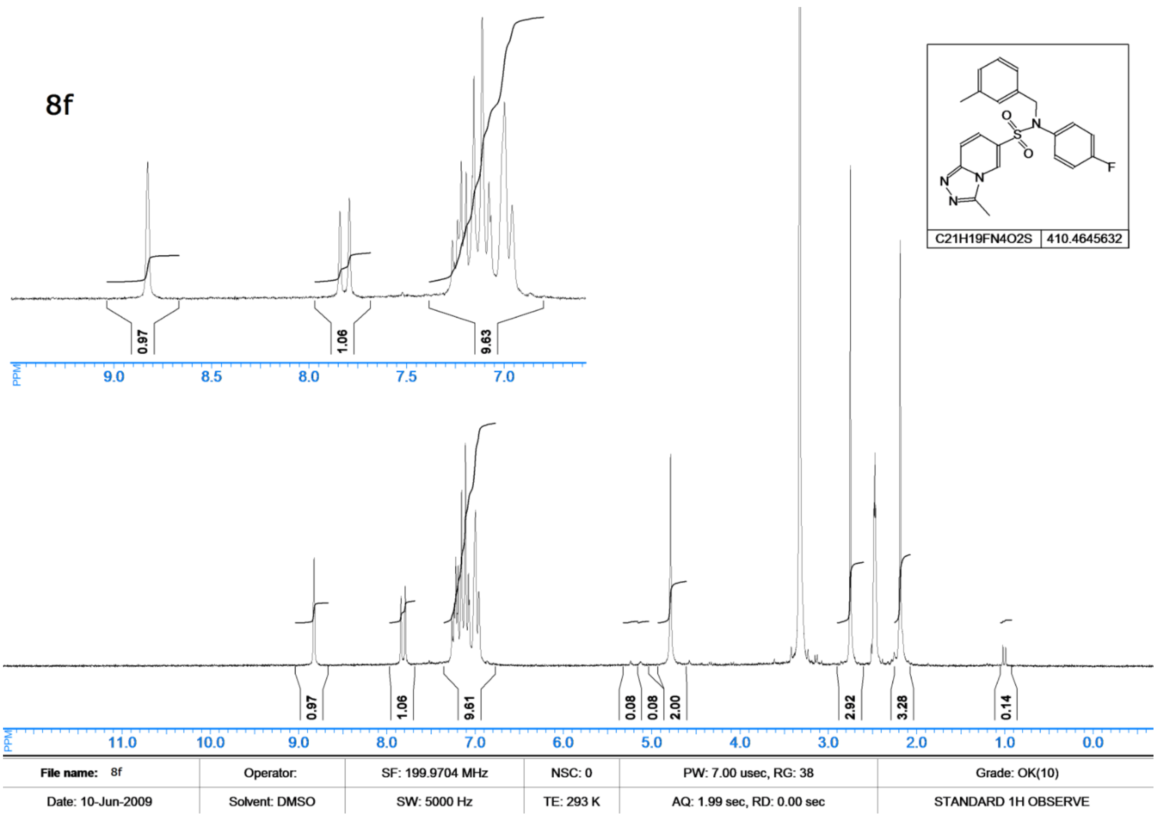

Figure S37. ^{1}H NMR spectrum (200 MHz, DMSO-d6) of N-(4-fluorophenyl)-3-methyl-N-(3-methylbenzyl)-[1,2,4]triazolo[4,3-a]pyridine-6-sulfonamide **8f**.

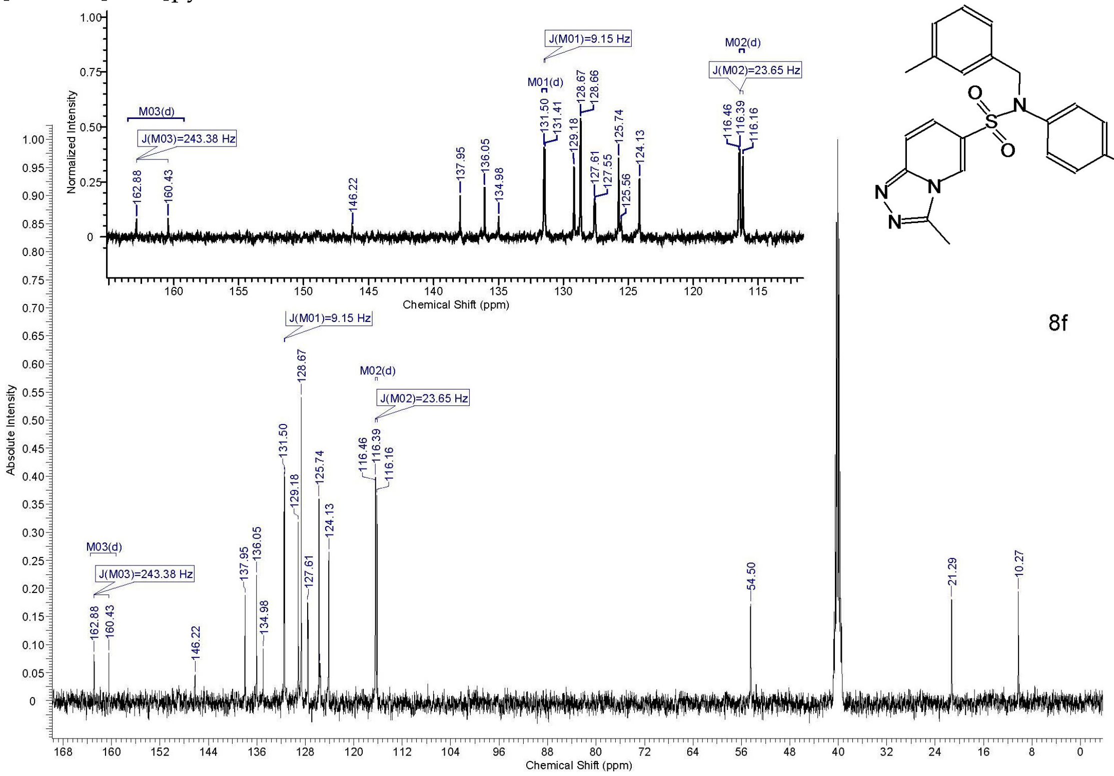

Figure S38. ^{13}C NMR spectrum (100 MHz, DMSO-d6) of N-(4-fluorophenyl)-3-methyl-N-(3-methylbenzyl)-[1,2,4]triazolo[4,3-a]pyridine-6-sulfonamide **8f**.

X:\2010\10_11\101101\M6940\34068550.D Sample Name: 8f Page 1 of

-.o.-Syntez Purity Report -.o.-

Agilent 1100 LC/MSD SL
Diodearrey G1315B (DAD1A-215nm; DAD1B-254nm)
Mass Quad G1956B (MSD1-Pos, MSD2-Neg)
ELSD Altech 3300 (ADC1 A, ELSD)

Mobile Phase:A-H2O+0.1%HCOOH;B-MeCN+0.1HCC
Separation column:
Rapid Resolutionn HT Cartige 4.6x30mm,
1.8-Micron, Zorbx SB-C18

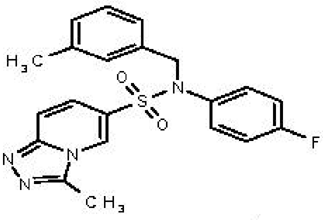

Mol.Weight: 410.47

M6940 ->

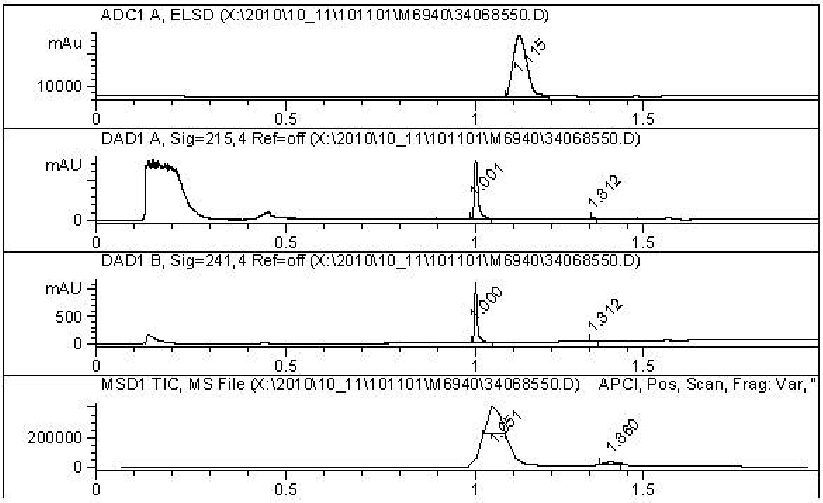

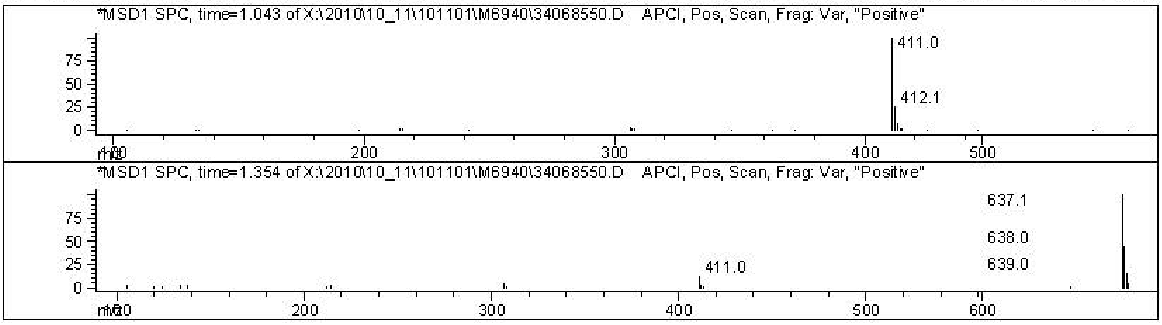

#	Signal	R.Time	Area %
1	ADC1 A, ELSD	1.115	100.000

#	Signal	R.Time	Area %
1	DAD1 A, Sig=215,4 Ref=off	1.001	98.000
2		1.312	2.000

#	Signal	R.Time	Area %
1	DAD1 B, Sig=241,4 Ref=off	1.000	98.392
2		1.312	1.608

#	Signal	R.Time	Area %
1	MSD1 TIC, MS File	1.051	91.905
2		1.360	8.095

Figure S39. LC/MS data for N-(4-fluorophenyl)-3-methyl-N-(3-methylbenzyl)-[1,2,4]triazolo[4,3-a]pyridine-6-sulfonamide **8f**.

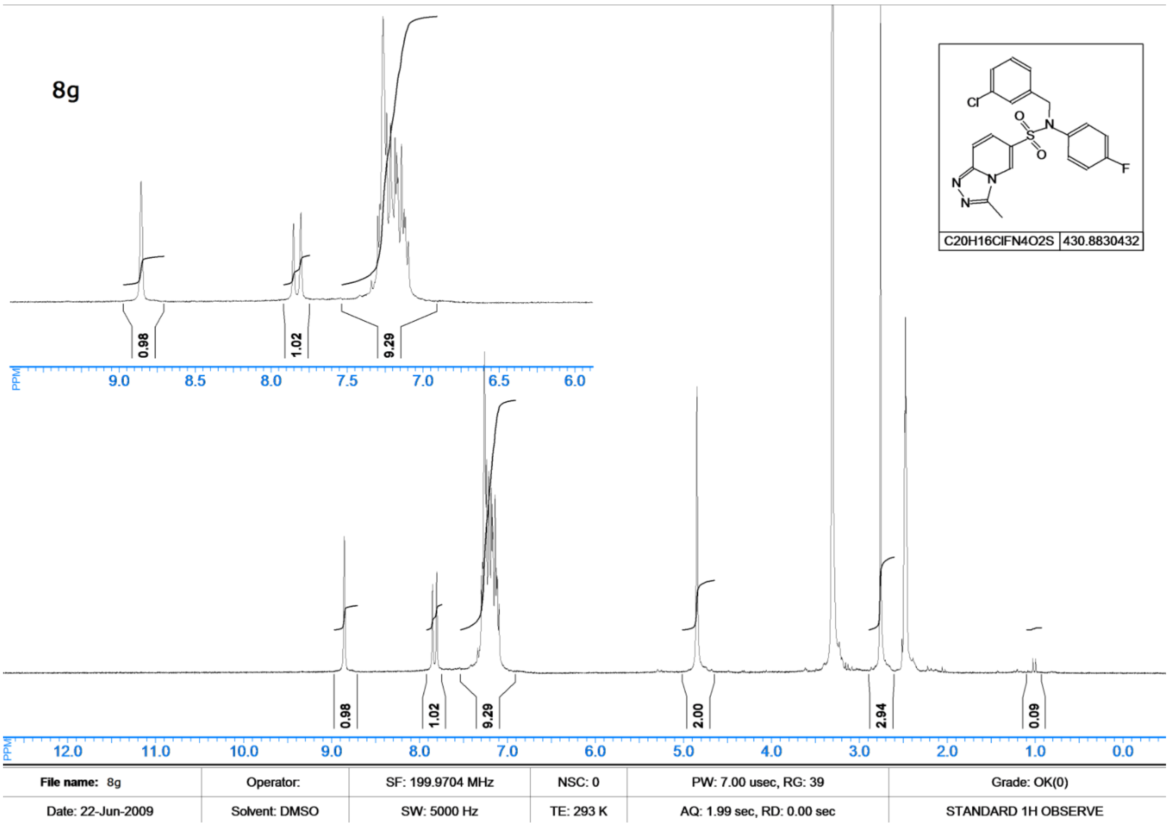

Figure S40. ¹H NMR spectrum (200 MHz, DMSO-d6) of N-(3-chlorobenzyl)-N-(4-fluorophenyl)-3-methyl-[1,2,4]triazolo[4,3-a]pyridine-6-sulfonamide **8g**.

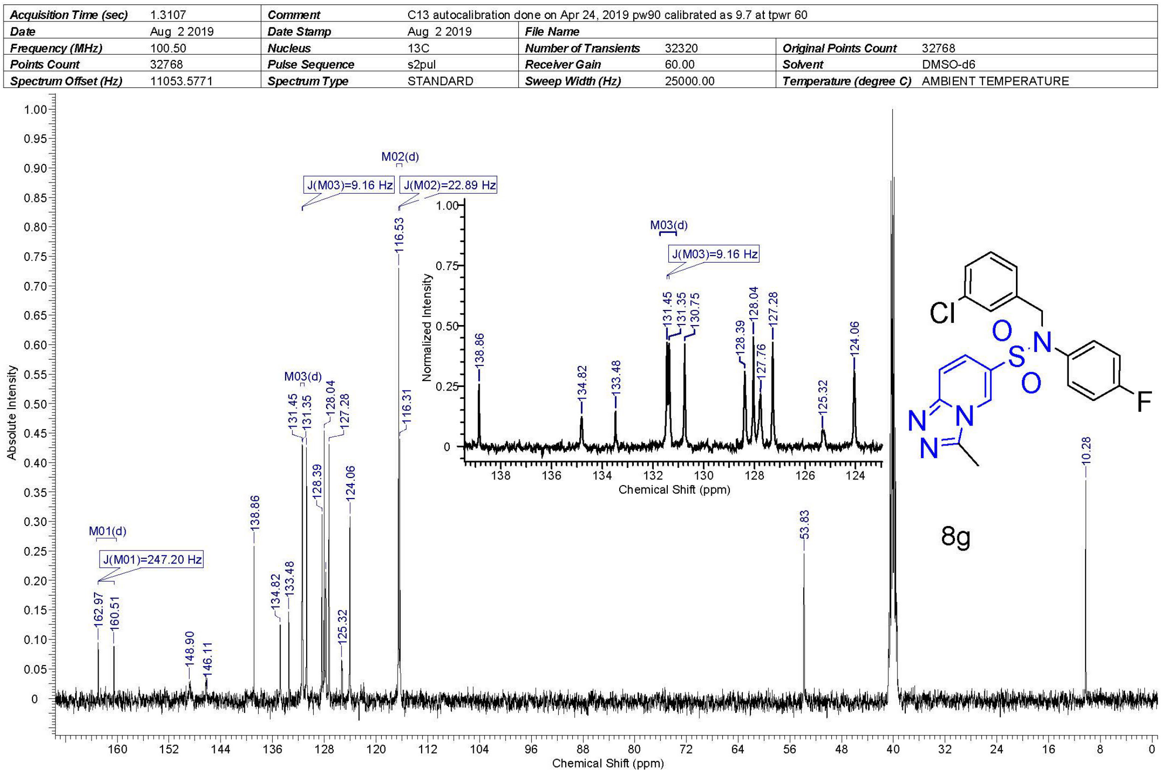

Figure S41. ¹³C NMR spectrum (100 MHz, DMSO-d6) of N-(3-chlorobenzyl)-N-(4-fluorophenyl)-3-methyl-[1,2,4]triazolo[4,3-a]pyridine-6-sulfonamide **8g**.

X:\2010\10_11\101101\M6940\34068559.D Sample Name: 8g Page 1 of 1

-.o.-Syntez Purity Report -.o.-

Agilent 1100 LC/MSD SL
Diodearrey G1315B (DAD1A-215nm; DAD1B-254nm)
Mass Quad G1956B (MSD1-Pos, MSD2-Neg)
ELSD Altech 3300 (ADC1 A, ELSD)

Mobile Phase:A-H2O+0.1%HCOOH;B-MeCN+0.1HCOOH
Separation column:
Rapid Resolutionn HT Cartige 4.6x30mm,
1.8-Micron, Zorbx SB-C18

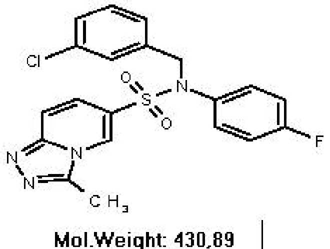

Mol.Weight: 430.89

M6940 ->

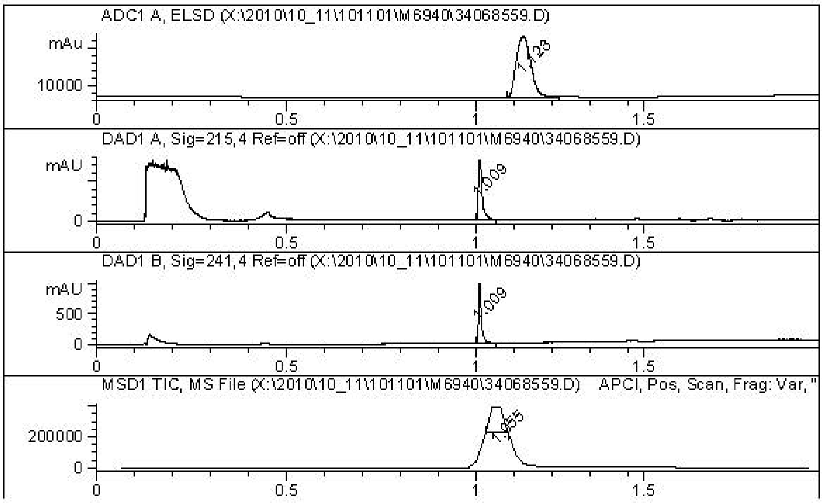

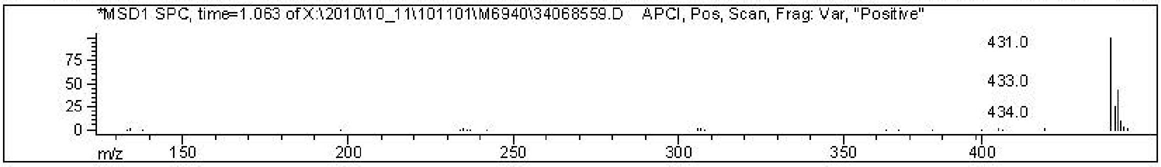

#	Signal	R.Time	Area %
1	ADC1 A, ELSD	1.123	100.000

#	Signal	R.Time	Area %
1	DAD1 A, Sig=215,4 Ref=off	1.009	100.000

#	Signal	R.Time	Area %
1	DAD1 B, Sig=241,4 Ref=off	1.009	100.000

#	Signal	R.Time	Area %
1	MSD1 TIC, MS File	1.055	100.000

Figure S42. LC/MS data for N-(3-chlorobenzyl)-N-(4-fluorophenyl)-3-methyl-[1,2,4]triazolo[4,3-a]pyridine-6-sulfonamide **8g**.

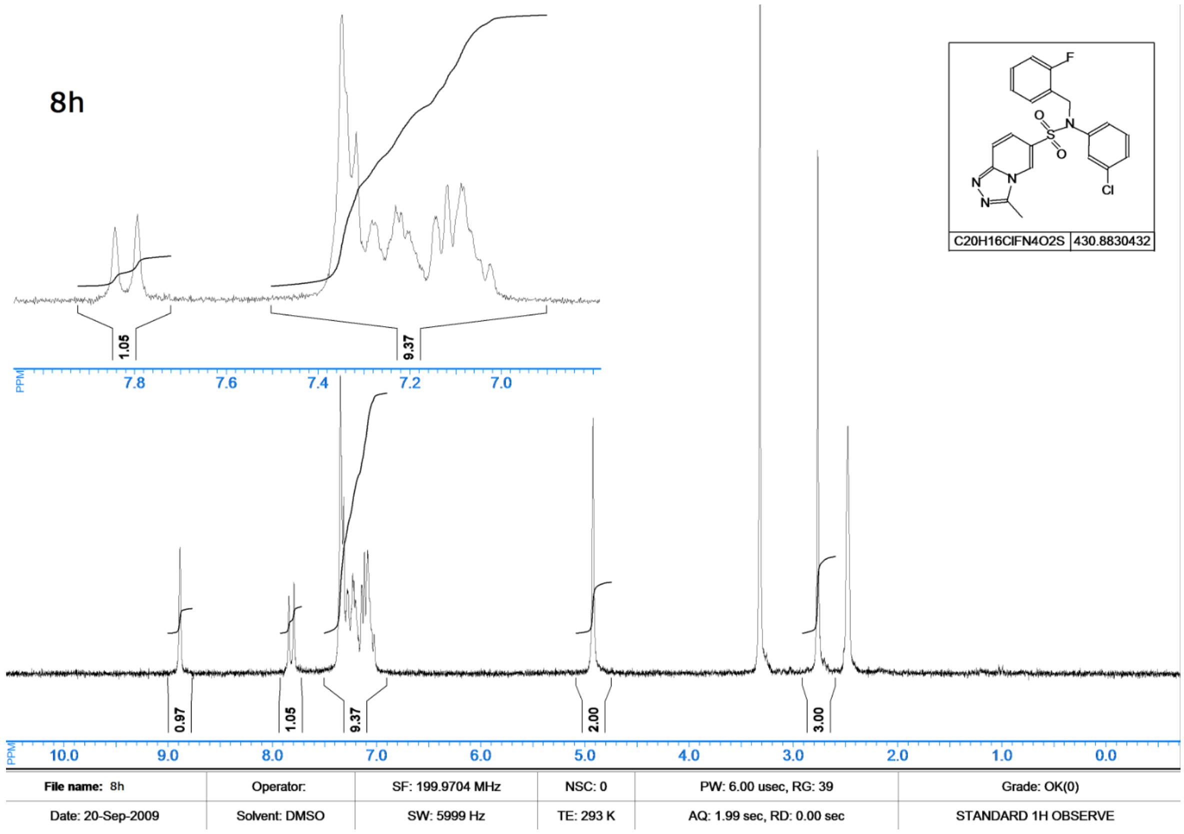

Figure S43. ^{1}H NMR spectrum (200 MHz, DMSO-d6) of N-(3-chlorophenyl)-N-(2-fluorobenzyl)-3-methyl-[1,2,4]triazolo[4,3-a]pyridine-6-sulfonamide **8h**.

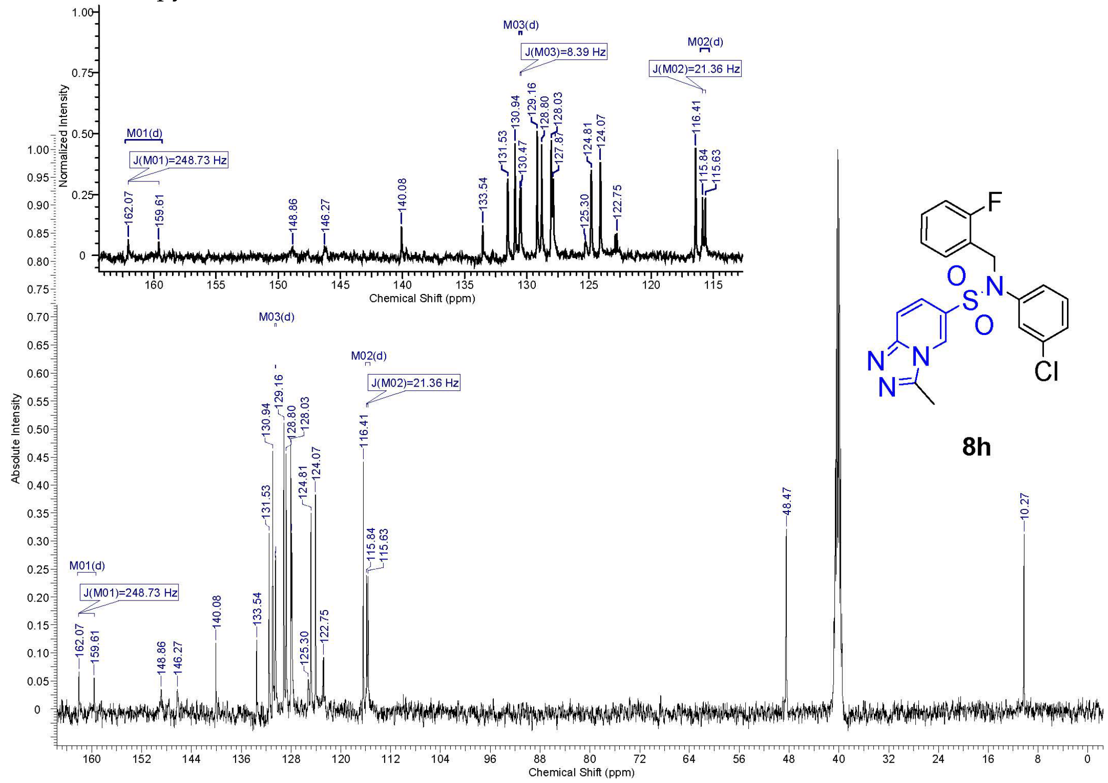

Figure S44. ^{13}C NMR spectrum (100 MHz, DMSO-d6) of N-(3-chlorophenyl)-N-(2-fluorobenzyl)-3-methyl-[1,2,4]triazolo[4,3-a]pyridine-6-sulfonamide **8h**.

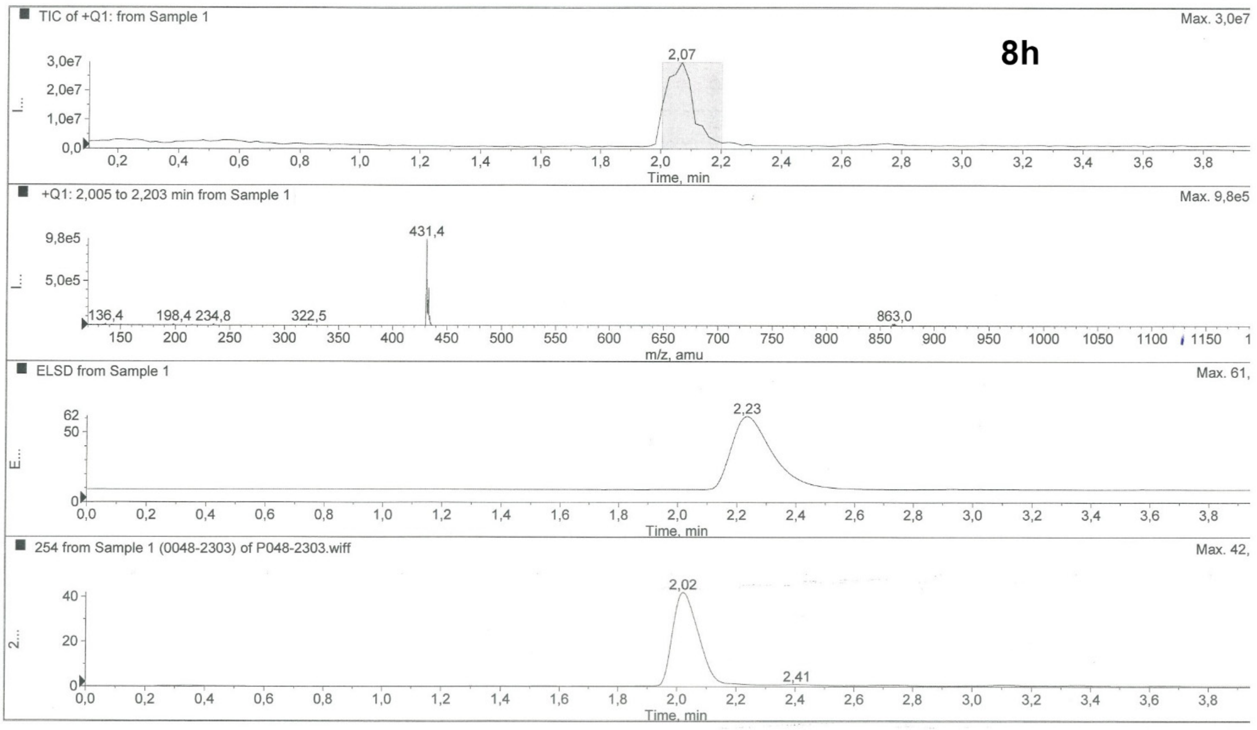

Figure S45. LC/MS data for N-(3-chlorophenyl)-N-(2-fluorobenzyl)-3-methyl-[1,2,4]triazolo[4,3-a]pyridine-6-sulfonamide **8h**.

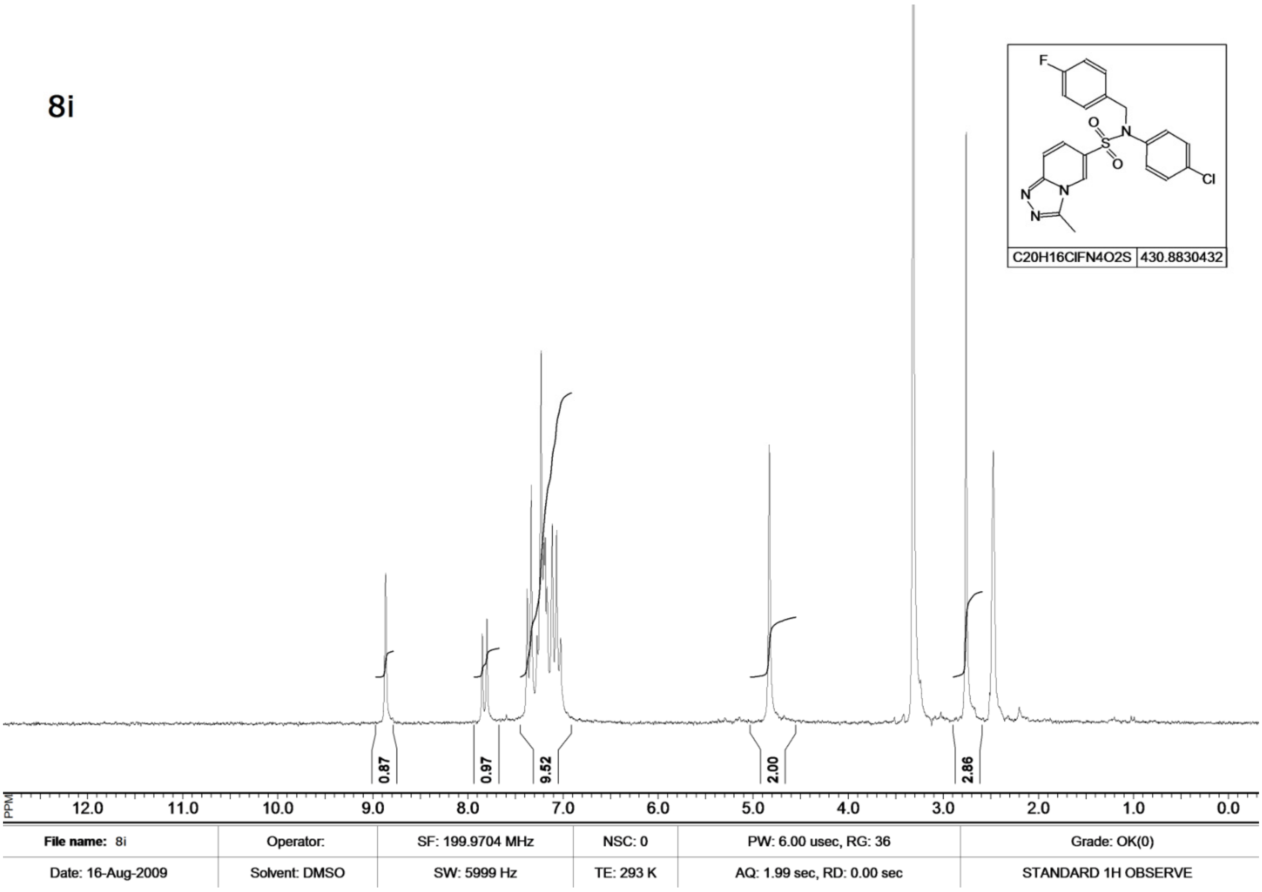

Figure S46. ^{1}H NMR spectrum (200 MHz, DMSO-d6) of N-(4-chlorophenyl)-N-(4-fluorobenzyl)-3-methyl-[1,2,4]triazolo[4,3-a]pyridine-6-sulfonamide **8i**.

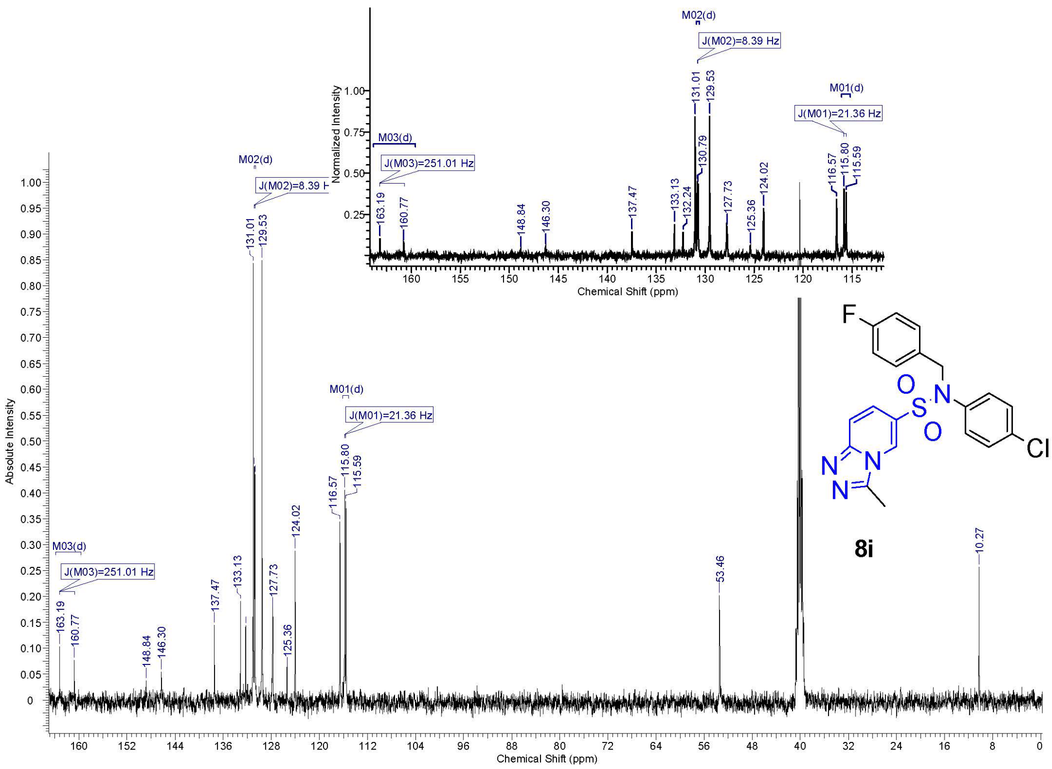

Figure S47. ^{13}C NMR spectrum (100 MHz, DMSO-d6) of N-(4-chlorophenyl)-N-(4-fluorobenzyl)-3-methyl-[1,2,4]triazolo[4,3-a]pyridine-6-sulfonamide **8i**

```
X:\2010\10_12\101225\M7370\34068594.D            Sample Name: 8i                Page 1 of 1
-------------------------------------------------------------------------------------------
                            -.o.-Syntez Purity Report -.o.-
Agilent 1100 LC/MSD SL                          Mobile Phase:A-H2O+0.1%HCOOH;B-MeCN+0.1HCOOH
Diodearrey G1315B (DAD1A-215nm; DAD1B-254nm)    Separation column:
Mass Quad  G1956B (MSD1-Pos,   MSD2-Neg)        Rapid Resolutionn HT Cartige 4.6x30mm,
ELSD Altech 3300  (ADC1 A, ELSD)                1.8-Micron, Zorbx SB-C18
-------------------------------------------------------------------------------------------
```

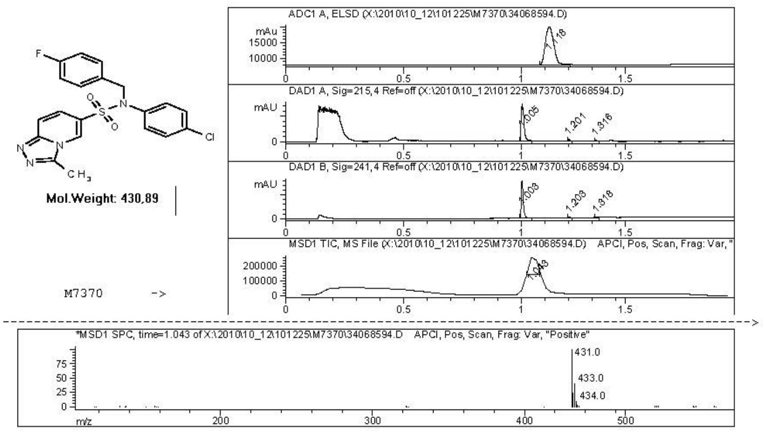

#	Signal	R.Time	Area %
1	ADC1 A, ELSD	1.118	100.000

#	Signal	R.Time	Area %
1	DAD1 A, Sig=215,4 Ref=off	1.005	95.670
2		1.201	2.385
3		1.316	1.945

#	Signal	R.Time	Area %
1	DAD1 B, Sig=241,4 Ref=off	1.003	96.801
2		1.203	1.994
3		1.318	1.206

#	Signal	R.Time	Area %
1	MSD1 TIC, MS File	1.043	100.000

Figure S48. LC/MS data for N-(4-chlorophenyl)-N-(4-fluorobenzyl)-3-methyl-[1,2,4]triazolo[4,3-a]pyridine-6-sulfonamide **8i.**

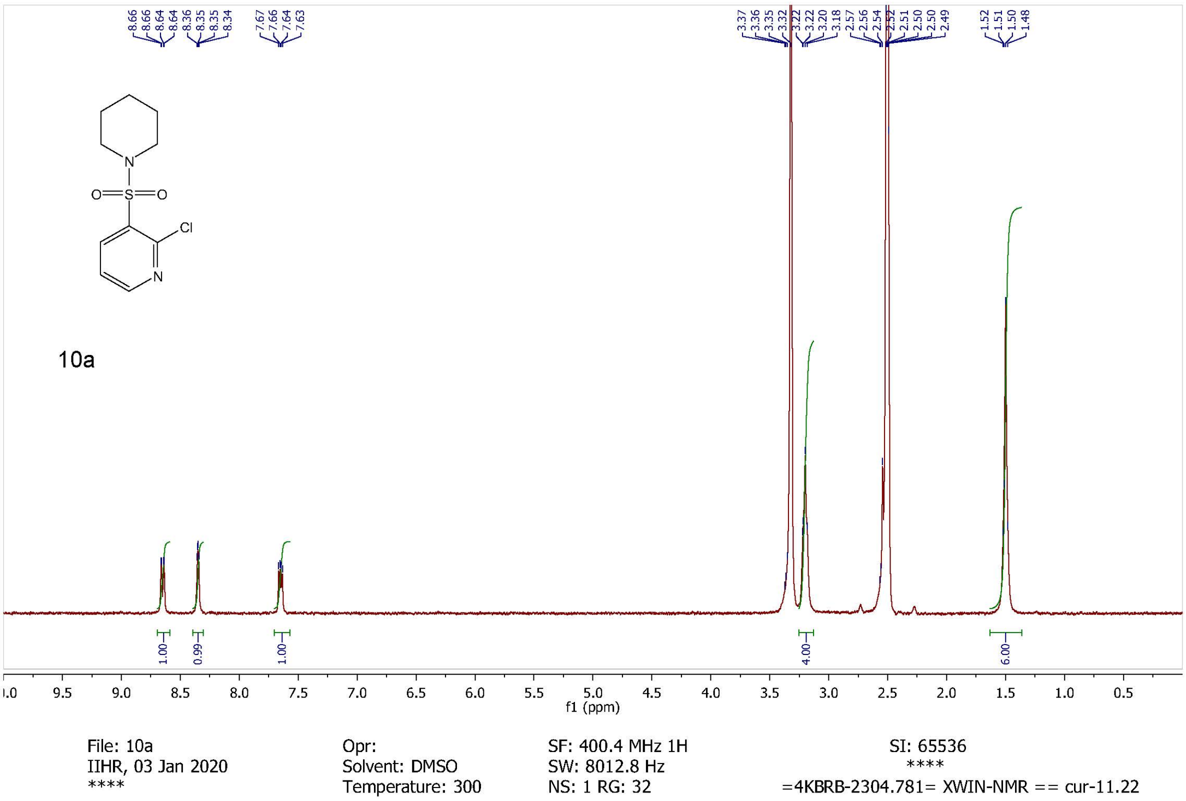

Figure S49. ^{1}H NMR spectrum (400 MHz, DMSO-d6) of 2-chloro-3-(piperidin-1-ylsulfonyl)pyridine **10a**.

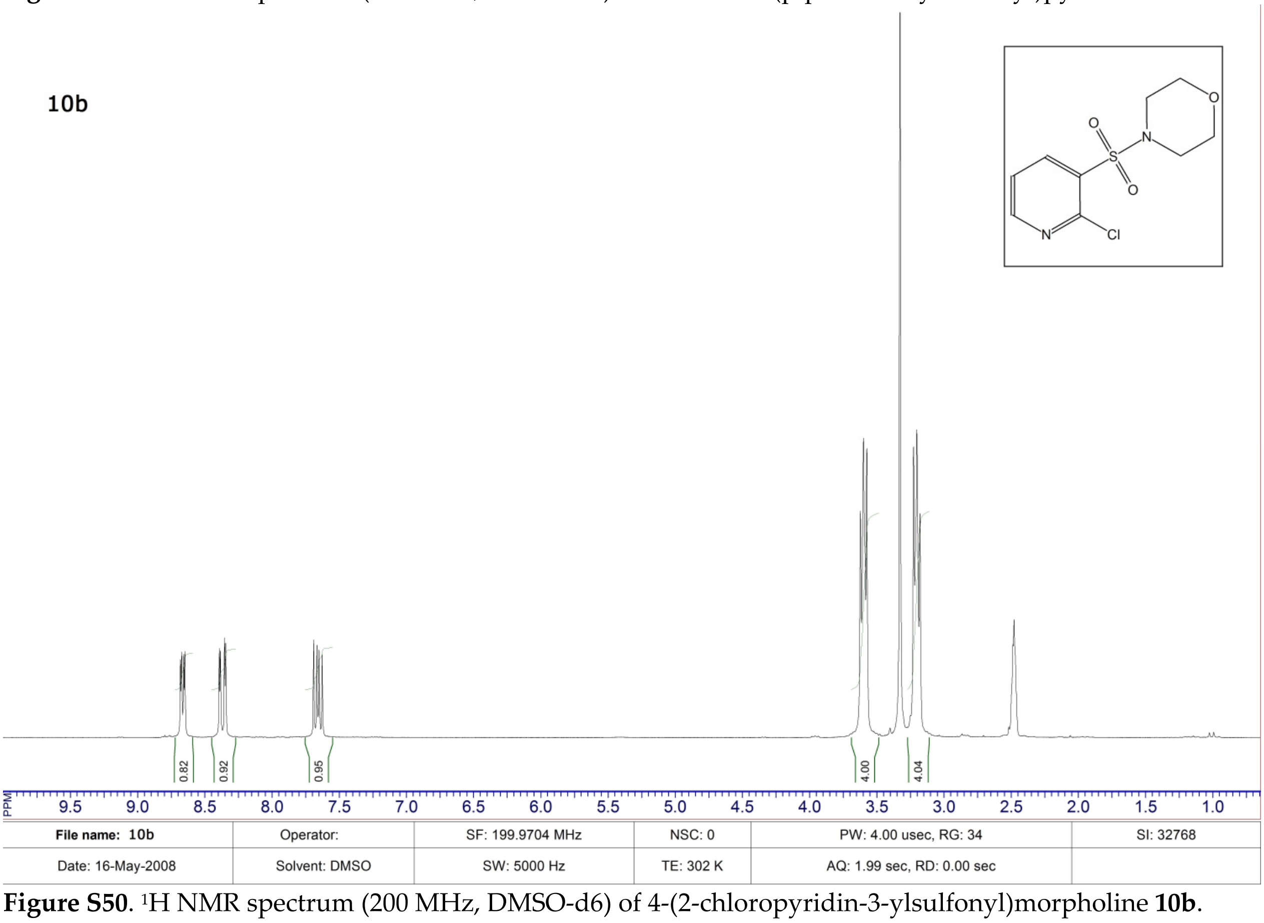

Figure S50. ^{1}H NMR spectrum (200 MHz, DMSO-d6) of 4-(2-chloropyridin-3-ylsulfonyl)morpholine **10b**.

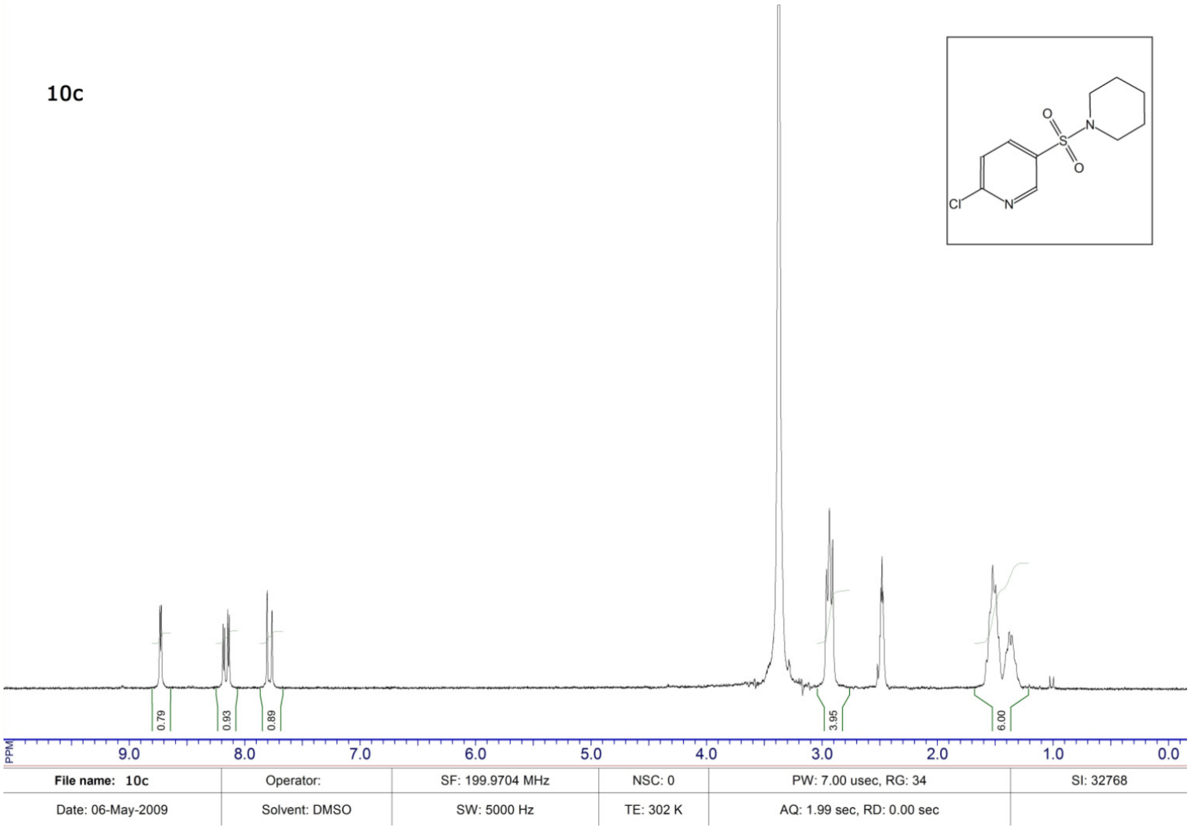

Figure S51. ^{1}H NMR spectrum (200 MHz, DMSO-d6) of 2-chloro-5-(piperidin-1-ylsulfonyl)pyridine **10c**.

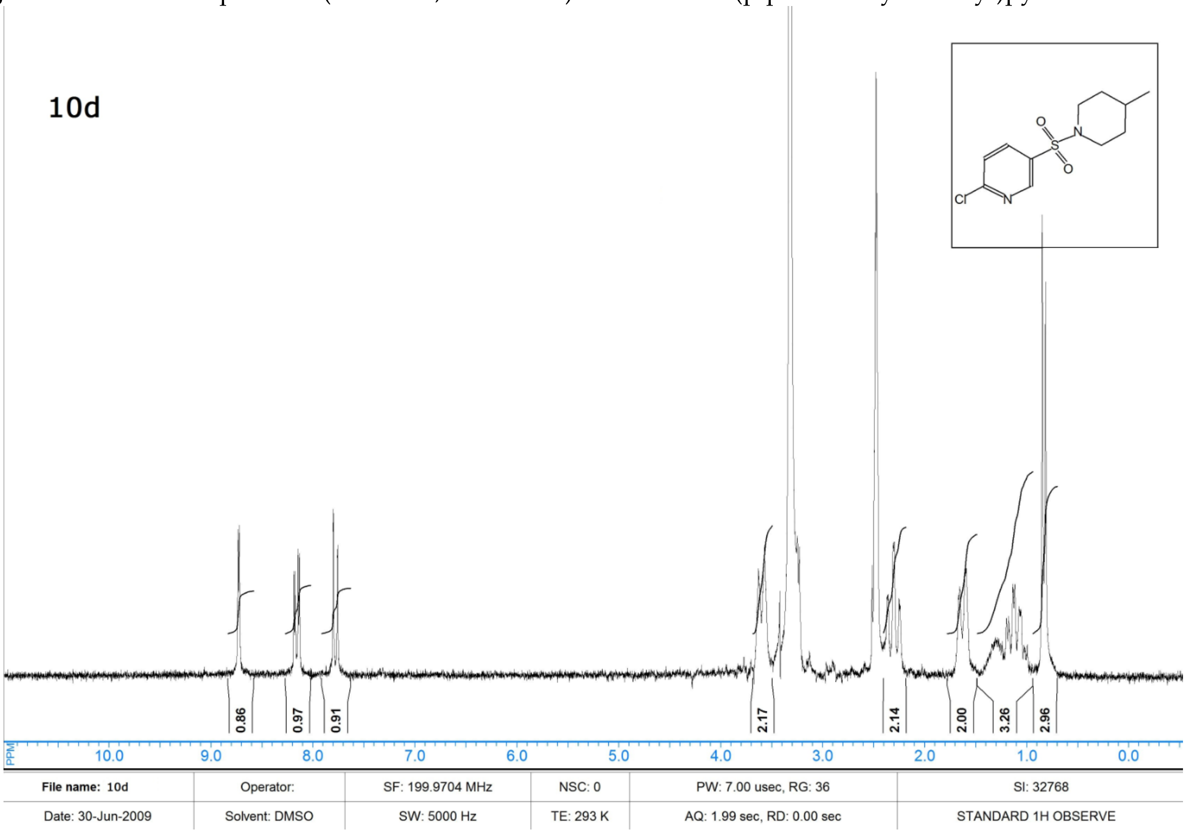

Figure S52. ^{1}H NMR spectrum (200 MHz, DMSO-d6) of 2-chloro-5-(4-methylpiperidin-1-ylsulfonyl)pyridine **10d**.

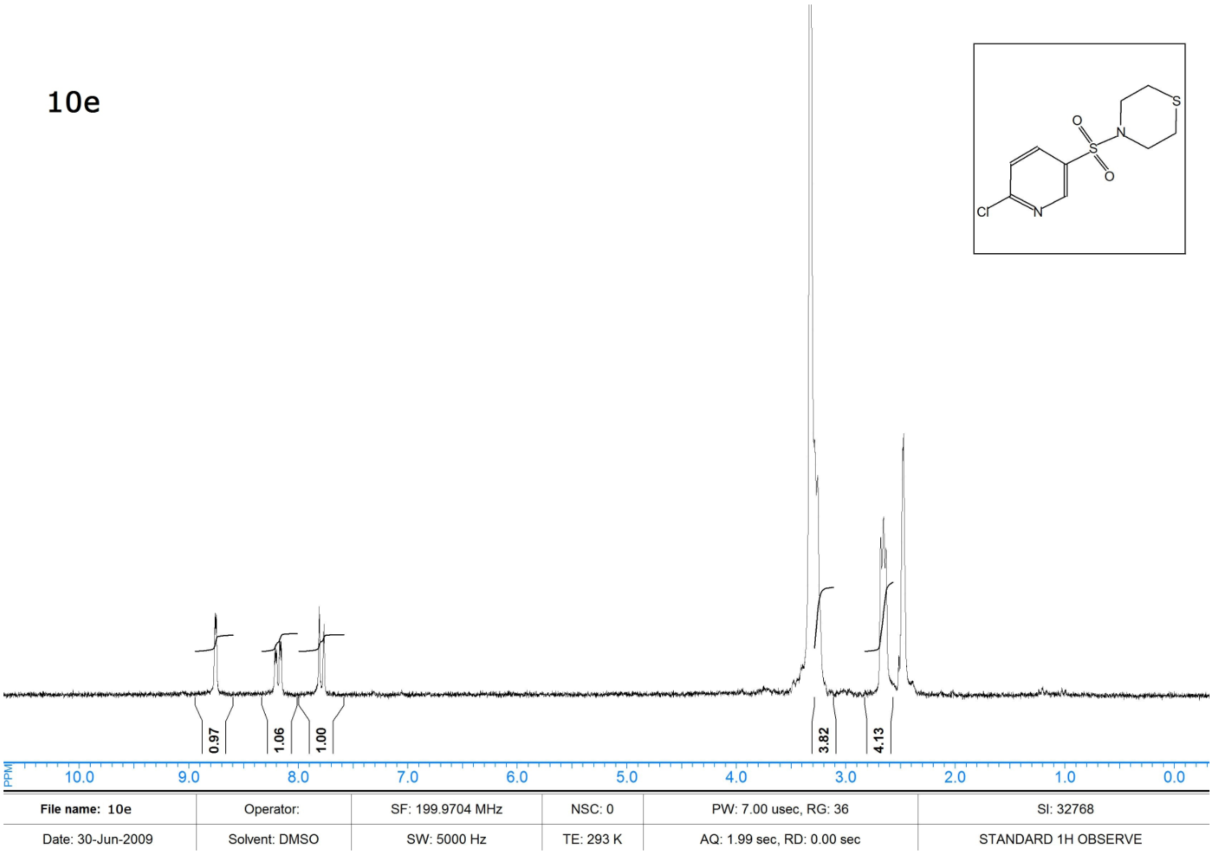

Figure S53. ^{1}H NMR spectrum (200 MHz, DMSO-d6) of 4-(6-chloropyridin-3-ylsulfonyl)thiomorpholine **10e**.

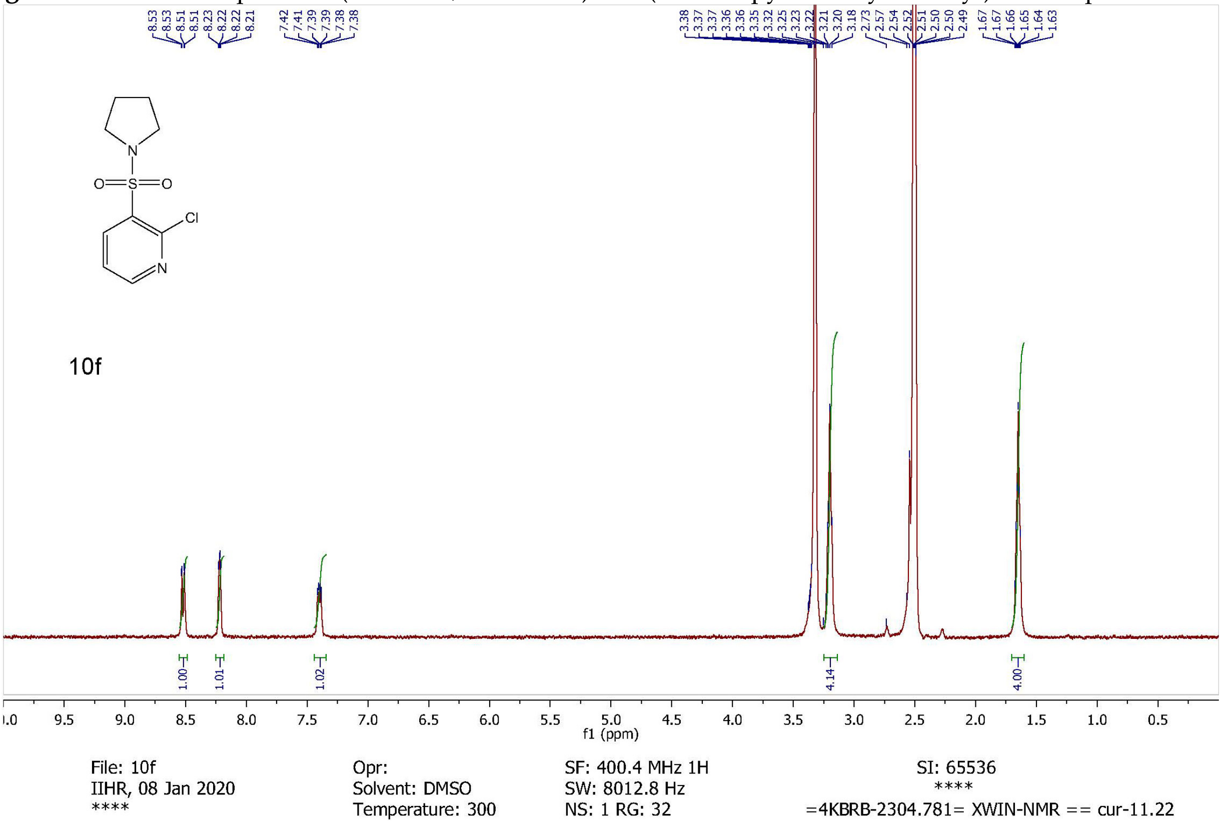

Figure S54. ^{1}H NMR spectrum (400 MHz, DMSO-d6) of 2-chloro-3-(pyrrolidin-1-ylsulfonyl)pyridine **10f.**

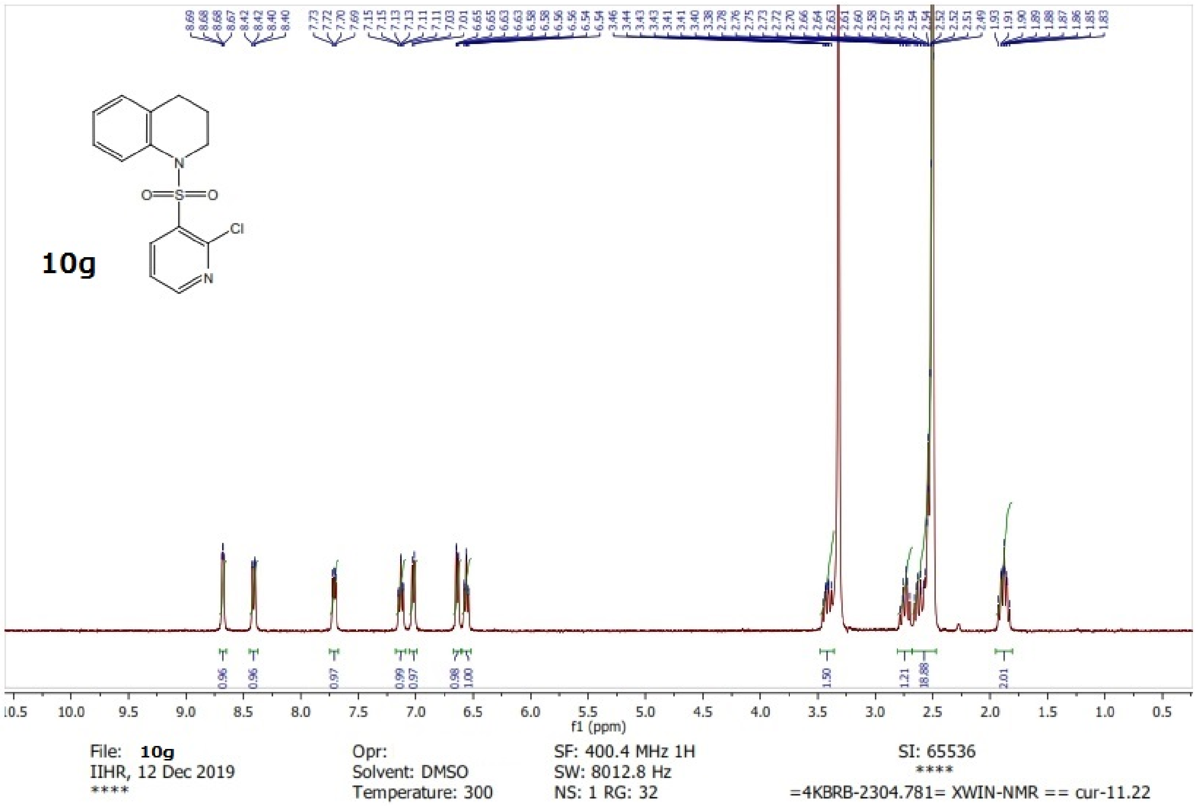

Figure S55. ^{1}H NMR spectrum (400 MHz, DMSO-d6) of 1-(2-chloropyridin-3-ylsulfonyl)-1,2,3,4-tetrahydroquinoline **10g**.

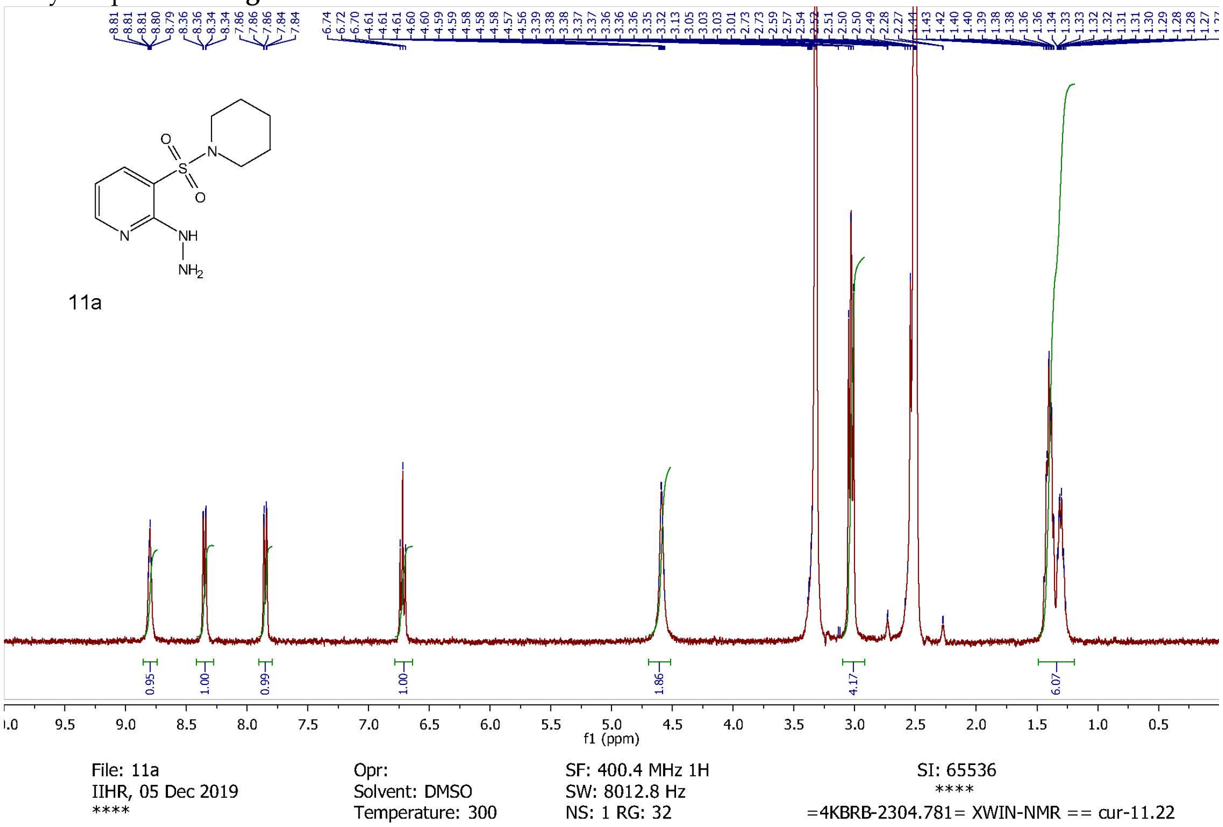

Figure S56. ^{1}H NMR spectrum (400 MHz, DMSO-d6) of 2-hydrazinyl-3-(piperidin-1-ylsulfonyl)pyridine **11a**.

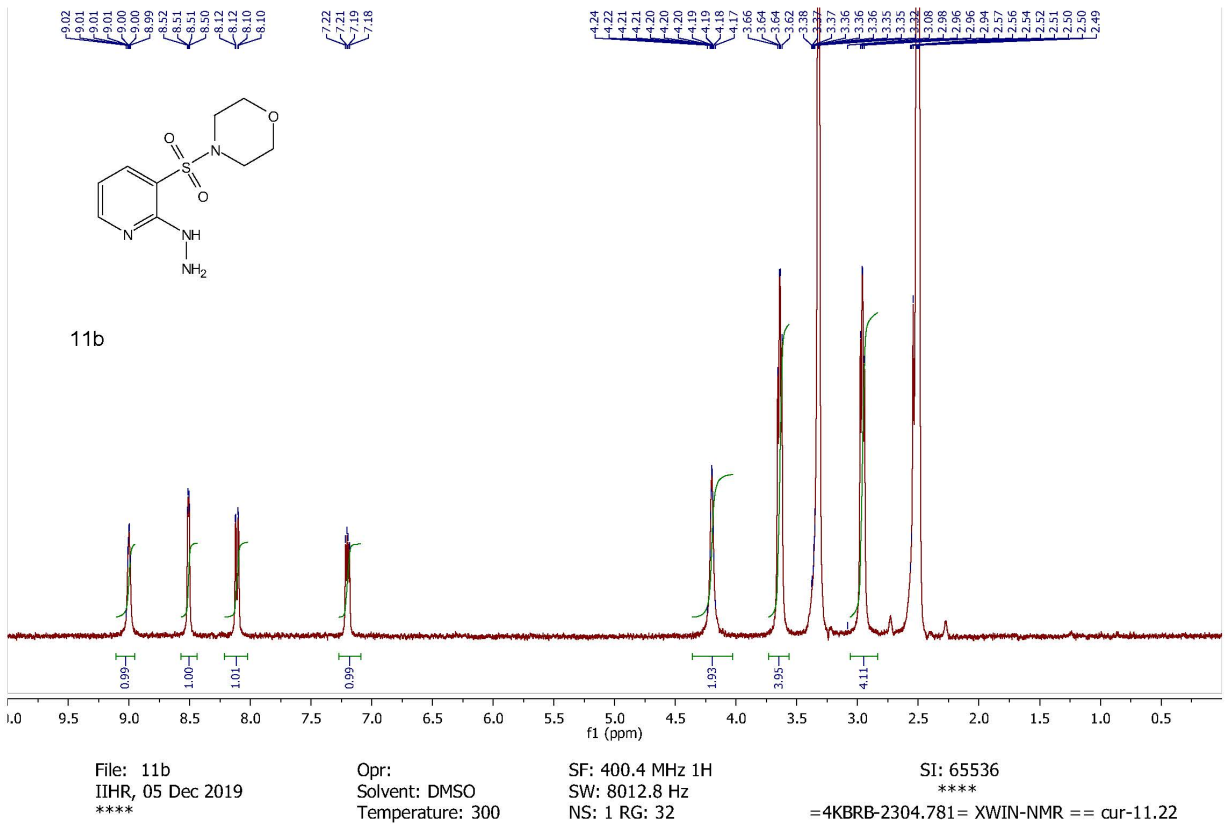

Figure S57. ^{1}H NMR spectrum (400 MHz, DMSO-d6) of 4-(2-hydrazinylpyridin-3-ylsulfonyl)morpholine **11b**.

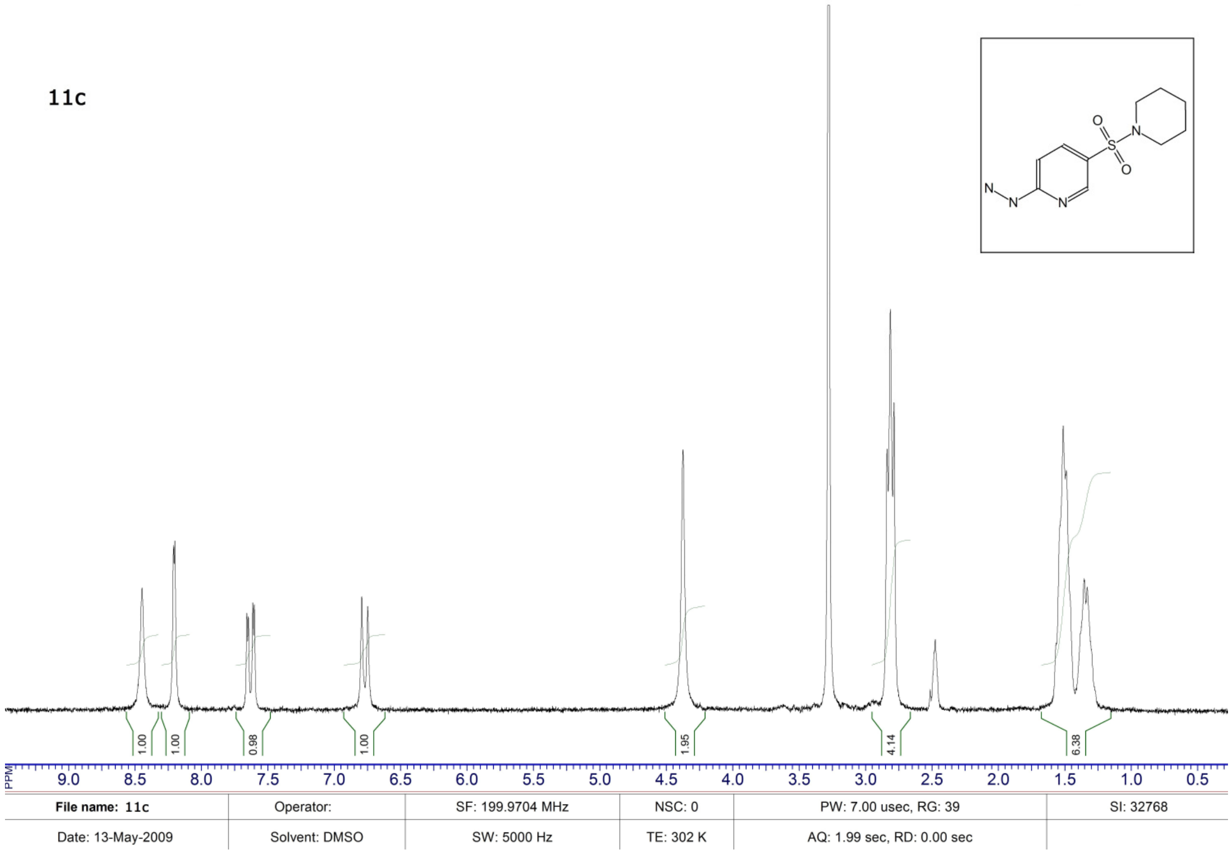

Figure S58. ^{1}H NMR spectrum (200 MHz, DMSO-d6) of 2-hydrazinyl-5-(piperidin-1-ylsulfonyl)pyridine **11c**.

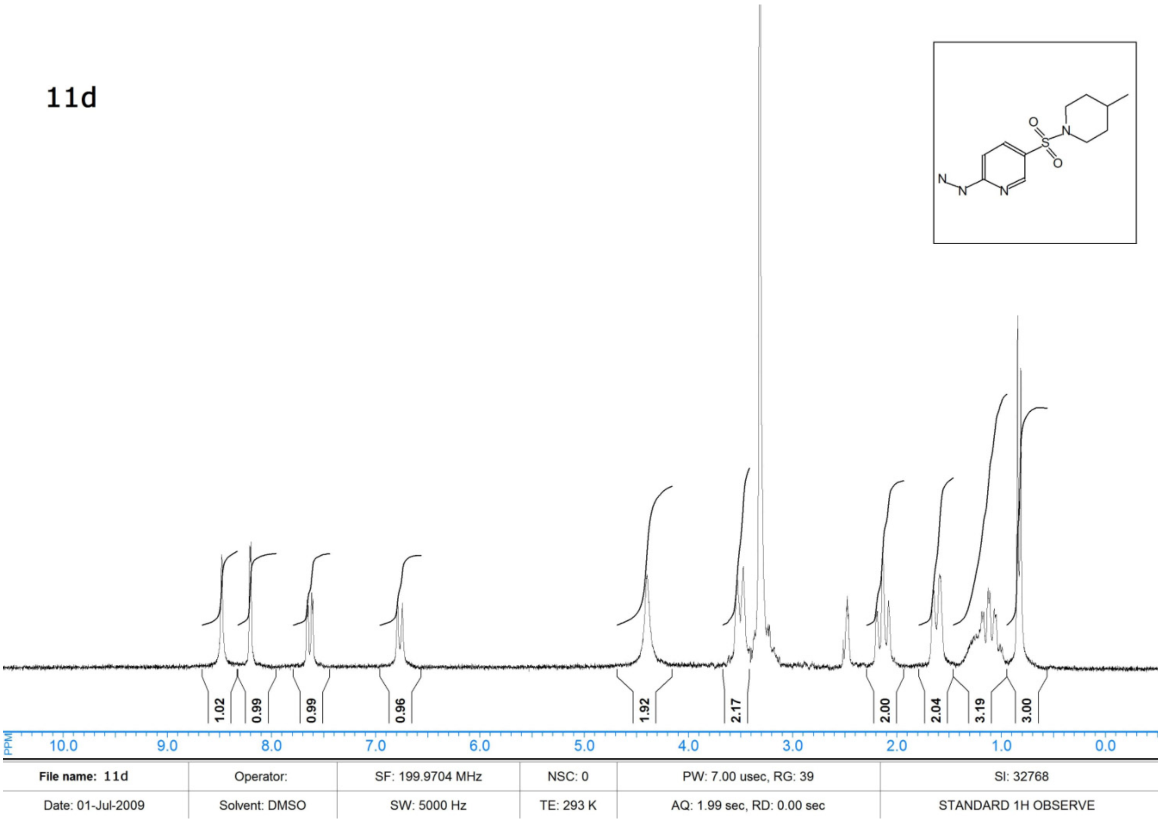

Figure S59. ^{1}H NMR spectrum (200 MHz, DMSO-d6) of 2-hydrazinyl-5-(4-methylpiperidin-1-ylsulfonyl)pyridine **11d**.

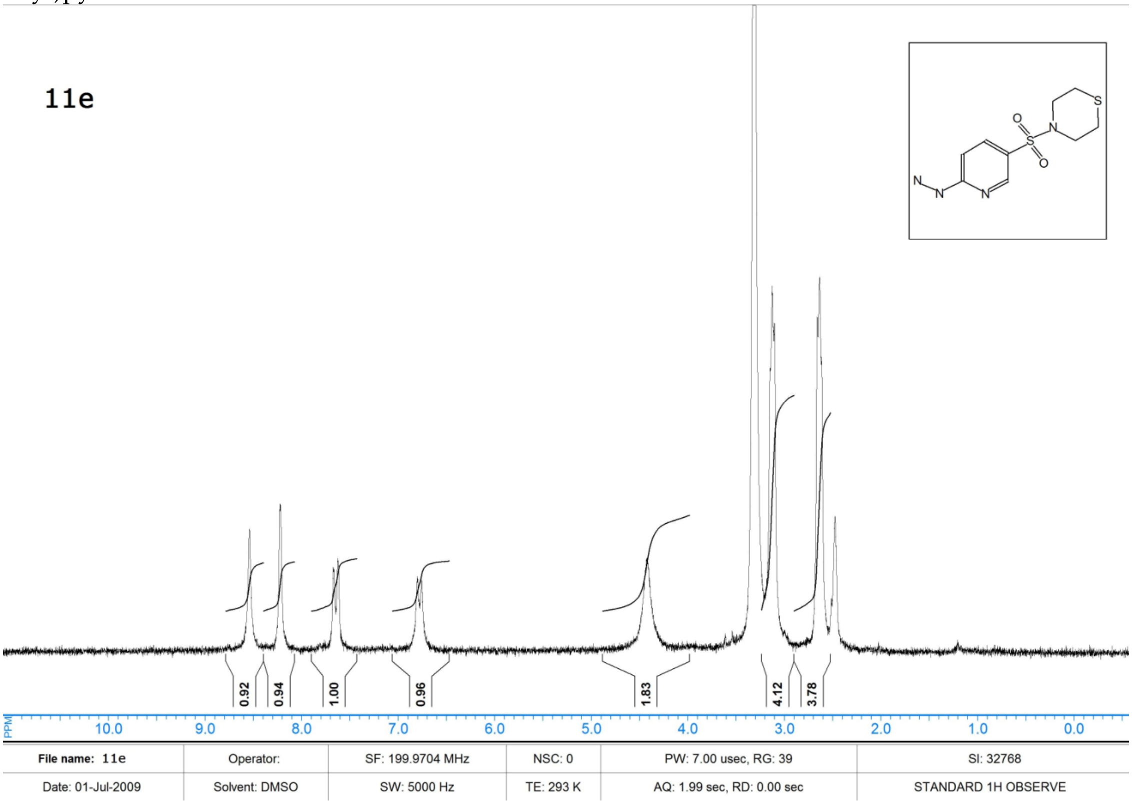

Figure S60. ^{1}H NMR spectrum (200 MHz, DMSO-d6) of 4-(6-hydrazinylpyridin-3-ylsulfonyl)thiomorpholine **11e.**

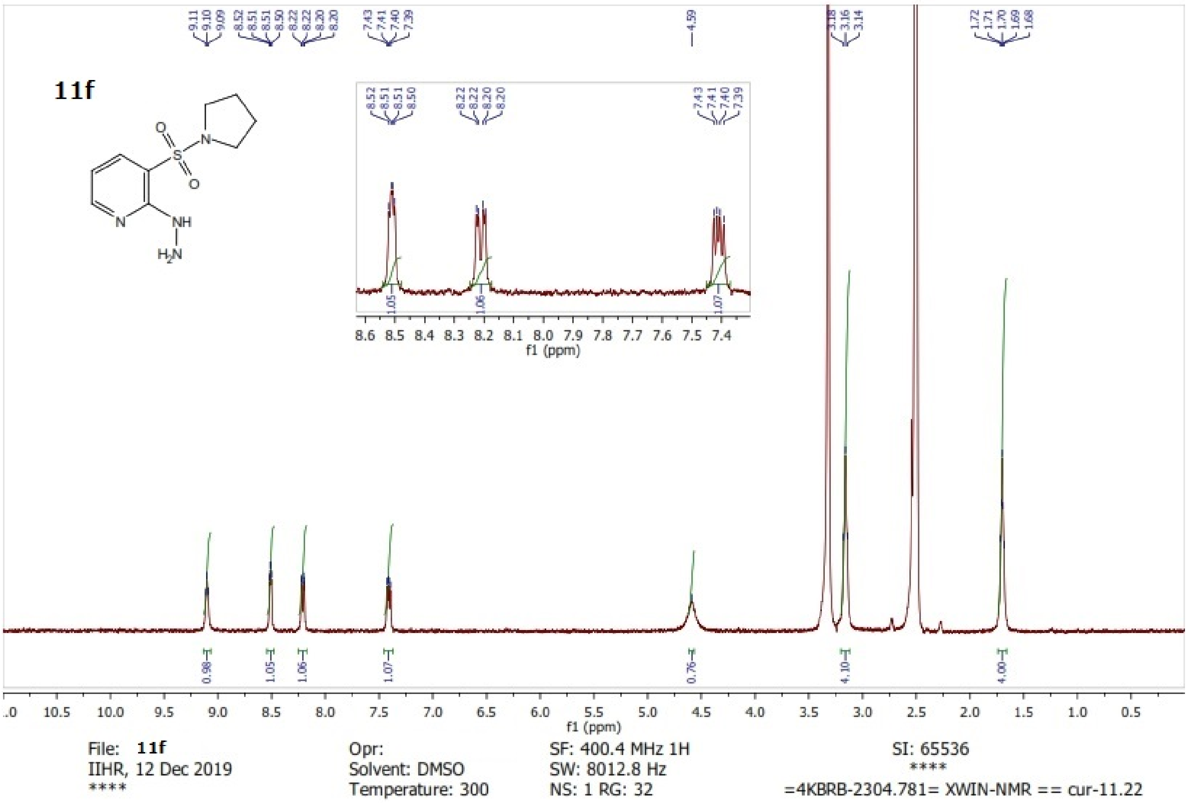

Figure S61. ^{1}H NMR spectrum (400 MHz, DMSO-d6) of 2-hydrazinyl-3-(pyrrolidin-1-ylsulfonyl)pyridine **11f.**

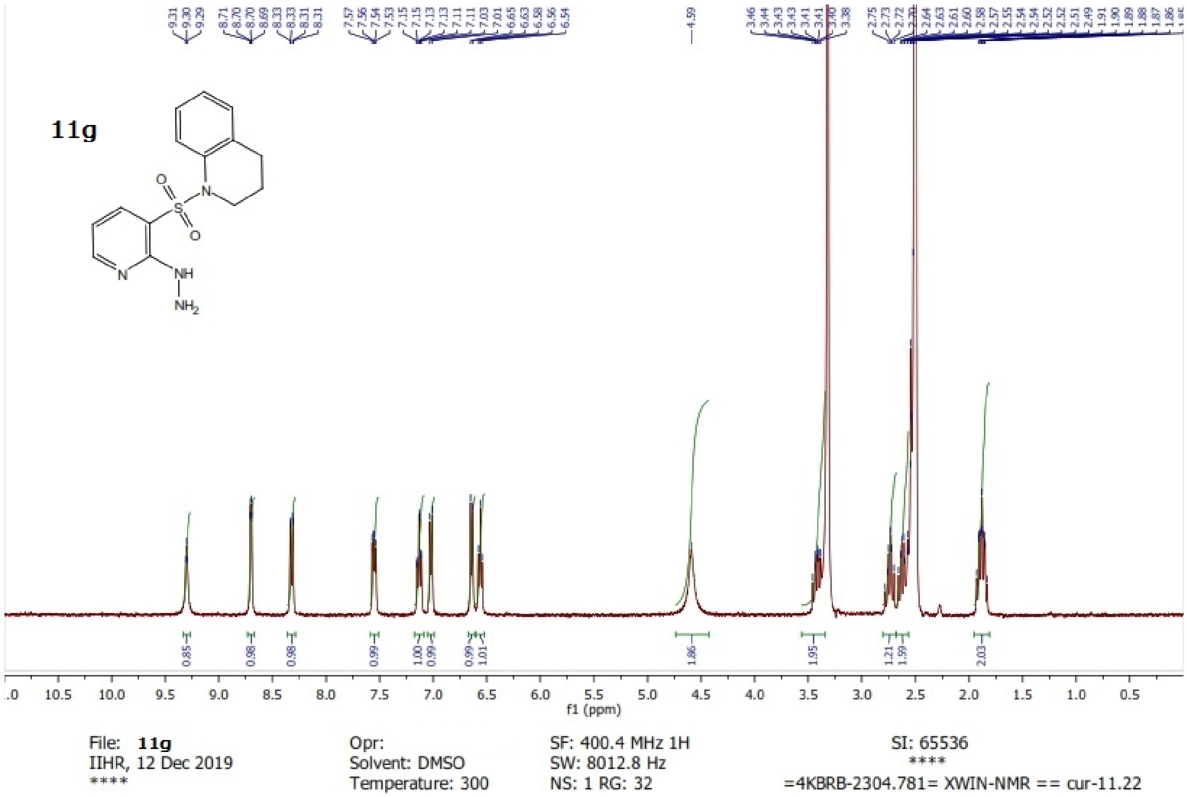

Figure S62. ^{1}H NMR spectrum (400 MHz, DMSO-d6) of 1-(2-hydrazinylpyridin-3-ylsulfonyl)-1,2,3,4-tetrahydroquinoline **11g**.

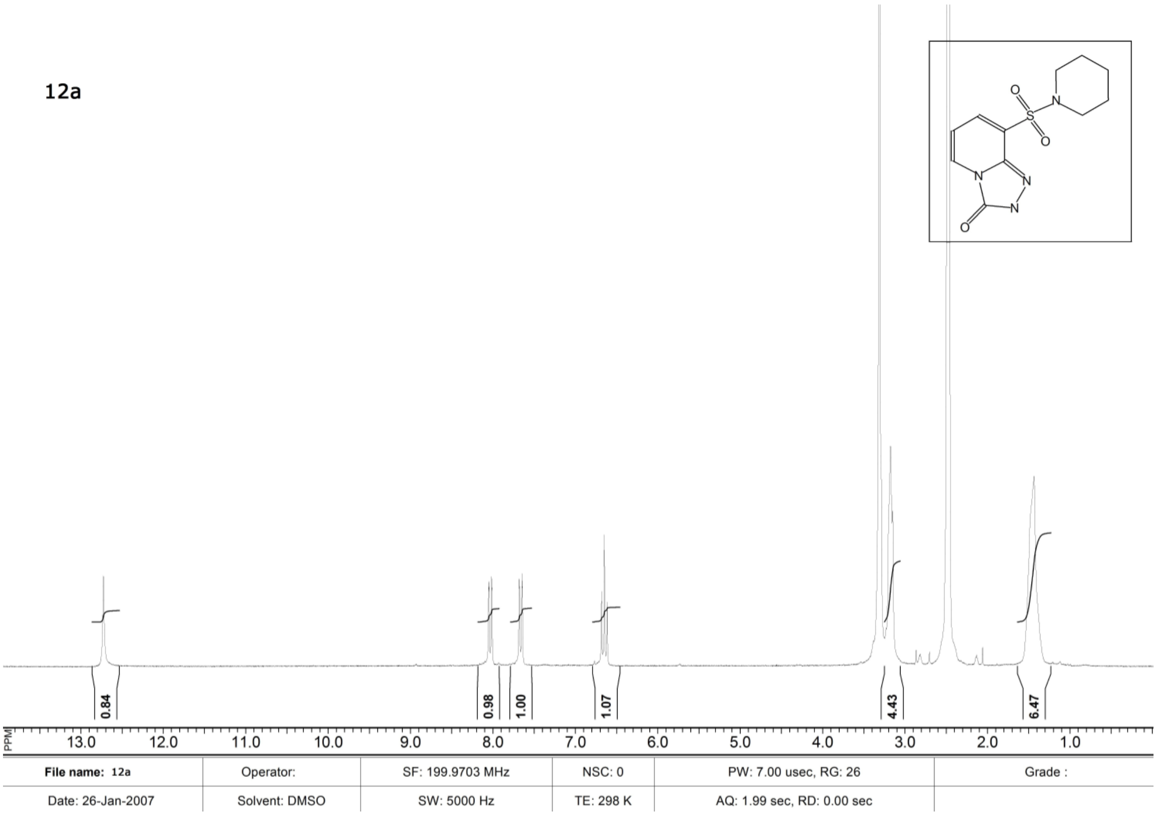

Figure S63. ^{1}H NMR spectrum (200 MHz, DMSO-d6) of 8-(piperidin-1-ylsulfonyl)-[1,2,4]triazolo[4,3-a]pyridin-3(2H)-one **12a.**

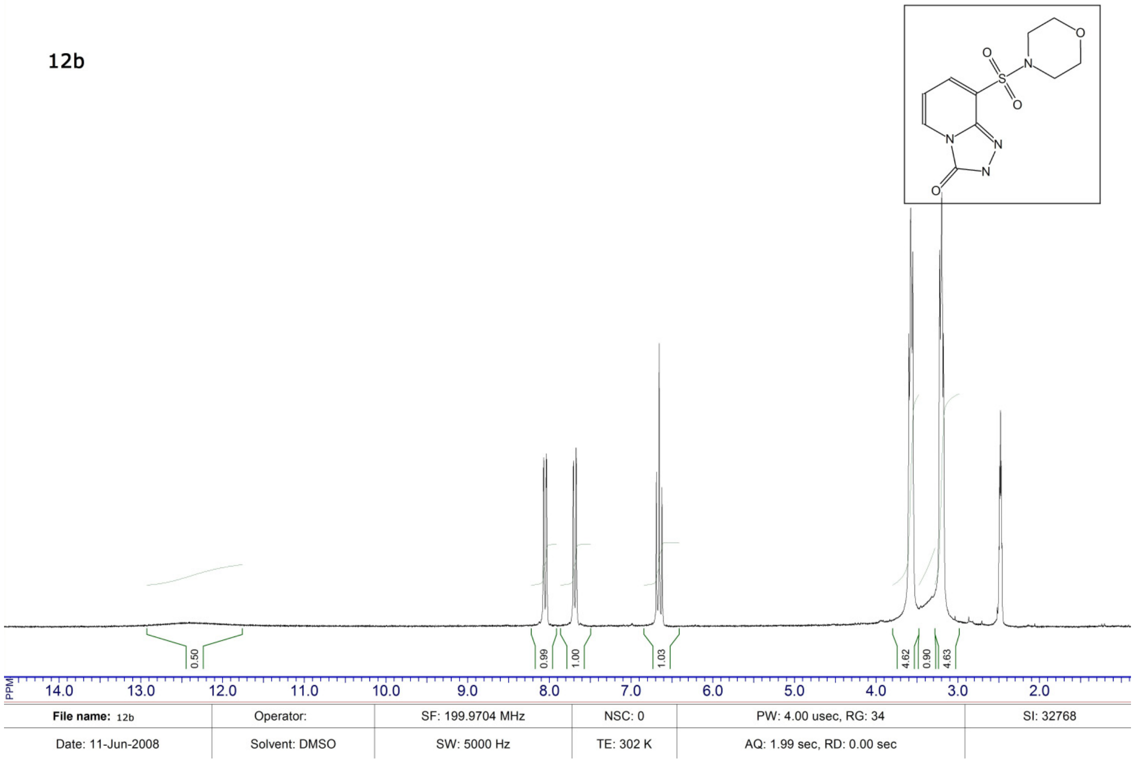

Figure S64. ^{1}H NMR spectrum (200 MHz, DMSO-d6) of 8-(morpholinosulfonyl)-[1,2,4]triazolo[4,3-a]pyridin-3(2H)-one **12b**.

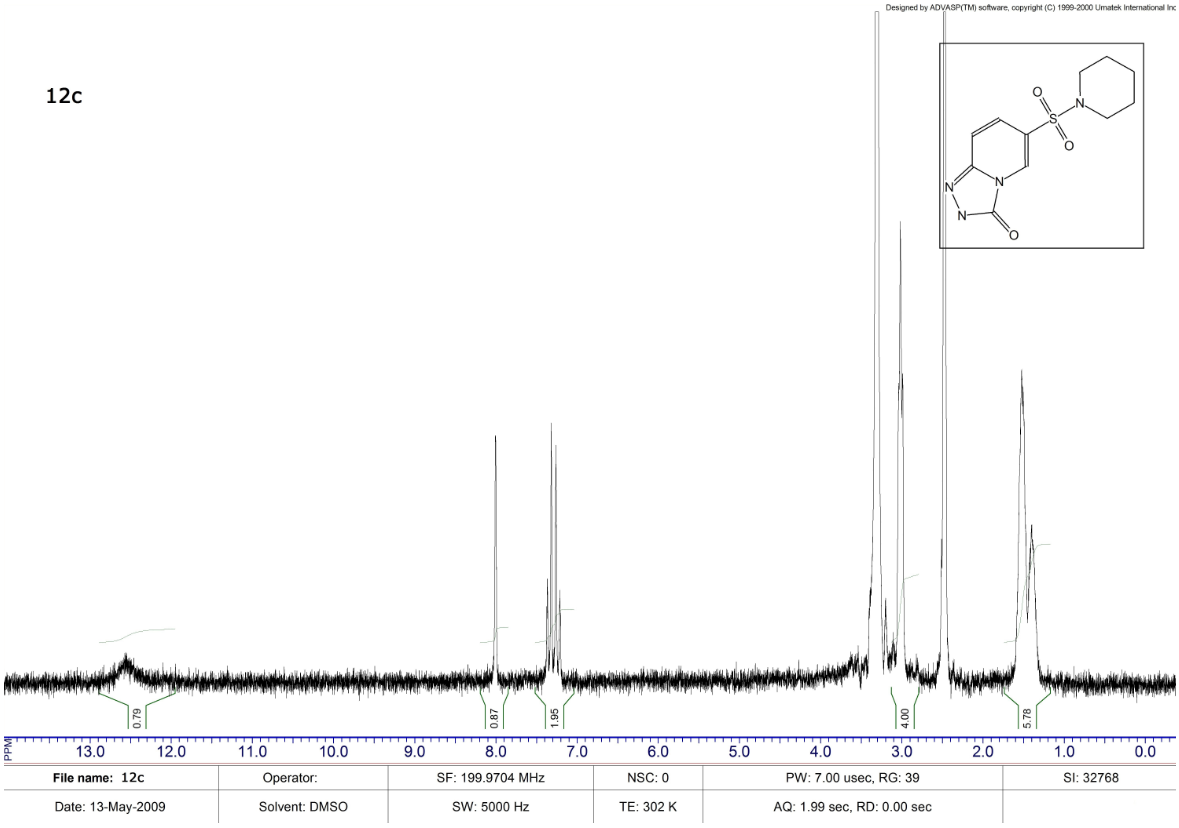

Figure S65. ^{1}H NMR spectrum (200 MHz, DMSO-d6) of 6-(piperidin-1-ylsulfonyl)-[1,2,4]triazolo[4,3-a]pyridin-3(2H)-one **12c**.

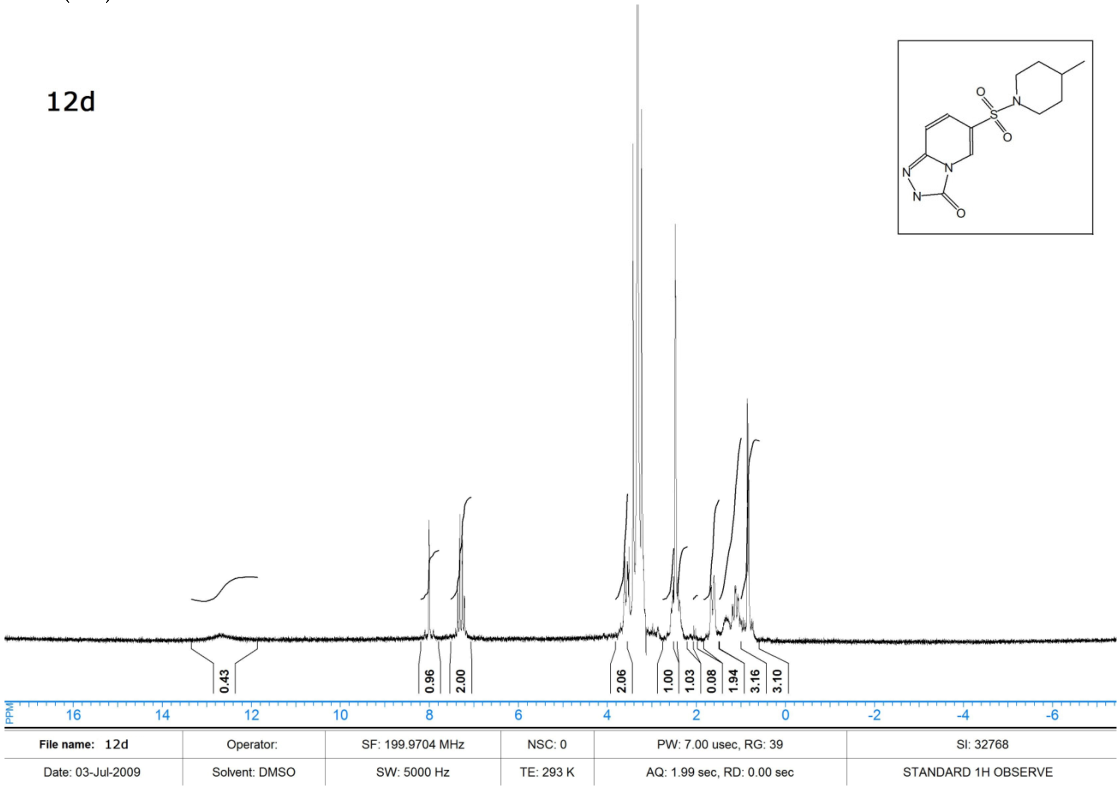

Figure S66. ^{1}H NMR spectrum (200 MHz, DMSO-d6) of 6-(4-methylpiperidin-1-ylsulfonyl)-[1,2,4]triazolo[4,3-a]pyridin-3(2H)-one **12d**.

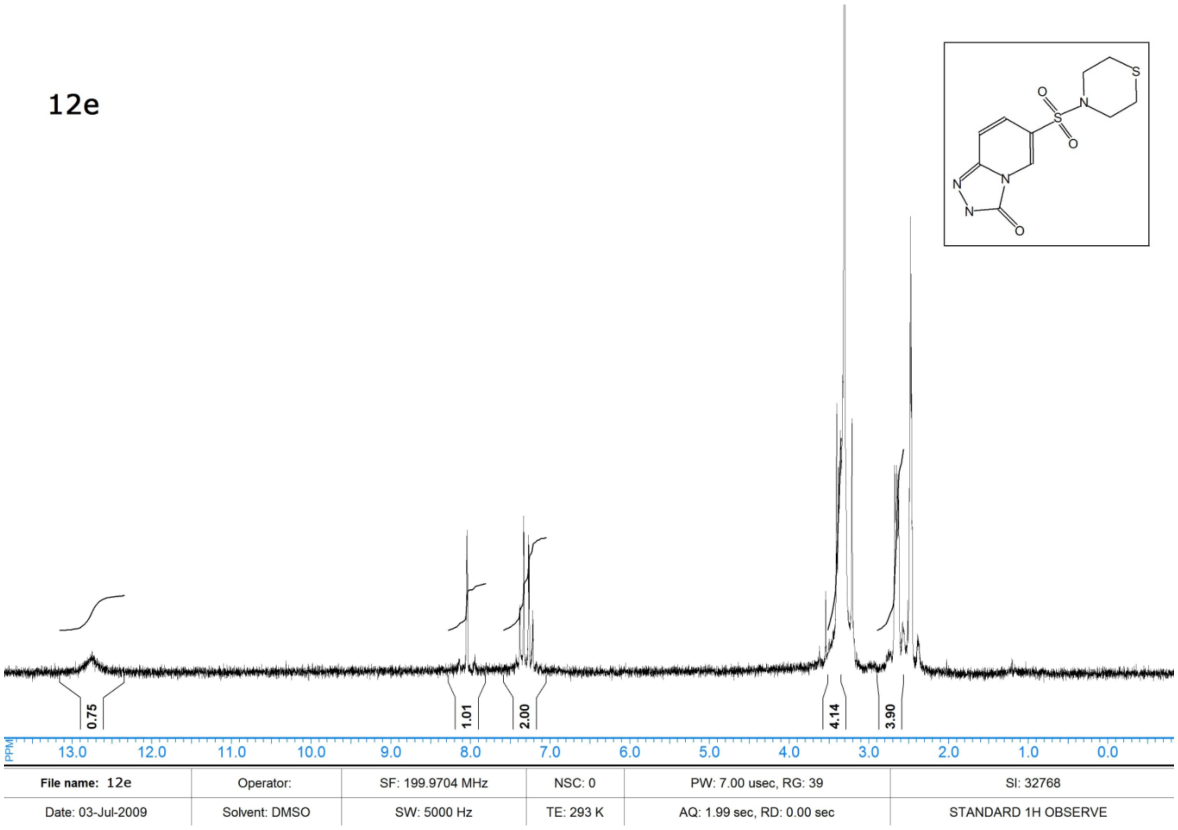

Figure S67. ^{1}H NMR spectrum (200 MHz, DMSO-d6) of 6-(thiomorpholinosulfonyl)-[1,2,4]triazolo[4,3-a]pyridin-3(2H)-one **12e**.

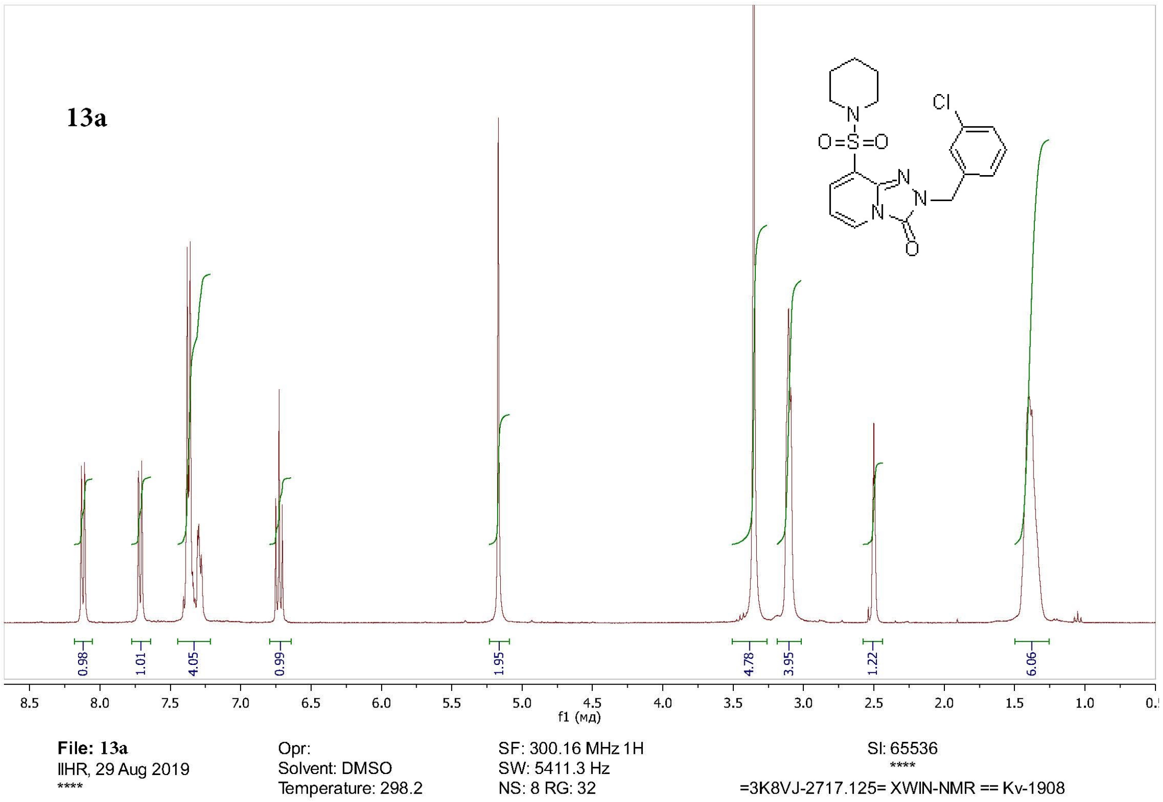

Figure S68. ^{1}H NMR spectrum (300 MHz, DMSO-d6) of 2-(3-chlorobenzyl)-8-(piperidin-1-ylsulfonyl)-[1,2,4]triazolo[4,3-a]pyridin-3(2H)-one **13a**.

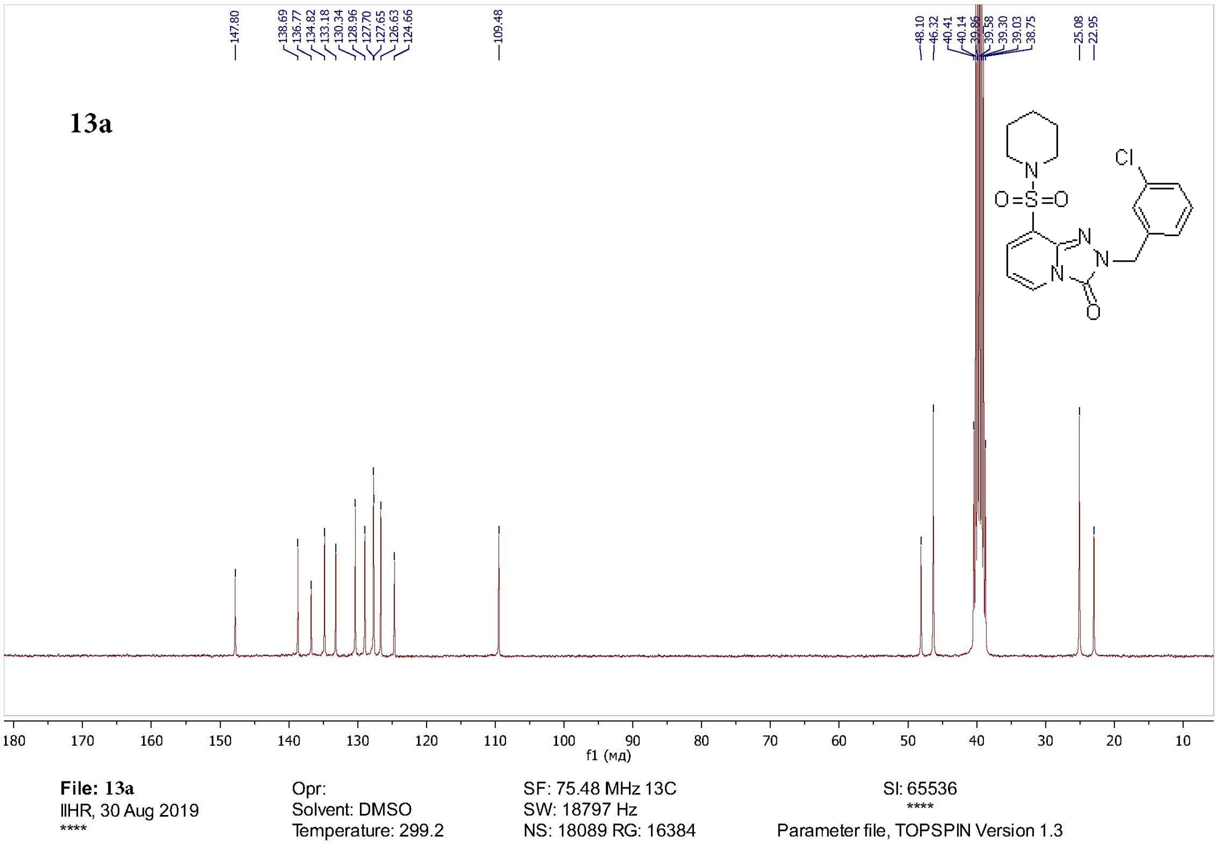

Figure S69. ^{13}C NMR spectrum (75 MHz, DMSO-d6) of 2-(3-chlorobenzyl)-8-(piperidin-1-ylsulfonyl)-[1,2,4]triazolo[4,3-a]pyridin-3(2H)-one **13a**.

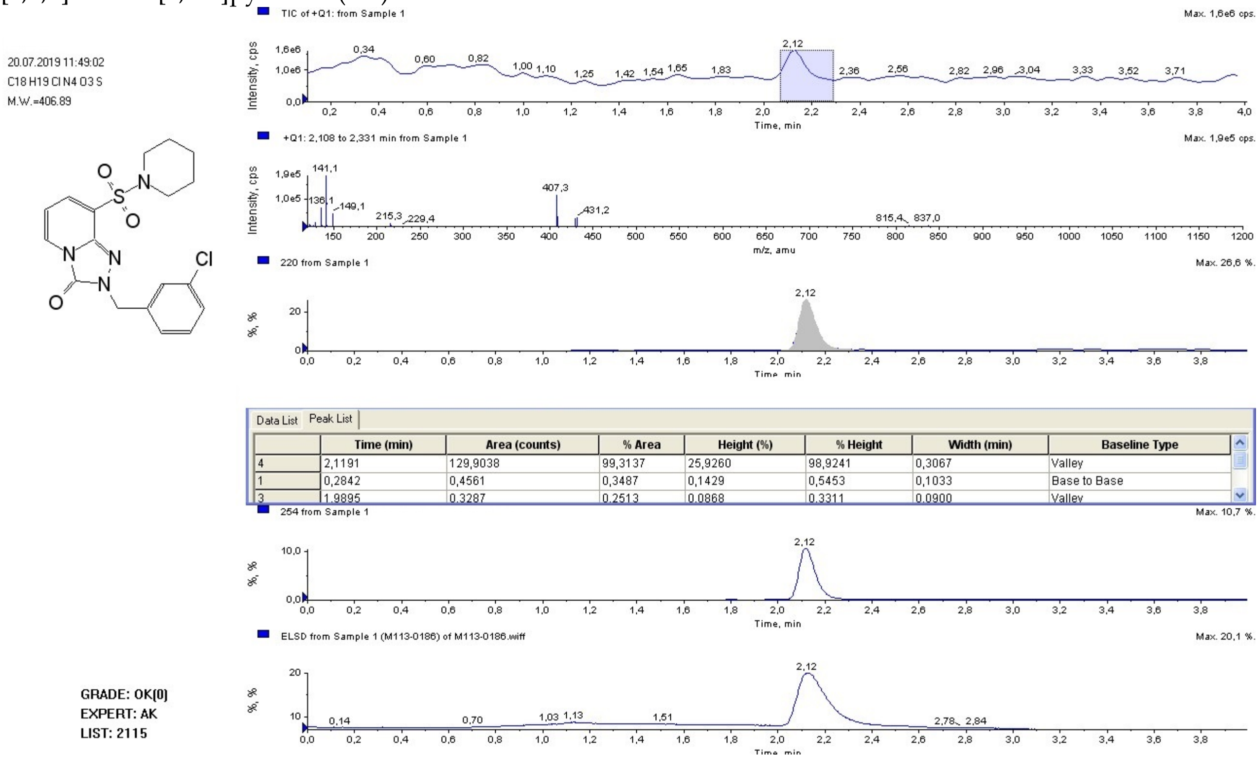

Figure S70. LC/MS data for 2-(3-chlorobenzyl)-8-(piperidin-1-ylsulfonyl)-[1,2,4]triazolo[4,3-a]pyridin-3(2H)-one **13a**.

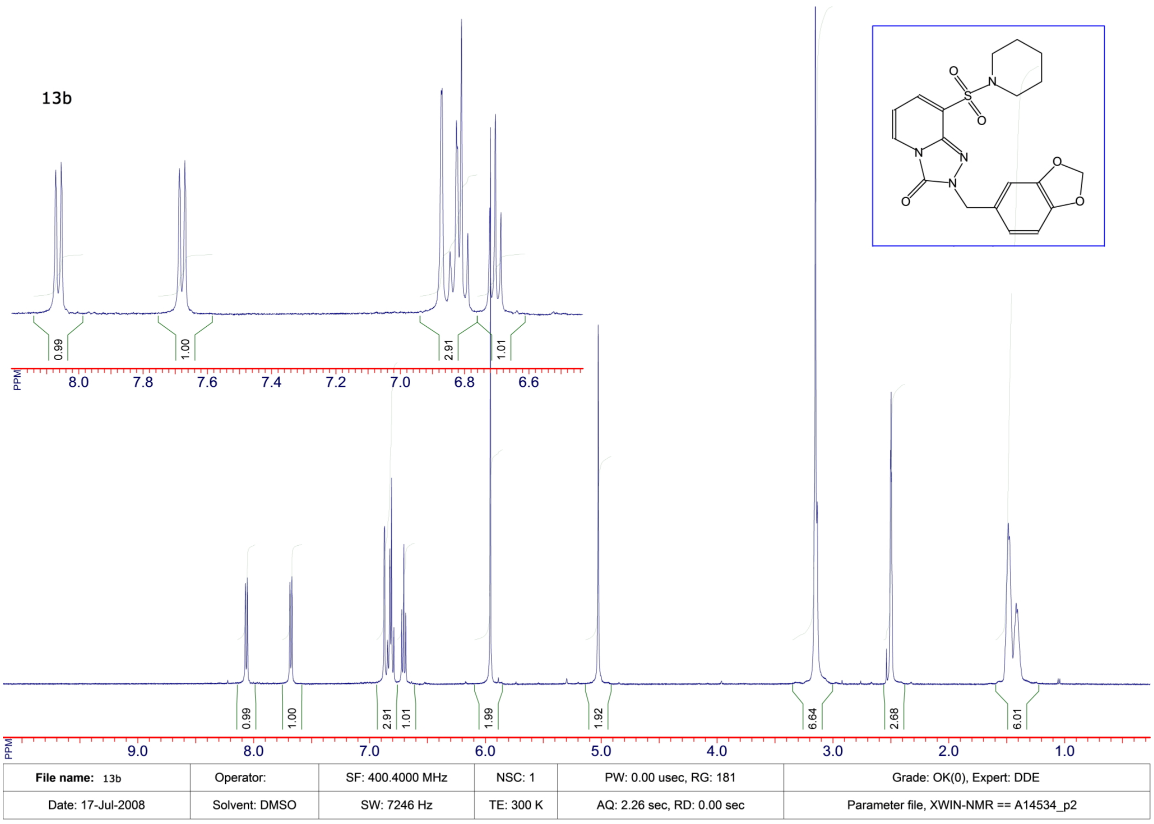

Figure S71. ^{1}H NMR spectrum (400 MHz, DMSO-d6) of 2-(benzo[d][1,3]dioxol-5-ylmethyl)-8-(piperidin-1-ylsulfonyl)-[1,2,4]triazolo[4,3-a]pyridin-3(2H)-one **13b**.

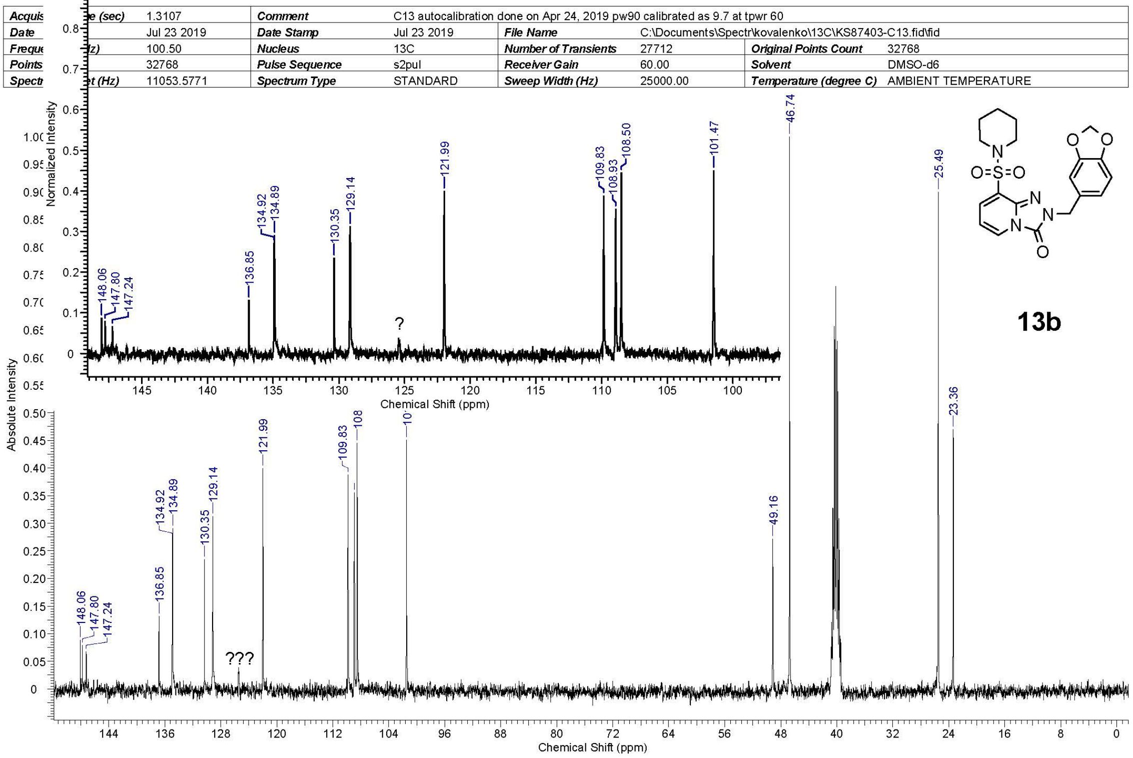

Figure S72. ^{13}C NMR spectrum (100 MHz, DMSO-d6) of 2-(benzo[d][1,3]dioxol-5-ylmethyl)-8-(piperidin-1-ylsulfonyl)-[1,2,4]triazolo[4,3-a]pyridin-3(2H)-one **13b**.

X:\2010\10_10\101015\M6839\34063623.D Sample Name: 13b Page 1 of 1

-.o.-Syntez Purity Report -.o.-

Agilent 1100 LC/MSD SL
Diodearrey G1315B (DAD1A-215nm; DAD1B-254nm)
Mass Quad G1956B (MSD1-Pos, MSD2-Neg)
ELSD Altech 3300 (ADC1 A, ELSD)

Mobile Phase:A-H2O+0.1%HCOOH;B-MeCN+0.1HCOOH
Separation column:
Rapid Resolutionn HT Cartige 4.6x30mm,
1.8-Micron, Zorbx SB-C18

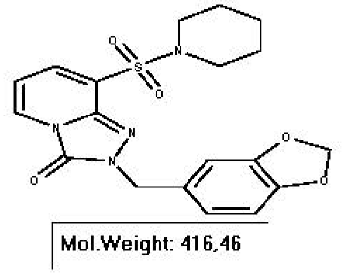

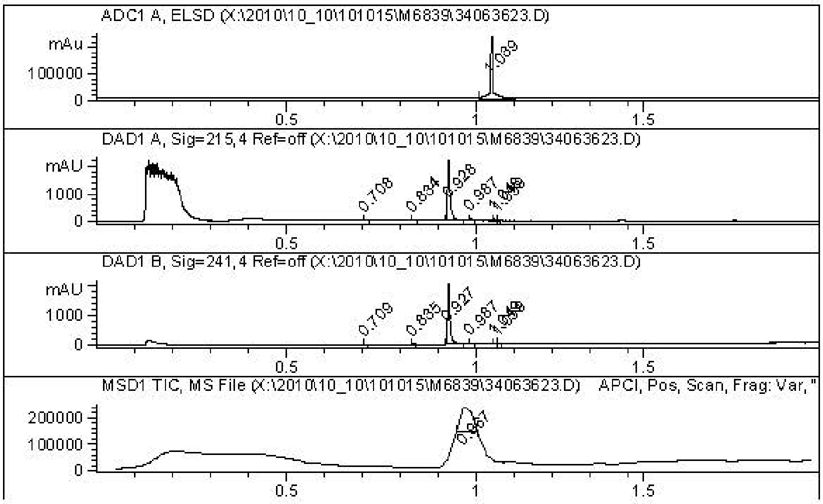

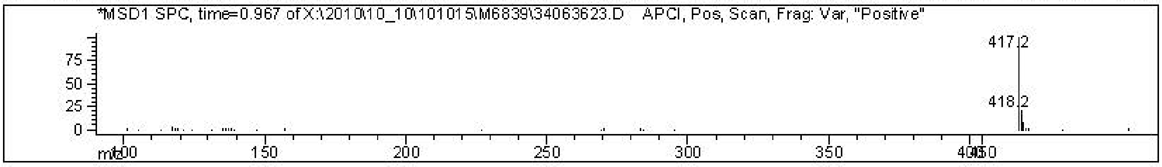

#	Signal	R.Time	Area %
1	ADC1 A, ELSD	1.039	100.000

#	Signal	R.Time	Area %
1	DAD1 A, Sig=215,4 Ref=off	0.708	0.839
2		0.834	0.495
3		0.928	94.038
4		0.987	1.715
5		1.048	2.056
6		1.059	0.857

#	Signal	R.Time	Area %
1	DAD1 B, Sig=241,4 Ref=off	0.709	0.404
2		0.835	0.390
3		0.927	95.723
4		0.987	1.516
5		1.049	1.278
6		1.059	0.689

#	Signal	R.Time	Area %
1	MSD1 TIC, MS File	0.967	100.000

Figure S73. LC/MS data for 2-(benzo[d][1,3]dioxol-5-ylmethyl)-8-(piperidin-1-ylsulfonyl)-[1,2,4]triazolo[4,3-a]pyridin-3(2H)-one **13b**.

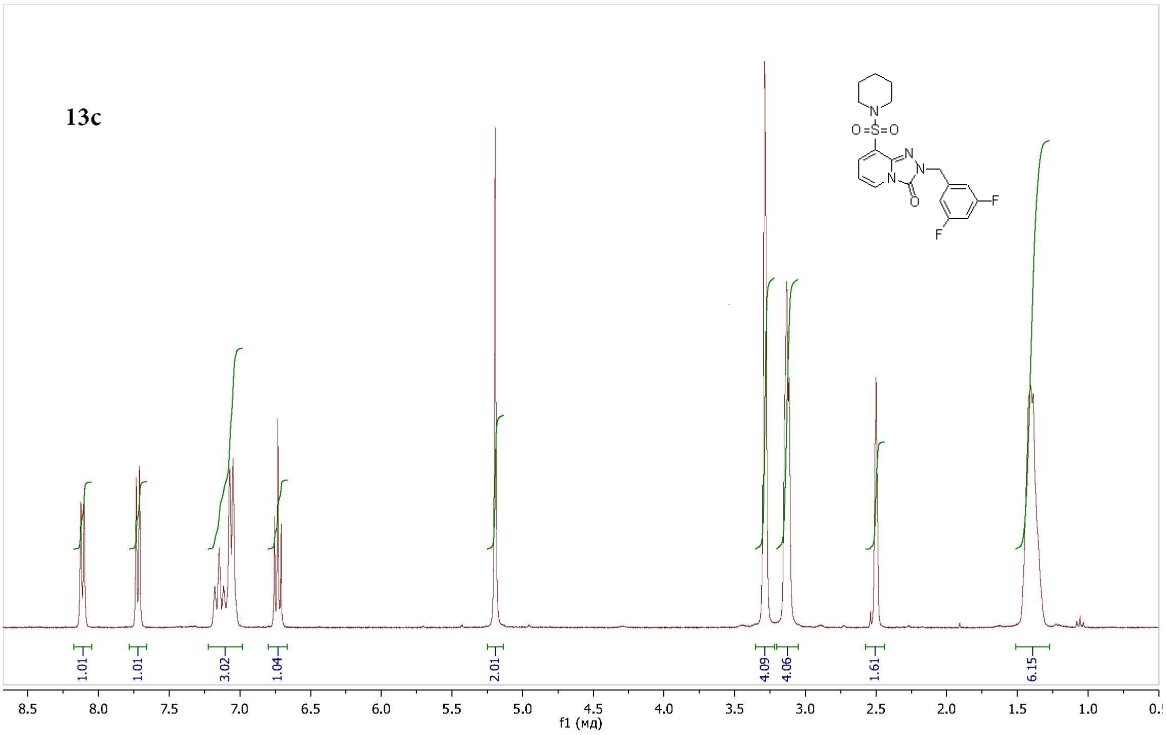

Figure S74. ^{1}H NMR spectrum (300 MHz, DMSO-d6) of 2-(3,5-difluorobenzyl)-8-(piperidin-1-ylsulfonyl)-[1,2,4]triazolo[4,3-a]pyridin-3(2H)-one **13c**.

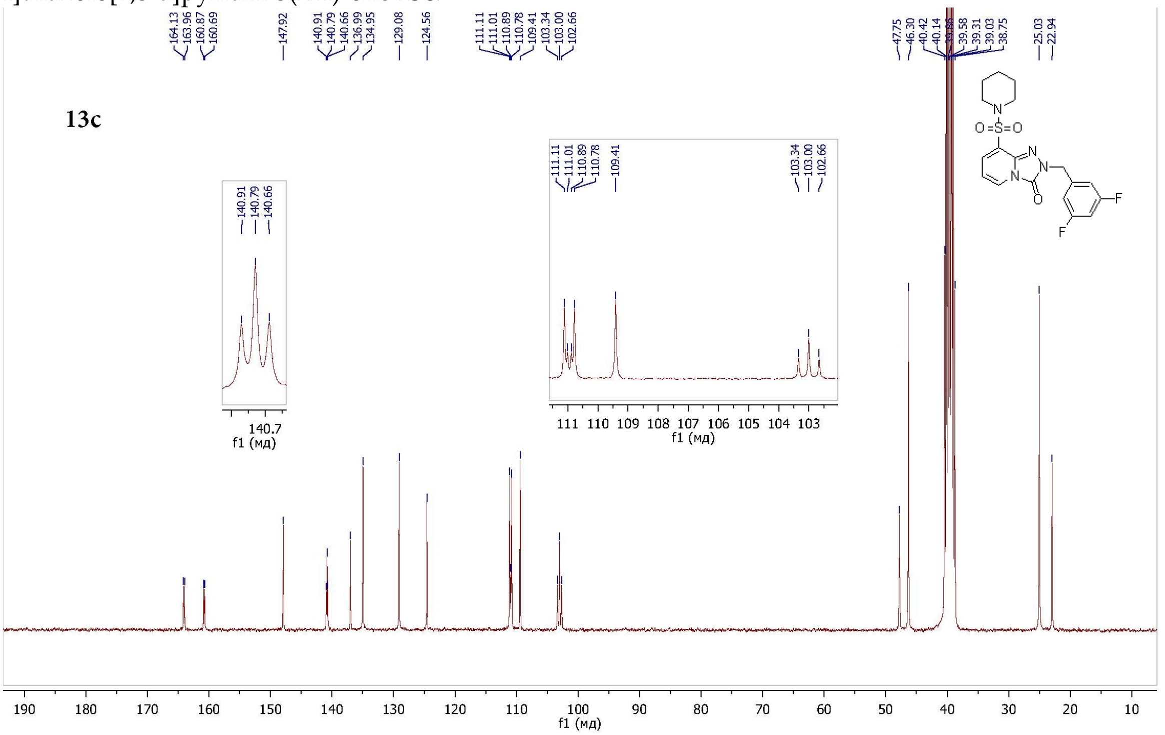

Figure S75. ^{13}C NMR spectrum (75 MHz, DMSO-d6) of 2-(3,5-difluorobenzyl)-8-(piperidin-1-ylsulfonyl)-[1,2,4]triazolo[4,3-a]pyridin-3(2H)-one **13c**.

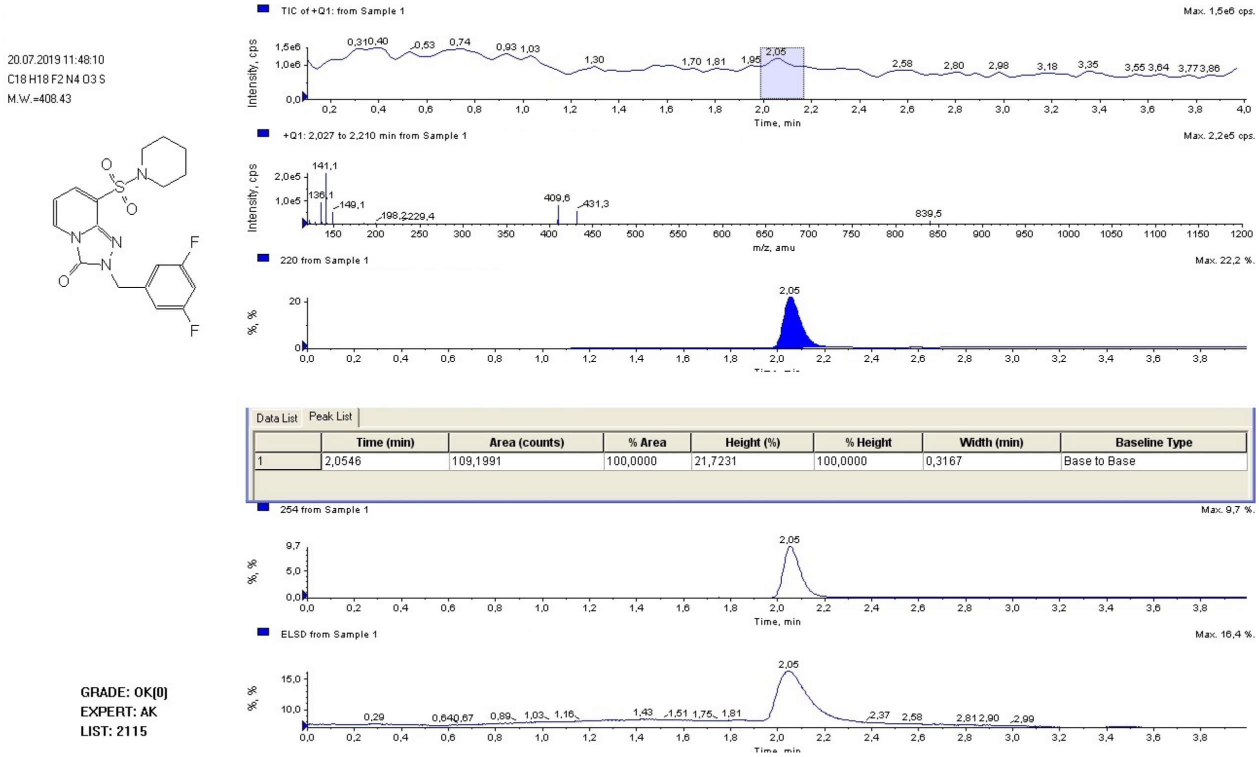

Figure S76. LC/MS data for 2-(3,5-difluorobenzyl)-8-(piperidin-1-ylsulfonyl)-[1,2,4]triazolo[4,3-a]pyridin-3(2H)-one **13c**.

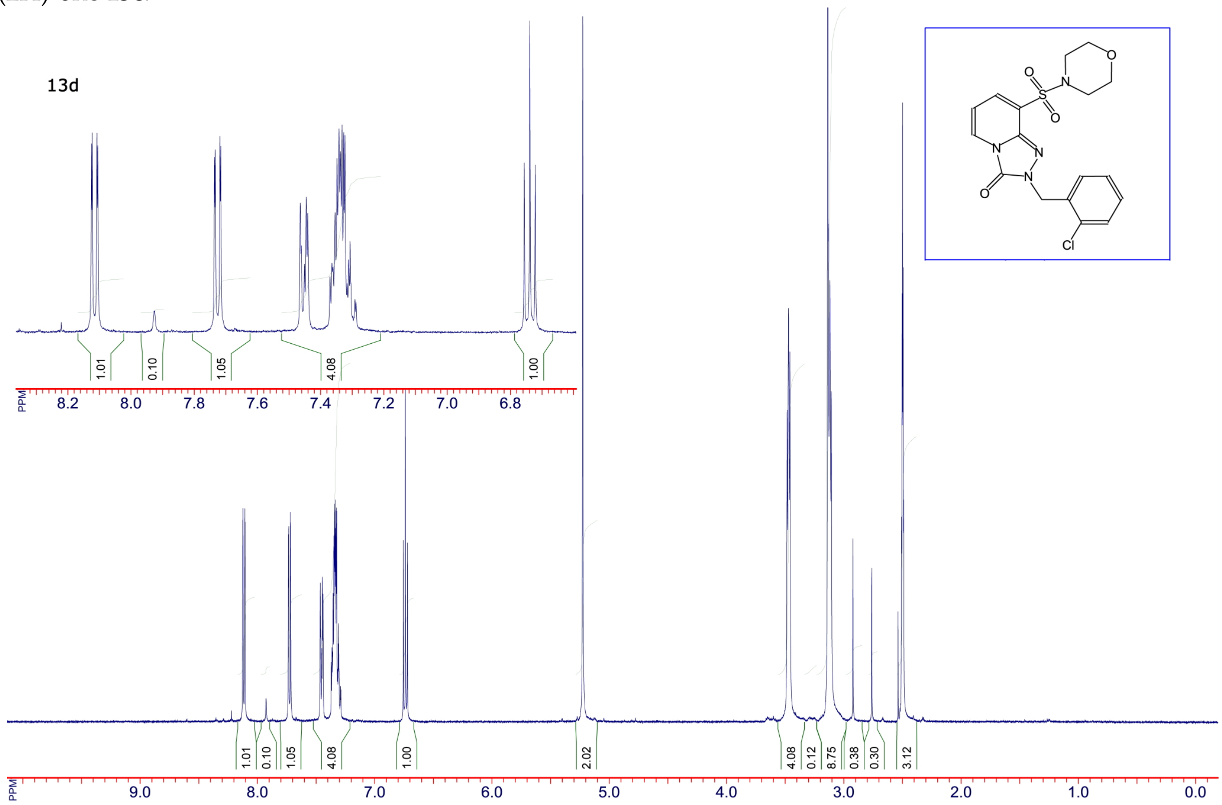

File name: 13d	Operator:	SF: 400.4000 MHz	NSC: 1	PW: 0.00 usec, RG: 181	Grade: OK(0), Expert: DDE
Date: 17-Jul-2008	Solvent: DMSO	SW: 7246 Hz	TE: 300 K	AQ: 2.26 sec, RD: 0.00 sec	Parameter file, XWIN-NMR == A14534_p2

Figure S77. ^{1}H NMR spectrum (400 MHz, DMSO-d6) of 2-(2-chlorobenzyl)-8-(morpholinosulfonyl)-[1,2,4]triazolo[4,3-a]pyridin-3(2H)-one **13d**.

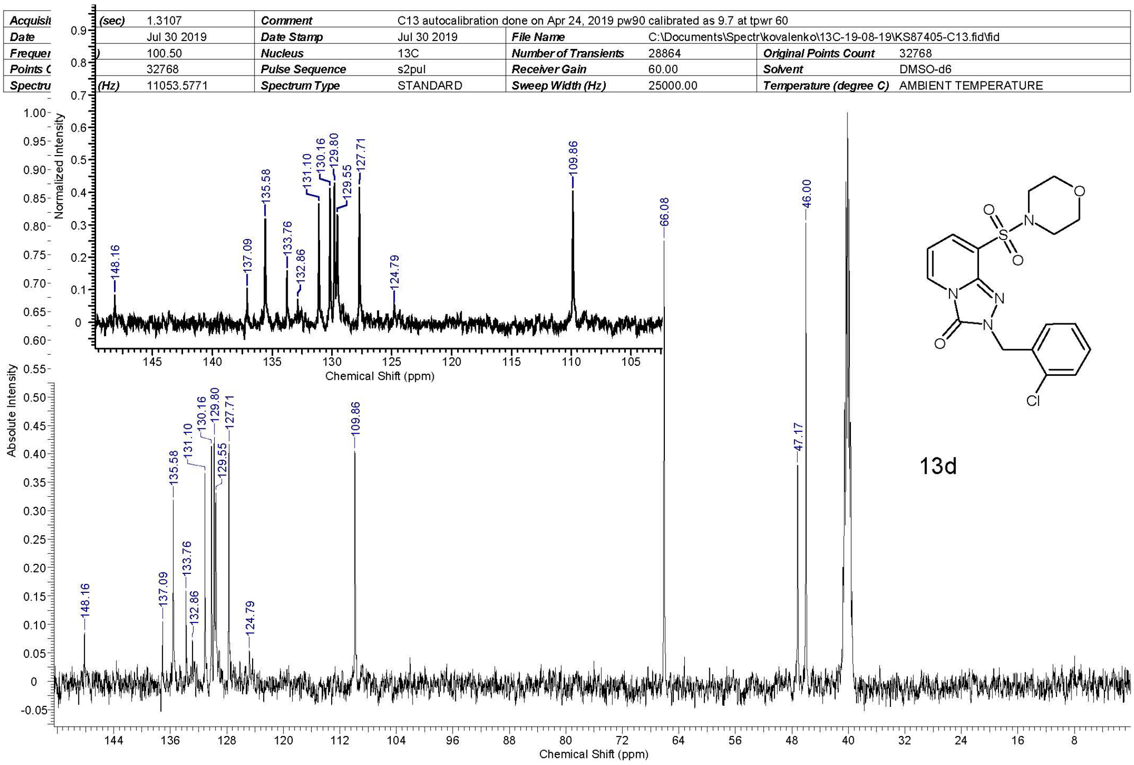

Figure S78. ^{13}C NMR spectrum (100 MHz, DMSO-d6) of 2-(2-chlorobenzyl)-8-(morpholinosulfonyl)-[1,2,4]triazolo[4,3-a]pyridin-3(2H)-one **13d**.

```
X:\2010\10_10\101015\M6839\34063645.D          Sample Name: 13d              Page 1 of 1
-------------------------------------------------------------------------------------------
                            -.o.-Syntez Purity Report -.o.-
Agilent 1100 LC/MSD SL                          Mobile Phase:A-H2O+0.1%HCOOH;B-MeCN+0.1HCOOH
Diodearrey G1315B (DAD1A-215nm; DAD1B-254nm)    Separation column:
Mass Quad  G1956B (MSD1-Pos,   MSD2-Neg)        Rapid Resolutionn HT Cartige 4.6x30mm,
ELSD Altech 3300  (ADC1 A, ELSD)                1.8-Micron, Zorbx SB-C18
-------------------------------------------------------------------------------------------
```

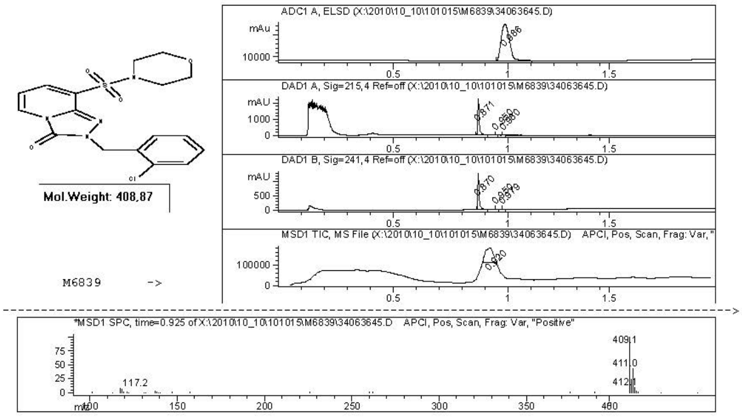

#	Signal	R.Time	Area %
1	ADC1 A, ELSD	0.986	100.000

#	Signal	R.Time	Area %
1	DAD1 A, Sig=215,4 Ref=off	0.871	98.288
2		0.950	0.765
3		0.980	0.947

#	Signal	R.Time	Area %
1	DAD1 B, Sig=241,4 Ref=off	0.870	97.590
2		0.950	1.531
3		0.979	0.879

#	Signal	R.Time	Area %
1	MSD1 TIC, MS File	0.920	100.000

Figure S79. LC/MS data for 2-(2-chlorobenzyl)-8-(morpholinosulfonyl)-[1,2,4]triazolo[4,3-a]pyridin-3(2H)-one **13d**.

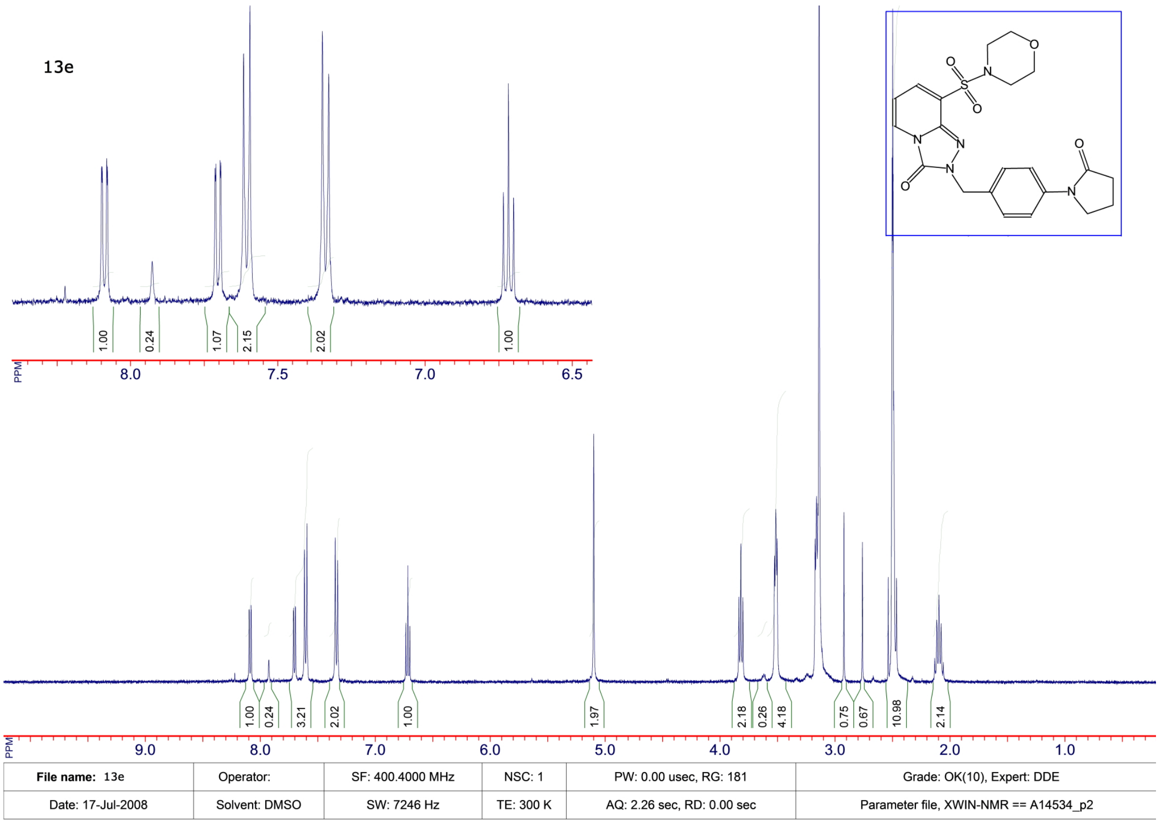

Figure S80. ^{1}H NMR spectrum (400 MHz, DMSO-d6) of 8-(morpholinosulfonyl)-2-[4-(2-oxopyrrolidin-1-yl)benzyl]-[1,2,4]triazolo[4,3-a]pyridin-3(2H)-one **13e**.

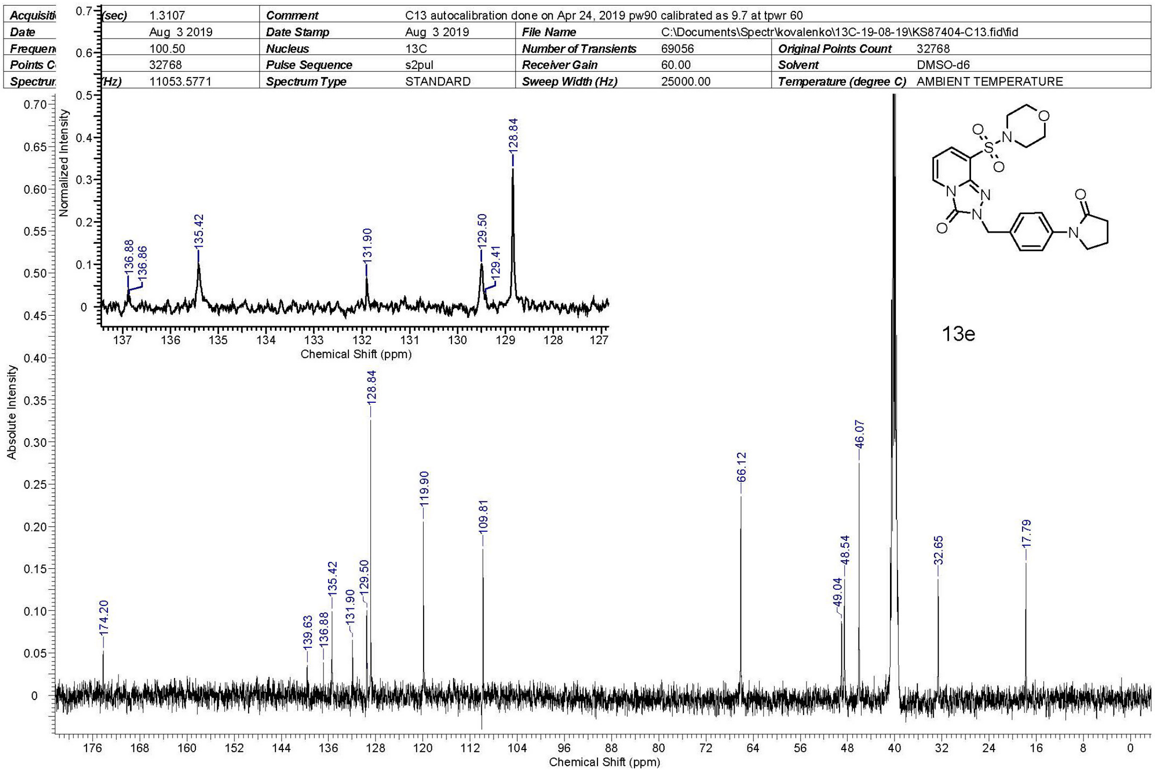

Figure S81. ^{13}C NMR spectrum (100 MHz, DMSO-d6) of 8-(morpholinosulfonyl)-2-[4-(2-oxopyrrolidin-1-yl)benzyl]-[1,2,4]triazolo[4,3-a]pyridin-3(2H)-one **13e**.

X:\2010\10_10\101015\M6839\34063644.D Sample Name: 13e Page 1 of 1

-.o.-Syntez Purity Report -.o.-

Agilent 1100 LC/MSD SL
Diodearrey G1315B (DAD1A-215nm; DAD1B-254nm)
Mass Quad G1956B (MSD1-Pos, MSD2-Neg)
ELSD Altech 3300 (ADC1 A, ELSD)

Mobile Phase:A-H2O+0.1%HCOOH;B-MeCN+0.1HCOOH
Separation column:
Rapid Resolutionn HT Cartige 4.6x30mm,
1.8-Micron, Zorbx SB-C18

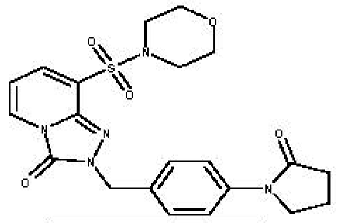

Mol.Weight: 457,51

M6839 ->

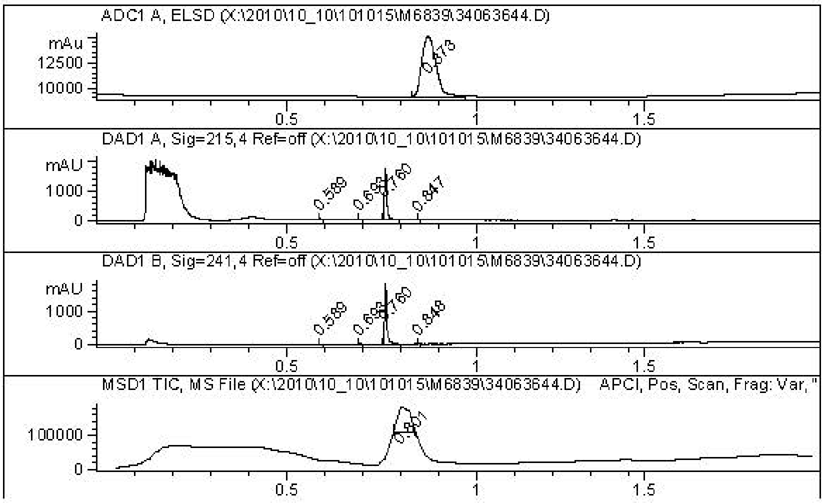

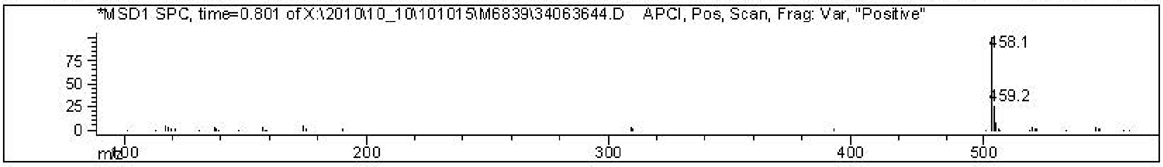

#	Signal	R.Time	Area %
1	ADC1 A, ELSD	0.873	100.000

#	Signal	R.Time	Area %
1	DAD1 A, Sig=215,4 Ref=off	0.589	1.990
2		0.693	1.011
3		0.760	96.705
4		0.847	0.293

#	Signal	R.Time	Area %
1	DAD1 B, Sig=241,4 Ref=off	0.589	0.537
2		0.693	1.114
3		0.760	98.030
4		0.848	0.319

#	Signal	R.Time	Area %
1	MSD1 TIC, MS File	0.801	100.000

Figure S82. LC/MS data for 8-(morpholinosulfonyl)-2-[4-(2-oxopyrrolidin-1-yl)benzyl]-[1,2,4]triazolo[4,3-a]pyridin-3(2H)-one **13e**.

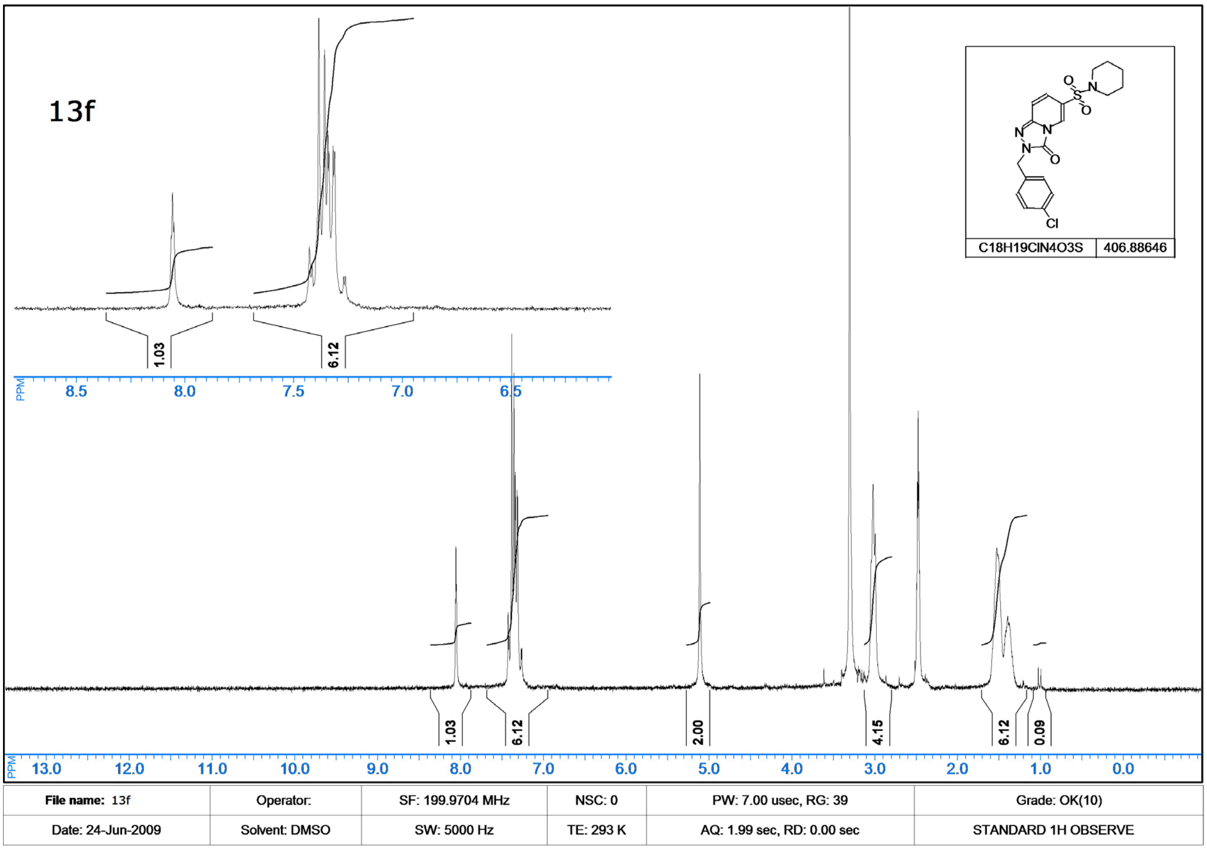

Figure S83. ^{1}H NMR spectrum (200 MHz, DMSO-d6) of 2-(4-chlorobenzyl)-6-(piperidin-1-ylsulfonyl)-[1,2,4]triazolo[4,3-a]pyridin-3(2H)-one **13f**.

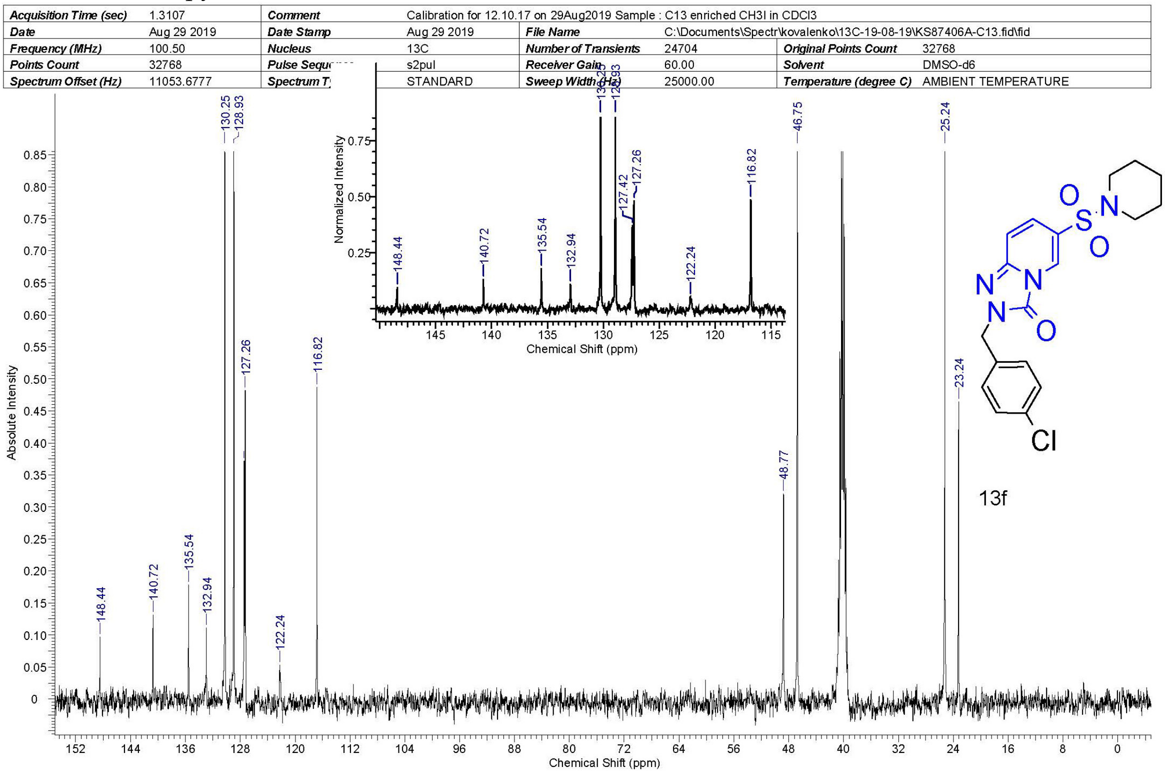

Figure S84. ^{13}C NMR spectrum (100 MHz, DMSO-d6) of 2-(4-chlorobenzyl)-6-(piperidin-1-ylsulfonyl)-[1,2,4]triazolo[4,3-a]pyridin-3(2H)-one **13f**.

-.o.-Syntez Purity Report -.o.-

Agilent 1100 LC/MSD SL
Diodearrey G1315B (DAD1A-215nm; DAD1B-254nm)
Mass Quad G1956B (MSD1-Pos, MSD2-Neg)
ELSD Altech 3300 (ADC1 A, ELSD)

Mobile Phase:A-H2O+0.1%HCOOH;B-MeCN+0.1HCOOH
Separation column:
Rapid Resolutionn HT Cartige 4.6x30mm,
1.8-Micron, Zorbx SB-C18

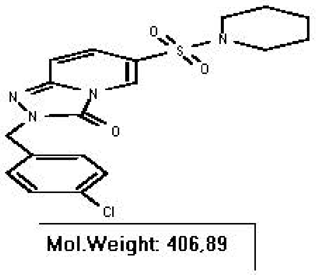

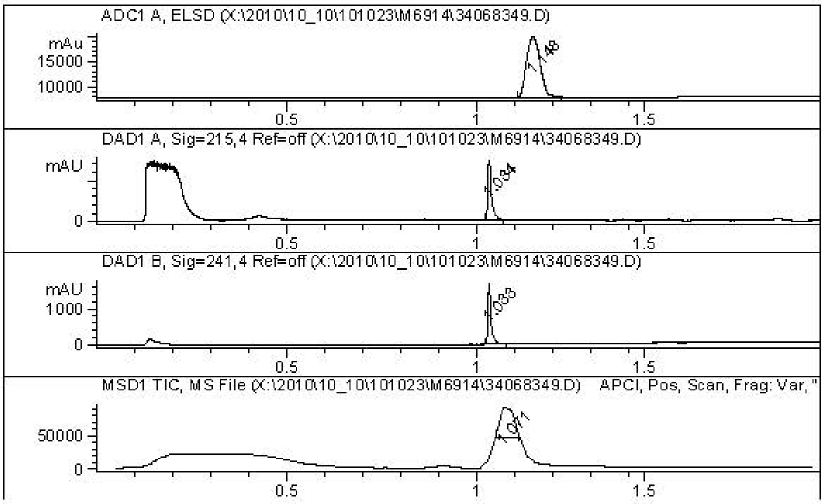

M6914 ->

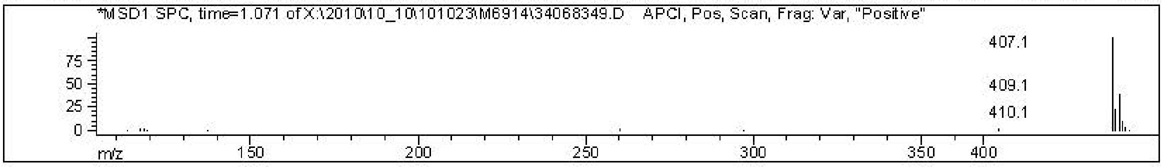

#	Signal	R.Time	Area %
1	ADC1 A, ELSD	1.148	100.000

#	Signal	R.Time	Area %
1	DAD1 A, Sig=215,4 Ref=off	1.034	100.000

#	Signal	R.Time	Area %
1	DAD1 B, Sig=241,4 Ref=off	1.033	100.000

#	Signal	R.Time	Area %
1	MSD1 TIC, MS File	1.071	100.000

Figure S85. LC/MS data for 2-(4-chlorobenzyl)-6-(piperidin-1-ylsulfonyl)-[1,2,4]triazolo[4,3-a]pyridin-3(2H)-one **13f**.

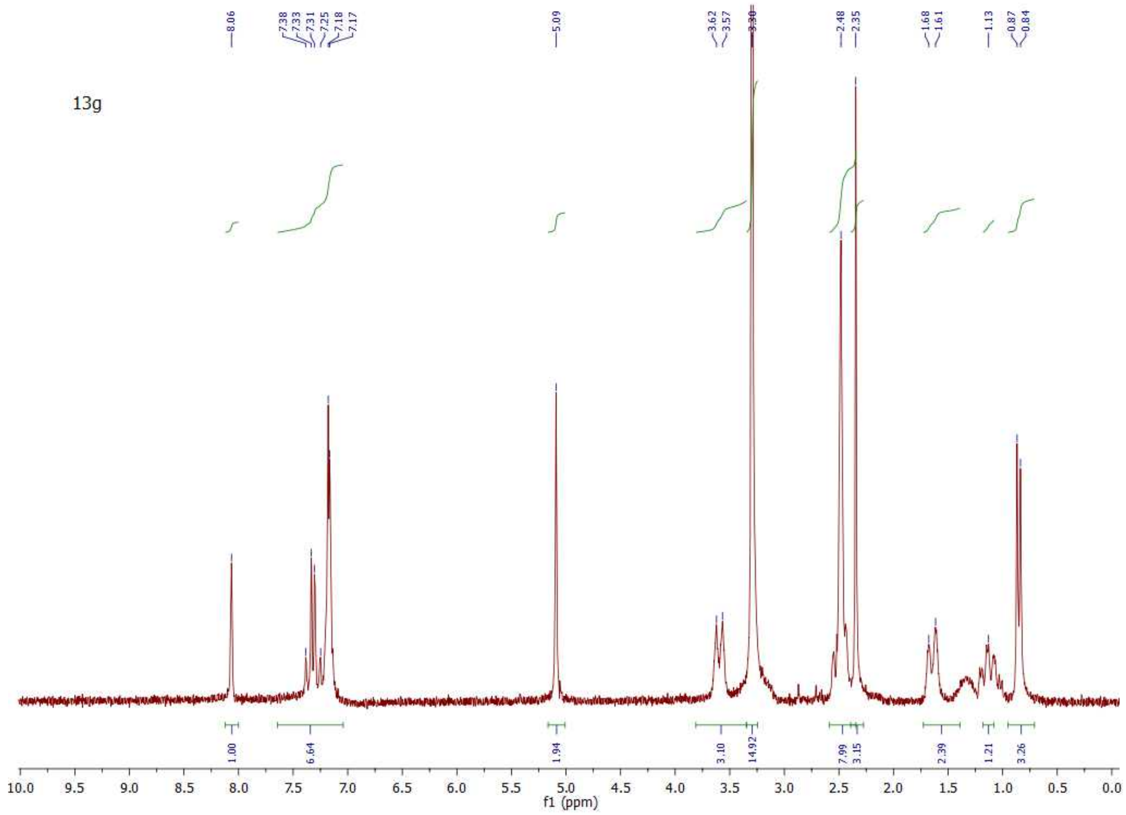

Figure S86. ^{1}H NMR spectrum (200 MHz, DMSO-d6) of 2-(3-methylbenzyl)-6-(4-methylpiperidin-1-ylsulfonyl)-[1,2,4]triazolo[4,3-a]pyridin-3(2H)-one **13g**.

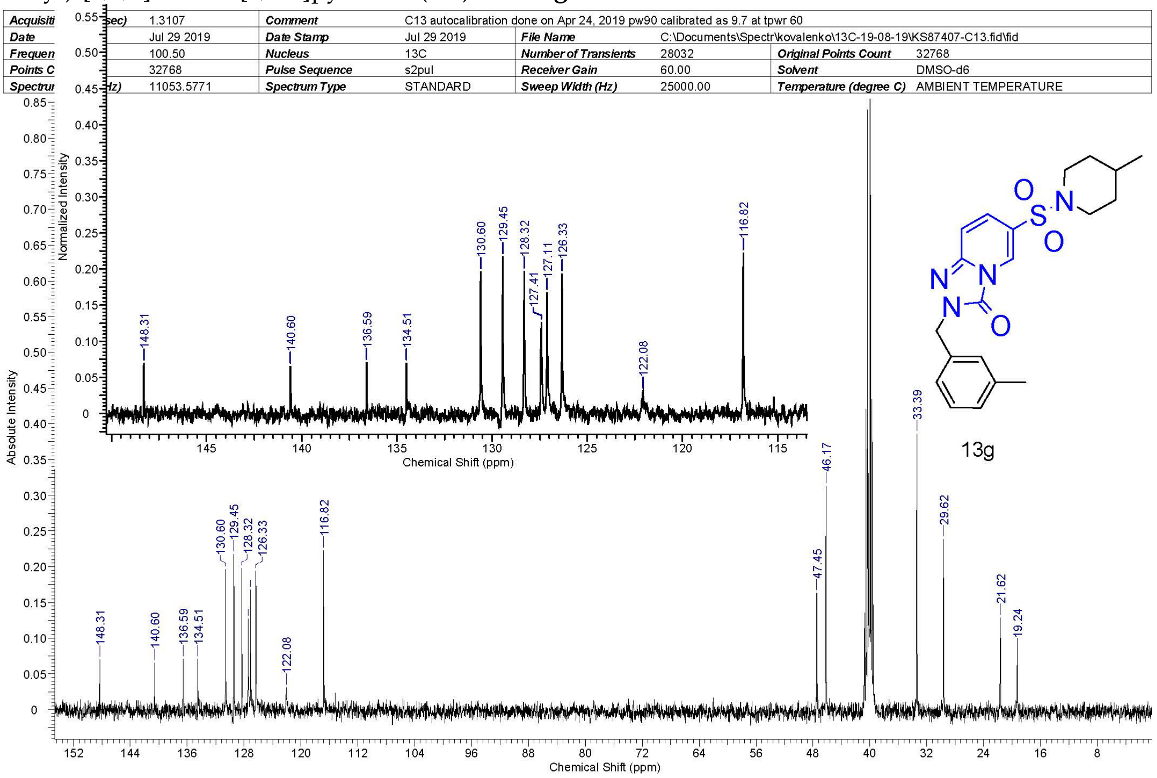

Figure S87. ^{13}C NMR spectrum (100 MHz, DMSO-d6) of 2-(3-methylbenzyl)-6-(4-methylpiperidin-1-ylsulfonyl)-[1,2,4]triazolo[4,3-a]pyridin-3(2H)-one **13g**.

X:\2010\10_10\101023\M6914\34068366.D   Sample Name: 13g   Page 1 of 1

-.o.-Syntez Purity Report -.o.-

Agilent 1100 LC/MSD SL
Diodearrey G1315B (DAD1A-215nm; DAD1B-254nm)
Mass Quad G1956B (MSD1-Pos, MSD2-Neg)
ELSD Altech 3300 (ADC1 A, ELSD)

Mobile Phase:A-H2O+0.1%HCOOH;B-MeCN+0.1HCOOH
Separation column:
Rapid Resolutionn HT Cartige 4.6x30mm,
1.8-Micron, Zorbx SB-C18

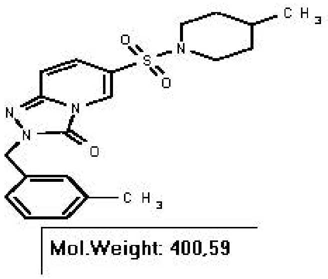

M6914 ->

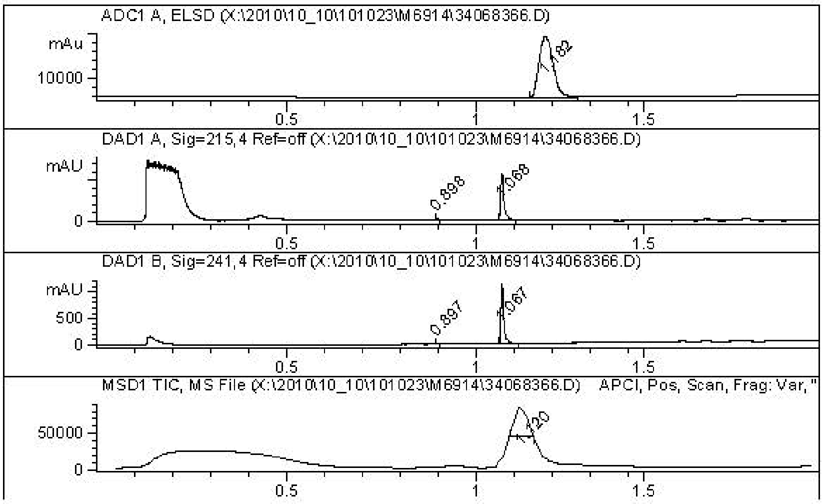

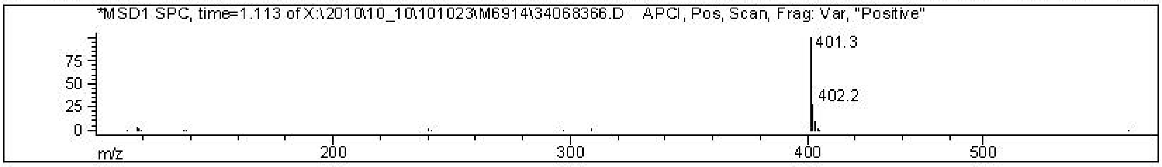

#	Signal	R.Time	Area %
1	ADC1 A, ELSD	1.182	100.000

#	Signal	R.Time	Area %
1	DAD1 A, Sig=215,4 Ref=off	0.898	1.457
2		1.068	98.543

#	Signal	R.Time	Area %
1	DAD1 B, Sig=241,4 Ref=off	0.897	0.761
2		1.067	99.239

#	Signal	R.Time	Area %
1	MSD1 TIC, MS File	1.120	100.000

Figure S88. LC/MS data for 2-(3-methylbenzyl)-6-(4-methylpiperidin-1-ylsulfonyl)-[1,2,4]triazolo[4,3-a]pyridin-3(2H)-one **13g**.

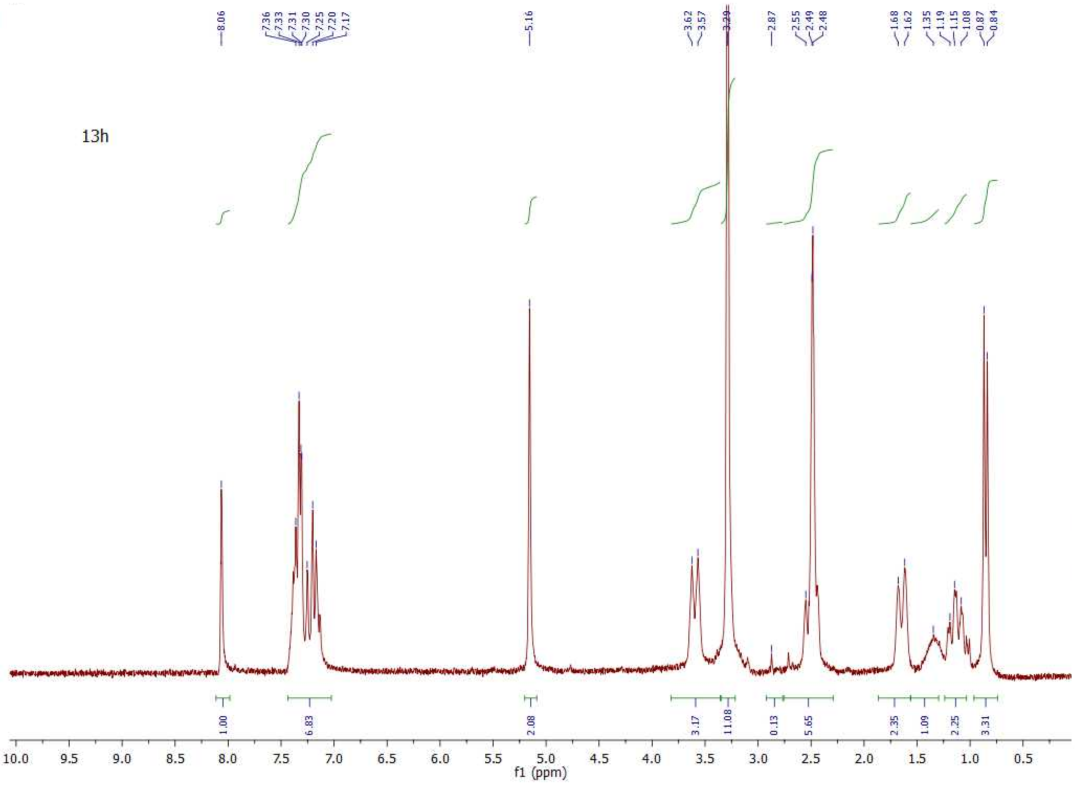

Figure S89. ^{1}H NMR spectrum (200 MHz, DMSO-d6) of 2-(2-fluorobenzyl)-6-(4-methylpiperidin-1-ylsulfonyl)-[1,2,4]triazolo[4,3-a]pyridin-3(2H)-one **13h**.

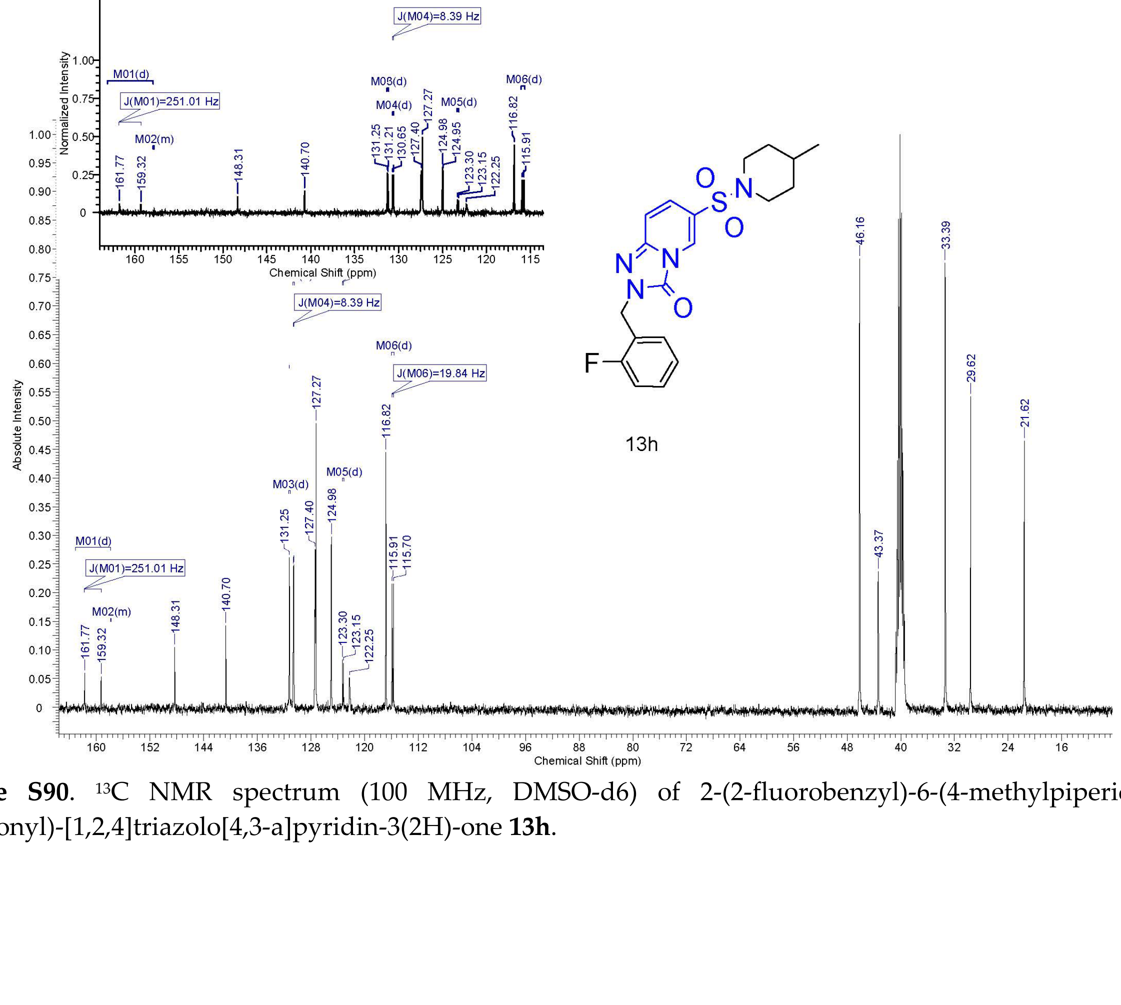

Figure S90. ^{13}C NMR spectrum (100 MHz, DMSO-d6) of 2-(2-fluorobenzyl)-6-(4-methylpiperidin-1-ylsulfonyl)-[1,2,4]triazolo[4,3-a]pyridin-3(2H)-one **13h**.

X:\2010\10_10\101023\M6914\34068371.D Sample Name: 13h Page 1 of

-.o.-Syntez Purity Report -.o.-

Agilent 1100 LC/MSD SL
Diodearrey G1315B (DAD1A-215nm; DAD1B-254nm)
Mass Quad G1956B (MSD1-Pos, MSD2-Neg)
ELSD Altech 3300 (ADC1 A, ELSD)

Mobile Phase:A-H2O+0.1%HCOOH;B-MeCN+0.1HCO
Separation column:
Rapid Resolutionn HT Cartige 4.6x30mm,
1.8-Micron, Zorbx SB-C18

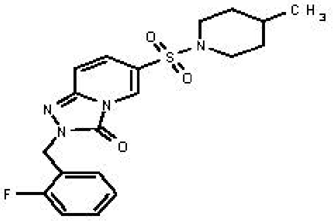

Mol.Weight: 404.47

M6914 ->

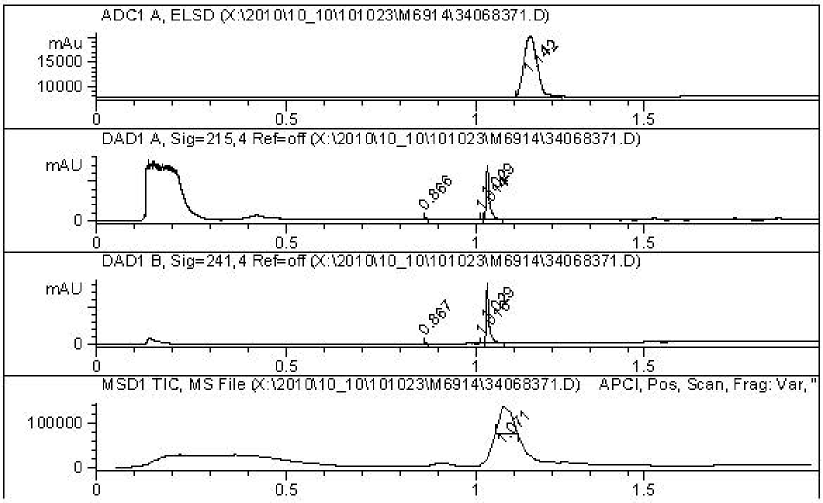

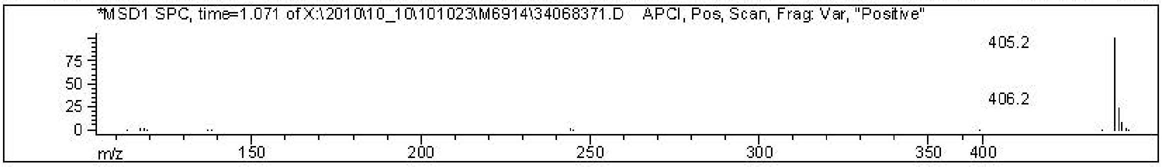

#	Signal	R.Time	Area %
1	ADC1 A, ELSD	1.142	100.000

#	Signal	R.Time	Area %
1	DAD1 A, Sig=215,4 Ref=off	0.866	1.103
2		1.014	0.597
3		1.029	98.300

#	Signal	R.Time	Area %
1	DAD1 B, Sig=241,4 Ref=off	0.867	0.713
2		1.016	0.493
3		1.029	98.793

#	Signal	R.Time	Area %
1	MSD1 TIC, MS File	1.071	100.000

Figure S91. LC/MS data for 2-(2-fluorobenzyl)-6-(4-methylpiperidin-1-ylsulfonyl)-[1,2,4]triazolo[4,3-a]pyridin-3(2H)-one **13h**.

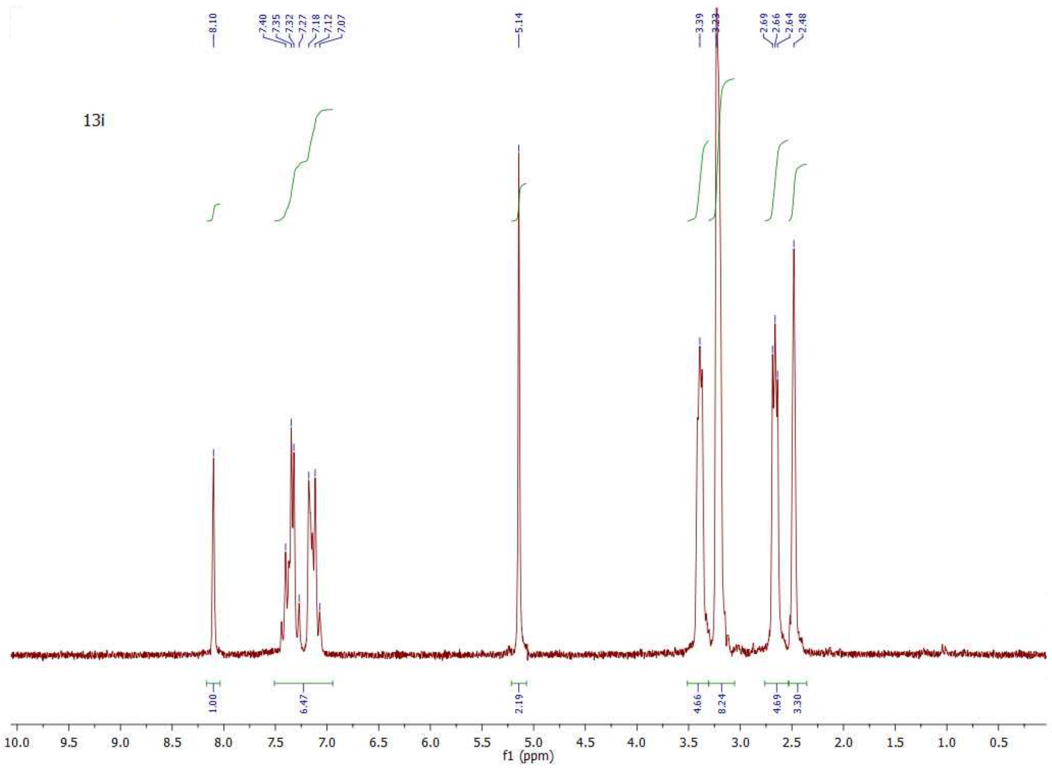

Figure S92. ^{1}H NMR spectrum (200 MHz, DMSO-d6) of 2-(3-fluorobenzyl)-6-(thiomorpholinosulfonyl)-[1,2,4]triazolo[4,3-a]pyridin-3(2H)-one **13i**.

Acquisition Time (sec)	1.3107	Comment	C13 autocalibration done on Apr 24, 2019 pw90 calibrated as 9.7 at tpwr 60				
Date	Jul 24 2019	Date Stamp	Jul 24 2019	File Name			
Frequency (MHz)	100.50	Nucleus	13C	Number of Transients	23744	Original Points Count	32768
Points Count	32768	Pulse Sequence	s2pul	Receiver Gain	60.00	Solvent	DMSO-d6
Spectrum Offset (Hz)	11053.5771	Spectrum Type	STANDARD	Sweep Width (Hz)	25000.00	Temperature (degree C)	AMBIENT TEMPERATURE

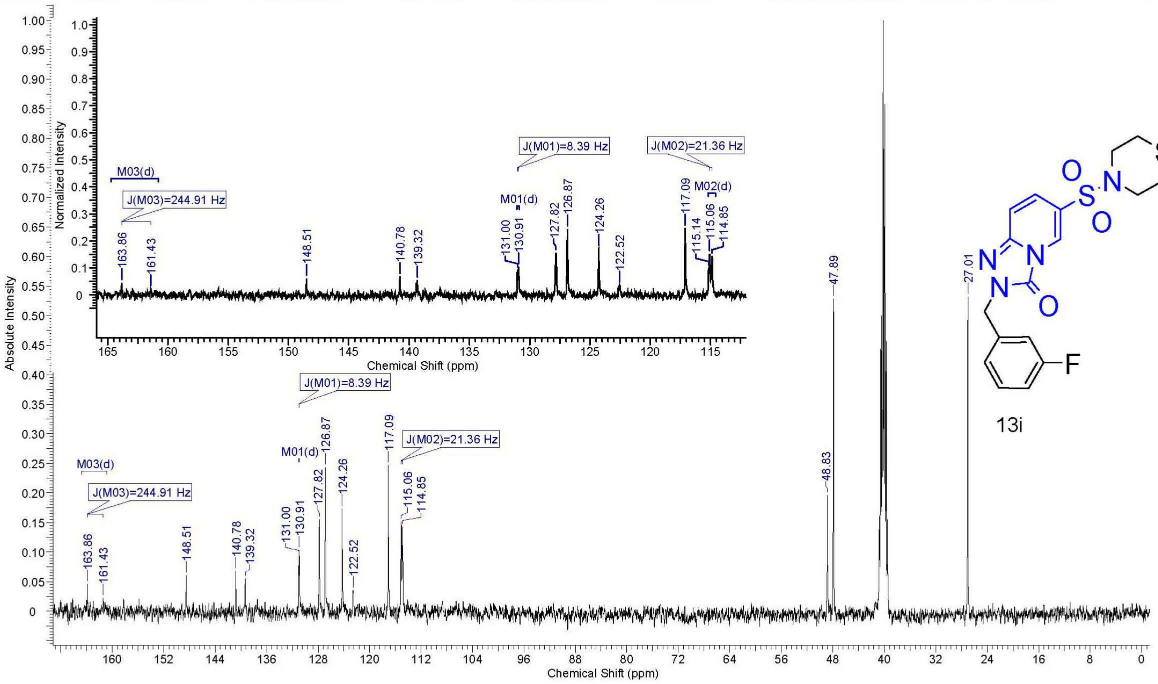

Figure S93. ^{13}C NMR spectrum (100 MHz, DMSO-d6) of 2-(3-fluorobenzyl)-6-(thiomorpholinosulfonyl)-[1,2,4]triazolo[4,3-a]pyridin-3(2H)-one **13i**.

X:\2010\10_10\101023\M6919\34068431.D Sample Name: 13i Page 1 of 1

-.o.-Syntez Purity Report -.o.-

Agilent 1100 LC/MSD SL
Diodearrey G1315B (DAD1A-215nm; DAD1B-254nm)
Mass Quad G1956B (MSD1-Pos, MSD2-Neg)
ELSD Altech 3300 (ADC1 A, ELSD)

Mobile Phase:A-H2O+0.1%HCOOH;B-MeCN+0.1HCOOH
Separation column:
Rapid Resolutionn HT Cartige 4.6x30mm,
1.8-Micron, Zorbx SB-C18

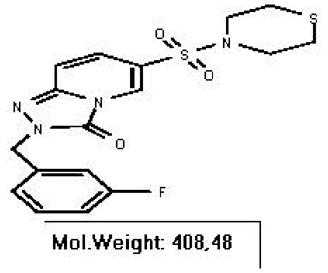

M6919 ->

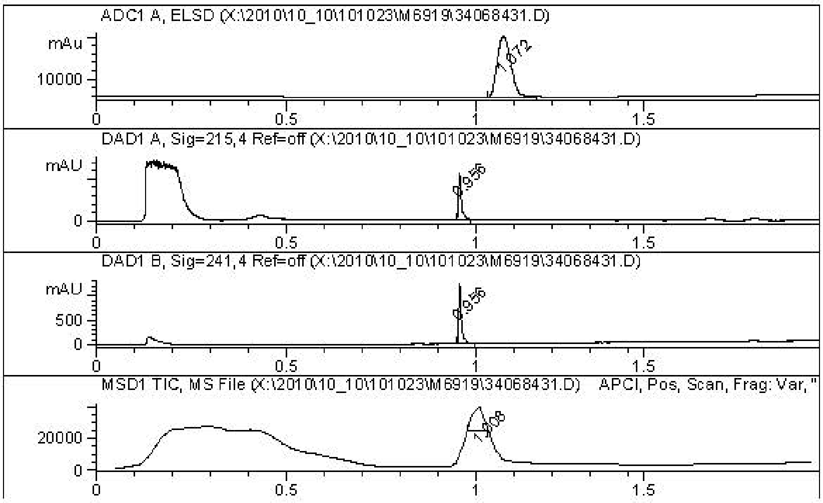

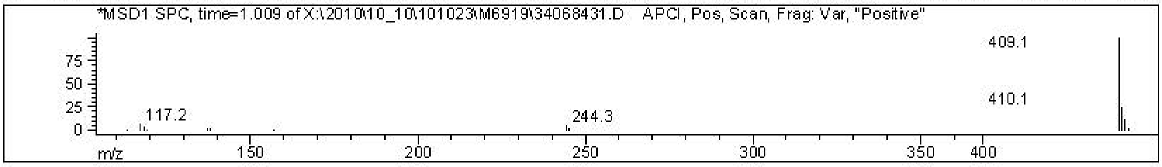

#	Signal	R.Time	Area %
1	ADC1 A, ELSD	1.072	100.000

#	Signal	R.Time	Area %
1	DAD1 A, Sig=215,4 Ref=off	0.956	100.000

#	Signal	R.Time	Area %
1	DAD1 B, Sig=241,4 Ref=off	0.956	100.000

#	Signal	R.Time	Area %
1	MSD1 TIC, MS File	1.008	100.000

Figure S94. LC/MS data for 2-(3-fluorobenzyl)-6-(thiomorpholinosulfonyl)-[1,2,4]triazolo[4,3-a]pyridin-3(2H)-one **13i**.

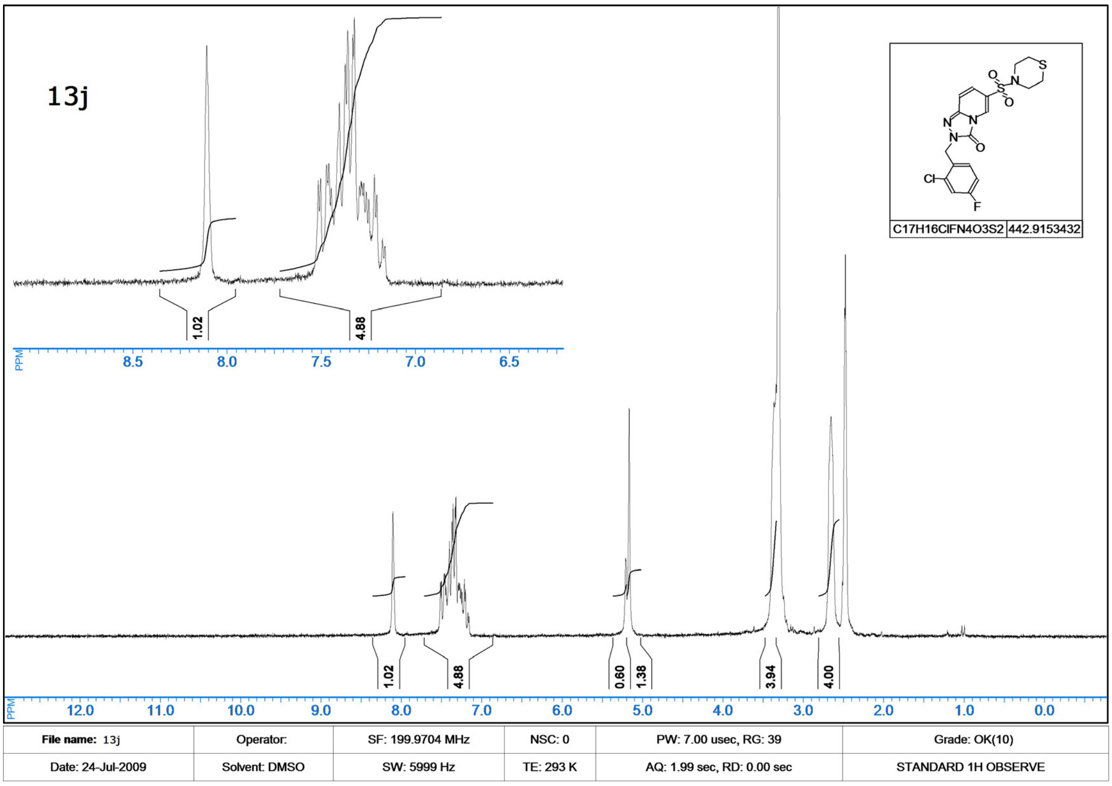

Figure S95. ^{1}H NMR spectrum (200 MHz, DMSO-d6) of 2-(2-chloro-4-fluorobenzyl)-6-(thiomorpholinosulfonyl)-[1,2,4]triazolo[4,3-a]pyridin-3(2H)-one **13j**.

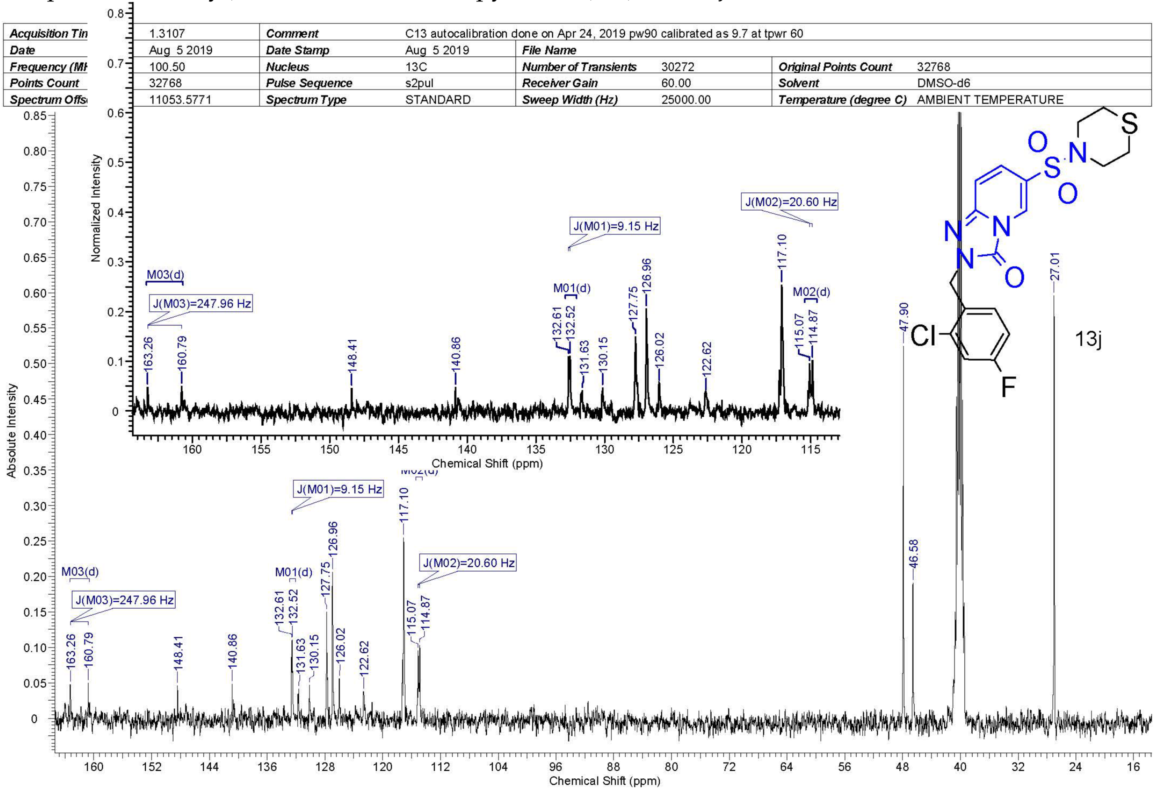

Figure S96. ^{13}C NMR spectrum (100 MHz, DMSO-d6) of 2-(2-chloro-4-fluorobenzyl)-6-(thiomorpholinosulfonyl)-[1,2,4]triazolo[4,3-a]pyridin-3(2H)-one **13j**.

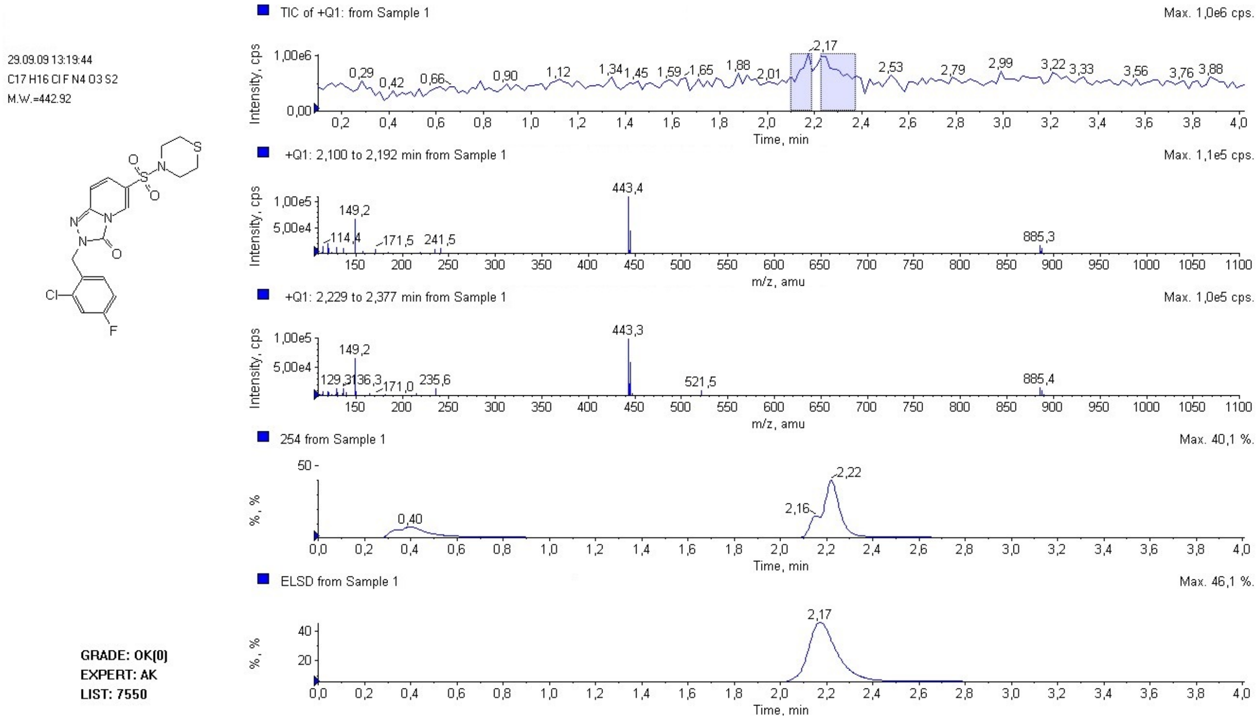

Figure S97. LC/MS data for 2-(2-chloro-4-fluorobenzyl)-6-(thiomorpholinosulfonyl)-[1,2,4]triazolo[4,3-a]pyridin-3(2H)-one **13j**.

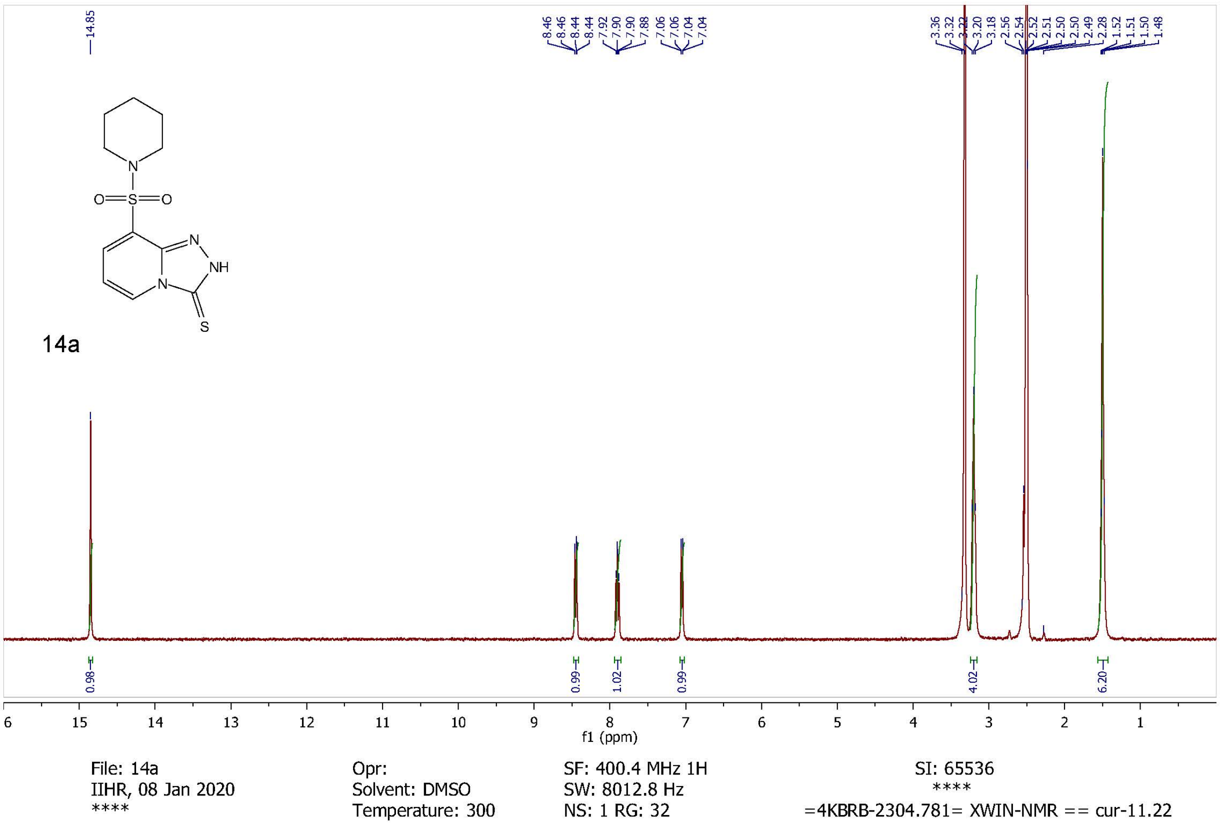

Figure S98. ^{1}H NMR spectrum (400 MHz, DMSO-d6) of 8-(piperidin-1-ylsulfonyl)-[1,2,4]triazolo[4,3-a]pyridine-3(2H)-thione **14a**.

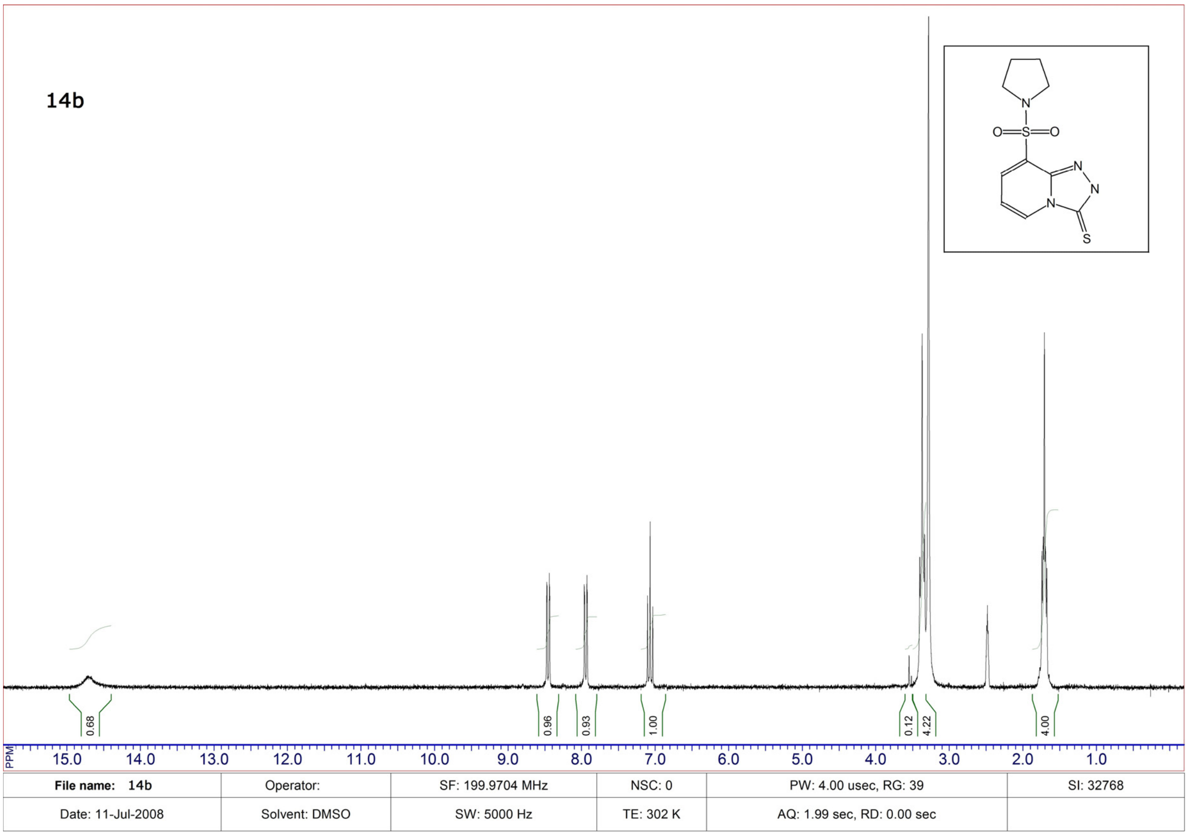

Figure S99. ^{1}H NMR spectrum (200 MHz, DMSO-d6) of 8-(pyrrolidin-1-ylsulfonyl)-[1,2,4]triazolo[4,3-a]pyridine-3(2H)-thione **14b**.

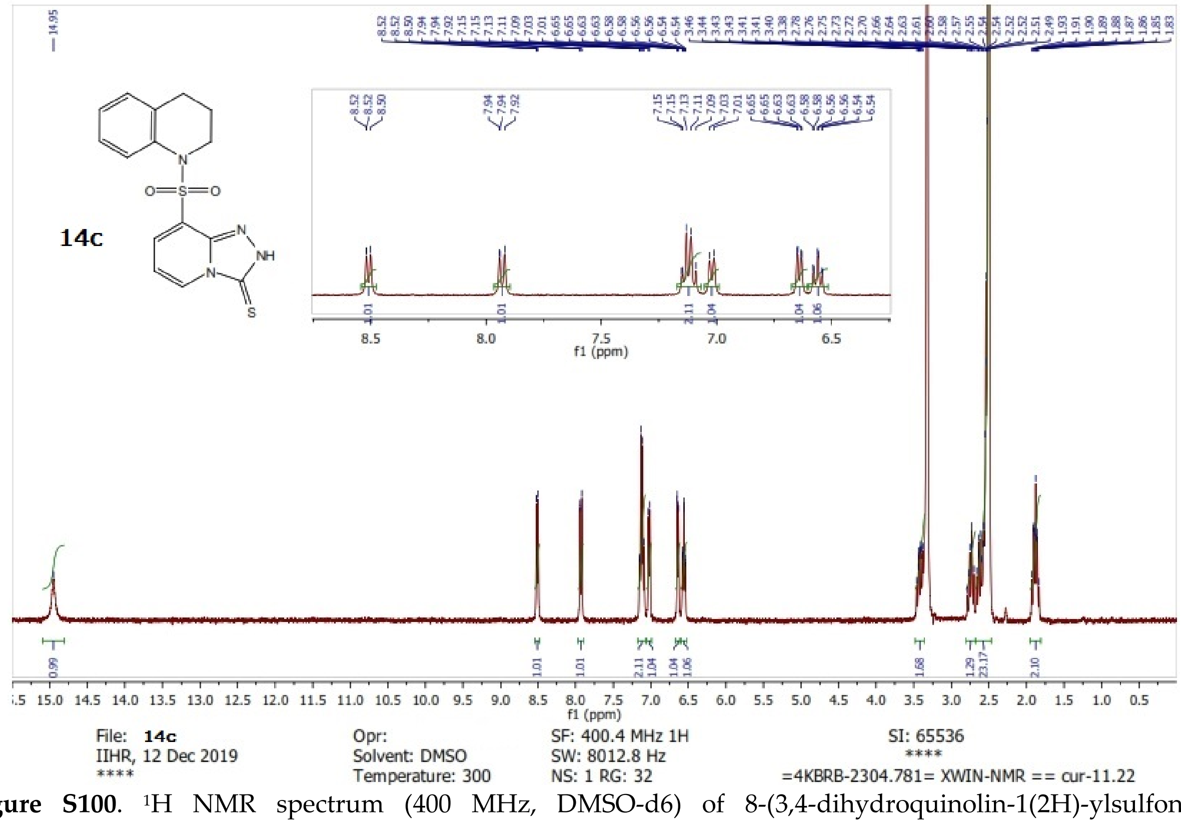

Figure S100. ^{1}H NMR spectrum (400 MHz, DMSO-d6) of 8-(3,4-dihydroquinolin-1(2H)-ylsulfonyl)-[1,2,4]triazolo[4,3-a]pyridine-3(2H)-thione **14c**.

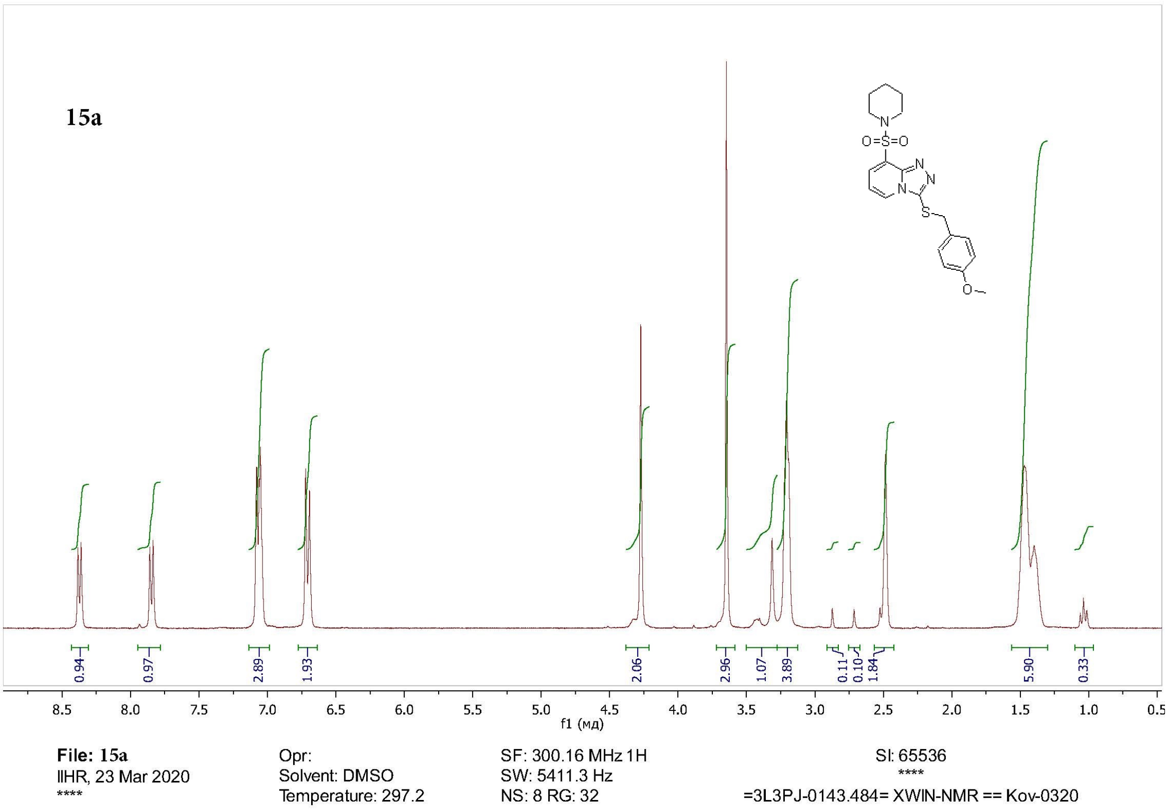

Figure S101. ^{1}H NMR spectrum (300 MHz, DMSO-d6) of 3-(4-methoxybenzylthio)-8-(piperidin-1-ylsulfonyl)-[1,2,4]triazolo[4,3-a]pyridine **15a**.

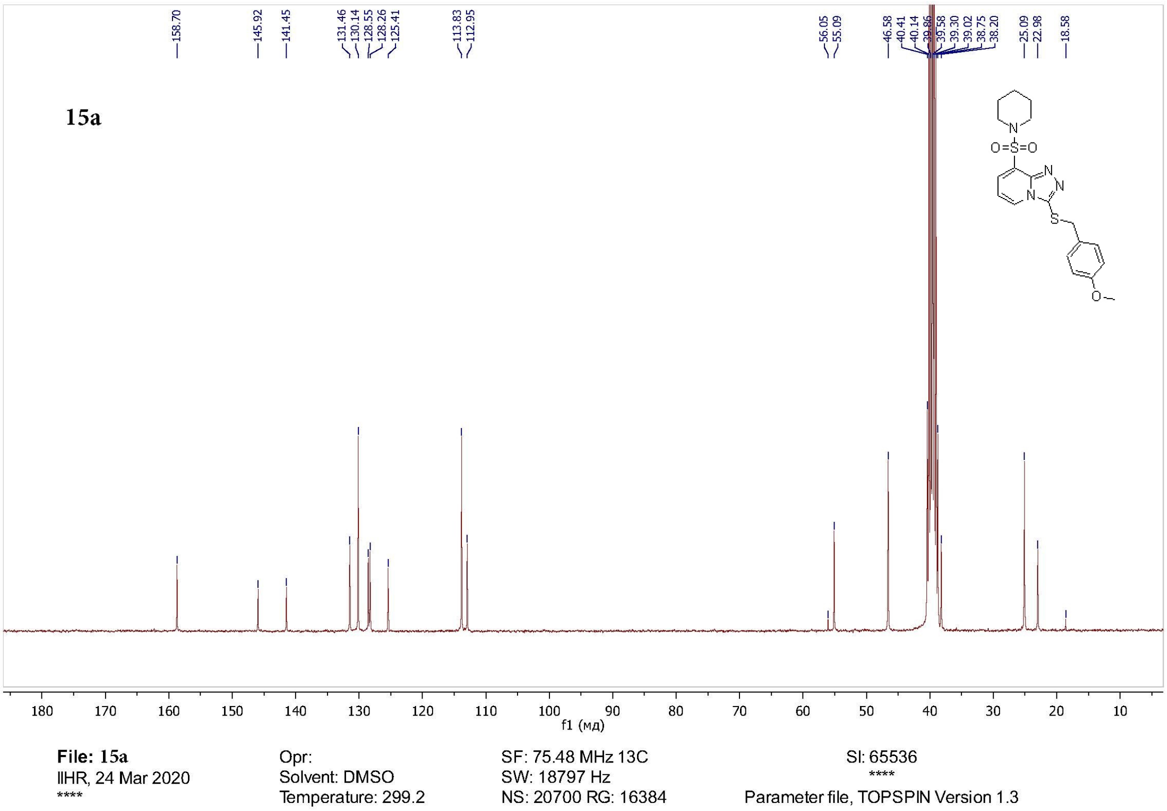

Figure S102. ^{13}C NMR spectrum (75 MHz, DMSO-d6) of 3-(4-methoxybenzylthio)-8-(piperidin-1-ylsulfonyl)-[1,2,4]triazolo[4,3-a]pyridine **15a**.

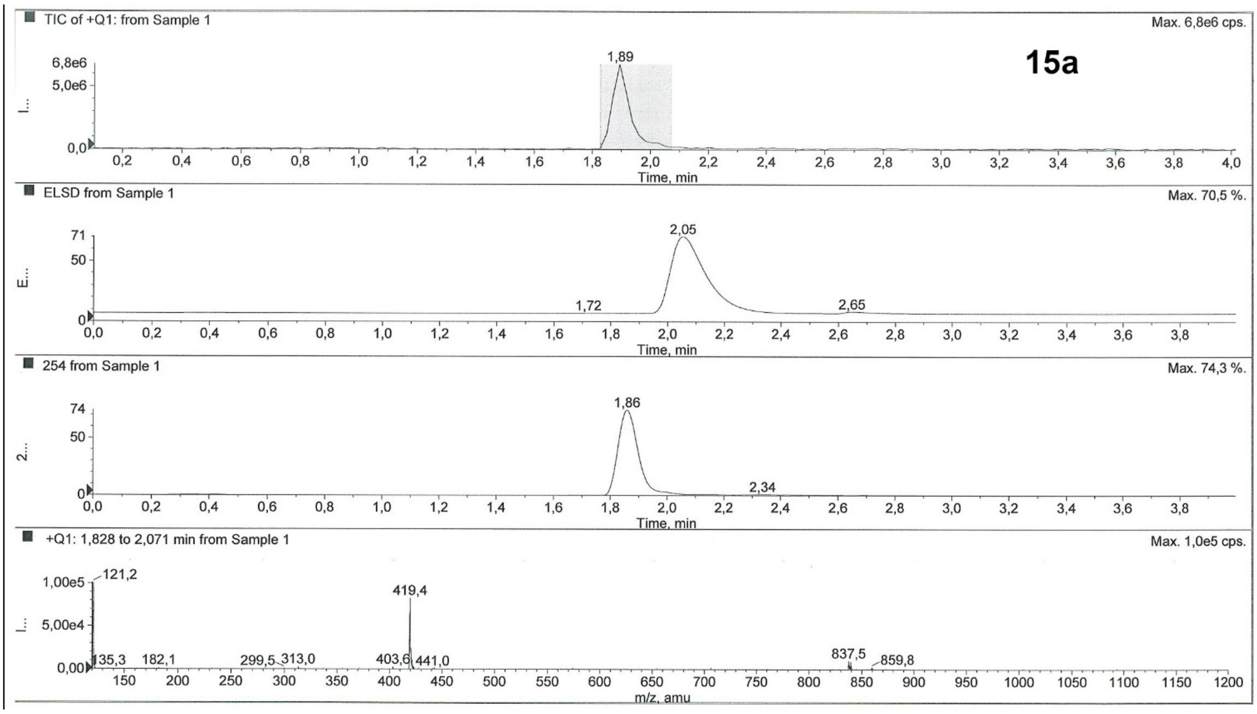

Figure S103. LC/MS data for 3-(4-methoxybenzylthio)-8-(piperidin-1-ylsulfonyl)-[1,2,4]triazolo[4,3-a]pyridine **15a**.

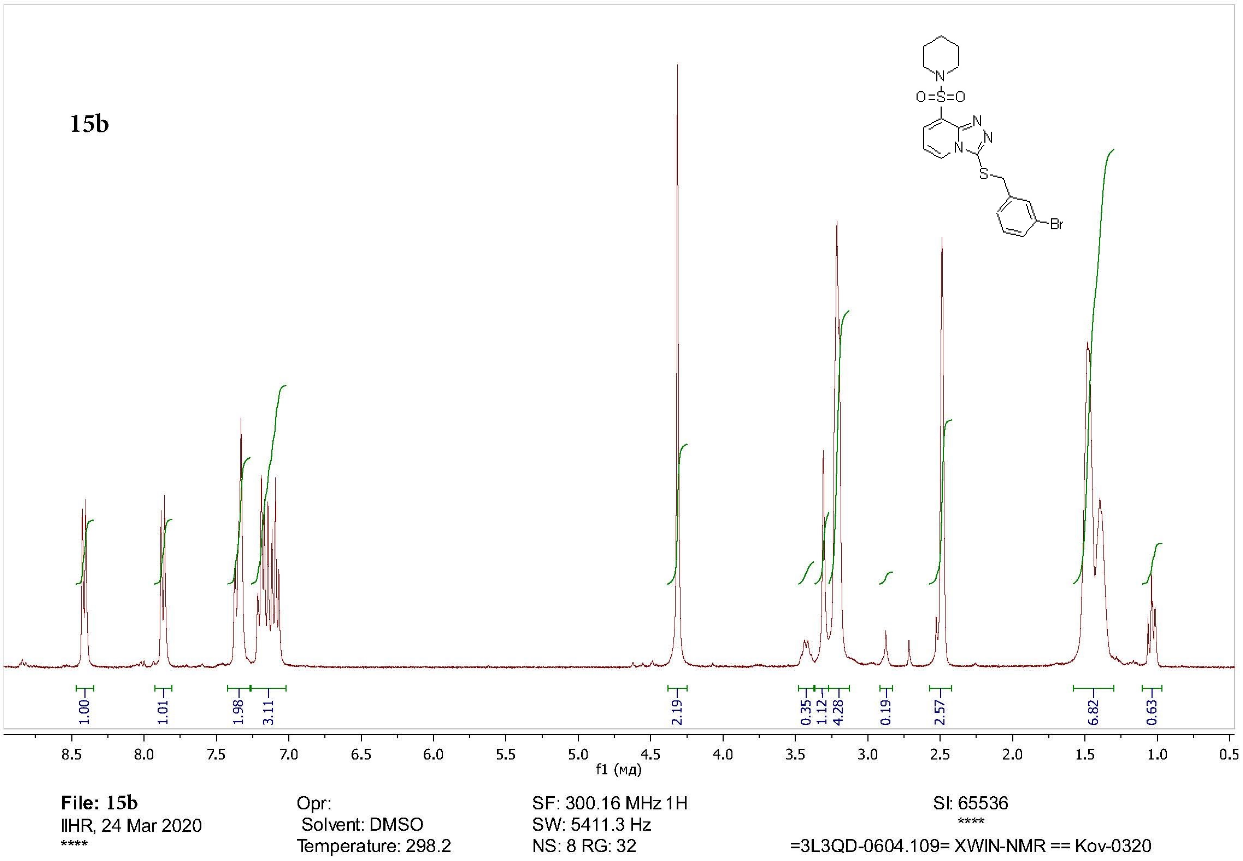

Figure S104. ^{1}H NMR spectrum (300 MHz, DMSO-d6) of 3-(3-bromobenzylthio)-8-(piperidin-1-ylsulfonyl)-[1,2,4]triazolo[4,3-a]pyridine **15b**.

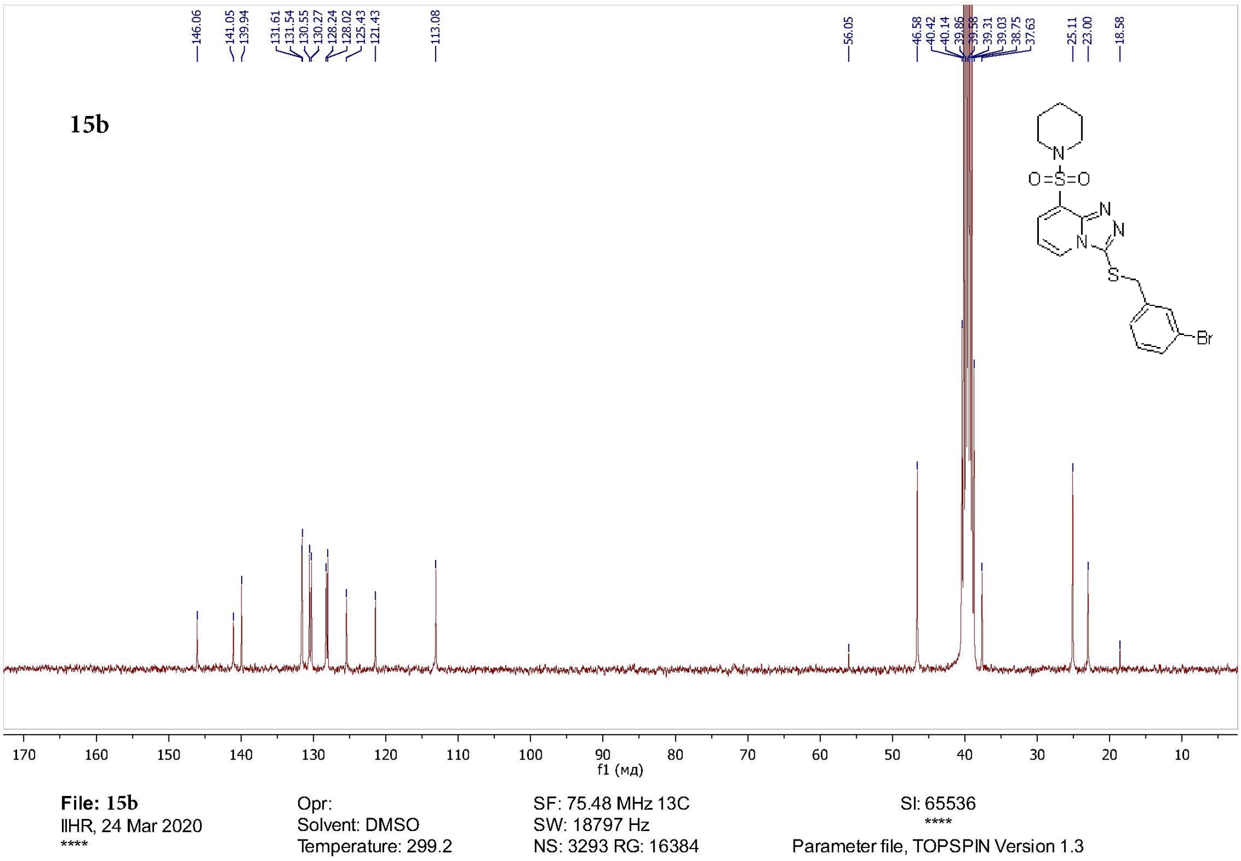

Figure S105. ^{13}C NMR spectrum (75 MHz, DMSO-d6) of 3-(3-bromobenzylthio)-8-(piperidin-1-ylsulfonyl)-[1,2,4]triazolo[4,3-a]pyridine **15b**.

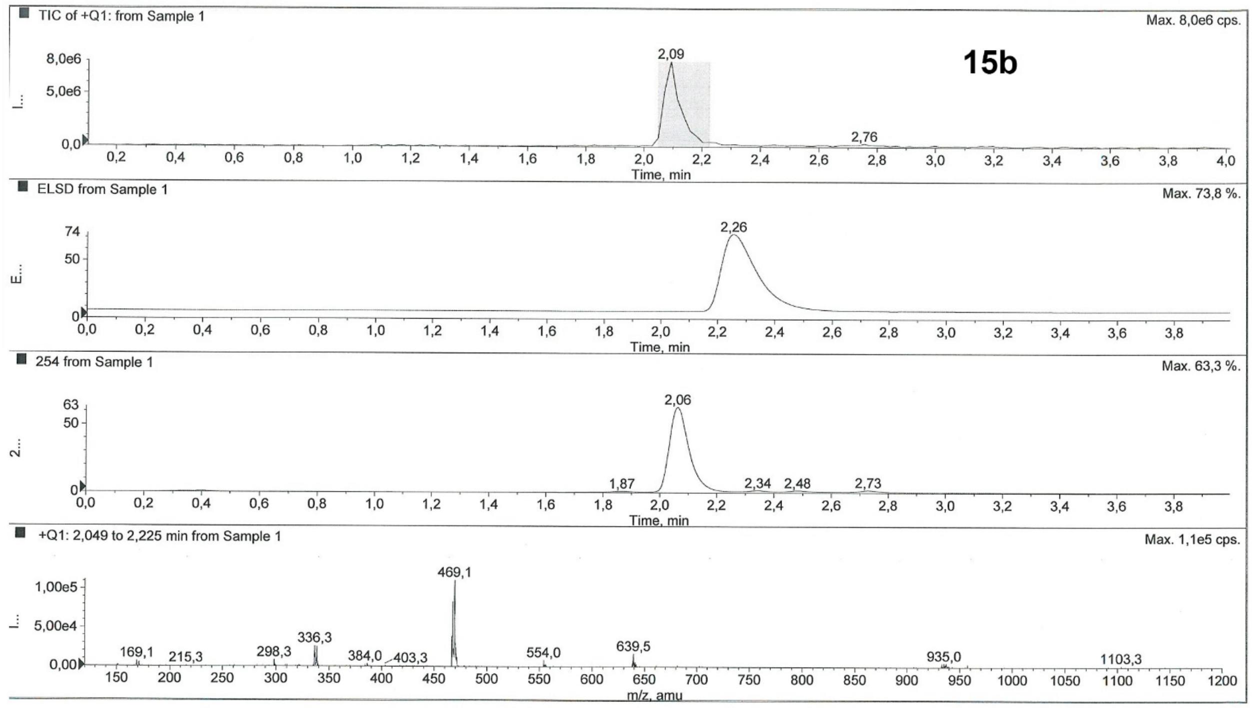

Figure S106. LC/MS data for 3-(3-bromobenzylthio)-8-(piperidin-1-ylsulfonyl)-[1,2,4]triazolo[4,3-a]pyridine **15b**.

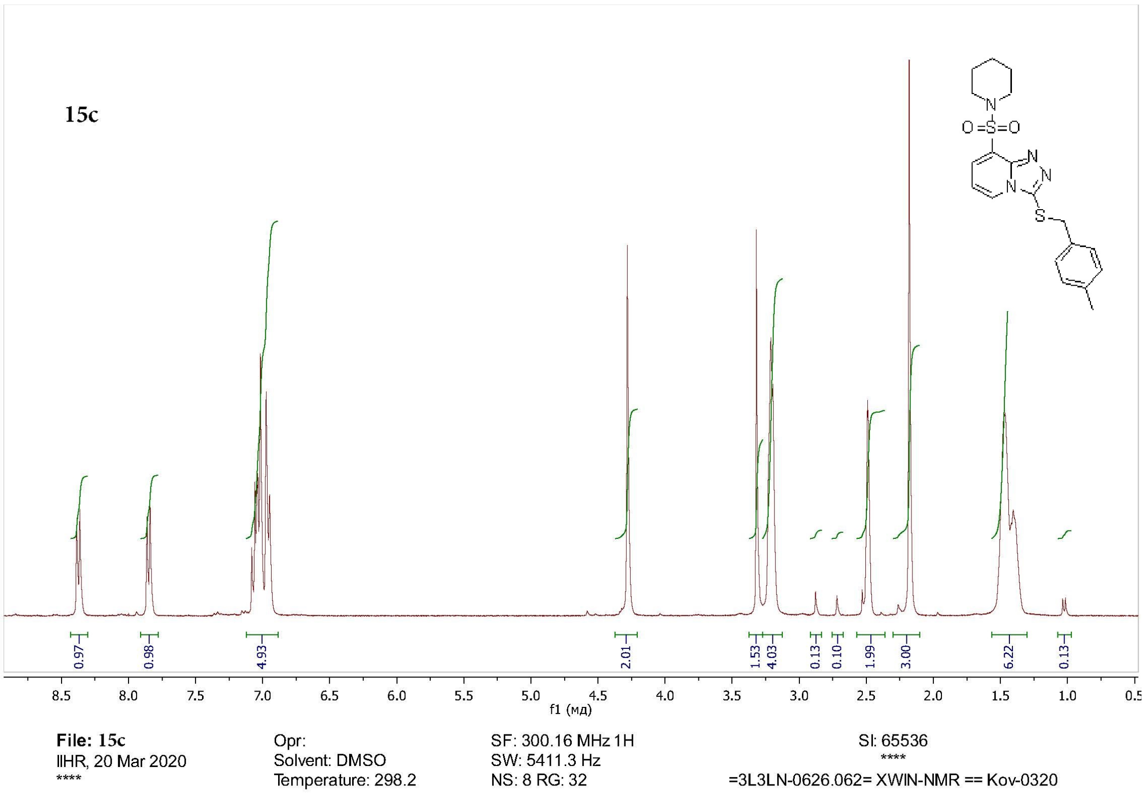

Figure S107. ^{1}H NMR spectrum (300 MHz, DMSO-d6) of 3-(4-methylbenzylthio)-8-(piperidin-1-ylsulfonyl)-[1,2,4]triazolo[4,3-a]pyridine **15c**.

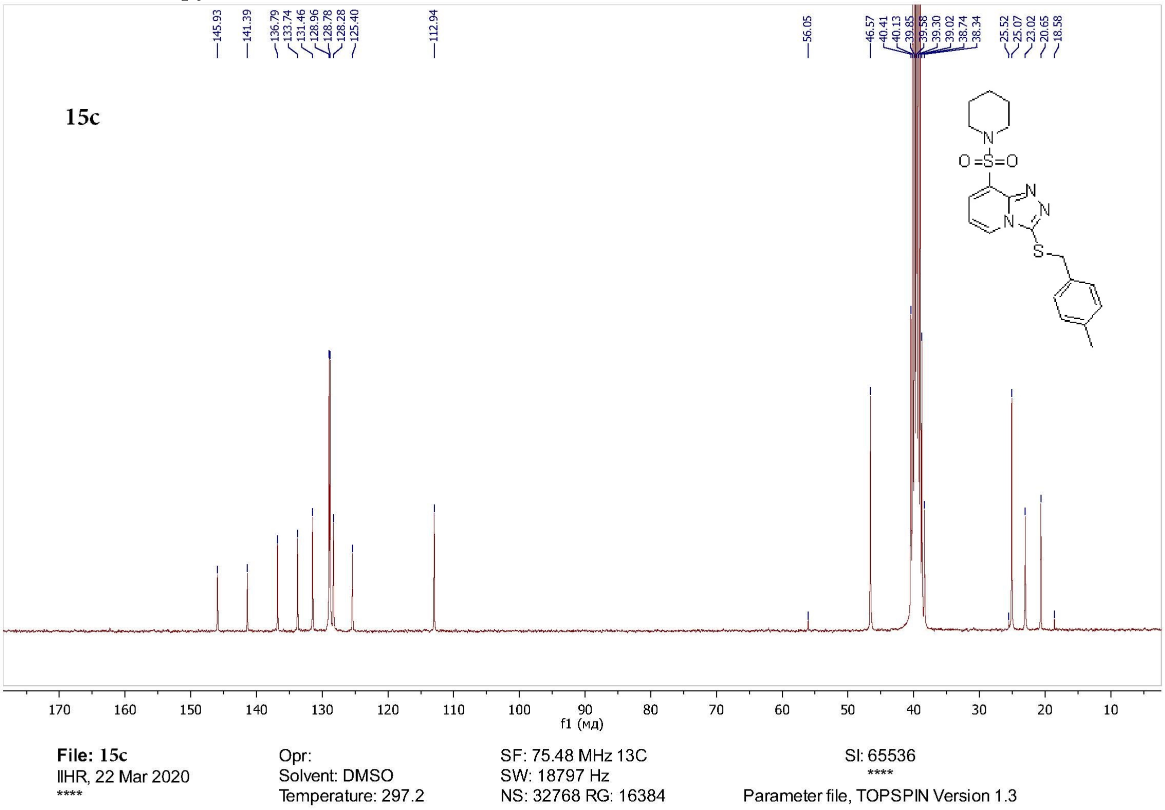

Figure S108. ^{13}C NMR spectrum (75 MHz, DMSO-d6) of 3-(4-methylbenzylthio)-8-(piperidin-1-ylsulfonyl)-[1,2,4]triazolo[4,3-a]pyridine **15c**.

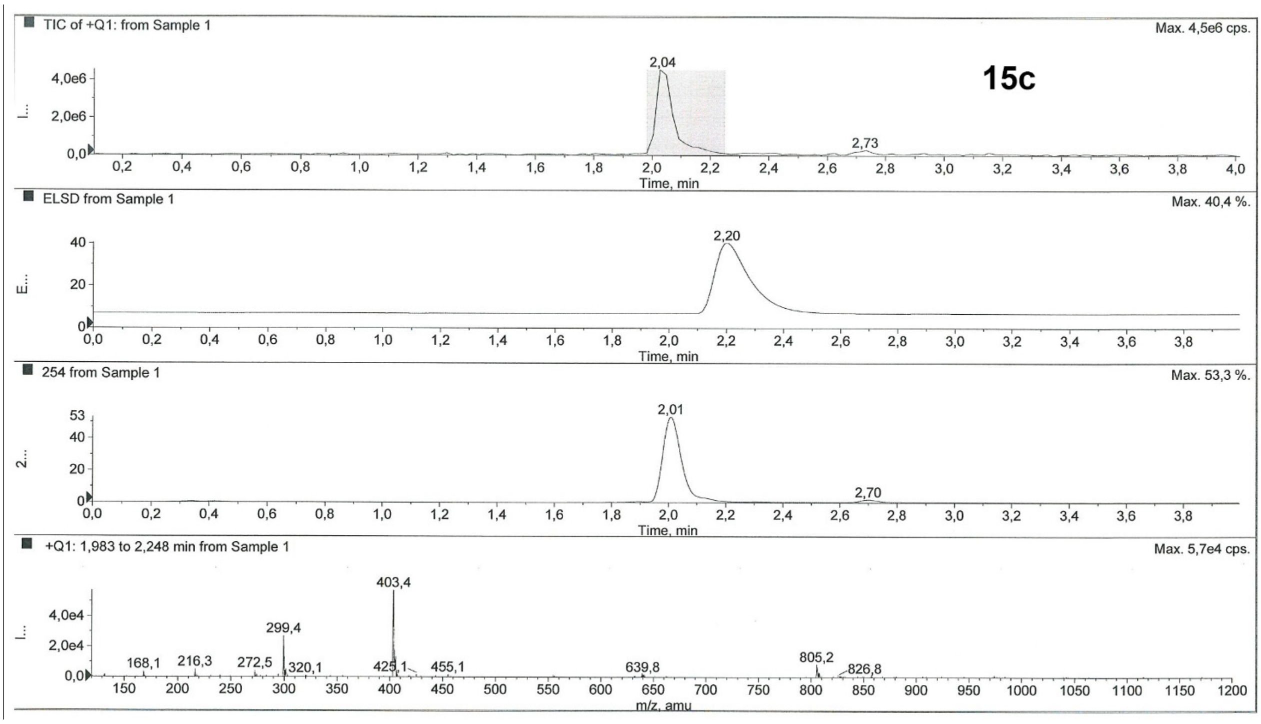

Figure S109. LC/MS data for 3-(4-methylbenzylthio)-8-(piperidin-1-ylsulfonyl)-[1,2,4]triazolo[4,3-a]pyridine **15c**.

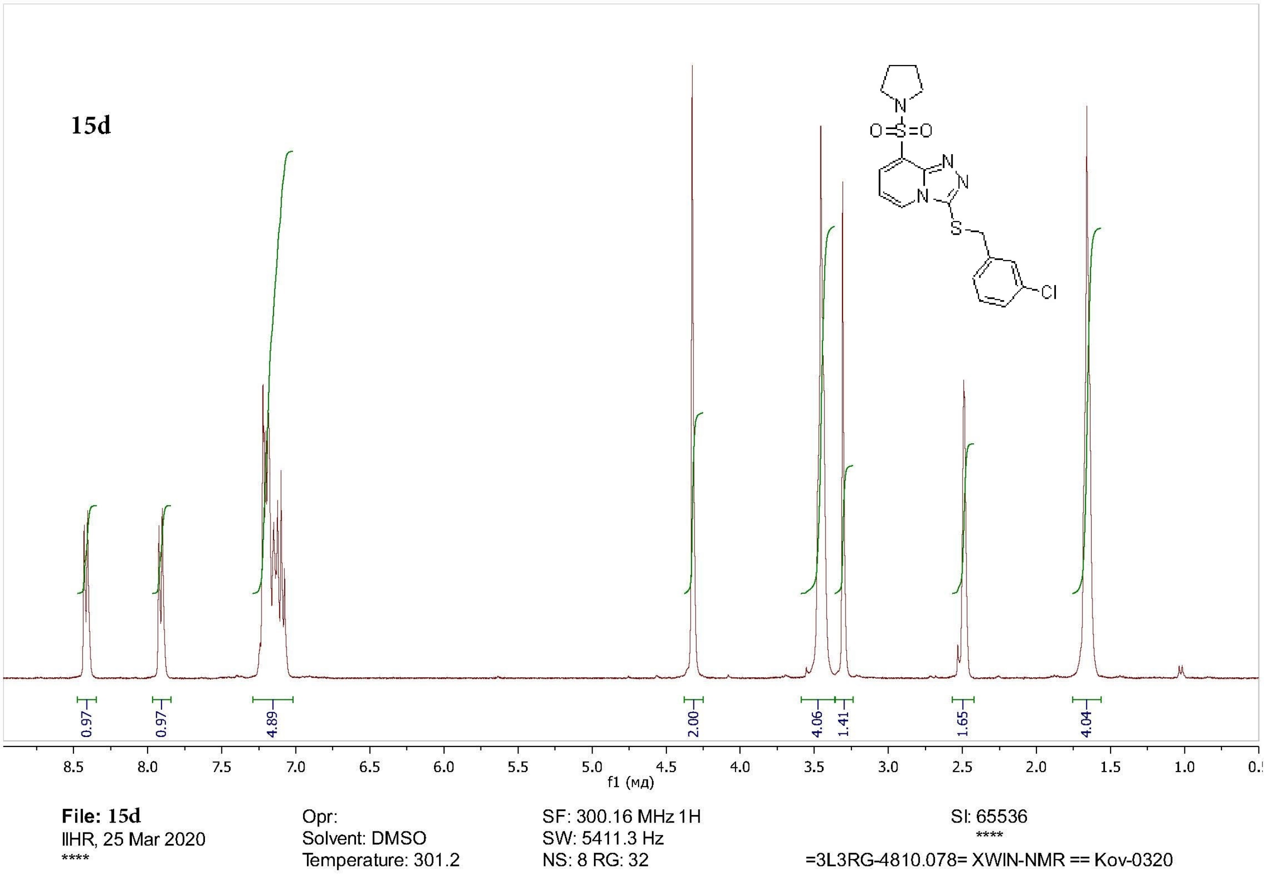

Figure S110. ^{1}H NMR spectrum (300 MHz, DMSO-d6) of 3-(3-chlorobenzylthio)-8-(pyrrolidin-1-ylsulfonyl)-[1,2,4]triazolo[4,3-a]pyridine **15d**.

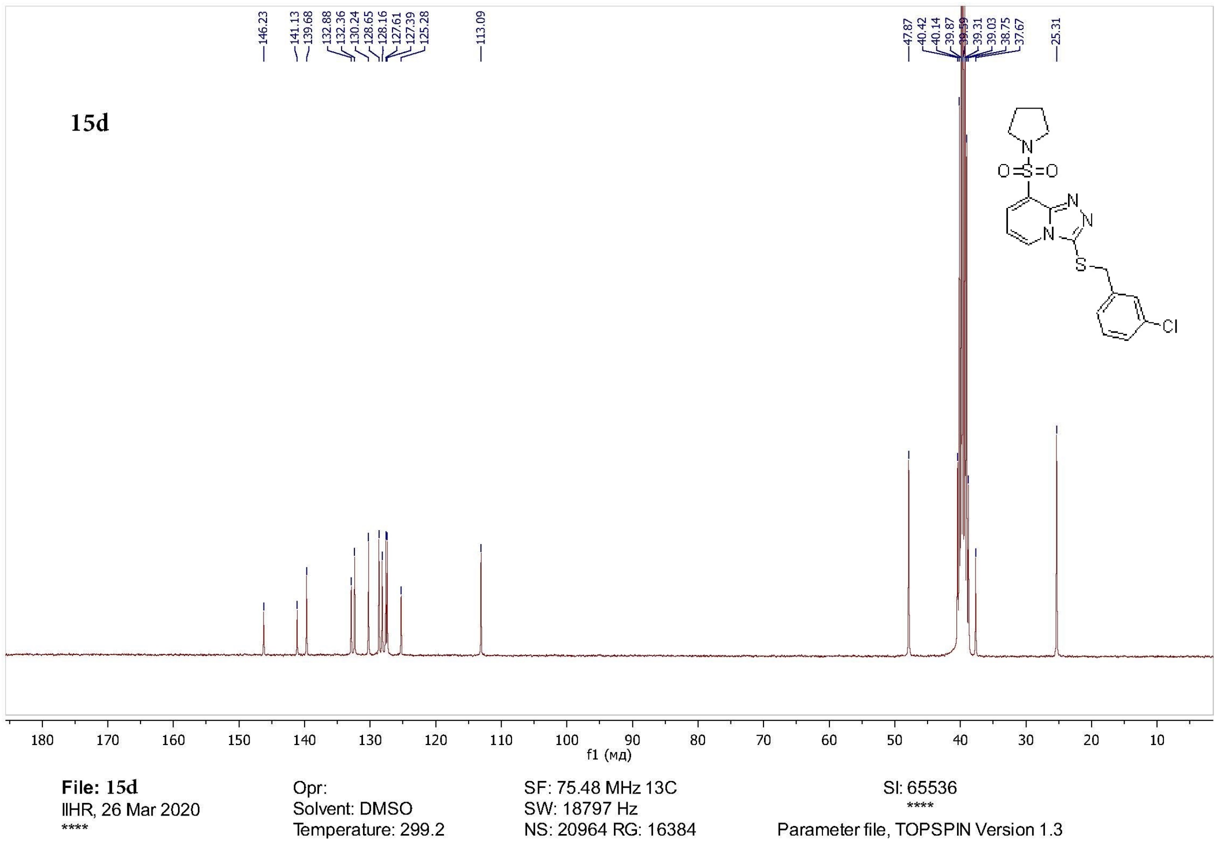

Figure S111. ^{13}C NMR spectrum (75 MHz, DMSO-d6) of 3-(3-chlorobenzylthio)-8-(pyrrolidin-1-ylsulfonyl)-[1,2,4]triazolo[4,3-a]pyridine **15d**.

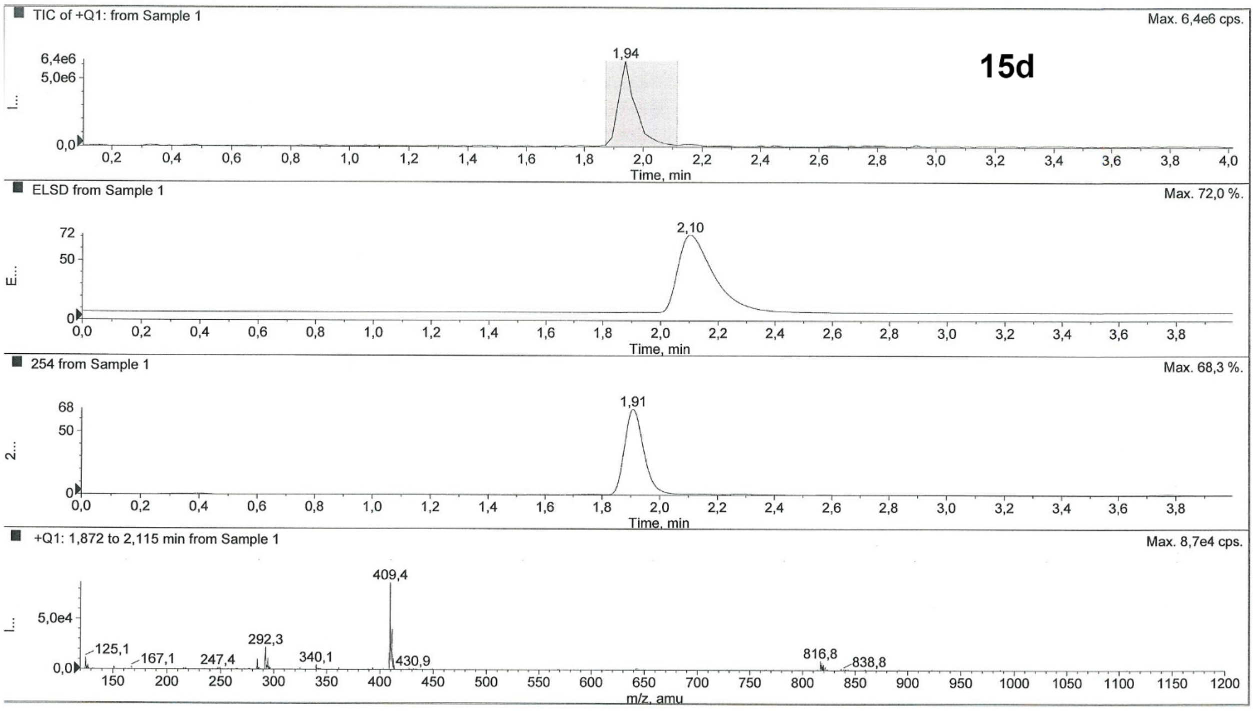

Figure S112. LC/MS data for 3-(3-chlorobenzylthio)-8-(pyrrolidin-1-ylsulfonyl)-[1,2,4]triazolo[4,3-a]pyridine **15d**.

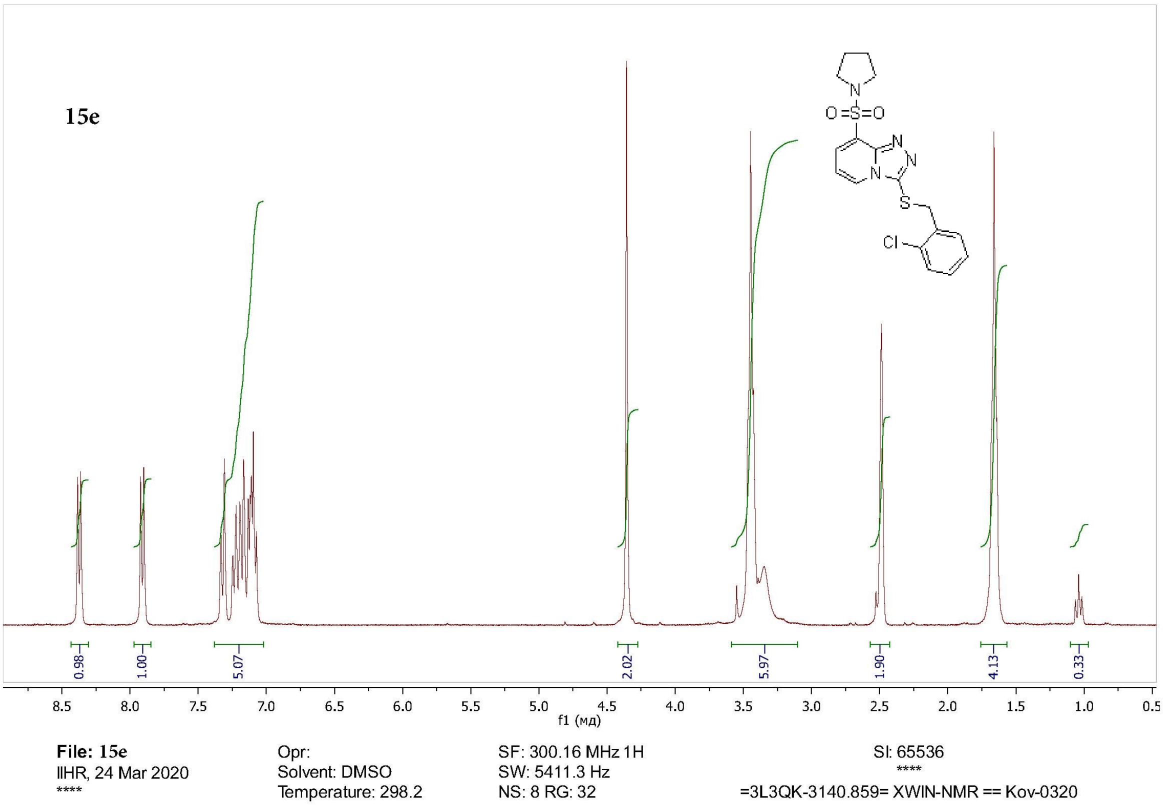

Figure S113. ^{1}H NMR spectrum (300 MHz, DMSO-d6) of 3-(2-chlorobenzylthio)-8-(pyrrolidin-1-ylsulfonyl)-[1,2,4]triazolo[4,3-a]pyridine **15e**.

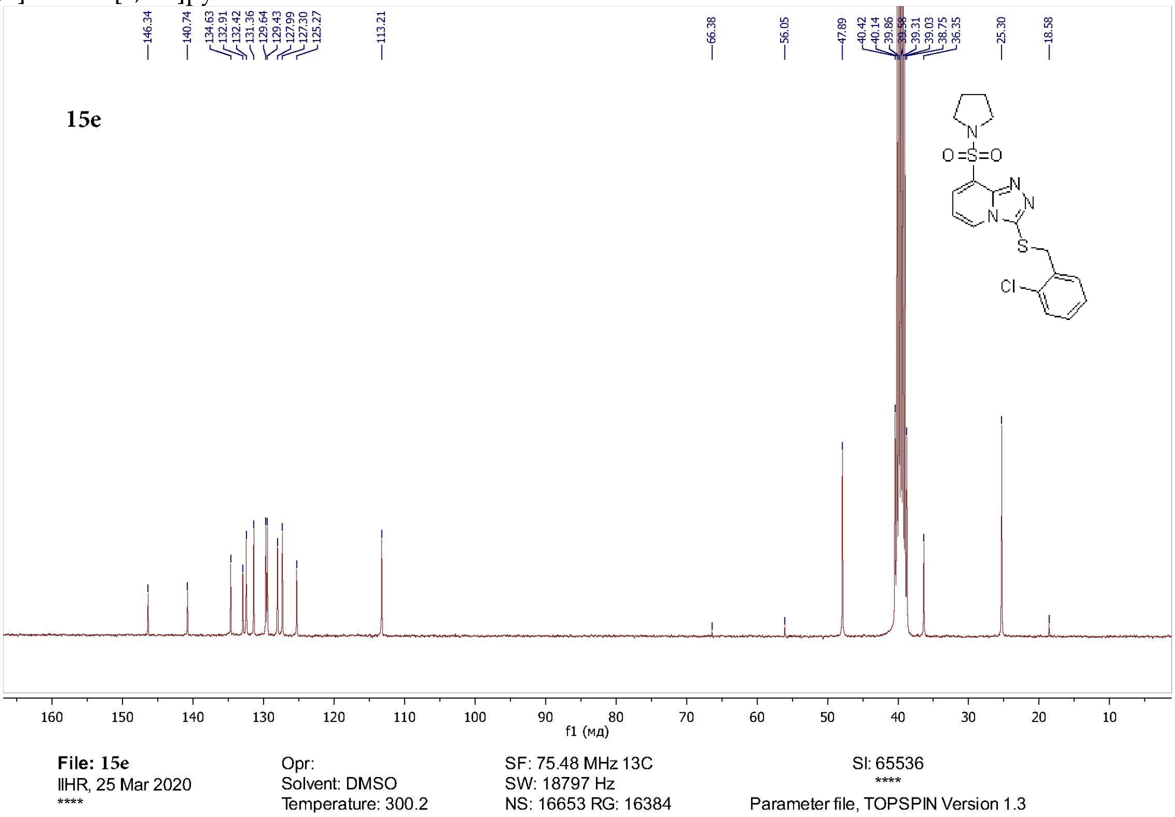

Figure S114. ^{13}C NMR spectrum (75 MHz, DMSO-d6) of 3-(2-chlorobenzylthio)-8-(pyrrolidin-1-ylsulfonyl)-[1,2,4]triazolo[4,3-a]pyridine **15e**.

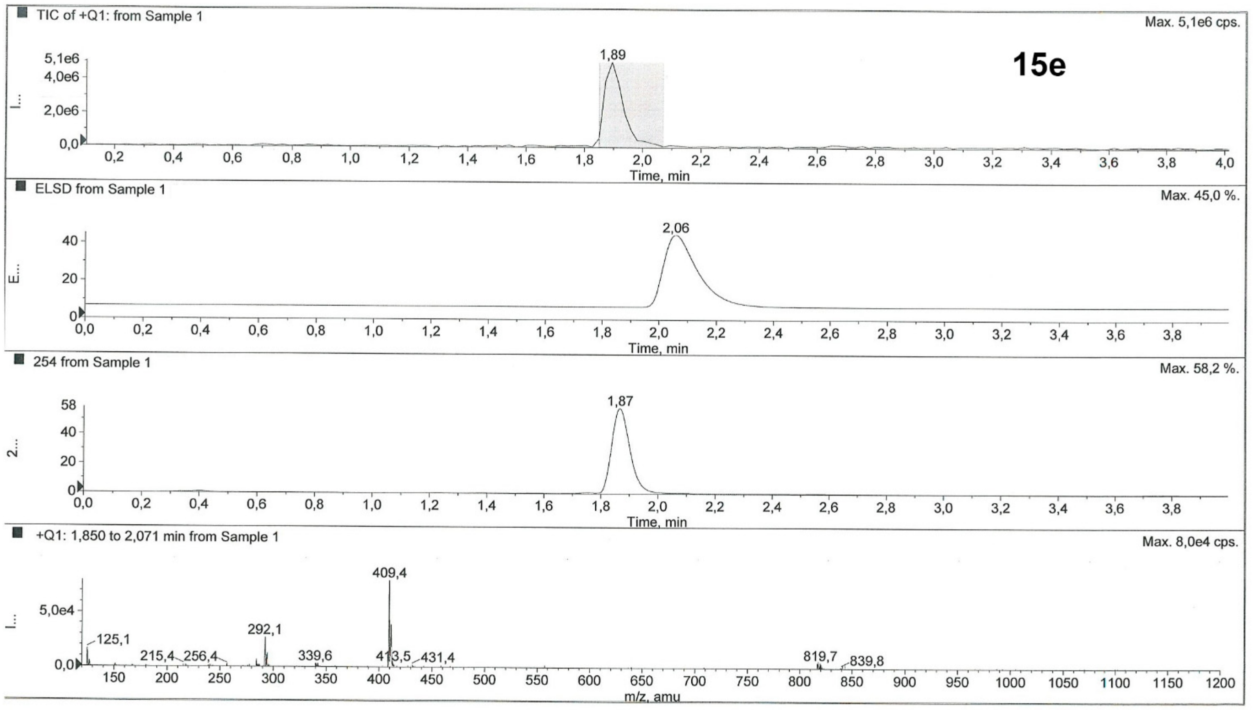

Figure S115. LC/MS data for 3-(2-chlorobenzylthio)-8-(pyrrolidin-1-ylsulfonyl)-[1,2,4]triazolo[4,3-a]pyridine **15e**.

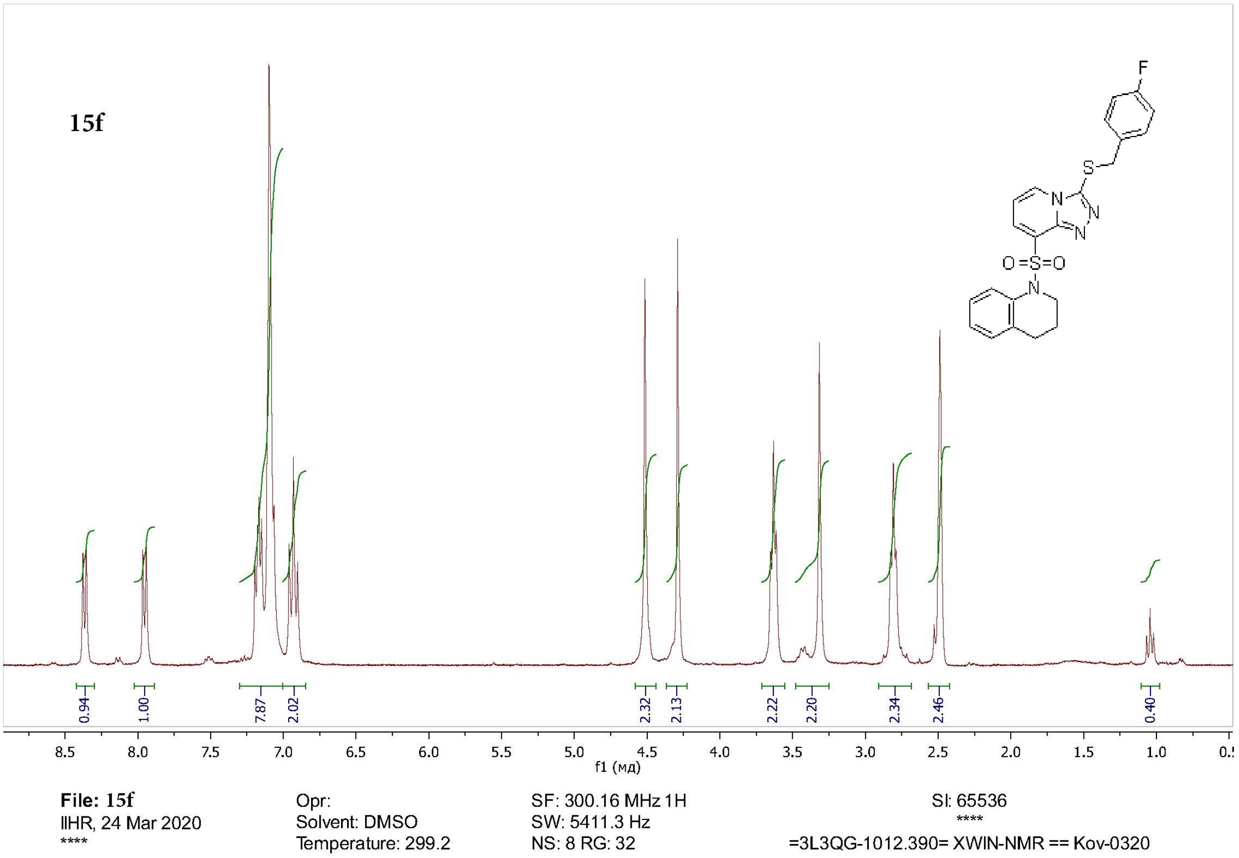

Figure S116. ^{1}H NMR spectrum (300 MHz, DMSO-d6) of 1-(3-(4-fluorobenzylthio)-[1,2,4]triazolo[4,3-a]pyridin-8-ylsulfonyl)-1,2,3,4-tetrahydroquinoline **15f**.

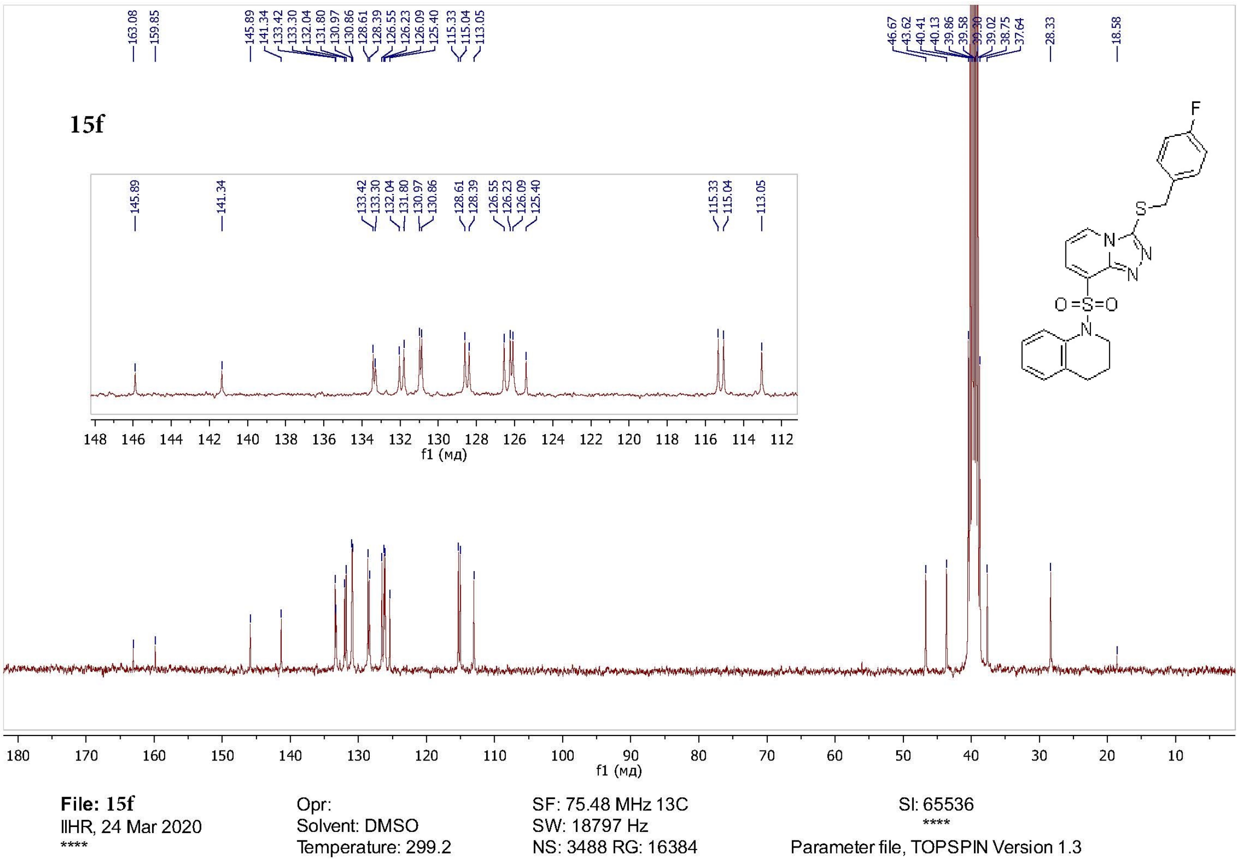

Figure S117. ^{13}C NMR spectrum (75 MHz, DMSO-d6) of 1-(3-(4-fluorobenzylthio)-[1,2,4]triazolo[4,3-a]pyridin-8-ylsulfonyl)-1,2,3,4-tetrahydroquinoline **15f**.

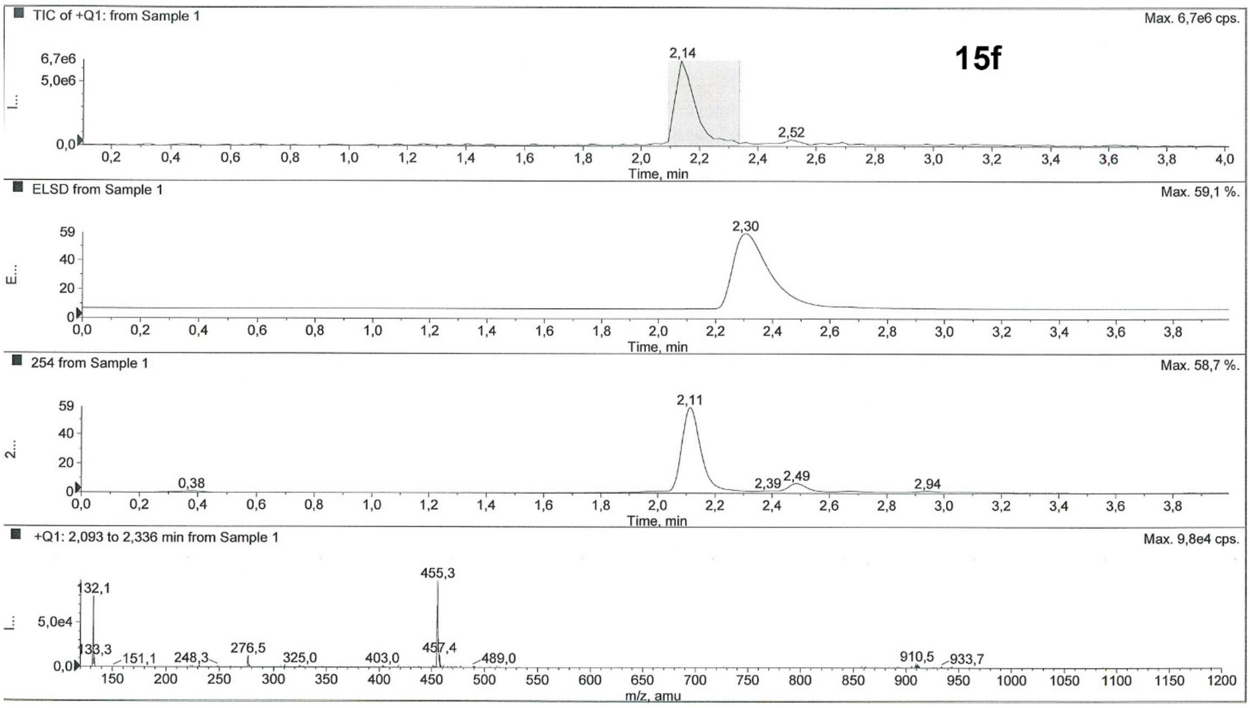

Figure S118. LC/MS data for 1-(3-(4-fluorobenzylthio)-[1,2,4]triazolo[4,3-a]pyridin-8-ylsulfonyl)-1,2,3,4-tetrahydroquinoline **15f**.
